# Abundant Genetic Overlap between Blood Lipids and Immune-Mediated Diseases Indicates Shared Molecular Genetic Mechanisms

**DOI:** 10.1371/journal.pone.0123057

**Published:** 2015-04-08

**Authors:** Ole A. Andreassen, Rahul S. Desikan, Yunpeng Wang, Wesley K. Thompson, Andrew J. Schork, Verena Zuber, Nadezhda T. Doncheva, Eva Ellinghaus, Mario Albrecht, Morten Mattingsdal, Andre Franke, Benedicte A. Lie, Ian Mills, Pål Aukrust, Linda K. McEvoy, Srdjan Djurovic, Tom H. Karlsen, Anders M. Dale

**Affiliations:** 1 NORMENT, KG Jebsen Centre for Psychosis Research, Institute of Clinical Medicine, University of Oslo and Division of Mental Health and Addiction, Oslo University Hospital, 0407 Oslo, Norway; 2 Department of Psychiatry, University of California San Diego, La Jolla, CA 92093, United States of America; 3 Multimodal Imaging Laboratory, University of California San Diego, La Jolla, CA 92093, United States of America; 4 Department of Radiology, University of California San Diego, La Jolla, CA 92093, United States of America; 5 Cognitive Sciences Graduate Program, University of California San Diego, La Jolla, CA 92093, United States of America; 6 Center for Human Development, University of California San Diego, La Jolla, CA 92093, United States of America; 7 Centre for Molecular Medicine Norway, Nordic EMBL Partnership, University of Oslo and Oslo University Hospital, 0407 Oslo, Norway; 8 Max Planck Institute for Informatics, 66123 Saarbrücken, Germany; 9 Institute of Clinical Molecular Biology, Christian-Albrechts-University of Kiel, 24118 Kiel, Germany; 10 Department of Bioinformatics, Institute of Biometrics and Medical Informatics, University Medicine Greifswald, 17475 Greifswald, Germany; 11 Institute for Knowledge Discovery, Graz University of Technology, 8010 Graz, Austria; 12 BioTechMed-Graz, 8010 Graz, Austria; 13 Sørlandet Hospital, 3000 Kristiansand, Norway; 14 Department of Medical Genetics, University of Oslo and Oslo University Hospital, 0407 Oslo, Norway; 15 Department of Cancer Prevention, Institute of Cancer Research and Department of Urology, Oslo University Hospital, 0407 Oslo, Norway; 16 K.G.Jebsen Inflammation Research Centre, Research Institute of Internal Medicine, Division of Cancer Medicine, Surgery and Transplantation, Oslo University Hospital Rikshospitalet, 0407 Oslo, Norway; 17 Section of Clinical Immunology and Infectious Diseases, Oslo University Hospital, 0407 Oslo Norway; 18 Division of Gastroenterology, Institute of Medicine, University of Bergen, 5000 Bergen, Norway; 19 Norwegian PSC Research Center, Department of Transplantation Medicine, Division of Cancer Medicine, Surgery and Transplantation, Oslo University Hospital Rikshospitalet, 0407 Oslo, Norway; 20 Department of Neurosciences, University of California San Diego, La Jolla, CA 92093, United States of America

## Abstract

Epidemiological studies suggest a relationship between blood lipids and immune-mediated diseases, but the nature of these associations is not well understood. We used genome-wide association studies (GWAS) to investigate shared single nucleotide polymorphisms (SNPs) between blood lipids and immune-mediated diseases. We analyzed data from GWAS (n~200,000 individuals), applying new False Discovery Rate (FDR) methods, to investigate genetic overlap between blood lipid levels [triglycerides (TG), low density lipoproteins (LDL), high density lipoproteins (HDL)] and a selection of archetypal immune-mediated diseases (Crohn’s disease, ulcerative colitis, rheumatoid arthritis, type 1 diabetes, celiac disease, psoriasis and sarcoidosis). We found significant polygenic pleiotropy between the blood lipids and all the investigated immune-mediated diseases. We discovered several shared risk loci between the immune-mediated diseases and TG (n = 88), LDL (n = 87) and HDL (n = 52). Three-way analyses differentiated the pattern of pleiotropy among the immune-mediated diseases. The new pleiotropic loci increased the number of functional gene network nodes representing blood lipid loci by 40%. Pathway analyses implicated several novel shared mechanisms for immune pathogenesis and lipid biology, including glycosphingolipid synthesis (e.g. *FUT2*) and intestinal host-microbe interactions (e.g. *ATG16L1*). We demonstrate a shared genetic basis for blood lipids and immune-mediated diseases independent of environmental factors. Our findings provide novel mechanistic insights into dyslipidemia and immune-mediated diseases and may have implications for therapeutic trials involving lipid-lowering and anti-inflammatory agents.

## Introduction

Plasma concentrations of low-density lipoprotein (LDL) cholesterol, high-density lipoprotein (HDL) cholesterol and triglycerides (TG) are heritable risk factors for cardiovascular disease and important pharmacological targets for prevention strategies. Several lines of evidence suggest a relationship between lipid biology and the immune system. For example, clinical and epidemiological data suggest an association between blood lipid levels and immune-mediated diseases, including Crohn’s disease (CD), ulcerative colitis (UC), rheumatoid arthritis (RA), type 1 diabetes (T1D), celiac disease (CeD), psoriasis (PSOR) and sarcoidosis (SARC)[1–5]. In addition to diet and other environmental factors, it is possible that the increased occurrence of dyslipidemia and subsequent atherosclerosis in patients with immune-mediated diseases could reflect genetically determined factors influencing both lipid metabolism and immune-mediated disorders that cannot be elucidated by observational epidemiological and clinical studies.

Genetic epidemiology approaches offer great promise for delineating the underlying basis of shared phenotypic correlations[6]. Traditional genome wide association studies (GWAS) have provided valuable insights into the role of biologic pathways in disease pathogenesis and identified several loci in CD[7], UC[8], RA[9], T1D[10], CeD[11], PSOR[12], and SARC[13], as well as in regulation of TG, HDL and LDL cholesterol[14]. Combining GWAS data from various phenotypes, it is now feasible to determine the association of single nucleotide polymorphisms (SNPs) with more than one phenotype (genetic pleiotropy)[15]. This may enable identification of genetic factors responsible for shared disease mechanisms[15–21].

This genetic epidemiology approach may be useful for elucidating the genetic basis of the clinically observed association between dyslipidemia, immune-mediated diseases and inflammation. Abnormal lipid profiles may occur in individuals before the onset of immune-mediated diseases, suggesting a potential pathogenic relationship[22]. Specifically, altered lipid levels and functions have been implicated in several immune-mediated diseases and inflammatory responses[23], including anti-inflammatory effects of HDL cholesterol and inflammatory responses of modified LDL cholesterol[24]. Conversely, inflammation has been found to modify lipid levels and lipoprotein composition at least partly through modulation of scavenger receptors, cholesterol efflux mechanisms and various enzymes in lipid metabolism such as lipoprotein lipase. This bidirectional interaction between lipid biology and inflammation is a major pathogenic feature of atherosclerosis and related metabolic disorders[25] and is accelerated in many autoimmune and inflammatory disorders[26].

Here, we applied a recently-developed genetic epidemiology framework [15–21] to systematically interrogate the potentially shared genetic basis between immune-mediated diseases (CD, UC, RA, T1D, CeD, PSOR, SARC) and blood lipid levels (TG, LDL, HDL). Using our new methodology, we have previously identified genetic pleiotropy (defined as SNPs associated with more than one phenotype) between a number of diseases/phenotypes and identified variants that significantly increase the risk of schizophrenia[16,17,19], bipolar disorder[19], hypertension[18], and primary sclerosing cholangitis[21] and prostate cancer[20].

## Materials and Methods

### Ethics Statement

All the GWAS of the different phenotypes investigated in the current study were approved by the local Ethic committee for each of the sub-samples included. Further, the Norwegian IRB for the South East region has evaluated the current protocol and found that no additional IRB approval needed, because of no individual data was used.

### Participant Samples

We obtained GWAS results in the form of summary statistic p-values from the original publications or from collaborating GWAS consortia (Table A in S1 File)[7–14]. We utilized summary statistics (p-values and z-scores) for conditional and conjunction FDR analyses. Details of the inclusion criteria and phenotype characteristics of the different GWAS are described in the original publications[7–14,27]. We corrected all p-values for inflation using a recently developed genomic control procedure[15,16,19]. The blood lipid GWAS of TG, HDL and LDL involved the same population. However, in the current analyses, we did not examine pleiotropy among the lipid traits. Approximately 2,000 to 3,000 overlapping controls were present between the RA, UC and CD GWAS. We used the recently published blood lipid GWAS[28] (n = 188,577) as confirmation sample, and included the cigarettes per day (CPD) GWAS[29] (n = 74,053) as a negative control.

### Statistical Analyses

#### Quantifying enrichment based on conditional Q-Q Plots

A common method for visualizing the enrichment of statistical association relative to that expected under the global null hypothesis is through Q-Q plots. Q-Q plots present nominal p-values obtained from GWAS summary statistics as a function of empirical p-values expected under the global null hypothesis. We constructed *conditional* Q-Q plots where we display the distribution of summary statistics for the primary trait conditional on different levels of significance in a secondary trait (i.e. associated with a immune mediated disease). Associations in the primary trait were conditioned on a p-value threshold in the secondary traits, i.e—log_10_ p-value >1, >2, and >3. If enrichment of the blood lipid trait is present among SNPs that are significantly associated with the immune trait (pleiotropic enrichment), there will be successive leftward deflections in the conditional Q-Q plot as levels of association with the immune trait increase[15–21]. In Figures A-C in S1 File we show Q-Q plots for the blood lipids GWAS conditioning on the significance in the immune-mediated diseases (CD, UC, RA, T1D, CeD, PSOR, SARC). To control for spurious enrichment due to large LD blocks, we calculated FDR after random pruning (see Below).

#### Detection of shared associations using conjunction FDR

For the detection of pleiotropic associations we used a genetic epidemiology framework based on the conjunction false discovery rate (conjFDR). We have previously used this approach to identify shared genetic risk loci between psychiatric, cardiovascular, immune-related diseases and cancer[15–20]. Conjunction FDR, denoted by FDR_trait1& trait2_ is defined as the posterior probability that a SNP is null for either phenotype or both simultaneously, given the p-values for both traits are as small or smaller than the observed p-values[15–20]. We obtained a conservative estimate of conjunction FDR via the conditional FDR (Tables B-D in S1 File). The conditional FDR, denoted by FDR_trait1|trait2_, is defined as the posterior probability that a given SNP is null for the first trait given that the p-values for both traits are as small or smaller than the observed p-values[15–20]. A conservative estimate of the conjunction FDR FDR_trait1& trait2_ is given by the maximum statistic[30] in taking the maximum of FDR_trait1|trait2_ and FDR_trait2|trait1_. While the conditional FDR can be used to reorder SNPs based on the additional information provided by the co-morbid secondary traits, the conjunction FDR pinpoints pleiotropic loci, since a low conjunction FDR is only possible if there is an association with the two traits of interest jointly.

**Fig 1:**
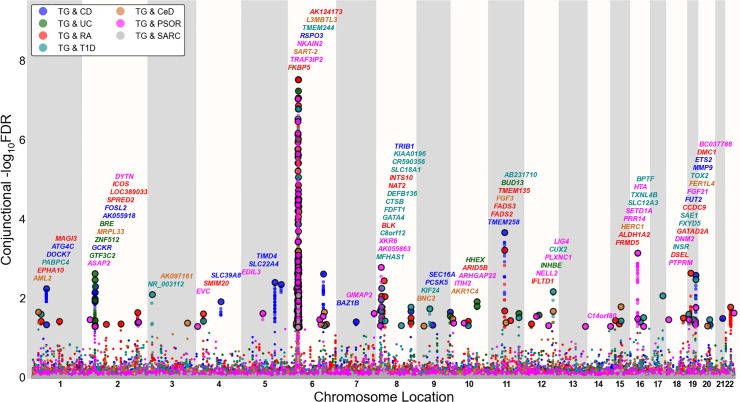
‘Conjunctional FDR Manhattan plot’ of conjunctional (FDR<0.05) values for triglycerides. Conjunctional—log_10_(FDR) values for triglycerides (TG) and Crohn’s Disease (CD), ulcerative colitis (UC), rheumatoid arthritis (RA), type 1 diabetes (T1D), celiac disease (CeD), psoriasis (PSOR) and G) sarcoidosis (SARC) were plotted along their chromosome locations. SNPs with conjunctional FDR < 0.05 (i.e.,—log_10_ FDR > 1.3) are shown with enlarged data points. A black circle around the enlarged data points indicates the most significant SNP in each LD block and this SNP was annotated with the closest gene which is listed above the symbols in each locus, except for the Major Histocompatibility Complex (MHC) region on chromosome 6. The figure shows the localization of 88 TG loci. Details for the associated loci outside of chromosome 6 are shown in Table E in S1 File.

**Fig 2:**
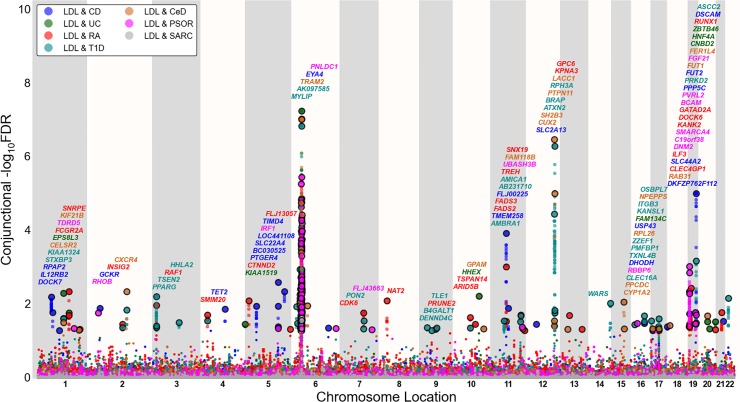
‘Conjunctional FDR Manhattan plot’ of conjunctional (FDR<0.05) values for low density lipoprotein. Conjunctional—log_10_(FDR) values for low density lipoproteins (LDL) cholesterol and Crohn’s Disease (CD), ulcerative colitis (UC), rheumatoid arthritis (RA), type 1 diabetes (T1D), celiac disease (CeD), psoriasis (PSOR) and G) sarcoidosis (SARC) were plotted along their chromosome locations. SNPs with conjunctional FDR < 0.05 (i.e.,—log_10_ FDR > 1.3) are shown with enlarged data points. A black circle around the enlarged data points indicates the most significant SNP in each LD block and this SNP was annotated with the closest gene which is listed above the symbols in each locus, except for the Major Histocompatibility Complex (MHC) region on chromosome 6. The figure shows the localization of 87 LDL loci. Details for the associated loci outside of chromosome 6 are shown in Table F in S1 File.

**Fig 3:**
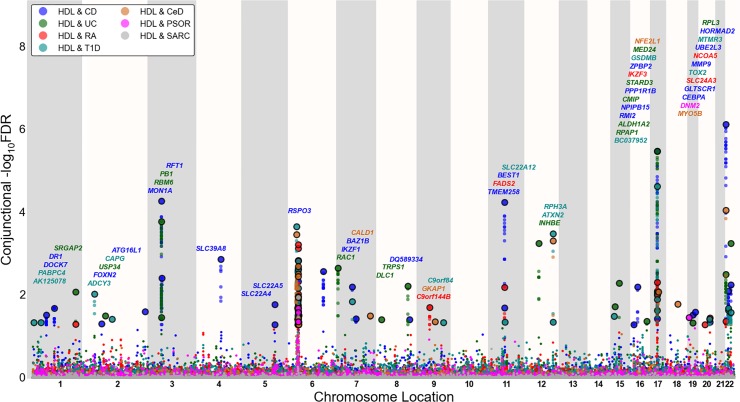
‘Conjunctional FDR Manhattan plot’ of conjunctional (FDR<0.05) values for high density lipoprotein. Conjunctional—log_10_(FDR) values for high density lipoproteins (HDL) cholesterol and Crohn’s Disease (CD), ulcerative colitis (UC), rheumatoid arthritis (RA), type 1 diabetes (T1D), celiac disease (CeD), psoriasis (PSOR) and G) sarcoidosis (SARC) were plotted along their chromosome locations. SNPs with conjunctional FDR < 0.05 (i.e.,—log_10_ FDR > 1.3) are shown with enlarged data points. A black circle around the enlarged data points indicates the most significant SNP in each LD block and this SNP was annotated with the closest gene which is listed above the symbols in each locus, except for the Major Histocompatibility Complex (MHC) region on chromosome 6. The figure shows the localization of 52 HDL loci. Details for the associated loci outside of chromosome 6 are shown in Table G in S1 File.

To visualize the localization of the pleiotropic genetic variants associated with both blood lipids and immune-mediated diseases, we used a ‘Conjunction FDR Manhattan plot’, showing all SNPs with a significant conjunction FDR within an LD block in relation to their chromosomal location. As illustrated in Figs 1, 2 and 3, the enlarged data points represent the significant SNPs (FDR _trait1& trait2_ < 0.05), whereas the small points represent the non-significant SNPs. All SNPs without pruning are shown, and the strongest signal in each LD block is encircled in black. The strongest signal was identified after ranking all SNPs based on the conjunction FDR and removed SNPs in LD r^2^ > 0.2 with any higher ranked SNP (Figs 1, 2 and 3 and Figures U-W in S1 File).

We further evaluated the clustering of the different phenotypes by constructing color-coded heat maps to illustrate the number of conjunctional loci across the different diseases and traits. The number of pleiotropic SNPs with conjFDR _trait1& trait2_ < 0.05 were calculated per phenotype pair, and illustrated using a color scale. Thus, colors shown on the diagonal reflect the number of the pleiotropic SNPs for a given lipid parameter and a specific immune-mediated disease. The off-diagonal elements indicate the number of SNPs showing three-way pleiotropy across the given lipid phenotype and a pair of different immune-mediated diseases.

#### Comparison with standard analytical approaches

To further evaluate our method, we compared our results to findings from conventional approaches. In a ‘confirmation analysis’, we calculated the fraction of SNPs identified with our FDR approach (condFDR < 0.01) that remained significant (p < 5 x 10^–8^) in the recently published larger blood lipid GWAS[28] compared with standard GWAS analysis methods (Figure Q in S1 File). We additionally compared the number of pleiotropic SNPs identified by our conjunctional FDR method to those identified by the standard GWAS approach, i.e., SNPs with p value < 5 x 10^–8^ for *both* blood lipids and immune-mediated diseases. Further, we investigated the direction of effect of these ‘pleiotropic’ SNPs by plotting the z-scores between blood lipid and immune-mediated diseases. In Tables H-J in S1 File, we display all loci with a conjunction of FDR_trait1& trait2_ < 0.05 in blood lipids and immune-mediated diseases. The significance threshold of FDR_trait1& trait2_ < 0.05 corresponds to five expected false positives per hundred reported associations. In Tables Q-S in S1 File we show all ‘pleiotropic’ SNPs identified by standard GWAS.

#### Influence of MHC region on enrichment

The major histocompatibility complex (MHC) is recognized as an important factor in the pathology of immune-mediated diseases. To examine the effect of the MHC for the conditional FDR analysis, we examined enrichment after removing the SNPs in the MHC region. We removed all SNPs located in the MHC (location 25652429–33368333) on chromosome 6 and all SNPs in LD with such SNPs (r^2^ > 0.2) then repeated the analyses described above.

#### Monogenic versus polygenic pleiotropy

To further investigate whether the observed pleiotropy between lipids and immune-mediated diseases was driven by one or few large LD blocks we reanalyzed the data using 100 random pruning. At each iteration, one SNP in every LD block was randomly selected and the empirical cdfs were computed using the corresponding p-values. These 100 CDF’s were averaged and used to generate conditional QQ plots (Figures R-T in S1 File).

#### Network analysis

For the construction of the gene and protein network, we considered the 92 genes (or their gene products) in the previously confirmed 87 blood lipid loci associated with either LDL, HDL or TG[14] as well as the 288 genes (or their gene products) in the novel pleiotropic loci from the present analysis. In the gene network, each network edge represents strong functional similarity of two genes based on their Gene Ontology annotations. The functional similarity is computed by the rfunSim measure, which considers the Biological Process and Molecular Function ontologies of the Gene Ontology[31]. The rfunSim similarity values above the recommended cutoff 0.8 were retrieved from the FunSimMat web service[32]. In the protein network, network edges represent physical protein-protein interactions extracted from the iRefIndex database (release 10.0)[33]. Enriched KEGG pathways [34] were identified for the genes/proteins in the respective networks by the GO-Elite software using the default parameters[35]. The resulting networks were visualized with Cytoscape[36] and the MultiColoredNodes plugin[37].

## Results

### Significant genetic overlap between blood lipids and immune-mediated diseases

The conditional Q-Q plots revealed strong polygenic pleiotropic enrichment between TG and CD, UC, RA, T1D, CeD, PSOR, with somewhat weaker enrichment with SARC (Figure A in S1 File). Similar results were observed for LDL (Figure B in S1 File). A different pattern of enrichment emerged for HDL, where strong pleiotropic enrichment was observed with CD, UC and RA; weaker enrichment with T1D, and little enrichment with CeD, PSOR and SARC (Figure C in S1 File). We also illustrate the high level of polygenic pleiotropic enrichment in blood lipids (TG, LDL, HDL) with the immune-mediated diseases using “Enrichment Plots”[38] (Figures D-F in S1 File). Further, the random pruning analysis showed that the enrichment was not driven by large LD blocks (Figures R-T in S1 File). The lack of enrichment between the blood lipids and CPD (Figure P in S1 File) showed that the polygenic overlap is not general for all phenotypes.

### Shared susceptibility loci for blood lipids and immune-mediated disorders

To provide a comprehensive, unselected map of pleiotropic loci between blood lipids and immune-mediated disorders, we performed a conjunction FDR analysis and constructed conjunction FDR Manhattan plots (Figs 1, 2 and 3). Based on conjFDR < 0.05, we detected independent pleiotropic loci between immune-mediated diseases and TG (n = 88), LDL (n = 87) and HDL (n = 52) after pruning (based on LD r^2^>0.2) excluding loci located in MHC region. The most significant pleiotropic loci (conjFDR < 0.01) for each blood lipid phenotype (non-MHC loci) are listed in Tables E-G in S1 File, and all loci are listed in Tables H-J in S1 File(conjFDR < 0.05). As can be seen from Figs 1, 2 and 3, pleiotropic loci were detected on almost all chromosomes. There were 6 HDL, 6 LDL and 5 TG pleiotropic loci that were associated with several phenotypes (Tables H-J in S1 File). The direction of effect (z-scores) for the SNPs with conjFDR<0.01 are presented in Tables K-M in S1 File. The directionality of effect was fairly consistent across the blood lipids and the different immune-mediated diseases, shown by positive correlations between blood lipids (TG, LDL, HDL) and CD, UC, RA and PSOR and negative correlations between the blood lipids and CeD and SARC (Table N in S1 File).

### Patterns of genetic overlap between blood lipids and immune-mediated diseases

Heat maps of the number of conjunctional loci revealed differences in the pattern of genetic overlap between the three blood lipids and the seven immune-mediated diseases (Figure M in S1 File). LDL showed a number of overlapping SNPs with each of the immune-mediated diseases except for SARC (as can be seen by the squares on the diagonal of Figure M in S1 File). Examination of results off the diagonal elements indicated extensive common association among TG, RA, T1D, CeD, and PSOR. A similar pattern of common SNPs clusters was observed for LDL. HDL, however, showed a different pattern of genetic overlap, primarily involving inflammatory bowel diseases (CD and UC), and to a lesser degree RA and T1D. As was the case with LDL and TG, there was little overlap with SARC.

### Influence of MHC region on enrichment

To test the hypothesis that the observed enrichment pattern between blood lipids and immune-mediated diseases may be driven solely by the MHC region, we examined enrichment after removing SNPs in the MHC region. As illustrated in Figures G, H, J and K in S1 File we found a slight decrease in enrichment between TG and T1D, CeD, PSOR and SARC, and between LDL and RA, CeD and SARC. However, there was almost no reduction in enrichment between HDL and the immune-mediated diseases (Figures I and L in S1 File). Three-way heat maps based on the non-MHC results illustrated that the pleiotropy between HDL and other immune diseases was highly polygenic and not driven by the MHC region, whereas both LDL and TG demonstrated less non-MHC related polygenic pleiotropy (Figure N in S1 File).

### Comparison with conventional approaches

#### Number of conditional loci

To further compare our pleiotropic approach with standard GWAS methods for detecting novel polymorphisms, we evaluated the number of blood lipid-associated loci using conditional FDR. To estimate the number of independent loci, we pruned the significantly associated SNPs and identified 570 gene loci with a significance threshold of conditional FDR < 0.01 (Tables B-D in S1 File), including 166 with TG, 195 with LDL and 209 with HDL. Of these 127 were associated with more than one blood lipid phenotype, yielding 443 unique blood lipid loci (for more details, see Results and Tables B-D in S1 File).

#### Number of pleiotropic loci

Table 1 shows the number of pleiotropic SNPs/gene loci identified with our conjunctional FDR method (conjFDR < 0.05) compared with the standard GWAS approach. When a SNP having conjFDR < 0.05 between one blood lipid trait and multiple immune-mediated diseases, we selected the immune-mediated disease showing the minimal conjFDR as the primary conjunctional phenotype. The number of pleiotropic SNPs in Table 1 indicates the overlapping loci between blood lipids and the primary immune-mediated disease. Across all evaluated lipid and immune-mediated phenotypes, our method increased the number of pleiotropic SNPs dramatically compared with the standard GWAS approach. TG showed the largest number of pleiotropic SNPs with immune-mediated diseases by both our method and the standard GWAS approach. On the other hand, LDL showed the fewest number of pleiotropic loci (Table 1 and Tables Q-S in S1 File). We also investigated the direction of these pleiotropic SNPs. Though there was no general patterns of directionality, SNPs having a large effect in CeD showed opposite direction of effect on all three blood lipid phenotypes; SNPs having larger effect in RA showed the same direction of effect in TG and HDL (Figures X-Z in S1 File).

**Table 1:** Comparison of number of ‘pleiotropic’ SNPs with standard GWAS approach.

Lipid	CD	UC	RA	T1D	CeD	PSOR	SARC
HDL	20(245;40)	16(107;17)	0(105;16)	0(75;18)	0(18;6)	0(2;1)	0(0)
LDL	1(134;29)	7(16;9)	13(417;66)	11(287;69)	7(57;24)	0(171;44)	0(0;0)
TG	0(205;23)	4(73;11)	37(678;95)	14(134;38)	24(25;13)	16(299;75)	0(0;0)

Comparison of the number of ‘pleiotropic’ SNPs/loci identified by standard GWAS and by conjFDR between lipid and immune-mediated diseases. Numbers in each cell indicate ‘pleiotropic’ SNPs from GWAS (p < 5 x10^-8^ for *both* lipid and immune phenotypes, correspondingly), from conjuctional FDR (conjFDR < 0.05; first number in parenthesis) and number of pruned loci (LD r^2^ > 0.2; second number in parenthesis). As illustrated, compared with a conventional GWAS approach, conjFDR identified a larger number of pleotropic loci across all evaluated lipid and immune-mediated phenotypes. Triglycerides (TG), low density lipoproteins (LDL) cholesterol, high density lipoproteins (HDL) cholesterol and Crohn’s Disease (CD), ulcerative colitis (UC), rheumatoid arthritis (RA), type 1 diabetes (T1D), celiac disease (CeD), psoriasis (PSOR) and sarcoidosis (SARC).

#### Confirmation analysis

The confirmation analysis using the larger recently published blood lipid GWAS[28] showed that reordering the SNPs according to conditional FDR results in an equal or better replication rate compared to the unconditional p-value ranking using the standard GWAS method, indicating that our FDR approach performs comparably to conventional methods (Figure Q in S1 File). The corresponding p-values from the larger GWAS are included in Tables B-D in S1 File.

### Shared biological pathways between blood lipids and immune-mediated diseases

**Fig 4:**
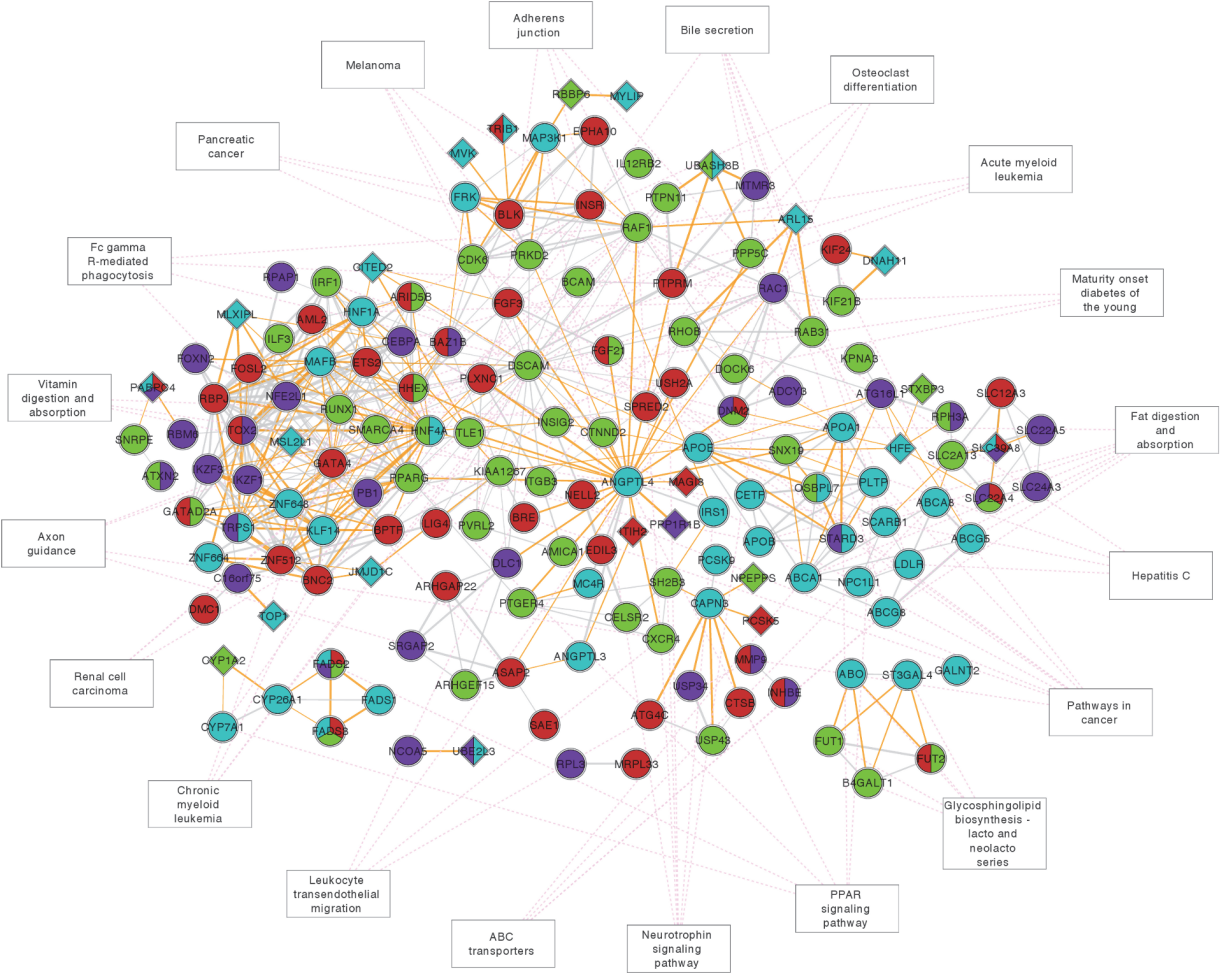
Functional gene network for novel pleiotropic loci from the present analysis and previously confirmed blood lipid loci. The protein-coding genes closest to the most associated SNP in the pleiotropic loci and the previously confirmed blood lipid loci were used to construct a functional similarity network of genes (see Methods). The network contains 158 gene nodes that are connected by 509 similarity edges. Genes previously reported to associate with blood lipids are represented by turquoise nodes (or turquoise node sectors). Red nodes (or red node sectors) represent pleiotropic loci between immune mediated diseases and Triglycerides (TG). Green nodes (or green node sectors) represent pleiotropic loci between immune-mediated diseases and low density lipoproteins (LDL) cholesterol. Purple nodes (or purple node sectors) represent pleiotropic loci between immune-mediated diseases and high density lipoproteins (HDL) cholesterol. Solid grey and orange edge lines indicate strong functional similarity between the connected genes based on their Gene Ontology annotations. Orange edge lines correspond to the 212 novel connections between nodes in the pleiotropic loci network and blood lipid loci network. Diamond node shapes represent the 23 new genes that arise after combining the pleiotropic loci and blood lipid loci into one network. Enriched KEGG pathways (see Tables O-P in S1 File) are connected to respective gene nodes with dotted pink lines. Genes and their nodes that are not connected to any other node in the network (48 genes) or annotated (82 genes) are omitted from the figure.

The biological relationships between pleiotropic loci identified with the new methodology were compared with that of loci previously shown to be associated with blood lipids using standard approaches[14], applying functional gene networks and protein interaction networks as well as biological pathway enrichment analysis. Notably, the connectivity among the loci in the combined network increased considerably compared to the networks represented by pleiotropic loci and by blood lipid loci only (Fig 4). This demonstrates biological relatedness between pleiotropic loci from the present analysis and blood lipid loci from previous reports[14]. In case of the functional gene network, a 40% increase in the number of network nodes representing blood lipid loci was observed compared to previous results (Fig 4). Furthermore, as revealed by the protein interaction network, the loci representing known hypercholesterolemic mouse models (e.g. *APOE* [MIM 107741] and *LDLR* [MIM 606945]) showed only a limited functional overlap with the pleiotropic loci (Figure O in S1 File). Enriched KEGG pathways represented by the pleiotropic loci include biological functions related to glycosphingolipid synthesis (e.g. *FUT2* [MIM 182100]) and to intestinal host-microbe interactions (e.g. *ATG16L1* [MIM 610767]) (Figure O in S1 File). Multiple enriched pathways for the pleiotropic loci also associate with cancer development (Figure O in S1 File).

## Discussion

Our findings indicate abundant polygenic overlap between blood lipid levels and immune-mediated diseases. Leveraging the association with immune-mediated diseases, we detected shared gene loci between these immune-mediated diseases and TG (n = 88 loci), LDL (n = 87 loci) and HDL (n = 52 loci). The variation in the genetic overlap for each observed immune-mediated disease group suggests differential underlying biological mechanisms for CD/UC, RA, T1D, CeD, SARC and PSOR. Pathway analysis showed little pleiotropy with core lipoprotein systems, but implicated multiple novel biological mechanisms of combined relevance concerning immune pathogenesis and lipid biology, which potentially also could include the gut microbiota as a possible common denominator for some of the shared mechanisms[39]. These findings may have implications for therapeutic strategies in immune and lipid-mediated diseases.

Our findings indicate polygenic overlap between blood lipids and immune-mediated diseases. Associations of some RA susceptibility genes with lipid levels have previously been reported[40], and overlap of individual gene loci between blood lipids and immune-mediated diseases has been previously recognized[8]. In addition, prior data have identified genes associated with lipid traits and inflammatory phenotypes. GWAS of the inflammatory biomarker C-reactive protein (CRP), identified several genes involved in metabolic syndrome, weight homeostasis, and premature atherothrombosis[41]. These pathways were confirmed in a recent meta-analysis of CRP GWAS, highlighting pleiotropy between immune response and metabolic regulatory pathways[42]. However, the extent of this pleiotropy may have been previously underestimated, since the polygenic pleiotropy between immune and lipid phenotypes was not identified. Further, several clinical and epidemiological studies have reported an association between blood lipids and individual immune-mediated diseases[1–5]. For example, CD and UC have been associated with low levels of HDL and high levels of LDL cholesterol[2,43,44]. However, the bases of these associations are difficult to discern when they are observed within the same individual, since both conditions may arise from shared environmental influences, or one condition may cause the other, with ambiguity in directionality of causation. Results from the current genetic epidemiology-based approach, in which shared genetic overlap was identified across separate study populations, strongly suggest that at least some of the phenotypic association arises from a shared genetic basis. Based on the selection criteria, it is unlikely that patients with unrecognized immune-related disorders were included in the blood lipids study population in large enough numbers to affect the results. However, we cannot exclude the possibility that some participants in the blood lipids population could develop immune-mediated disorders over time. In fact, as we discuss below, the genetic results imply that this may be likely to occur in some individuals. Further, the presence of overlapping genes between blood lipids and immune-related diseases does not necessarily reflect ‘true’ genetic pleiotropy as this is defined as genes common to independent phenotypes, which are not co-morbid. Instead, the shared loci presented here may be associated with overlapping pathophysiological mechanisms and additional experimental work is needed to further elucidate the biological underpinnings of these findings.

Leveraging the polygenic overlap between immune-mediated diseases and blood lipids, our findings identify novel loci associated with blood lipids. Compared with a conventional GWAS approach (p < 5 x10^-8^ for both lipid and immune phenotypes), we found that *conjunctional* FDR identified a larger number of pleotropic loci across all evaluated lipid and immune phenotypes. The z-scores suggest that the direction of effect varies—some gene loci increase risk of both phenotypes whereas others show opposing effects in the two phenotypes. Applying the *conditional* FDR approach, we used the signal in the associated phenotype to boost the discovery of blood lipid risk factors. Building upon our prior work[15–21], compared with a conventional FDR approach, we identified 374 additional blood lipid-associated SNPs using our conditional FDR method indicating that genetic overlap can improve statistical power for gene discovery. One potential concern relates to whether these novel analysis methods lower the statistical threshold (sensitivity) for novel SNP identification without improving the likelihood of detecting true findings (specificity). The present findings of increased confirmation rate in the larger blood lipid GWAS[28] of our conditional FDR approach compared to the standard GWAS methods suggest a reduced likelihood of type I error. Importantly, these findings illustrate that rather than lowering the statistical threshold, the current approach of re-ranking SNPs using pleiotropy identifies those variants that are more likely to replicate in independent samples (true positives). We have previously shown that genetic loci in cancer, brain related and cardiovascular phenotypes identified with our methods replicate at the same or higher rate as standard methods[15–17,19,20], and that these methods result in improved sensitivity for a given specificity[19]. It is important to note that few, if any, of our conjunctional loci (as indicated in Figures X-Z and Tables Q-S in S1 File) reached a traditional level of statistical significance within a genome-wide context and additional replication in independent cohorts is needed. However, for polygenic phenotypes, including cardiovascular and immune-mediated diseases, a single phenotype approach using standard approaches, i.e. Bonferroni-corrected significance thresholds, may not sufficiently explain phenotypic variance[15] or sufficiently identify shared genetic risk factors.

The polygenic overlap between blood lipids and immune-mediated disorders suggested by the present findings may inform novel pathogenic aspects of atherosclerosis, a chronic inflammatory process of the arterial wall, initiated by subsequent subendothelial accumulation of blood lipids[45]. Experimental evidence supports a key role for inflammation in atherosclerotic plaque formation, and the increased events of atherosclerosis in patients with chronic inflammatory rheumatic disorders[46,47] further support a role for inflammation in atherogenesis. In this context, the current findings of a polygenic link between lipid and inflammatory biology are likely to be relevant, suggesting that the well recognized bidirectional interaction between inflammatory and lipid pathways in atherogenesis may in fact involve some commen genetic factors between these pathways. Additional pathogenic insight from the novel loci is emphasized by the network analysis because loci related to known hypercholesterolemic mouse models (i.e. *Apoe* ^*−/−*^ and *Ldlr* ^*−/−*^ mice) show limited functional overlap with the pleiotropic loci. Further, recent evidence suggest that lipids may play a specific role in the local disruption of the epithelial barrier, including intestinal mucosa, in inflammatory disorders[48]. The present findings further implicate abnormal lipid biology in the disease mechanisms of CD, UC and CeD. The strong polygenic pleiotropy between lipids and T1D also supports a role of lipid pathobiology, as shown in relation to insulin secretion[49], diabetes risk[50] and pathology[51]. The current findings of statistical associations must be followed by experimental work to determine the specific underlying biology. The present results will be helpful for guiding the experimental work in direction of genes with potential involvement in disease mechanism.

Although we found an overlap between genetic risk factors for immune-mediated diseases and blood lipids, the specific loci involved for each trait differed. Recent findings suggest a common genetic basis for a number of immune-mediated diseases[52]. However, the pattern of genetic overlap for individual traits differs but has been poorly delineated. In the present analysis, there was a strong polygenic pleiotropic enrichment between TG and LDL and most of the examined immune-mediated disorders, whereas genetic overlap with HDL was primarily found for CD, UC and RA. Furthermore, we found a differential effect of the MHC region on immune-mediated enrichment on HDL compared with TG and LDL: removal of the MHC region resulted in significant enrichment attenuation in TG and LDL but not HDL. This suggests that the overlap between HDL and immune-mediated diseases is more polygenic and driven by non-MHC genetic regions whereas the MHC locus likely accounts for a large portion of the pleiotropy between LDL/TG and immune-mediated diseases. For SARC, little polygenic pleiotropic enrichment was found for either of the three blood lipid parameters. Whether this reflects a lower risk for development of cardiovascular disease in SARC relative to the other immune-mediated disorders is a question that merits further study. The current analytical approach depends on the power of the different GWAS samples. Thus, we must be careful when comparing results that are obtained from GWAS with different sample sizes. This is of particular concern for SARC, which has a much smaller sample size than several of the other GWAS.

Our findings suggest that there are underlying genetic risk factors that interplay with environment across all of these diseases. A striking risk locus in this regard is rs968567 linked to fatty acid desaturase 2 (*FADS2* [MIM 606149]). In the network analysis, *FADS2* connects with *FADS1*([MIM 606148]) and *FADS3*([MIM 606150]) as well as *CYP7A1*([MIM 118455]), the rate limiting enzyme in bile acid synthesis. By the introduction of double bands, some of these FADS play a key role in the formation of omega-6 and omega-3 polyunsaturated FA (PUFAs), such as eicosopentaenoic, docosahexaenoic acids and arachidonic acid derived from the dietary intake of essential FA[53,54]. The *FADS2* polymorphism (rs968567) is located in the promoter region of the gene and may affect the binding of a transcription factor, ELK1([MIM 311040])[53]. Variant status may impact *FADS2* expression and consequently the levels of PUFAs within cells and the inflammatory signalling through eicosanoids, and these pathway have been suggested as resolving mediators in chronic inflammatory disorders like atherosclerosis and the investigated immune-mediated disorders[55]. Further evidence for gene-environment interactions potentially underlying parts of the observed pleiotropy stems from the strong associations at *6p21* (MHC) as well as from the network analysis. One network cluster associates with the *FUT2* gene, which is a susceptibility gene for CD that contributes to the composition of the gut microbial community even in individuals without inflammatory bowel disease[39]. The gut microbial metabolism likely needs to be considered an integral part of the host metabolism of bile acids and short-chain fatty acids[56]. Bile acids are metabolized by intestinal bacteria and some of these metabolites are ligands for the farnesoid X receptor which regulates cholesterol, triglyceride and bile acid synthesis[39]. Consequently, alterations in the gut microbiota are likely to influence the regulation of bile acids and lipids metabolism[56], and bacterial derived bile acids predict response of statin treatment in humans[57]. Notably, changes in the gut microbiota are linked to the inflammatory status of patients with atherosclerosis[58], and some antibiotics possess lipid-lowering effects[59]. Recently, phosphatidylcholine metabolism, which is related to glycosphingolipid synthesis (*FUT2*), was implicated in atherosclerosis risk[60]. Altogether, several lines of evidence suggest that future experimental and genetic studies should incorporate assessments related to gene-microbial interactions and dietary intake of lipids[60,61].

This work has clinical implications. Our data further support the notion that among individuals with cardiovascular risk factors, immune and inflammatory status should be monitored and patients with immune-mediated disorders should be screened for abnormal blood lipid levels[22]. These findings also suggest the need for further investigating the role of lipid lowering drugs such as statins in patients with immune-mediated and inflammatory disorders. Such treatments could not only prevent the development of cardiovascular disorders, but could potentially influence autoimmunity and inflammation in these patients. It is tempting to hypothesize that those apparently healthy individuals with high load of these shared risk genotypes, predisposing for inflammation and lipid disturbances, could be at particularly high risk for developing cardiovascular disorders. Further prospective studies in these individuals could clarify these issues. There is some evidence indicating that anti-rheumatic drugs affect lipid profiles[62], and that cholesterol lowering drugs (statins) reduce risk of [63], suggesting that novel treatment regimens may evolve from the exploration of pathogenic mechanisms influenced by pleiotropic disease loci.

To conclude, we show abundant genetic overlap between blood lipids and a selection of archetypical immune-mediated disorders and identify a range of shared loci across the genome. These findings strongly support interactions between the immune system and lipid biology in human disease which may have implications for therapeutic trials involving lipid-lowering and anti-inflammatory agents.

## Supporting Information

S1 File (DOC)
