# Correction: Abundant Genetic Overlap between Blood Lipids and Immune-Mediated Diseases Indicates Shared Molecular Genetic Mechanisms

**DOI:** 10.1371/journal.pone.0128048

**Published:** 2015-05-15

**Authors:** Ole A. Andreassen, Rahul S. Desikan, Yunpeng Wang, Wesley K. Thompson, Andrew J. Schork, Verena Zuber, Nadezhda T. Doncheva, Eva Ellinghaus, Mario Albrecht, Morten Mattingsdal, Andre Franke, Benedicte A. Lie, Ian G. Mills, Pål Aukrust, Linda K. McEvoy, Srdjan Djurovic, Tom H. Karlsen, Anders M. Dale

The middle initial for the thirteenth author is missing. The correct name is: Ian G. Mills.

Affiliation 7 for authors Ian G. Mills and Verena Zuber is incorrect. The correct affiliation is: Centre for Molecular Medicine Norway, Nordic EMBL Partnership, University of Oslo and Oslo University Hospital, 0349 Oslo, Norway.
