# Supplementary material for: Abundant Genetic Overlap between Blood Lipids and Immune-Mediated Diseases Indicates Shared Molecular Genetic Mechanisms
Source: PLoS One. 2015 Apr 8;10(4):e0123057. doi: 10.1371/journal.pone.0123057 (PMC4390360; doi:10.1371/journal.pone.0123057)
Supplement: S1 File — (DOC) [file pone.0123057.s001.doc]

**SUPPORTING INFORMATION**

**Abundant genetic overlap between blood lipids and immune-mediated diseases indicates shared molecular genetic mechanisms**

Table of Contents

Figure A. QQ plots of triglycerides conditioned on immune-mediated diseases.

Figure B. QQ plots of low density lipoproteins conditioned on immune-mediated diseases.

Figure C. QQ plots of high density lipoproteins conditioned on immune-mediated diseases.

Figure D. Enrichment plots of triglycerides conditioned on immune-mediated diseases.

**Figure E. Enrichment plots of low-density lipoproteins conditioned on immune-mediated diseases.**

**Figure F. Enrichment plots of high density lipoproteins conditioned on immune-mediated diseases.**

Figure G. QQ plots of triglycerides conditioned on immune-mediated diseases without MHC-related SNPs.

Figure H. QQ plots of low density lipoprotein conditioned on immune-mediated diseases without MHC-related SNPs.

Figure I. QQ plots of high density lipoprotein conditioned on immune-mediated diseases without MHC-related SNPs.

Figure J. Enrichment plots of triglycerides conditioned on immune-mediated diseases without MHC-related SNPs.

Figure K. Enrichment plots low of density lipoprotein conditioned on immune-mediated diseases without MHC-related SNPs.

Figure L. Enrichment plots high of density lipoprotein conditioned on immune-mediated diseases without MHC-related SNPs.

Figure M. ‘Heat maps’ of the number of conjunctive SNPs (overlapping SNPs) at conjunctional FDR values < 0.05.

**Figure N. ‘Heat maps’ of the number of conjunctive SNPs (overlapping) at conjunctional FDR values < 0.05 wihout MHC-related SNPs.**

Figure O. Protein interaction network for novel pleiotropic loci from the present analysis and previously confirmed blood lipid loci.

**Figure P. Enrichment plots of lipids conditioned on cigarettes per day.**

**Figure Q. Relative confirmation rate.**

**Figure R. QQ plots of high density lipoprotein conditioned on immune-mediated diseases after random pruning.**

**Figure S. QQ plots of low density lipoprotein conditioned on immune-mediated diseases after random pruning.**

**Figure T. QQ plots of triglycerides conditioned on immune-mediated diseases after random pruning.**

**Figure U. Conjunctional FDR Manhattan plot for triglycerides (TG).**

### Figure V. Conjunctional FDR Manhattan plot for low density lipoprotein (LDL).

### Figure W. Conjunctional FDR Manhattan plot for high density lipoprotein (HDL).

### Figure X. Effect direction of 'pleiotropic' SNP between high density lipoprotein (HDL) and

### immune-mediated diseases.

### Figure Y. Effect direction of 'pleiotropic' SNP between low density lipoprotein (LDL) and

### immune-mediated diseases.

### Figure Z. Effect direction of 'pleiotropic' SNP between triglycerides (TG) and

**immune-mediated diseases.**

**Table A. Summary data from all GWAS used in the current study.**

Table B. SNPs in triglycerides (TG) conditoned on immune-mediated diseases (condFDR<0.01).

Table C. SNPs in low density lipoprotein (LDL) conditoned on immune-mediated diseases (condFDR<0.01).

Table D. SNPs in high density lipoprotein (HDL) conditoned on immune-mediated diseases (condFDR<0.01).

Table E. Non-MHC pleiotropic SNPs in triglycerides (TG) and immune-mediated diseases (conj FDR<0.01).

Table F. Non-MHC pleiotropic SNPs in low density lipoprotein (LDL) and immune-mediated diseases (conj FDR<0.01).

Table G. Non-MHC pleiotropic SNPs in high density lipoprotein (HDL) and immune-mediated diseases.

Table H. Pleiotropic SNPs in triglycerides (TG) and immune mediated-diseases (conjFDR<0.05).

Table I. Pleiotropic SNPs in low density lipoprotein (LDL) and immune-mediated diseases (conjFDR<0.05).

Table J. Pleiotropic SNPs in high density lipoprotein (HDL) and immune-mediated diseases (conjFDR<0.05).

Table K. Independent complex or single gene loci (r2 < 0.2) with z-scores for each significant pleiotropic loci from Table S5. in immune-mediated diseases and triglycerides (TG).

Table L. Independent complex or single gene loci (r^2^ < 0.2) with z-scores for each significant pleiotropic loci from Table S6. in immune-mediated diseases and low density lipoprotein (LDL).

Table M. Independent complex or single gene loci (r2 < 0.2) with z-scores for each significant pleiotropic loci from Table S7 in immune-mediated diseases and high density lipoprotein (HDL).

Table N. Correlation coefficients of z-scores.

Table O. Enriched KEGG pathways in the functional gene network.

Table P. Enriched KEGG pathways in the protein interaction network.

Table Q. Pleiotropy SNPs between high density lipoprotein (HDL) and immune identified by GWAS.

Table R. Pleiotropy SNPs between low density lipoprotein (LDL) and immune identified by GWAS.

Table S. Pleiotropy SNPs between triglycerides (TG) and immune identified by GWAS.

**Figure A. QQ plots of triglycerides conditioned on immune-mediated diseases.**

**Figure A.** **A-G. Pleiotropic enrichment of triglycerides and immune-mediated diseases.** Conditional Q-Q plot of nominal versus empirical -log_10_ p-values (corrected for inflation) in triglycerides (TG) below the standard GWAS threshold of p < 5x10^-8^ as a function of significance of association with **A**) Crohn’s Disease (CD), **B**) ulcerative colitis (UC), **C**) rheumatoid arthritis (RA), **D**) type 1 diabetes (T1D), **E**) celiac disease (CeD), **F**) psoriasis (PSOR) and **G**) sarcoidosis (SARC) at the level of -log_10_(p) > 0, –log_10_(p) > 1, –log_10_(p) > 2, –log_10_(p) > 3 corresponding to p < 1, p < 0.1, p < 0.01, p < 0.001, respectively. Dotted lines indicate the null-hypothesis.

### Figure B. QQ plots of low-density lipoproteins conditioned on immune-mediated diseases.

**Figure B.** **A-G. Pleiotropic enrichment of low density lipoproteins (LDL) and immune-mediated diseases.** Conditional Q-Q plot of nominal versus empirical -log_10_ p-values (corrected for inflation) in low density lipoprotein (LDL) cholesterol below the standard GWAS threshold of p < 5x10^-8^ as a function of significance of association with **A**) Crohn’s Disease (CD), **B**) ulcerative colitis (UC), **C**) rheumatoid arthritis (RA), **D**) type 1 diabetes (T1D), **E**) celiac disease (CeD), **F**) psoriasis (PSOR) and **G**) sarcoidosis (SARC) at the level of -log_10_(p) > 0, –log_10_(p) > 1, –log_10_(p) > 2, –log_10_(p) > 3 corresponding to p < 1, p < 0.1, p < 0.01, p < 0.001, respectively. Dotted lines indicate the null-hypothesis.

### Figure C. QQ plots of high density lipoproteins conditioned on immune-mediated diseases.

**Figure C.** **A-G. Pleiotropic enrichment of high density lipoproteins (HDL) and immune-mediated diseases.** Conditional Q-Q plot of nominal versus empirical -log_10_ p-values (corrected for inflation) in high density lipoprotein (HDL) cholesterol below the standard GWAS threshold of p < 5x10^-8^ as a function of significance of association with **A**) Crohn’s Disease (CD), **B**) ulcerative colitis (UC), **C**) rheumatoid arthritis (RA), **D**) type 1 diabetes (T1D), **E**) celiac disease (CeD), **F**) psoriasis (PSOR) and **G**) sarcoidosis (SARC) at the level of -log_10_(p) > 0, –log_10_(p) > 1, –log_10_(p) > 2, –log_10_(p) > 3 corresponding to p < 1, p < 0.1, p < 0.01, p < 0.001, respectively. Dotted lines indicate the null-hypothesis.

### Figure D. Enrichment plots of triglycerides conditioned on immune-mediated diseases.

**Figure D.** **A-G.** Pleiotropic Enrichment. Enrichment plot of x-fold enrichment vs. empirical -log_10_ p-values (corrected for inflation) in Triglycerides (TG) below the standard GWAS threshold of p < 5x10^-8^ as a function of significance of association with **A**) Crohn’s Disease (CD), **B**) ulcerative colitis (UC), **C**) rheumatoid arthritis (RA), **D**) type 1 diabetes (T1D), **E**) celiac disease (CeD), **F**) psoriasis (PSOR) and **G**) sarcoidosis (SARC) at the level of -log_10_(p) > 0, –log_10_(p) > 1, –log_10_(p) > 2, –log_10_(p) > 3 corresponding to p < 1, p < 0.1, p < 0.01, p < 0.001, respectively.

Figure E. Enrichment plots of Low Density Lipoprotein **conditioned on** immune-mediated diseases.

**Figure E.** A-G. Pleiotropic Enrichment. Enrichment plot of x-fold enrichment vs. empirical -log_10_ p-values (corrected for inflation) in Low Density Lipoprotein (LDL) below the standard GWAS threshold of p < 5x10^-8^ as a function of significance of association with **A**) Crohn’s Disease (CD), **B**) ulcerative colitis (UC), **C**) rheumatoid arthritis (RA), **D**) type 1 diabetes (T1D), **E**) celiac disease (CeD), **F**) psoriasis (PSOR) and **G**) sarcoidosis (SARC) at the level of -log_10_(p) > 0, –log_10_(p) > 1, –log_10_(p) > 2, –log_10_(p) > 3 corresponding to p < 1, p < 0.1, p < 0.01, p < 0.001, respectively.

Figure F. Enrichment plots of High Density Lipoprotein **conditioned on** immune-mediated diseases.

**Figure F. A-G.** Pleiotropic Enrichment. Enrichment plot of x-fold enrichment vs. empirical -log_10_ p-values (corrected for inflation) in High Density Lipoprotein (HDL) below the standard GWAS threshold of p < 5x10^-8^ as a function of significance of association with **A**) Crohn’s Disease (CD), **B**) ulcerative colitis (UC), **C**) rheumatoid arthritis (RA), **D**) type 1 diabetes (T1D), **E**) celiac disease (CeD), **F**) psoriasis (PSOR) and **G**) sarcoidosis (SARC) at the level of -log_10_(p) > 0, –log_10_(p) > 1, –log_10_(p) > 2, –log_10_(p) > 3 corresponding to p < 1, p < 0.1, p < 0.01, p < 0.001, respectively.

### Figure G. Enrichment of triglycerides conditioned on immune-mediated diseases without MHC-related SNPs.

**Figure G.** **A-G. Pleiotropic enrichment of triglycerides (TG) and immune-mediated diseases without MHC-related SNPs.** Conditional Q-Q plot of nominal versus empirical -log_10_ p-values (corrected for inflation) in triglycerides (TG) cholesterol below the standard GWAS threshold of p < 5x10^-8^ as a function of significance of association with **A**) Crohn’s Disease (CD), **B**) ulcerative colitis (UC), **C**) rheumatoid arthritis (RA), **D**) type 1 diabetes (T1D), **E**) celiac disease (CeD), **F**) psoriasis (PSOR) and **G**) sarcoidosis (SARC) at the level of -log_10_(p) > 0, –log_10_(p) > 1, –log_10_(p) > 2, –log_10_(p) > 3 corresponding to p < 1, p < 0.1, p < 0.01, p < 0.001, respectively. MHC-related SNPs were removed before the analysis. Dotted lines indicate the null-hypothesis.

### Figure H. QQ plots of Low Density Lipoprotein conditioned on immune-mediated diseases without MHC-related SNPs.

**Figure H.** **A-G. Pleiotropic enrichment of low density lipoproteins (LDL) and immune-mediated diseases without MHC-related SNPs.** Conditional Q-Q plot of nominal versus empirical -log_10_ p-values (corrected for inflation) in low density lipoprotein (LDL) cholesterol below the standard GWAS threshold of p < 5x10^-8^ as a function of significance of association with **A**) Crohn’s Disease (CD), **B**) ulcerative colitis (UC), **C**) rheumatoid arthritis (RA), **D**) type 1 diabetes (T1D), **E**) celiac disease (CeD), **F**) psoriasis (PSOR) and **G**) sarcoidosis (SARC) at the level of -log_10_(p) > 0, –log_10_(p) > 1, –log_10_(p) > 2, –log_10_(p) > 3 corresponding to p < 1, p < 0.1, p < 0.01, p < 0.001, respectively. MHC-related SNPs were removed before the analysis. Dotted lines indicate the null-hypothesis.

### Figure I. QQ plots of high density lipoprotein conditioned on immune-mediated diseases without MHC-related SNPs.

**Figure I.** **A-G. Pleiotropic enrichment of high density lipoproteins (HDL) and immune-mediated diseases without MHC-related SNPs.** Conditional Q-Q plot of nominal versus empirical -log_10_ p-values (corrected for inflation) in high density lipoprotein (HDL) cholesterol below the standard GWAS threshold of p < 5x10^-8^ as a function of significance of association with **A**) Crohn’s Disease (CD), **B**) ulcerative colitis (UC), **C**) rheumatoid arthritis (RA), **D**) type 1 diabetes (T1D), **E**) celiac disease (CeD), **F**) psoriasis (PSOR) and **G**) sarcoidosis (SARC) at the level of -log_10_(p) > 0, –log_10_(p) > 1, –log_10_(p) > 2, –log_10_(p) > 3 corresponding to p < 1, p < 0.1, p < 0.01, p < 0.001, respectively. MHC-related SNPs were removed before the analysis. Dotted lines indicate the null-hypothesis.

### Figure J. Enrichment plots of triglycerides conditioned on immune-mediated diseases without MHC-related SNPs.

**Figure J. A-G.** Pleiotropic Enrichment. Enrichment plot of x-fold enrichment vs. empirical -log_10_ p-values (corrected for inflation) in Triglycerides (TG) below the standard GWAS threshold of p < 5x10^-8^ as a function of significance of association with **A**) Crohn’s Disease (CD), **B**) ulcerative colitis (UC), **C**) rheumatoid arthritis (RA), **D**) type 1 diabetes (T1D), **E**) celiac disease (CeD), **F**) psoriasis (PSOR) and **G**) sarcoidosis (SARC) at the level of -log_10_(p) > 0, –log_10_(p) > 1, –log_10_(p) > 2, –log_10_(p) > 3 corresponding to p < 1, p < 0.1, p < 0.01, p < 0.001, respectively. MHC-related SNPs were removed before the analysis.

### Figure K. Enrichment plots of low density lipoprotein conditioned on immune-mediated diseases without MHC-related SNPs.

**Figure K. A-G.** Pleiotropic Enrichment. Enrichment plot of x-fold enrichment vs. empirical -log_10_ p-values (corrected for inflation) in Low Density Lipoprotein (LDL) below the standard GWAS threshold of p < 5x10^-8^ as a function of significance of association with **A**) Crohn’s Disease (CD), **B**) ulcerative colitis (UC), **C**) rheumatoid arthritis (RA), **D**) type 1 diabetes (T1D), **E**) celiac disease (CeD), **F**) psoriasis (PSOR) and **G**) sarcoidosis (SARC) at the level of -log_10_(p) > 0, –log_10_(p) > 1, –log_10_(p) > 2, –log_10_(p) > 3 corresponding to p < 1, p < 0.1, p < 0.01, p < 0.001, respectively. MHC-related SNPs were removed before the analysis.

### Figure L. Enrichment plots of high density lipoprotein conditioned on immune-mediated diseases without MHC-related SNPs.

**Figure L. A-G.** Pleiotropic Enrichment. Enrichment plot of x-fold enrichment vs. empirical -log_10_ p-values (corrected for inflation) in High Density Lipoprotein (HDL) below the standard GWAS threshold of p < 5x10^-8^ as a function of significance of association with **A**) Crohn’s Disease (CD), **B**) ulcerative colitis (UC), **C**) rheumatoid arthritis (RA), **D**) type 1 diabetes (T1D), **E**) celiac disease (CeD), **F**) psoriasis (PSOR) and **G**) sarcoidosis (SARC) at the level of -log_10_(p) > 0, –log_10_(p) > 1, –log_10_(p) > 2, –log_10_(p) > 3 corresponding to p < 1, p < 0.1, p < 0.01, p < 0.001, respectively. MHC-related SNPs were removed before the analysis.

### Figure M. ‘Heat maps’ of the number of conjunctive SNPs (overlapping SNPs) at conjunctional FDR values < 0.05.


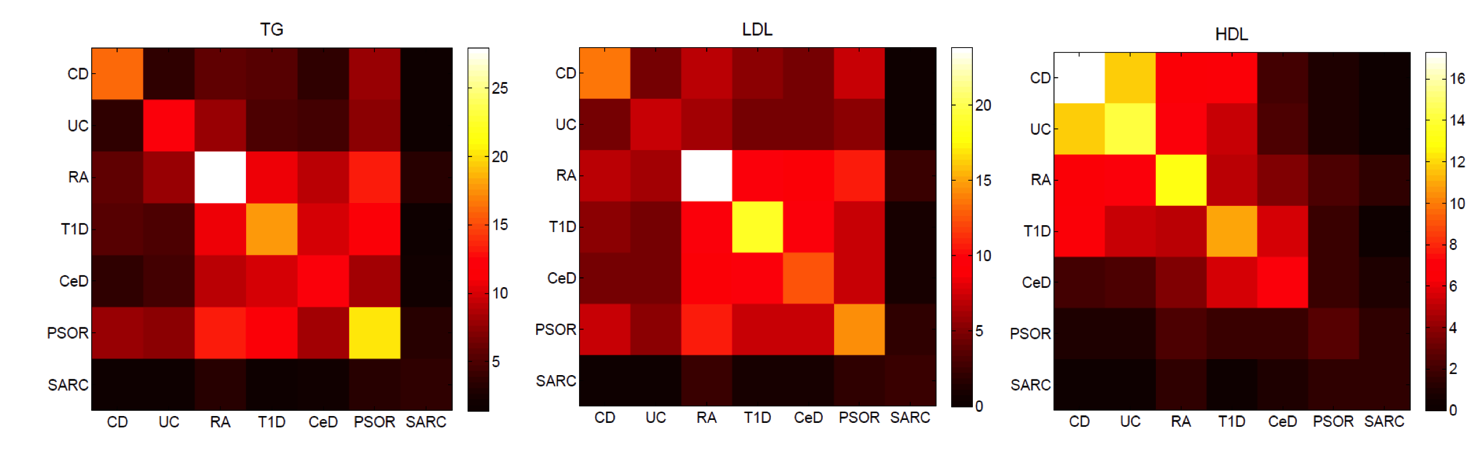


### Figure M. A-C. The number of SNPs with conjunctional FDR values (< 0.05) for A) TG, B) LDL and C) HDL given the associated phenotypes CD, UC, RA, T1D, CeD, PSOR and SARC are coded by color scale. The colors shown on the diagonal reflect the number of pleiotropic SNPs for a given lipid parameter and a specific disease. For example, in A, there are more than 25 SNPs that show pleiotropy between TG and RA. The off-diagonal elements indicate the number of SNPs showing three-way pleiotropy across the given lipid phenotype and a pair of different immune-mediated diseases. For example, several SNPs that show pleiotropic TG-RA signal also show pleiotropic TG-T1D signal. In contrast, very few show common TG-RA and TG-SARC associations. These maps illustrate a similar pattern of overlap of TG and LDL with several immune-mediated diseases (mainly RA, T1D and CeD) that is distinct from the pattern of overlap displayed for HDL, which involves a different set of immune-mediated diseases (i.e. CD and UC).

### Figure N. ‘Heat maps’ of the number of conjunctive SNPs (overlapping SNPs) at conjunctional FDR values < 0.05 without MHC-related SNPs.


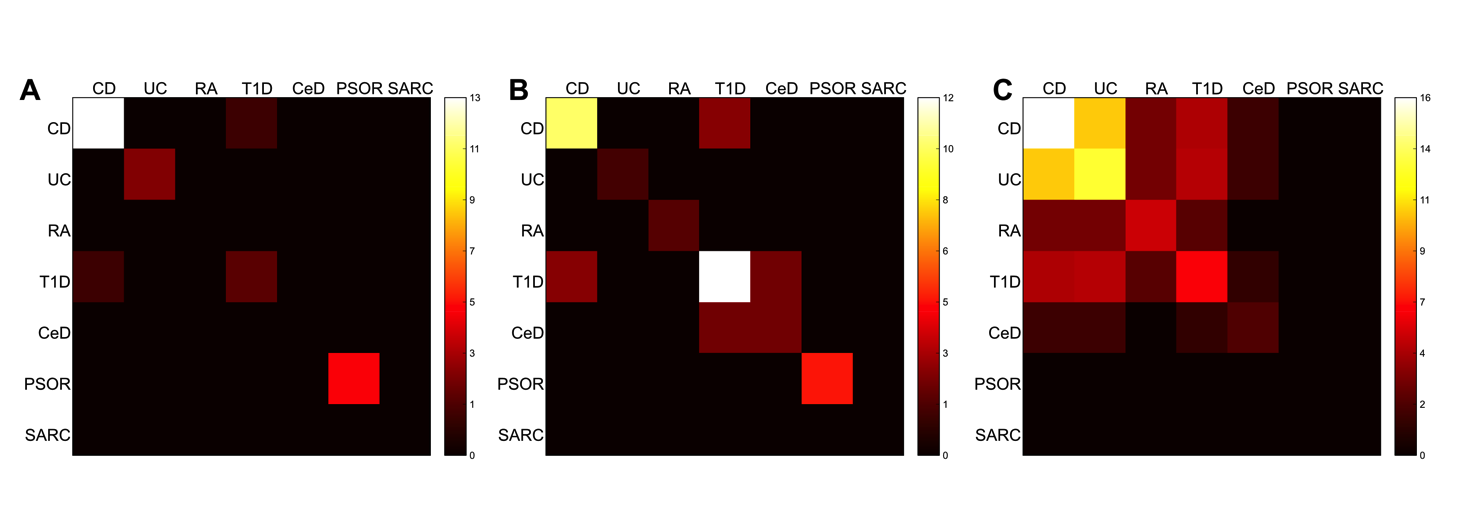


### Figure N. A-C. The number of SNPs with conjunctional FDR values (< 0.05) for A) TG, B) LDL and C) HDL given the associated phenotypes CD, UC, RA, T1D, CeD, PSOR and SARC are coded by color scale. The colors shown on the diagonal reflect the number of pleiotropic SNPs for a given lipid parameter and a specific disease. See caption to Figure 2 in the main text for further details on interpreting the heat maps. Removal of MHC-related SNPs reduced much of the common overlap between immune-mediated diseases and TG and LDL phenotypes, but had much less impact on the pattern of overlap between HLD and immune-mediated diseases.

Figure O. Protein interaction network.

### Figure O. Protein interaction network for novel pleiotropic loci from the present analysis and previously confirmed blood lipid loci^1^. Protein interaction data was retrieved for the proteins encoded by the genes closest to the most associated SNP in the pleiotropic loci and the previously confirmed blood lipid loci (see Methods). The network contains 79 protein nodes that are connected by 77 interaction edges. Proteins encoded by genes previously reported to associate with blood lipids are represented by turquoise nodes (or turquoise node sectors). Red nodes (or red node sectors) represent pleiotropic loci between immune-mediated diseases and triglycerides (TG). Green nodes (or green node sectors) represent pleiotropic loci between immune-mediated diseases and low density lipoprotein (LDL) cholesterol. Purple nodes (or purple node sectors) represent pleiotropic loci between immune-mediated diseases and high density lipoprotein (HDL) cholesterol. Solid grey and orange edge lines correspond to protein-protein interactions. Orange edge lines indicate 20 novel connections between nodes in the pleiotropic loci network and blood lipid loci network. Diamond node shapes represent the 10 new proteins that arise after combining the pleiotropic loci and blood lipid loci into one network. Enriched KEGG pathways (see Table S13) are connected to respective protein nodes with dotted pink lines. Proteins and their nodes that are not connected to any other node in the network (161 proteins) or annotated (48 proteins) are omitted from the figure.

**Figure P. QQ plots of lipids conditioned on cigarettes per day**


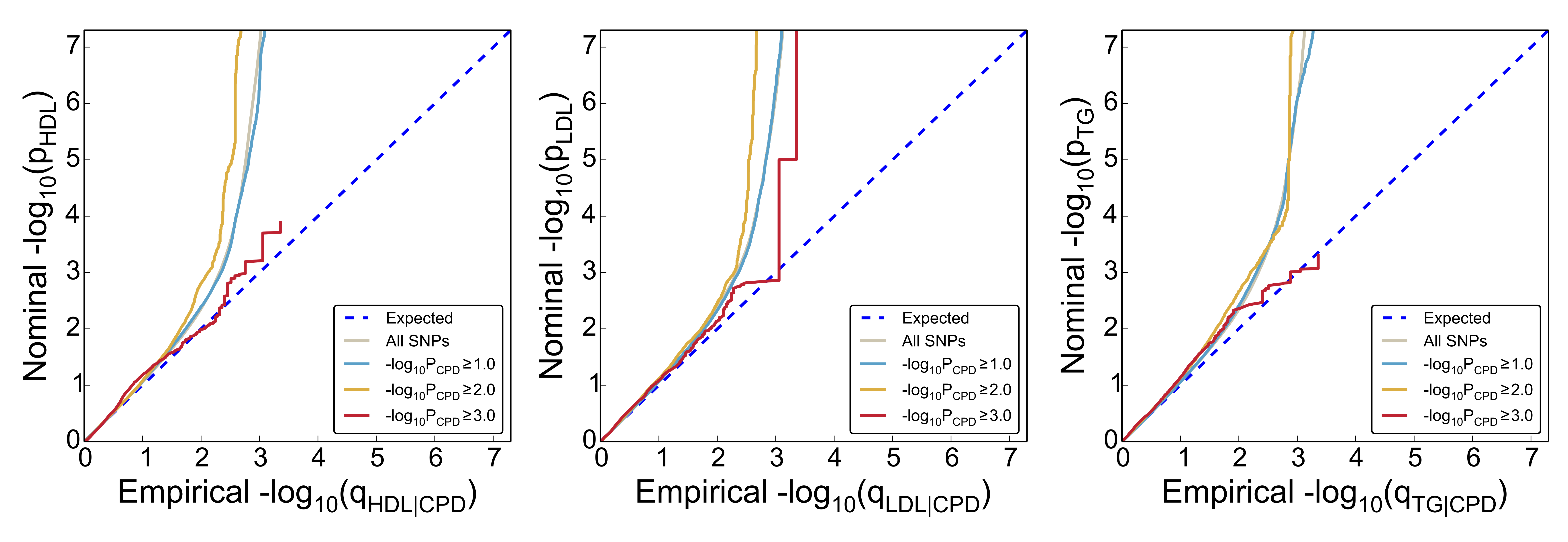


**Figure P.** **A-C. Pleiotropic enrichment of A) high density lipoprotein (HDL), B) low density lipoprotein (LDL) and C) triglycerides (TG) conditioned on cigarette per day (CPD).** Conditional Q-Q plot of nominal versus empirical -log_10_ p-values (corrected for inflation) in HDL, LDL and TG below the standard GWAS threshold of p < 5x10^-8^ as a function of significance of association CPD at the level of -log_10_(p) > 0, –log_10_(p) > 1, –log_10_(p) > 2, –log_10_(p) > 3 corresponding to p < 1, p < 0.1, p < 0.01, p < 0.001, respectively. Dotted lines indicate the null-hypothesis.

**Figure Q. Relative confirmation rate**


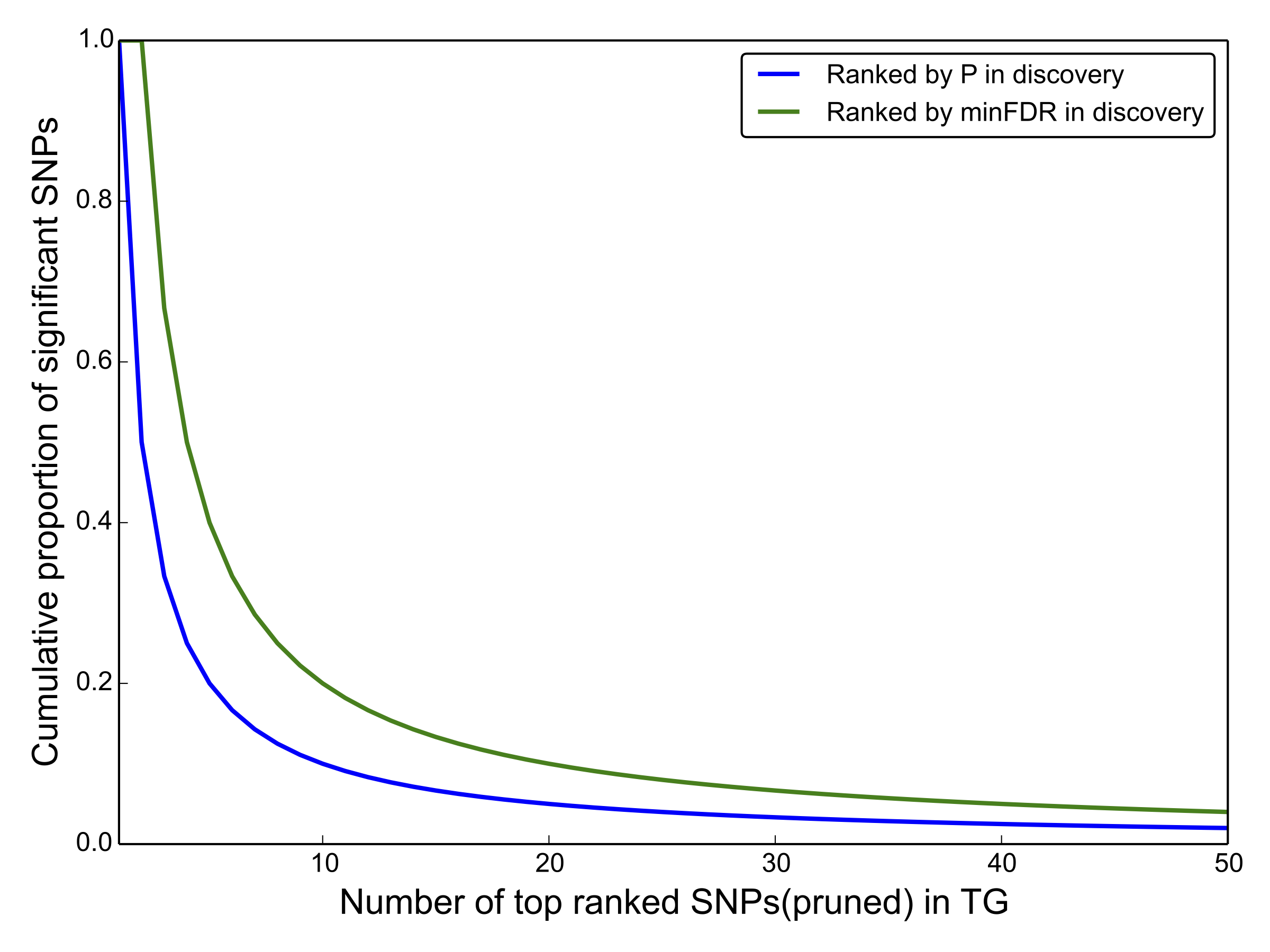


**Figure Q. Relative confirmation rate of triglycerides (TG) SNPs in the larger lipid GWAS sample**. Fraction of SNPs identified with our FDR approach (green) compared with standard GWAS analysis method (blue) that remained significant (p < 5 x 10^-8^) in the larger lipid GWAS(1). SNPs were ranked by P values (blue) and FDR values conditioned on other traits (cond

FDR; green). In order to focus on new gene discoveries, only SNPs with p > 5 x 10^-8^ in the primary GWAS analysis were included in the calculation. The proportion of SNPs with P < 5 x 10^-8^ in the larger confirmation GWAS was plotted against the number of top ranked SNPs (after LD pruning, R^2^ <0.2).

**Figure R. QQ plots of high density lipoprotein conditioned on immune-mediated diseases after random pruning.**

**Figure R.** **A-G. Pleiotropic enrichment of high density lipoprotein and immune-mediated diseases after 100 random pruning.** Conditional Q-Q plot of nominal versus empirical -log_10_ p-values (corrected for inflation) in high density lipoprotein(HDL) below the standard GWAS threshold of p < 5x10^-8^ as a function of significance of association with **A**) Crohn’s Disease (CD), **B**) ulcerative colitis (UC), **C**) rheumatoid arthritis (RA), **D**) type 1 diabetes (T1D), **E**) celiac disease (CeD), **F**) psoriasis (PSOR) and **G**) sarcoidosis (SARC) at the level of -log_10_(p) > 0, –log_10_(p) > 1, –log_10_(p) > 2, –log_10_(p) > 3 corresponding to p < 1, p < 0.1, p < 0.01, p < 0.001, respectively. The plotted data are based on the average of 100 random pruning. Dotted lines indicate the null-hypothesis.

**Figure S. QQ plots of low density lipoprotein conditioned on immune-mediated diseases after random pruning.**

**Figure S.** **A-G. Pleiotropic enrichment of low density lipoprotein and immune-mediated diseases after 100 random pruning.** Conditional Q-Q plot of nomiersus empirical -log_10_ p-values (corrected for inflation) in low density lipoprotein(LDL) below the standard GWAS threshold of p < 5x10^-8^ as a function of significance of association with **A**) Crohn’s Disease (CD), **B**) ulcerative colitis (UC), **C**) rheumatoid arthritis (RA), **D**) type 1 diabetes (T1D), **E**) celiac disease (CeD), **F**) psoriasis (PSOR) and **G**) sarcoidosis (SARC) at the level of -log_10_(p) > 0, –log_10_(p) > 1, –log_10_(p) > 2, –log_10_(p) > 3 corresponding to p < 1, p < 0.1, p < 0.01, p < 0.001, respectively. The plotted data are based on the average of 100 random pruning. Dotted lines indicate the null-hypothesis.

**Figure T. QQ plots of triglycerides conditioned on immune-mediated diseases after random pruning.**

**Figure T.** **A-G. Pleiotropic enrichment of triglycerides and immune-mediated diseases after 100 random pruning.** Conditional Q-Q plot of nomiersus empirical -log_10_ p-values (corrected for inflation) in triglycerides(TG) below the standard GWAS threshold of p < 5x10^-8^ as a function of significance of association with **A**) Crohn’s Disease (CD), **B**) ulcerative colitis (UC), **C**) rheumatoid arthritis (RA), **D**) type 1 diabetes (T1D), **E**) celiac disease (CeD), **F**) psoriasis (PSOR) and **G**) sarcoidosis (SARC) at the level of -log_10_(p) > 0, –log_10_(p) > 1, –log_10_(p) > 2, –log_10_(p) > 3 corresponding to p < 1, p < 0.1, p < 0.01, p < 0.001, respectively. The plotted data are based on the average of 100 random pruning. Dotted lines indicate the null-hypothesis.

### Figure U. Conjunctional FDR Manhattan plot for triglycerides (TG).


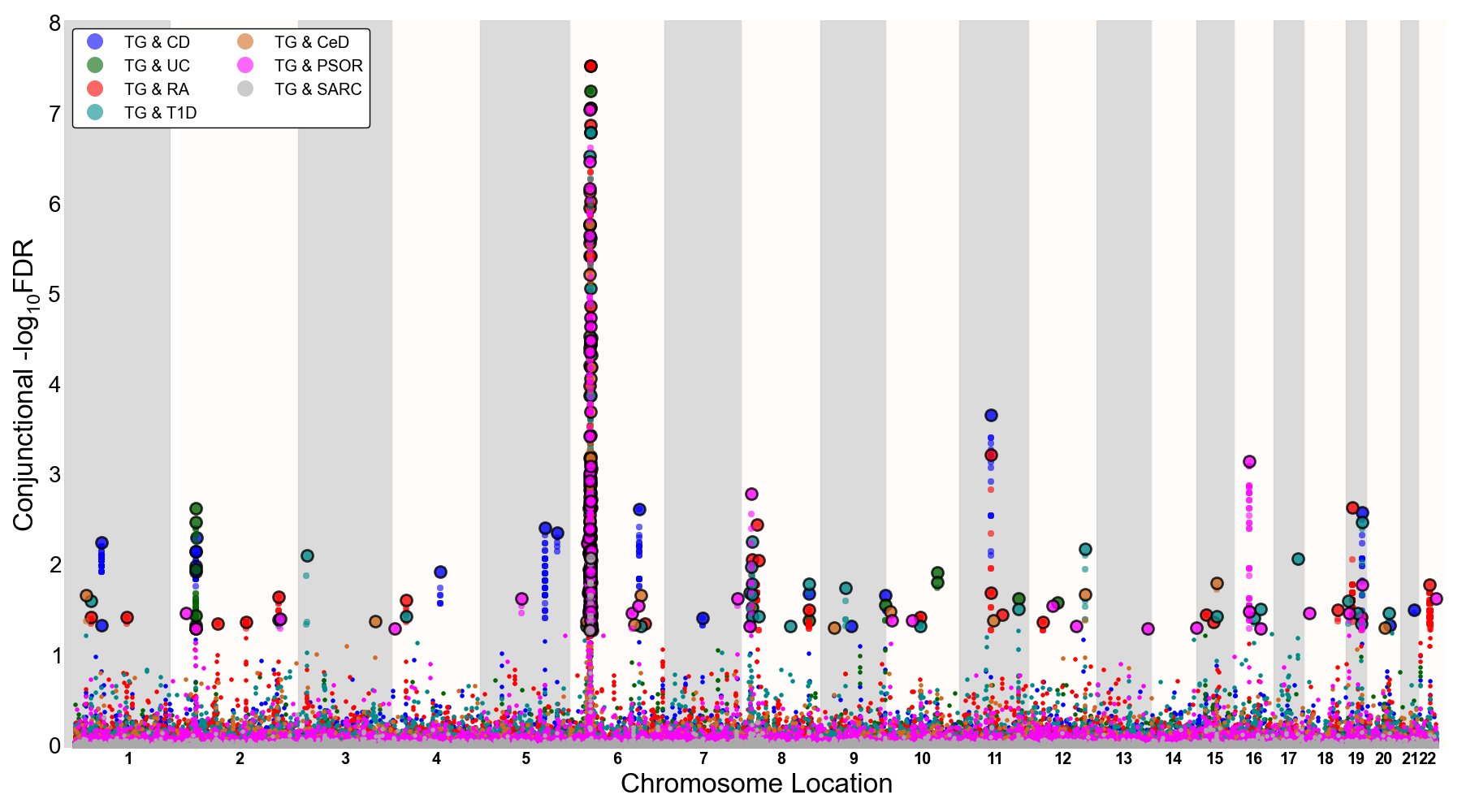


**Figure U. Conjunctional FDR Manhattan plot for triglycerides (TG) without showing gene symbols**. Conjunctional –log_10_(FDR) values for triglycerides (TG) and Crohn’s Disease (CD), ulcerative colitis (UC), rheumatoid arthritis (RA), type 1 diabetes (T1D), celiac disease (CeD), psoriasis (PSOR) and G) sarcoidosis (SARC) were plotted along their chromosome locations. SNPs with conjunctional FDR < 0.05 (i.e., –log_10_ FDR > 1.3) are shown with enlarged data points. A black circle around the enlarged data points indicates the most significant SNP in each LD block. Details for the associated loci outside of chromosome 6 are shown in Table S5.

### Figure V. Conjunctional FDR Manhattan plot for low density lipoprotein (LDL).


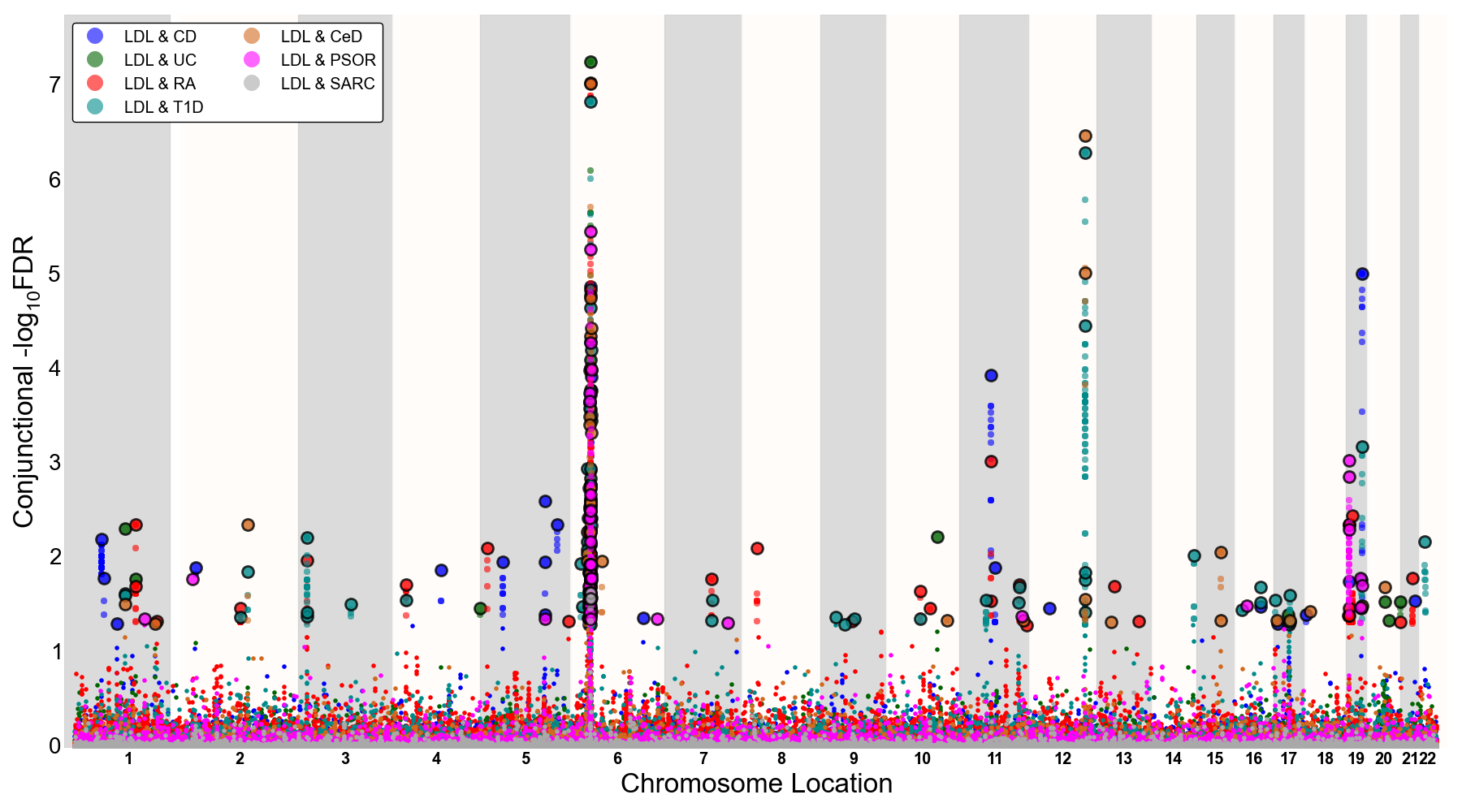


**Figure V. Conjunctional FDR Manhattan plot for low density lipoprotein (LDL) without showing gene symbols**. Conjunctional –log_10_(FDR) values for low density lipoprotein (LDL) and Crohn’s Disease (CD), ulcerative colitis (UC), rheumatoid arthritis (RA), type 1 diabetes (T1D), celiac disease (CeD), psoriasis (PSOR) and G) sarcoidosis (SARC) were plotted along their chromosome locations. SNPs with conjunctional FDR < 0.05 (i.e., –log_10_ FDR > 1.3) are shown with enlarged data points. A black circle around the enlarged data points indicates the most significant SNP in each LD block. Details for the associated loci outside of chromosome 6 are shown in Table S6.

### Figure W. Conjunctional FDR Manhattan plot for high density lipoprotein (HDL).


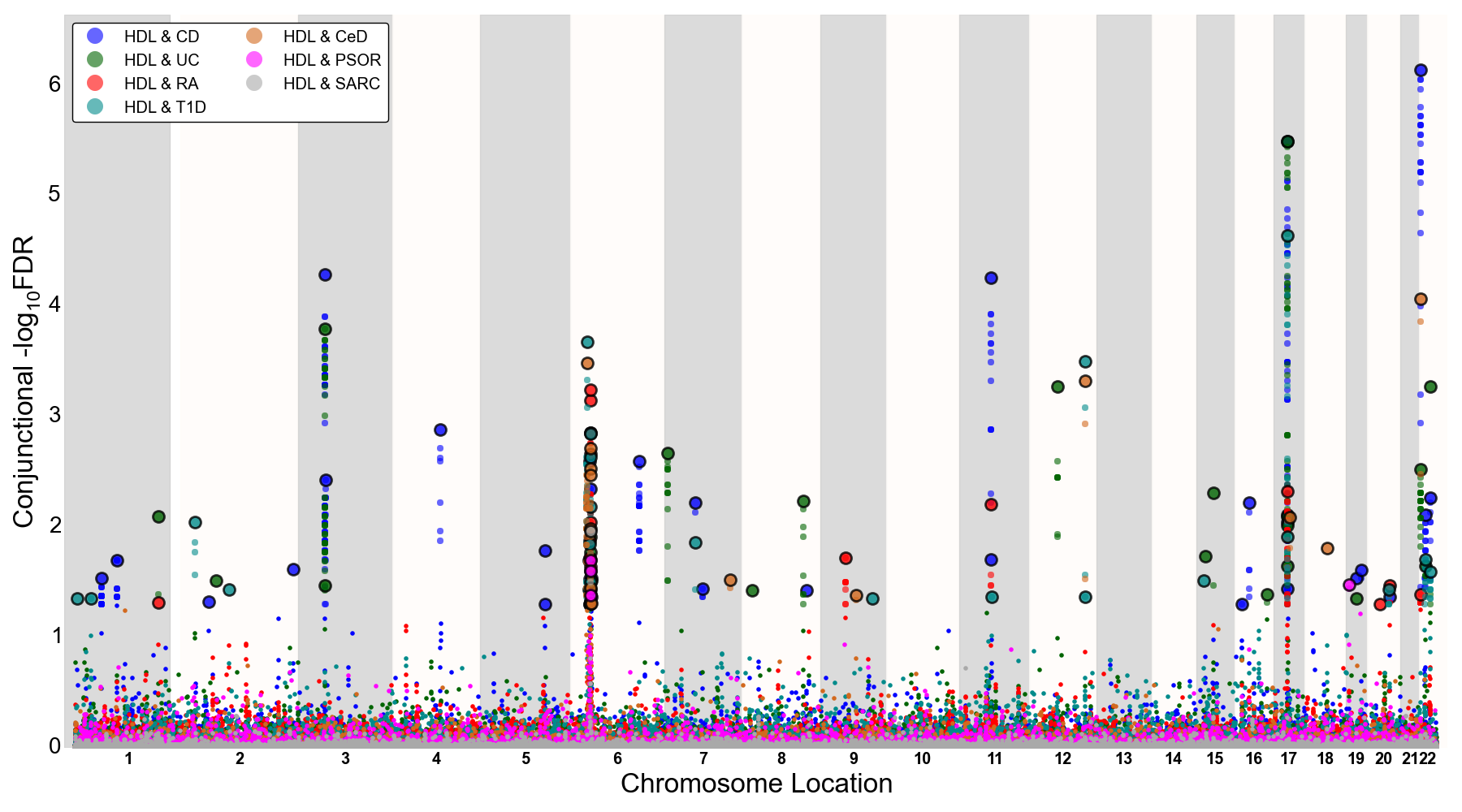


**Figure W. Conjunctional FDR Manhattan plot for high density lipoprotein (HDL) without showing gene symbols**. Conjunctional –log_10_(FDR) values for high density lipoprotein (HDL) and Crohn’s Disease (CD), ulcerative colitis (UC), rheumatoid arthritis (RA), type 1 diabetes (T1D), celiac disease (CeD), psoriasis (PSOR) and G) sarcoidosis (SARC) were plotted along their chromosome locations. SNPs with conjunctional FDR < 0.05 (i.e., –log_10_ FDR > 1.3) are shown with enlarged data points. A black circle around the enlarged data points indicates the most significant SNP in each LD block. Details for the associated loci outside of chromosome 6 are shown in Table S7.

### Figure X. Effect direction of ‘pleiotropic’ SNP between high density lipoprotein (HDL) and immune-mediated diseases.

###

### Figure X. Z scores for high density lipoprotein (x axis) for ‘pleiotropic’ SNPs from Table S13 were plotted against z scores of SNP from immune-mediated disease (y axis). The panel headers indicate the immune-mediated diseases. The blue triangles indicate the ‘pleiotropic’ SNPs identified by standard GWAS (see Table S17). Crohn’s Disease (CD), ulcerative colitis (UC), rheumatoid arthritis (RA), celiac disease (CeD), psoriasis (PSOR) and sarcoidosis (SARC).

### Figure Y. Effect direction of ‘pleiotropic’ SNP between low density lipoprotein (LDL) and immune-mediated diseases.

###

### Figure Y. Z scores for low density lipoprotein (x-axis) for ‘pleiotropic’ SNPs from Table S12 were plotted against z scores of SNP from immune mediated disease (y axis). The panel headers indicate the immune-mediated diseases. The blue triangles indicate the ‘pleiotropic’ SNPs identified by standard GWAS (see Table S18). Crohn’s Disease (CD), ulcerative colitis (UC), rheumatoid arthritis (RA), celiac disease (CeD), psoriasis (PSOR) and sarcoidosis (SARC).

### Figure Z. Effect direction of ‘pleiotropic’ SNP between triglycerides (TG) and

**immune-mediated diseases.**

###

### Figure Z. Z scores for triglycerides (x axis) for ‘pleiotropic’ SNPs from Table S11 were plotted against z scores of SNP from immune mediated disease (y axis). The panel headers indicate the immune-mediated diseases. The blue triangles indicate the ‘pleiotropic’ SNPs identified by standard GWAS (see Table S19). Crohn’s Disease (CD), ulcerative colitis (UC), rheumatoid arthritis (RA), celiac disease (CeD), psoriasis (PSOR) and sarcoidosis (SARC).

### Table A. Summary data from all GWAS used in the current study.

| **Disease/Trait** | | **N** | **# SNPs** | **Reference** |
| --- | --- | --- | --- | --- |
| Triglycerides (TG) |  | 96,568 | 2,508,369 | Teslovich TM, Musunuru K, Smith AV, et al. Biological, clinical and population relevance of 95 loci for blood lipids. Nature 2010;466:707-13. |
| Low Density Lipoprotein (LDL) |  | 99,900 | 2,508,375 |
| High Density Lipoprotein (HDL) |  | 96,598 | 2,508,370 |
| Crohn’s Disease  (CD) |  | 51,109 | 942,858 | Franke, A. *et al.* Genome-wide meta-analysis increases to 71 the number of confirmed Crohn's disease susceptibility loci. *Nat Genet* **42**, 1118-25 (2010). |
| Ulcerative Colitis  (UC) |  | 26,405 | 1,273,589 | Anderson, C.A. *et al.* Meta-analysis identifies 29 additional ulcerative colitis risk loci, increasing the number of confirmed associations to 47. *Nat Genet* **43**, 246-52 (2011). |
| Rheumatoid Arthritis  (RA) |  | 25,708 | 2,554,714 | Stahl, E.A. *et al.* Genome-wide association study meta-analysis identifies seven new rheumatoid arthritis risk loci. *Nat Genet* **42**, 508-14 (2010). |
| Type 1 Diabetes (T1D) |  | 16,559 | 841,622 | Barrett, J.C. et al .Genome-wide association study and meta-analysis find that over 40 loci affect risk of type 1 diabetes. NatGen 2009, 41, 703-7. |
| Celiac disease (CeD) |  | 15,283 | 528,969 | Dubois, P.C. et al Multiple common variants for celiac disease influencing immune gene expression. Nat Genet 2010, 42, 295-302 |
| Psoriasis (PSOR) |  | 7,484 | 1,121,166 | Ellinghaus, D. *et al.* Combined analysis of genome-wide association studies for Crohn disease and psoriasis identifies seven shared susceptibility loci. *Am J Hum Genet* **90**, 636-47 (2012). |
| Sarcoidosis (SARC) |  | 2,153 | 1,294,967 | Fischer, A. *et al.* A Novel Sarcoidosis Risk Locus for Europeans on Chromosome 11q13.1. *Am J Respir Crit Care Med* (2012). |

For more details, see also <http://www.genome.gov/gwastudies>

### Table B. SNPs in triglycerides (TG) conditioned on immune-mediated diseases (condFDR<0.01).

| **# Locus** | **SNP** | **Chr** | **Gene**  **symbol** | **TG**  **P-value** | **TG**  **FDR** | **TG2**  **P-value** | **minFDR** | **Driving**  **phenotype** |
| --- | --- | --- | --- | --- | --- | --- | --- | --- |
| 1 | rs16837533 | 1 | *MACF1* | 7.85E-07 | 0.000658 | 3.873E-08 | 0.000293 | T1D |
|  | rs11586824 | 1 | *MACF1* | 6.54E-05 | 0.0272 | 0.0006074 | 0.00681 | T1D |
|  | rs4660808 | 1 | *LOC728448* | 1.84E-07 | 0.000195 | 4.58E-07 | 0.000242 | RA |
|  | rs2293476 | 1 | *PABPC4* | 3.48E-07 | 0.000295 | 8.64E-07 | 5.93E-05 | CD |
| 2 | rs10888768 | 1 | *SLC1A7* | 1.35E-05 | 0.00715 | 0.002192 | 0.00606 | RA |
| 3 | rs6587970 | 1 | *KANK4* | 2.08E-09 | 2.75E-06 | 9.648E-07 | 2.99E-06 | RA |
|  | rs6587971 | 1 | *KANK4* | 3.47E-09 | 4.28E-06 | 6.577E-09 | 1.66E-06 | CD |
|  | rs1473754 | 1 | *USP1* | 1.17E-08 | 1.56E-05 | 2.049E-06 | 1.89E-05 | RA |
|  | rs2095403 | 1 | *USP1* | 1.49E-08 | 1.93E-05 | 1.063E-08 | 1.63E-05 | UC |
|  | rs631106 | 1 | *USP1* | 1.37E-40 | 1.59E-07 | 6.204E-77 | 6.49E-09 | CD |
|  | rs10889332 | 1 | *DOCK7* | 7.28E-41 | 1.59E-07 | 3.295E-78 | 6.49E-09 | CD |
|  | rs1184865 | 1 | *DOCK7* | 6.24E-41 | 1.59E-07 | 4.424E-78 | 1.57E-07 | T1D |
|  | rs13375691 | 1 | *DOCK7* | 2.96E-09 | 4.28E-06 | 2.537E-16 | 5.29E-06 | RA |
|  | rs12048208 | 1 | *DOCK7* | 3.13E-11 | 1.59E-07 | 1.212E-11 | 3.68E-08 | CD |
|  | rs2031373 | 1 | *DOCK7* | 7.41E-22 | 1.59E-07 | 1.239E-26 | 2.91E-08 | CD |
|  | rs912540 | 1 | *DOCK7* | 3.59E-28 | 1.59E-07 | 1.829E-50 | 2.57E-08 | CD |
|  | rs12136083 | 1 | *DOCK7* | 7.32E-39 | 1.59E-07 | 7.51E-68 | 6.49E-09 | CD |
|  | rs9787151 | 1 | *DOCK7* | 1.90E-36 | 1.59E-07 | 1.301E-56 | 6.49E-09 | CD |
|  | rs6664692 | 1 | *DOCK7* | 2.92E-05 | 0.0156 | 1.105E-06 | 0.00423 | CD |
|  | rs11208012 | 1 | *ATG4C* | 1.43E-12 | 1.59E-07 | 6.962E-10 | 1.02E-07 | CD |
|  | rs11208024 | 1 | *ATG4C* | 3.90E-10 | 5.97E-07 | 3.808E-07 | 8.73E-08 | CD |
|  | rs11208027 | 1 | *ATG4C* | 6.40E-11 | 1.59E-07 | 1.431E-17 | 2.08E-07 | RA |
|  | rs12121207 | 1 | *ATG4C* | 8.65E-11 | 1.59E-07 | 1.904E-09 | 1.12E-07 | CD |
|  | rs12061691 | 1 | *ATG4C* | 4.48E-11 | 1.59E-07 | 2.22E-20 | 1.15E-07 | CeD |
|  | rs11208054 | 1 | *ATG4C* | 1.13E-11 | 1.59E-07 | 4.123E-20 | 1.97E-07 | RA |
|  | rs7512480 | 1 | *ATG4C* | 9.38E-10 | 1.43E-06 | 1.53E-14 | 8.25E-08 | CD |
| 4 | rs476769 | 1 | *SLC25A24* | 1.49E-06 | 0.00121 | 0.005581 | 0.000783 | PSOR |
|  | rs499424 | 1 | *SLC25A24* | 2.29E-06 | 0.00181 | 3.38E-06 | 0.00109 | UC |
|  | rs550136 | 1 | *SLC25A24* | 1.55E-06 | 0.00121 | 9.702E-07 | 0.00124 | RA |
|  | rs1012840 | 1 | *SLC25A24* | 4.18E-06 | 0.0027 | 0.006548 | 0.00185 | PSOR |
| 5 | rs4471211 | 1 | *NBPF1* | 2.11E-05 | 0.0105 | 9.716E-05 | 0.00543 | UC |
| 6 | rs1543358 | 1 | *TIPRL* | 3.79E-05 | 0.0188 | 7.024E-05 | 0.00832 | RA |
| 8 | rs12476708 | 2 | *AK123120* | 3.39E-05 | 0.0156 | 0.000217 | 0.00898 | RA |
| 9 | rs10469801 | 2 | *C2orf43* | 7.16E-06 | 0.00484 | 0.0003573 | 0.00584 | RA |
| 9 | rs6729843 | 2 | *C2orf43* | 1.21E-05 | 0.00715 | 1.685E-05 | 0.00599 | T1D |
| 11 | rs12714026 | 2 | *MAPRE3* | 2.13E-05 | 0.0105 | 1.26E-06 | 0.0039 | T1D |
| 13 | rs12993460 | 2 | *AK055918* | 1.86E-08 | 2.38E-05 | 6.019E-08 | 2.88E-05 | RA |
|  | rs906805 | 2 | *AK055918* | 3.43E-07 | 0.000295 | 8.026E-07 | 2.20E-05 | CD |
|  | rs12624279 | 2 | *FOSL2* | 2.23E-06 | 0.00148 | 3.9E-06 | 0.000104 | CD |
| 14 | rs11676985 | 2 | *FOSL2* | 2.05E-08 | 2.38E-05 | 7.479E-08 | 1.09E-06 | CD |
|  | rs10174692 | 2 | *FOSL2* | 1.48E-08 | 1.93E-05 | 3.613E-09 | 2.33E-05 | RA |
|  | rs2338013 | 2 | *FOSL2* | 1.84E-08 | 2.38E-05 | 3.654E-08 | 1.44E-06 | CD |
|  | rs2338437 | 2 | *FOSL2* | 1.10E-06 | 0.000805 | 2.178E-05 | 5.28E-05 | CD |
| 15 | rs10167952 | 2 | *ALK* | 2.87E-05 | 0.0156 | 5.397E-05 | 0.00242 | PSOR |
| 16 | rs2160069 | 2 | *BCL11A* | 4.61E-06 | 0.00328 | 0.0004598 | 0.00255 | T1D |
| 17 | rs2521920 | 2 | *LOC389033* | 1.93E-05 | 0.0105 | 0.002263 | 0.00538 | RA |
| 19 | rs9646748 | 2 | *MFSD6* | 2.06E-05 | 0.0105 | 0.007631 | 0.00829 | PSOR |
|  | rs4324342 | 2 | *MFSD6* | 4.73E-05 | 0.0227 | 0.0001533 | 0.0087 | UC |
| 20 | rs17725929 | 2 | *MAP2* | 5.45E-05 | 0.0227 | 0.0003658 | 0.00352 | CD |
| 22 | rs4241234 | 2 | *BC132948* | 4.65E-05 | 0.0227 | 0.002608 | 0.00866 | T1D |
| 23 | rs598747 | 3 | *SYN2* | 3.79E-05 | 0.0188 | 0.0001794 | 0.00343 | T1D |
| 24 | rs4135247 | 3 | *PPARG* | 2.49E-06 | 0.00181 | 0.0001395 | 0.001 | RA |
| 25 | rs17819328 | 3 | *PPARG* | 3.11E-07 | 0.000295 | 2.889E-10 | 0.000158 | T1D |
| 26 | rs13326165 | 3 | *STAB1* | 8.34E-06 | 0.00484 | 2.959E-06 | 0.00141 | T1D |
| 28 | rs16862394 | 3 | *PFN2* | 3.59E-05 | 0.0188 | 0.0006165 | 0.00494 | T1D |
|  | rs6789518 | 3 | *PFN2* | 4.03E-05 | 0.0188 | 0.0002475 | 0.00566 | CeD |
| 29 | rs16855567 | 3 | *EIF5A2* | 2.71E-05 | 0.0128 | 3.18E-06 | 0.00471 | T1D |
| 30 | rs762861 | 4 | *RGS12* | 3.71E-06 | 0.0027 | 9.681E-06 | 0.00324 | RA |
|  | rs6831256 | 4 | *DOK7* | 6.09E-06 | 0.00398 | 1.602E-12 | 0.000758 | CD |
| 31 | rs907496 | 4 | *C4orf52* | 0.000114 | 0.046 | 7.974E-05 | 0.00378 | RA |
| 33 | rs170869 | 4 | *SLC39A8* | 0.000312 | 0.0888 | 0.0008898 | 0.0096 | CD |
| 34 | rs12650033 | 4 | *ARHGAP10* | 1.99E-05 | 0.0105 | 0.007228 | 0.00899 | PSOR |
| 35 | rs12501328 | 4 | *DQ266889* | 4.21E-05 | 0.0188 | 0.03824 | 0.00511 | CD |
| 36 | rs17767636 | 5 | *FLJ33360* | 3.15E-05 | 0.0156 | 0.0149 | 0.00477 | T1D |
|  | rs12658401 | 5 | *FLJ33360* | 9.83E-06 | 0.00588 | 0.00303 | 0.00496 | RA |
|  | rs10069535 | 5 | *FLJ33360* | 2.97E-06 | 0.00221 | 3.749E-06 | 0.00181 | RA |
| 37 | rs959650 | 5 | *LOC729862* | 1.03E-05 | 0.00588 | 1.889E-05 | 0.00553 | RA |
| 38 | rs4957485 | 5 | *BC026261* | 2.40E-05 | 0.0128 | 0.0005522 | 0.00841 | T1D |
| 39 | rs6450176 | 5 | *ARL15* | 1.22E-05 | 0.00715 | 3.613E-07 | 0.00208 | UC |
| 40 | rs448666 | 5 | *ARL15* | 9.75E-06 | 0.00588 | 2.264E-05 | 0.00459 | T1D |
| 41 | rs16885555 | 5 | *ANKRD55* | 2.31E-05 | 0.0128 | 0.0002521 | 0.00848 | PSOR |
|  | rs30350 | 5 | *ANKRD55* | 1.89E-06 | 0.00148 | 9.456E-06 | 0.00137 | SARC |
| 42 | rs40271 | 5 | *ANKRD55* | 2.80E-05 | 0.0128 | 6.777E-06 | 0.00372 | RA |
| 44 | rs453701 | 5 | *ZNF366* | 7.48E-05 | 0.0324 | 0.001586 | 0.00789 | RA |
| 45 | rs6867167 | 5 | *AK056485* | 4.71E-05 | 0.0227 | 0.0002092 | 0.00442 | T1D |
|  | rs6889914 | 5 | *AK056485* | 1.36E-05 | 0.00715 | 0.0002935 | 0.00842 | RA |
| 46 | rs161645 | 5 | *CR610784* | 1.97E-05 | 0.0105 | 1.271E-05 | 0.00905 | RA |
|  | rs60271 | 5 | *CR610784* | 2.49E-05 | 0.0128 | 0.0001572 | 0.00834 | CeD |
| 47 | rs272869 | 5 | *SLC22A4* | 0.000104 | 0.0386 | 0.005645 | 0.00364 | CD |
| 50 | rs2076311 | 6 | *COL11A2* | 8.98E-07 | 0.000805 | 2.882E-06 | 7.37E-05 | RA |
|  | rs2072915 | 6 | *RXRB* | 8.88E-07 | 0.000658 | 2.363E-06 | 6.28E-05 | RA |
| 51 | rs462093 | 6 | *WDR46* | 6.02E-05 | 0.0272 | 8.873E-05 | 0.00178 | RA |
| 52 | rs7763535 | 6 | *FKBP5* | 0.000114 | 0.046 | 6.992E-05 | 0.00466 | RA |
| 53 | rs998584 | 6 | *VEGFA* | 2.46E-07 | 0.00024 | 3.424E-15 | 9.69E-05 | RA |
|  | rs1358980 | 6 | *VEGFA* | 3.88E-07 | 0.000361 | 6.02E-13 | 9.90E-05 | RA |
| 54 | rs2180314 | 6 | *GSTA2* | 4.51E-05 | 0.0227 | 3.3E-06 | 0.00692 | RA |
| 55 | rs2475251 | 6 | *BMP5* | 0.000232 | 0.0757 | 0.005989 | 0.00724 | PSOR |
| 56 | rs9285458 | 6 | *RSPO3* | 8.46E-06 | 0.00484 | 1.032E-06 | 0.000385 | CD |
|  | rs2326566 | 6 | *RSPO3* | 1.55E-05 | 0.00868 | 1.306E-06 | 0.000734 | CD |
| 57 | rs4897361 | 6 | *L3MBTL3* | 5.98E-06 | 0.00398 | 2.211E-06 | 0.00155 | UC |
|  | rs6928313 | 6 | *L3MBTL3* | 1.36E-05 | 0.00715 | 2.994E-06 | 0.00322 | UC |
| 58 | rs668459 | 6 | *AK123801* | 2.01E-06 | 0.00148 | 9.893E-14 | 0.000772 | CD |
|  | rs628751 | 6 | *AK123801* | 3.40E-07 | 0.000295 | 2.059E-13 | 0.000162 | CD |
| 59 | rs3105748 | 6 | *SLC22A3* | 2.89E-07 | 0.000295 | 7.27E-09 | 0.000236 | CD |
|  | rs2665357 | 6 | *SLC22A3* | 3.03E-07 | 0.000295 | 8.327E-10 | 0.000236 | CD |
|  | rs3127596 | 6 | *LPA* | 9.74E-08 | 0.000104 | 1.241E-07 | 7.75E-05 | SARC |
|  | rs3124785 | 6 | *LPA* | 8.18E-08 | 8.42E-05 | 6.163E-08 | 0.000102 | RA |
|  | rs11751605 | 6 | *LPA* | 3.45E-06 | 0.00221 | 0.0001536 | 0.000532 | UC |
| 60 | rs852425 | 7 | *ACTG1* | 0.000156 | 0.0544 | 0.0009047 | 0.00568 | PSOR |
| 61 | rs11762757 | 7 | *ISPD* | 0.000102 | 0.0386 | 6.758E-05 | 0.00981 | T1D |
| 62 | rs12154905 | 7 | *DM004234* | 1.01E-06 | 0.000805 | 4.564E-09 | 0.000461 | CD |
|  | rs6951827 | 7 | *DM004234* | 4.93E-07 | 0.00044 | 8.975E-07 | 0.000515 | RA |
| 63 | rs2908290 | 7 | *GCK* | 1.94E-05 | 0.0105 | 1.453E-06 | 0.00287 | CD |
| 66 | rs2530492 | 7 | *POM121* | 1.72E-05 | 0.00868 | 0.0002315 | 0.00592 | RA |
| 69 | rs38855 | 7 | *MET* | 0.000101 | 0.0386 | 2.109E-08 | 0.00996 | CD |
| 70 | rs7787531 | 7 | *AHCYL2* | 9.42E-06 | 0.00588 | 0.006156 | 0.00224 | CD |
|  | rs2402951 | 7 | *AHCYL2* | 1.18E-05 | 0.00715 | 0.006765 | 0.00271 | CD |
| 71 | rs1596972 | 7 | *KLF14* | 3.04E-07 | 0.000295 | 1.234E-08 | 0.000274 | CD |
|  | rs1562398 | 7 | *KLF14* | 1.82E-07 | 0.000195 | 3.266E-08 | 0.000219 | RA |
| 74 | rs4279608 | 8 | *CSGALNACT1* | 1.35E-06 | 0.000985 | 2.339E-06 | 0.00077 | UC |
|  | rs4628268 | 8 | *CSGALNACT1* | 2.39E-06 | 0.00181 | 1.641E-09 | 0.00151 | UC |
|  | rs7003476 | 8 | *CSGALNACT1* | 3.54E-06 | 0.00221 | 1.009E-07 | 0.000735 | CD |
| 76 | rs13263508 | 8 | *SLC18A1* | 2.42E-26 | 1.59E-07 | 3.383E-48 | 1.38E-07 | SARC |
|  | rs13265868 | 8 | *SLC18A1* | 2.94E-25 | 1.59E-07 | 1.766E-41 | 2.23E-08 | SARC |
| 77 | rs7003526 | 8 | *SLC18A1* | 9.55E-06 | 0.00588 | 1.12E-07 | 0.00292 | UC |
| 78 | rs16842 | 8 | *SLC18A1* | 1.21E-07 | 0.000128 | 6.325E-13 | 8.51E-05 | CD |
| 79 | rs41364444 | 8 | *STMN4* | 1.67E-05 | 0.00868 | 2.604E-05 | 0.00326 | PSOR |
| 80 | rs2303525 | 8 | *KIAA0196* | 0.000101 | 0.0386 | 0.0001364 | 0.00432 | T1D |
| 86 | rs7033354 | 9 | *BNC2* | 9.23E-06 | 0.00588 | 4.441E-07 | 0.00268 | CD |
| 87 | rs1323467 | 9 | *DQ673934* | 0.00011 | 0.0386 | 0.0002166 | 0.00981 | T1D |
| 88 | rs1937849 | 10 | *AKR1C3* | 6.38E-06 | 0.00398 | 8.872E-06 | 0.00443 | RA |
|  | rs17134531 | 10 | *AKR1C4* | 4.84E-07 | 0.00044 | 2.747E-07 | 0.00033 | CD |
|  | rs17134533 | 10 | *AKR1C4* | 5.41E-07 | 0.00044 | 7.2E-12 | 0.000425 | T1D |
|  | rs17134601 | 10 | *AKR1C4* | 4.69E-07 | 0.00044 | 1.065E-07 | 0.00033 | CD |
|  | rs9423393 | 10 | *AKR1C4* | 4.30E-06 | 0.0027 | 5.958E-07 | 0.000363 | CeD |
|  | rs6601936 | 10 | *AKR1C4* | 1.32E-06 | 0.000985 | 1.089E-06 | 0.00115 | RA |
|  | rs2398235 | 10 | *AKR1C4* | 3.59E-06 | 0.0027 | 6.739E-06 | 0.000846 | T1D |
| 89 | rs11101342 | 10 | *ARHGAP22* | 0.00032 | 0.0888 | 0.04549 | 0.00705 | PSOR |
| 91 | rs4617527 | 10 | *C10orf41* | 4.80E-06 | 0.00328 | 1.233E-05 | 0.00272 | CD |
| 93 | rs1962270 | 10 | *GPAM* | 1.00E-06 | 0.000805 | 2.044E-07 | 0.000967 | RA |
| 94 | rs11195943 | 10 | *ACSL5* | 4.55E-05 | 0.0227 | 9.02E-07 | 0.00713 | PSOR |
| 95 | rs6485690 | 11 | *CKAP5* | 5.96E-07 | 0.000538 | 2.061E-07 | 0.000205 | T1D |
|  | rs2697920 | 11 | *MYBPC3* | 4.43E-05 | 0.0188 | 3.255E-06 | 0.00222 | PSOR |
| 96 | rs2453712 | 11 | *RPLP0L2* | 5.29E-06 | 0.00328 | 1.825E-05 | 0.000817 | CD |
|  | rs198435 | 11 | *DAGLA* | 2.03E-06 | 0.00148 | 1.722E-06 | 0.00176 | RA |
|  | rs4963243 | 11 | *DAGLA* | 7.49E-06 | 0.00484 | 0.0003508 | 0.00185 | CD |
|  | rs2240287 | 11 | *DAGLA* | 6.00E-06 | 0.00398 | 0.0002619 | 0.00154 | CD |
| 98 | rs7479857 | 11 | *FGF3* | 0.000115 | 0.046 | 0.001759 | 0.00682 | CeD |
| 99 | rs495033 | 11 | *BUD13* | 2.55E-07 | 0.00024 | 5.057E-07 | 0.00019 | T1D |
| 100 | rs11599994 | 11 | *BUD13* | 5.58E-06 | 0.00328 | 5.005E-07 | 0.00308 | CD |
|  | rs11215972 | 11 | *BUD13* | 2.15E-05 | 0.0105 | 0.0001397 | 0.00698 | T1D |
| 101 | rs11605293 | 11 | *BUD13* | 0.000191 | 0.0643 | 0.0002071 | 0.00758 | UC |
| 102 | rs7122363 | 11 | *BUD13* | 1.97E-06 | 0.00148 | 8.134E-08 | 0.000534 | CD |
|  | rs10891996 | 11 | *BUD13* | 1.07E-06 | 0.000805 | 2.235E-06 | 0.00045 | RA |
| 103 | rs1240658 | 11 | *BUD13* | 1.03E-05 | 0.00588 | 3.012E-12 | 0.00282 | PSOR |
| 104 | rs519000 | 11 | *BUD13* | 7.35E-06 | 0.00484 | 2.085E-07 | 0.00436 | T1D |
| 106 | rs7928525 | 11 | *RNF214* | 1.76E-06 | 0.00121 | 5.204E-14 | 0.000475 | RA |
|  | rs1056136 | 11 | *RNF214* | 4.98E-06 | 0.00328 | 3.147E-06 | 0.00137 | RA |
| 107 | rs10750360 | 11 | *BC030092* | 3.14E-06 | 0.00221 | 0.001151 | 0.00158 | T1D |
| 108 | rs3782640 | 12 | *ANO2* | 7.11E-06 | 0.00484 | 3.204E-05 | 0.00411 | SARC |
|  | rs11063843 | 12 | *ANO2* | 7.04E-06 | 0.00398 | 0.0004897 | 0.00372 | RA |
| 109 | rs11046505 | 12 | *ETNK1* | 8.83E-07 | 0.000658 | 6.567E-06 | 0.000445 | PSOR |
|  | rs11046525 | 12 | *ETNK1* | 6.85E-07 | 0.000538 | 2.39E-05 | 0.000369 | PSOR |
|  | rs10743443 | 12 | *ETNK1* | 3.24E-07 | 0.000295 | 8.655E-06 | 0.000235 | PSOR |
| 110 | rs10842564 | 12 | *IFLTD1* | 7.81E-06 | 0.00484 | 0.004431 | 0.00036 | RA |
| 112 | rs2227492 | 12 | *IL22* | 6.12E-05 | 0.0272 | 0.0006826 | 0.0095 | UC |
| 113 | rs10861661 | 12 | *RIC8B* | 0.000136 | 0.046 | 2.598E-07 | 0.00816 | CD |
| 114 | rs7297186 | 12 | *CUX2* | 0.000274 | 0.0757 | 0.0003165 | 0.00862 | T1D |
|  | rs7398833 | 12 | *CUX2* | 0.000164 | 0.0544 | 0.0002191 | 0.00611 | T1D |
| 116 | rs7995033 | 13 | *MTMR6* | 3.52E-05 | 0.0156 | 0.0002192 | 0.00793 | CD |
| 117 | rs9600212 | 13 | *KLF12* | 1.29E-14 | 1.59E-07 | 0.000785 | 1.79E-07 | PSOR |
| 118 | rs17505711 | 13 | *FGF14* | 8.74E-05 | 0.0324 | 0.01403 | 0.00761 | PSOR |
| 119 | rs1059264 | 14 | *SLC25A29* | 6.10E-06 | 0.00398 | 0.0004025 | 0.00255 | CD |
| 122 | rs11855184 | 15 | *DMXL2* | 7.77E-06 | 0.00484 | 0.0005878 | 0.0057 | RA |
| 125 | rs1007076 | 15 | *LACTB* | 6.31E-05 | 0.0272 | 2.537E-07 | 0.00533 | CD |
|  | rs2729835 | 15 | *LACTB* | 0.000106 | 0.0386 | 5.023E-07 | 0.00981 | T1D |
| 126 | rs17184382 | 15 | *USP3* | 1.09E-05 | 0.00588 | 6.942E-06 | 0.00184 | T1D |
|  | rs4074448 | 15 | *BC036918* | 5.56E-07 | 0.00044 | 6.591E-05 | 0.000246 | CD |
|  | rs10519220 | 15 | *HERC1* | 1.36E-06 | 0.000985 | 0.000335 | 0.000711 | RA |
| 127 | rs8033122 | 15 | *ZWILCH* | 6.66E-05 | 0.0272 | 0.00129 | 0.00738 | RA |
|  | rs2119264 | 15 | *BC016970* | 3.02E-05 | 0.0156 | 7.429E-06 | 0.00898 | RA |
| 128 | rs1035744 | 15 | *BRUNOL6* | 1.25E-05 | 0.00715 | 1.448E-07 | 0.00715 | CD |
|  | rs11633028 | 15 | *BRUNOL6* | 7.07E-06 | 0.00398 | 1.726E-05 | 0.00373 | CeD |
|  | rs8030477 | 15 | *AK127877* | 2.40E-06 | 0.00181 | 5.758E-06 | 0.00213 | RA |
|  | rs7164727 | 15 | *AK127877* | 5.74E-06 | 0.00398 | 1.857E-05 | 0.00231 | CeD |
|  | rs2415168 | 15 | *AK127877* | 3.25E-06 | 0.00221 | 2.775E-07 | 0.00164 | SARC |
| 129 | rs4985124 | 16 | *PDXDC1* | 2.89E-06 | 0.00221 | 2.33E-07 | 0.00042 | UC |
|  | rs7200543 | 16 | *PDXDC1* | 4.49E-06 | 0.00328 | 2.656E-07 | 0.000721 | UC |
| 133 | rs2000999 | 16 | *TXNL4B* | 3.78E-06 | 0.0027 | 7.488E-07 | 0.000371 | T1D |
| 134 | rs8060878 | 16 | *PMFBP1* | 5.36E-06 | 0.00328 | 7.351E-05 | 0.00112 | T1D |
| 135 | rs12927703 | 16 | *BC042734* | 8.45E-05 | 0.0324 | 0.002567 | 0.0098 | T1D |
| 136 | rs11642655 | 16 | *CMIP* | 1.15E-05 | 0.00715 | 2.254E-06 | 0.00673 | RA |
| 137 | rs2086247 | 17 | *ARL4D* | 1.10E-05 | 0.00588 | 0.002733 | 0.00352 | RA |
| 138 | rs8077889 | 17 | *MPP3* | 6.77E-06 | 0.00398 | 9.879E-09 | 0.00174 | UC |
| 139 | rs16948048 | 17 | *ZNF652* | 3.09E-05 | 0.0156 | 4.797E-07 | 0.00643 | CD |
| 140 | rs9903638 | 17 | *CA10* | 3.96E-05 | 0.0188 | 0.0001248 | 0.00467 | CD |
| 141 | rs7221651 | 17 | *BPTF* | 5.79E-05 | 0.0272 | 9.784E-05 | 0.00306 | T1D |
| 142 | rs9948620 | 18 | *C18orf1* | 2.41E-05 | 0.0128 | 1.746E-05 | 0.00902 | CD |
| 143 | rs11663816 | 18 | *MC4R* | 2.53E-06 | 0.00181 | 0.0006967 | 0.000602 | PSOR |
|  | rs489693 | 18 | *MC4R* | 2.93E-07 | 0.000295 | 0.0008889 | 0.000267 | RA |
|  | rs12964203 | 18 | *MC4R* | 4.08E-06 | 0.0027 | 0.0009037 | 0.000886 | PSOR |
| 144 | rs3752240 | 19 | *ABCA7* | 3.51E-05 | 0.0156 | 0.06405 | 0.00388 | T1D |
| 145 | rs10426094 | 19 | *INSR* | 1.27E-07 | 0.000128 | 8.468E-09 | 3.34E-05 | T1D |
|  | rs7508679 | 19 | *INSR* | 1.06E-06 | 0.000805 | 2.219E-09 | 0.000312 | CD |
|  | rs7248104 | 19 | *INSR* | 4.13E-07 | 0.000361 | 5.045E-10 | 0.000133 | CD |
| 146 | rs8101064 | 19 | *INSR* | 1.09E-06 | 0.000805 | 1.91E-06 | 0.000687 | SARC |
| 147 | rs3810444 | 19 | *SFRS14* | 2.98E-05 | 0.0156 | 1.571E-16 | 0.00766 | T1D |
|  | rs3890384 | 19 | *MEF2B* | 1.22E-07 | 0.000128 | 4.55E-06 | 6.59E-05 | T1D |
|  | rs3819578 | 19 | *LOC729991-MEF2B* | 2.84E-08 | 3.63E-05 | 1.238E-06 | 1.42E-05 | CD |
|  | rs2228603 | 19 | *NCAN* | 2.23E-24 | 1.59E-07 | 1.739E-57 | 1.27E-07 | T1D |
|  | rs2238675 | 19 | *NCAN* | 1.04E-15 | 1.59E-07 | 2.226E-37 | 1.46E-07 | CD |
|  | rs8105094 | 19 | *HAPLN4* | 1.63E-12 | 1.59E-07 | 6.687E-29 | 6.11E-08 | CD |
|  | rs8105984 | 19 | *HAPLN4* | 2.33E-12 | 1.59E-07 | 1.288E-27 | 5.54E-08 | CD |
|  | rs735273 | 19 | *HAPLN4* | 3.44E-08 | 3.63E-05 | 4.881E-17 | 3.54E-06 | RA |
|  | rs968525 | 19 | *KIAA0892* | 4.59E-11 | 1.59E-07 | 8.543E-24 | 6.11E-08 | CD |
|  | rs2965185 | 19 | *GATAD2A* | 1.09E-09 | 1.43E-06 | 1.646E-22 | 2.35E-07 | RA |
|  | rs2304130 | 19 | *ZNF101* | 6.13E-16 | 1.59E-07 | 5.434E-39 | 1.23E-07 | CD |
|  | rs17699261 | 19 | *ZNF14* | 3.31E-06 | 0.00221 | 1.447E-16 | 0.00217 | SARC |
| 148 | rs1688043 | 19 | *HPN* | 8.33E-06 | 0.00484 | 1.63E-06 | 0.00426 | CD |
| 149 | rs6508747 | 19 | *BC042630* | 0.000127 | 0.046 | 0.0004391 | 0.00815 | PSOR |
|  | rs7250821 | 19 | *WDR87* | 0.000125 | 0.046 | 0.0001693 | 0.00815 | PSOR |
| 150 | rs3208856 | 19 | *CBLC* | 8.08E-06 | 0.00484 | 1.55E-05 | 0.00281 | PSOR |
| 152 | rs7259004 | 19 | *EF553526* | 6.66E-07 | 0.000538 | 8.96E-25 | 0.000646 | RA |
| 153 | rs12721109 | 19 | *APOC4* | 5.99E-07 | 0.000538 | 0.000133 | 0.000665 | RA |
|  | rs3760627 | 19 | *CLPTM1* | 9.19E-07 | 0.000805 | 5.293E-09 | 0.00045 | RA |
|  | rs3760629 | 19 | *CLPTM1* | 4.56E-07 | 0.00044 | 7.859E-09 | 0.000246 | CD |
|  | rs16979600 | 19 | *CLPTM1* | 2.13E-06 | 0.00148 | 1.622E-06 | 0.0013 | PSOR |
| 154 | rs10408163 | 19 | *ZC3H4* | 3.54E-05 | 0.0156 | 6.759E-07 | 0.00188 | UC |
|  | rs11669142 | 19 | *ZC3H4* | 6.44E-06 | 0.00398 | 0.0004479 | 0.00195 | UC |
|  | rs307896 | 19 | *SAE1* | 2.62E-05 | 0.0128 | 0.00499 | 0.00202 | T1D |
| 155 | rs676388 | 19 | *FUT2* | 5.49E-05 | 0.0227 | 8.274E-05 | 0.002 | CD |
|  | rs6111051 | 20 | *KIF16B* | 1.03E-05 | 0.00588 | 7.115E-05 | 0.00693 | RA |
|  | rs4814468 | 20 | *KIF16B* | 1.04E-05 | 0.00588 | 0.01975 | 0.00484 | CD |
|  | rs4814475 | 20 | *KIF16B* | 1.28E-05 | 0.00715 | 0.02099 | 0.00599 | T1D |
| 157 | rs6059958 | 20 | *HM13* | 0.000103 | 0.0386 | 0.003028 | 0.00673 | CeD |
| 158 | rs2378199 | 20 | *PIGU* | 4.12E-07 | 0.000361 | 3.264E-06 | 0.000185 | CeD |
|  | rs6060030 | 20 | *NCOA6* | 6.68E-08 | 6.82E-05 | 8.609E-07 | 5.81E-05 | T1D |
|  | rs11546155 | 20 | *GGT7* | 4.48E-08 | 5.53E-05 | 1.723E-06 | 4.69E-05 | CD |
| 159 | rs6029143 | 20 | *MAFB* | 2.83E-06 | 0.00221 | 4.933E-08 | 0.00158 | CD |
| 160 | rs6016399 | 20 | *MAFB* | 1.34E-05 | 0.00715 | 0.0005569 | 0.00842 | RA |
|  | rs6129653 | 20 | *MAFB* | 1.93E-05 | 0.0105 | 1.651E-05 | 0.00773 | UC |
| 161 | rs3795131 | 20 | *PLCG1* | 9.91E-06 | 0.00588 | 0.0006786 | 0.00104 | CD |
|  | rs6102322 | 20 | *ZHX3* | 1.28E-05 | 0.00715 | 9.942E-06 | 0.00117 | CD |
| 163 | rs1206773 | 20 | *EYA2* | 1.11E-06 | 0.000805 | 7.479E-05 | 0.000137 | RA |
|  | rs6066149 | 20 | *EYA2* | 2.65E-07 | 0.00024 | 5.222E-06 | 0.00015 | RA |
| 164 | rs2835676 | 21 | *DSCR9* | 3.42E-05 | 0.0156 | 0.000174 | 0.00871 | PSOR |
| 166 | rs4821815 | 22 | *GTPBP1* | 0.000306 | 0.0888 | 0.000116 | 0.00692 | RA |
| 7 | rs2144300 | 1 | *GALNT2** | 1.76E-13 | 1.59E-07 | 4.102E-30 | 1.17E-07 | T1D |
|  | rs17315646 | 1 | *GALNT2** | 7.63E-14 | 1.59E-07 | 2.757E-27 | 5.02E-08 | CD |
|  | rs2281719 | 1 | *GALNT2** | 4.72E-14 | 1.59E-07 | 4.762E-30 | 4.55E-08 | CD |
|  | rs10779835 | 1 | *GALNT2** | 3.68E-14 | 1.59E-07 | 3.816E-29 | 4.55E-08 | CD |
|  | rs10489615 | 1 | *GALNT2** | 9.98E-14 | 1.59E-07 | 3.178E-13 | 4.55E-08 | CD |
| 10 | rs4341893 | 2 | *APOB** | 1.15E-09 | 1.78E-06 | 7.622E-12 | 1.41E-06 | CD |
|  | rs9306897 | 2 | *APOB** | 6.51E-10 | 9.25E-07 | 1.82E-12 | 8.41E-07 | T1D |
|  | rs11096689 | 2 | *APOB** | 7.53E-11 | 1.59E-07 | 6.568E-12 | 1.52E-07 | PSOR |
|  | rs4533439 | 2 | *APOB** | 4.65E-10 | 7.43E-07 | 5.943E-17 | 5.86E-07 | CD |
|  | rs13414987 | 2 | *APOB** | 3.97E-09 | 5.34E-06 | 6.033E-12 | 4.94E-06 | CD |
|  | rs3923037 | 2 | *APOB** | 5.79E-20 | 1.59E-07 | 6.749E-28 | 6.77E-08 | CD |
|  | rs2337382 | 2 | *APOB** | 5.83E-10 | 9.25E-07 | 1.226E-15 | 5.48E-07 | UC |
|  | rs4665639 | 2 | *APOB** | 2.46E-11 | 1.59E-07 | 6.586E-16 | 6.73E-08 | UC |
|  | rs17041662 | 2 | *APOB** | 1.14E-11 | 1.59E-07 | 3.922E-18 | 9.93E-08 | UC |
|  | rs11902417 | 2 | *APOB** | 3.58E-45 | 1.59E-07 | 6.679E-67 | 1.12E-07 | CD |
|  | rs10172650 | 2 | *APOB** | 5.50E-09 | 6.63E-06 | 1.414E-11 | 4.84E-06 | CD |
|  | rs13392272 | 2 | *APOB** | 1.85E-23 | 1.59E-07 | 3.399E-27 | 1.34E-07 | CD |
|  | rs676210 | 2 | *APOB** | 3.48E-47 | 1.59E-07 | 3.284E-71 | 8.38E-08 | CeD |
|  | rs693 | 2 | *APOB** | 1.34E-23 | 1.59E-07 | 2.167E-30 | 1.34E-07 | CD |
|  | rs533617 | 2 | *APOB** | 1.25E-15 | 1.59E-07 | 6.879E-27 | 1.37E-07 | RA |
|  | rs673548 | 2 | *APOB** | 7.95E-47 | 1.59E-07 | 1.972E-68 | 8.98E-08 | CeD |
|  | rs11126598 | 2 | *APOB** | 1.28E-27 | 1.59E-07 | 6.652E-39 | 1.26E-07 | UC |
|  | rs11676704 | 2 | *APOB** | 8.00E-09 | 1.02E-05 | 1.794E-06 | 8.08E-06 | UC |
|  | rs1469513 | 2 | *APOB** | 3.06E-15 | 1.59E-07 | 2.239E-15 | 1.37E-07 | RA |
|  | rs520354 | 2 | *APOB** | 1.43E-12 | 1.59E-07 | 8.163E-13 | 1.23E-07 | CD |
|  | rs1367117 | 2 | *APOB** | 1.93E-10 | 3.08E-07 | 1.057E-11 | 2.39E-07 | RA |
|  | rs1429974 | 2 | *APOB** | 1.67E-07 | 0.000158 | 4.569E-07 | 8.91E-05 | RA |
|  | rs12471982 | 2 | *APOB** | 3.11E-05 | 0.0156 | 2.883E-06 | 0.00325 | CD |
|  | rs4635554 | 2 | *APOB** | 7.00E-10 | 9.25E-07 | 2.007E-08 | 1.08E-06 | RA |
| 12 | rs7588926 | 2 | *CGREF1* | 3.85E-09 | 5.34E-06 | 1.394E-15 | 4.38E-07 | UC |
|  | rs1275539 | 2 | *TCF23* | 2.56E-11 | 1.59E-07 | 4.736E-16 | 6.11E-08 | RA |
|  | rs2580754 | 2 | *TCF23* | 7.43E-18 | 1.59E-07 | 4.431E-19 | 1.12E-07 | CD |
|  | rs1659682 | 2 | *SLC5A6* | 3.28E-11 | 1.59E-07 | 1.282E-20 | 7.34E-08 | RA |
|  | rs1659700 | 2 | *SLC5A6* | 1.06E-11 | 1.59E-07 | 8.958E-12 | 7.34E-08 | RA |
|  | rs1275526 | 2 | *SLC5A6* | 2.72E-11 | 1.59E-07 | 2.245E-11 | 6.91E-08 | RA |
|  | rs13399758 | 2 | *CAD* | 6.04E-12 | 1.59E-07 | 9.376E-21 | 9.99E-08 | PSOR |
|  | rs2304684 | 2 | *CAD* | 3.31E-08 | 3.63E-05 | 5.004E-11 | 4.28E-05 | RA |
|  | rs1561535 | 2 | *CAD* | 2.02E-17 | 1.59E-07 | 1.965E-33 | 1.57E-07 | T1D |
|  | rs6759518 | 2 | *SLC30A3* | 4.64E-12 | 1.59E-07 | 1.866E-21 | 1.17E-07 | PSOR |
|  | rs11686131 | 2 | *TRIM54* | 4.23E-12 | 1.59E-07 | 2.503E-11 | 9.93E-08 | UC |
|  | rs4665965 | 2 | *MPV17* | 1.34E-21 | 1.59E-07 | 2.886E-38 | 1.12E-07 | UC |
|  | rs1975384 | 2 | *MPV17* | 5.30E-12 | 1.59E-07 | 3.128E-11 | 8.75E-08 | UC |
|  | rs1049817 | 2 | *GTF3C2* | 1.91E-37 | 1.59E-07 | 7.329E-63 | 9.63E-09 | UC |
|  | rs6743819 | 2 | *GTF3C2* | 9.18E-37 | 1.59E-07 | 7.297E-63 | 9.63E-09 | UC |
|  | rs1058065 | 2 | *EIF2B4* | 1.21E-05 | 0.00715 | 1.6E-06 | 0.00895 | RA |
|  | rs2384629 | 2 | *PPM1G* | 1.59E-22 | 1.59E-07 | 1.026E-21 | 1.49E-07 | PSOR |
|  | rs11675428 | 2 | *NRBP1* | 2.62E-20 | 1.59E-07 | 5.244E-25 | 2.19E-08 | UC |
|  | rs4665978 | 2 | *NRBP1* | 4.23E-54 | 1.59E-07 | 2.77E-90 | 1.95E-07 | RA |
|  | rs6747190 | 2 | *IFT172* | 7.16E-08 | 8.42E-05 | 5.382E-09 | 0.000101 | RA |
|  | rs8179219 | 2 | *GCKR** | 7.22E-11 | 1.59E-07 | 1.713E-18 | 1.12E-07 | CD |
|  | rs1260326* | 2 | *GCKR** | 6.76E-138 | 1.59E-07 | 2.29E-239 | 6.49E-09 | CD |
|  | rs3817588 | 2 | *GCKR** | 3.51E-40 | 1.59E-07 | 1.295E-55 | 3.68E-08 | CD |
|  | rs780094 | 2 | *GCKR** | 1.70E-129 | 1.59E-07 | 2.65E-220 | 2.19E-08 | UC |
|  | rs780093 | 2 | *GCKR** | 1.47E-128 | 1.59E-07 | 6.17E-220 | 6.49E-09 | CD |
|  | rs780092 | 2 | *GCKR** | 3.89E-35 | 1.59E-07 | 1.996E-46 | 4.10E-08 | CD |
|  | rs4665381 | 2 | *GCKR** | 9.78E-11 | 1.59E-07 | 5.43E-14 | 9.63E-09 | UC |
|  | rs2068834 | 2 | *ZNF512* | 6.06E-64 | 1.59E-07 | 8.73E-100 | 9.63E-09 | UC |
|  | rs2141371 | 2 | *GPN1* | 1.68E-37 | 1.59E-07 | 9.2E-49 | 1.26E-07 | UC |
|  | rs17706100 | 2 | *SUPT7L* | 2.06E-10 | 3.08E-07 | 3.591E-20 | 3.15E-07 | PSOR |
|  | rs2272406 | 2 | *SLC4A1AP* | 1.38E-45 | 1.59E-07 | 1.115E-63 | 9.68E-08 | RA |
|  | rs2178198 | 2 | *SLC4A1AP* | 9.51E-31 | 1.59E-07 | 1.039E-55 | 7.17E-08 | CeD |
|  | rs13023094 | 2 | *SLC4A1AP* | 7.32E-45 | 1.59E-07 | 7.929E-71 | 9.63E-09 | UC |
|  | rs1881394 | 2 | *AK124439* | 2.34E-15 | 1.59E-07 | 4.808E-22 | 7.09E-08 | CeD |
|  | rs867282 | 2 | *AK124439* | 4.02E-31 | 1.59E-07 | 2.59E-45 | 6.11E-08 | CD |
|  | rs4233716 | 2 | *RBKS* | 1.17E-12 | 1.59E-07 | 9.776E-12 | 1.12E-07 | CD |
|  | rs6547811 | 2 | *RBKS* | 7.69E-12 | 1.59E-07 | 2.627E-12 | 2.05E-07 | RA |
|  | rs17758146 | 2 | *BRE* | 1.05E-09 | 1.43E-06 | 7.161E-13 | 7.57E-08 | UC |
|  | rs898031 | 2 | *BRE* | 9.51E-10 | 1.43E-06 | 6.377E-15 | 7.57E-08 | UC |
|  | rs13016086 | 2 | *BRE* | 2.33E-08 | 2.94E-05 | 2.328E-07 | 2.65E-05 | PSOR |
|  | rs6547829 | 2 | *BRE* | 7.13E-10 | 1.15E-06 | 7.445E-20 | 7.70E-07 | CD |
|  | rs4401177 | 2 | *BRE* | 9.88E-19 | 1.59E-07 | 2.399E-37 | 6.49E-09 | CD |
|  | rs6751559 | 2 | *BRE* | 1.69E-11 | 1.59E-07 | 1.562E-22 | 4.56E-08 | UC |
|  | rs6710247 | 2 | *BRE* | 4.11E-11 | 1.59E-07 | 6.664E-23 | 4.03E-08 | UC |
|  | rs13000936 | 2 | *BRE* | 3.53E-09 | 4.28E-06 | 5.939E-14 | 3.22E-06 | RA |
|  | rs11891642 | 2 | *BRE* | 4.61E-06 | 0.00328 | 3.47E-09 | 0.00307 | CeD |
|  | rs4666042 | 2 | *BRE* | 7.63E-09 | 1.02E-05 | 8.583E-17 | 2.37E-06 | CD |
|  | rs7349418 | 2 | *BRE* | 5.81E-18 | 1.59E-07 | 1.063E-28 | 6.49E-09 | CD |
|  | rs12617913 | 2 | *BRE* | 3.89E-13 | 1.59E-07 | 4.546E-20 | 6.49E-09 | CD |
|  | rs12621972 | 2 | *BRE* | 8.09E-06 | 0.00484 | 6.737E-09 | 0.00426 | CD |
|  | rs4666052 | 2 | *BRE* | 2.25E-15 | 1.59E-07 | 4.479E-21 | 6.49E-09 | CD |
|  | rs17739364 | 2 | *BRE* | 3.88E-05 | 0.0188 | 4.914E-05 | 0.00279 | UC |
|  | rs1565326 | 2 | *BRE* | 5.15E-07 | 0.00044 | 3.168E-11 | 0.000221 | CD |
|  | rs884437 | 2 | *BRE* | 4.93E-06 | 0.00328 | 2.295E-05 | 0.000249 | CD |
|  | rs10184619 | 2 | *BRE* | 4.33E-06 | 0.0027 | 0.0001253 | 0.000227 | PSOR |
|  | rs2175095 | 2 | *AK055918* | 7.01E-06 | 0.00398 | 0.0001009 | 0.00054 | CD |
|  | rs871521 | 2 | *AK055918* | 4.68E-06 | 0.00328 | 8.489E-05 | 0.000371 | CD |
| 18 | rs10195252* | 2 | *COBLL1** | 7.44E-11 | 1.59E-07 | 6.987E-15 | 3.68E-08 | CD |
|  | rs1128249 | 2 | *COBLL1** | 1.03E-10 | 1.59E-07 | 2.754E-15 | 4.55E-08 | CD |
| 21 | rs2943645* | 2 | *BC017935* | 1.29E-08 | 1.56E-05 | 3.756E-15 | 6.01E-06 | T1D |
|  | rs2972146 | 2 | *BC017935* | 1.36E-08 | 1.56E-05 | 2.974E-15 | 1.88E-05 | RA |
| 27 | rs645040* | 3 | *MSL2** | 1.38E-08 | 1.56E-05 | 1.83E-12 | 1.56E-05 | CD |
|  | rs687339 | 3 | *MSL2** | 1.83E-08 | 2.38E-05 | 2.508E-12 | 2.38E-05 | CD |
|  | rs9820513 | 3 | *STAG1* | 1.64E-05 | 0.00868 | 2.564E-05 | 0.00813 | CD |
|  | rs6771713 | 3 | *STAG1* | 3.19E-05 | 0.0156 | 7.596E-05 | 0.0081 | UC |
|  | rs1872240 | 3 | *STAG1* | 2.97E-05 | 0.0156 | 7.598E-05 | 0.00906 | CeD |
| 32 | rs4299551 | 4 | *MAPK10* | 7.01E-07 | 0.000538 | 1.495E-06 | 0.000312 | RA |
|  | rs17409478 | 4 | *MAPK10* | 5.28E-07 | 0.00044 | 2.131E-06 | 0.000206 | RA |
|  | rs13149938 | 4 | *SLC10A6* | 1.68E-07 | 0.000158 | 4.61E-07 | 0.000146 | CD |
|  | rs13106574 | 4 | *SLC10A6* | 1.52E-07 | 0.000158 | 6.652E-07 | 0.000135 | CD |
|  | rs1992876 | 4 | *AFF1* | 4.69E-07 | 0.00044 | 2.788E-10 | 0.00044 | CD |
|  | rs236995 | 4 | *AFF1* | 1.03E-10 | 1.59E-07 | 7.002E-15 | 2.01E-07 | RA |
|  | rs236996 | 4 | *AFF1* | 8.25E-11 | 1.59E-07 | 3.782E-16 | 1.46E-07 | PSOR |
|  | rs342438 | 4 | *AFF1* | 1.97E-11 | 1.59E-07 | 1.908E-18 | 1.46E-07 | CD |
|  | rs1037814 | 4 | *AFF1* | 2.51E-11 | 1.59E-07 | 2.842E-18 | 1.46E-07 | CD;PSOR |
|  | rs6847980 | 4 | *KLHL8** | 1.04E-08 | 1.26E-05 | 3.438E-08 | 6.29E-06 | UC |
|  | rs6817297 | 4 | *HSD17B13* | 5.45E-09 | 6.63E-06 | 3.747E-08 | 6.98E-06 | UC |
|  | rs11735092 | 4 | *HSD17B13* | 1.01E-08 | 1.26E-05 | 2.265E-12 | 3.67E-06 | UC |
| 43 | rs3843467 | 5 | *MAP3K1** | 4.81E-10 | 7.43E-07 | 4.339E-15 | 2.94E-07 | CD |
|  | rs9686661* | 5 | *MAP3K1** | 5.96E-11 | 1.59E-07 | 2.541E-16 | 2.05E-07 | RA |
| 48 | rs7724832 | 5 | *TIMD4** | 1.93E-11 | 1.59E-07 | 1.405E-13 | 6.49E-09 | CD |
|  | rs1363232 | 5 | *TIMD4** | 2.34E-11 | 1.59E-07 | 6.172E-15 | 6.49E-09 | CD |
|  | rs1501908 | 5 | *TIMD4** | 6.84E-11 | 1.59E-07 | 8.288E-15 | 6.49E-09 | CD |
|  | rs4704728 | 5 | *TIMD4** | 2.54E-06 | 0.00181 | 2.7E-06 | 0.000892 | RA |
|  | rs1501909 | 5 | *hHAVcr-1* | 1.52E-06 | 0.00121 | 8.875E-11 | 0.000568 | CD |
|  | rs1553318* | 5 | *hHAVcr-1* | 1.46E-12 | 1.59E-07 | 8.977E-13 | 1.69E-07 | RA |
| 49 | rs13194781 | 6 | *HIST1H2BN* | 0.000112 | 0.0386 | 9.458E-05 | 0.00217 | PSOR |
|  | rs6922111 | 6 | *ZKSCAN3* | 0.000231 | 0.0757 | 0.000253 | 0.0062 | RA |
|  | rs2746150 | 6 | *OR2H1* | 0.000126 | 0.046 | 7.169E-07 | 0.00259 | PSOR |
|  | rs1235162 | 6 | *GABBR1* | 3.76E-05 | 0.0188 | 3.041E-06 | 0.00216 | T1D |
|  | rs2844762 | 6 | *HLA** | 0.000272 | 0.0757 | 0.001345 | 0.00447 | PSOR |
|  | rs3132625 | 6 | *TRIM39* | 1.50E-06 | 0.00121 | 1.346E-09 | 8.02E-05 | PSOR |
|  | rs9262143 | 6 | *KIAA1949* | 1.05E-06 | 0.000805 | 3.88E-06 | 9.20E-05 | PSOR |
|  | rs9468830 | 6 | *C6orf214* | 0.000349 | 0.0888 | 0.02519 | 0.00538 | PSOR |
|  | rs7752959 | 6 | *C6orf214* | 0.000229 | 0.0757 | 0.0009852 | 0.00572 | RA |
|  | rs1264344 | 6 | *C6orf214* | 3.24E-05 | 0.0156 | 2.551E-07 | 0.000912 | PSOR |
|  | rs2517544 | 6 | *HCG22* | 5.00E-07 | 0.00044 | 2.212E-05 | 2.83E-05 | RA |
|  | rs2248386 | 6 | *HCG22* | 8.76E-06 | 0.00484 | 4.527E-08 | 0.000288 | PSOR |
|  | rs3130544 | 6 | *C6orf15* | 2.30E-10 | 3.85E-07 | 8.434E-15 | 9.18E-08 | CD |
|  | rs9263565 | 6 | *C6orf15* | 2.24E-06 | 0.00181 | 2.975E-06 | 9.76E-05 | RA |
|  | rs3130991 | 6 | *PSORS1C1* | 1.90E-08 | 2.38E-05 | 1.744E-08 | 1.30E-06 | UC |
|  | rs3094205 | 6 | *PSORS1C1* | 2.20E-08 | 2.38E-05 | 8.764E-13 | 1.30E-06 | UC |
|  | rs1265098 | 6 | *PSORS1C1* | 2.19E-07 | 0.000195 | 6.853E-13 | 1.49E-05 | PSOR |
|  | rs1966 | 6 | *PSORS1C1* | 4.07E-09 | 5.34E-06 | 7.051E-13 | 6.13E-07 | PSOR |
|  | rs1265089 | 6 | *PSORS1C1* | 3.23E-09 | 4.28E-06 | 1.361E-08 | 6.09E-07 | RA |
|  | rs2073717 | 6 | *CCHCR1* | 4.02E-05 | 0.0188 | 0.0009589 | 0.00109 | PSOR |
|  | rs2239524 | 6 | *CCHCR1* | 6.03E-05 | 0.0272 | 4.384E-05 | 0.00154 | PSOR |
|  | rs879882 | 6 | *POU5F1* | 7.70E-05 | 0.0324 | 7.218E-05 | 0.00183 | PSOR |
|  | rs9264594 | 6 | *HLA** | 1.57E-14 | 1.59E-07 | 4.584E-13 | 2.74E-08 | RA |
|  | rs9264601 | 6 | *HLA** | 5.09E-14 | 1.59E-07 | nan | 2.74E-08 | RA |
|  | rs9264603 | 6 | *HLA** | 7.04E-14 | 1.59E-07 | 4.059E-06 | 2.74E-08 | RA |
|  | rs13200569 | 6 | *HLA** | 1.68E-10 | 2.47E-07 | 1.683E-09 | 4.22E-08 | RA |
|  | rs2524074 | 6 | *HLA** | 3.97E-14 | 1.59E-07 | 3.011E-10 | 6.49E-09 | CD |
|  | rs9366778 | 6 | *HLA** | 1.03E-09 | 1.43E-06 | 3.47E-17 | 2.81E-07 | PSOR |
|  | rs6931332 | 6 | *HLA** | 0.00018 | 0.0643 | 0.0004237 | 0.00372 | PSOR |
|  | rs9378249 | 6 | *HLA** | 9.59E-05 | 0.0386 | 0.0002373 | 0.00263 | RA |
|  | rs2523554 | 6 | *HLA** | 8.29E-05 | 0.0324 | 0.01158 | 0.00298 | CD |
|  | rs7761068 | 6 | *HLA** | 0.000116 | 0.046 | 0.01047 | 0.00839 | UC |
|  | rs2844575 | 6 | *HLA** | 3.96E-05 | 0.0188 | 8.085E-08 | 0.00121 | RA |
|  | rs9295986 | 6 | *HLA** | 0.000166 | 0.0544 | 1.907E-06 | 0.00388 | RA |
|  | rs2516440 | 6 | *HCG26* | 8.61E-06 | 0.00484 | 7.992E-06 | 0.000278 | RA |
|  | rs4413654 | 6 | *HCG26* | 0.000258 | 0.0757 | 0.002289 | 0.00486 | PSOR |
|  | rs2844503 | 6 | *HCG26* | 0.000158 | 0.0544 | 0.0002227 | 0.00388 | RA |
|  | rs2259435 | 6 | *MCCD1* | 4.81E-07 | 0.00044 | 3.301E-08 | 2.66E-05 | CD |
|  | rs2516478 | 6 | *BAT1* | 1.37E-07 | 0.000128 | 3.596E-09 | 6.74E-06 | CD |
|  | rs2071592 | 6 | *NFKBIL1* | 0.000193 | 0.0643 | 0.0001134 | 0.00471 | RA |
|  | rs9469027 | 6 | *LST1* | 8.12E-05 | 0.0324 | 0.0003808 | 0.00216 | RA |
|  | rs2857700 | 6 | *AIF1* | 3.67E-05 | 0.0188 | 3.992E-06 | 0.00118 | PSOR |
|  | rs9267531 | 6 | *CSNK2B* | 1.80E-10 | 3.08E-07 | 2.602E-14 | 1.30E-08 | CD |
|  | rs4569 | 6 | *CSNK2B* | 2.01E-06 | 0.00148 | 2.587E-06 | 0.000765 | SARC |
|  | rs2142234 | 6 | *CSNK2B* | 2.96E-07 | 0.000295 | 4.951E-07 | 2.09E-05 | RA |
|  | rs9267546 | 6 | *BAT5* | 3.27E-08 | 3.63E-05 | 7.741E-10 | 3.54E-06 | RA |
|  | rs385306 | 6 | *BAT5* | 1.36E-05 | 0.00715 | 2.817E-05 | 0.000426 | RA |
|  | rs15574 | 6 | *LY6G6C* | 3.07E-06 | 0.00221 | 3.761E-09 | 0.000173 | RA |
|  | rs1065356 | 6 | *LY6G6C* | 2.26E-06 | 0.00181 | 1.17E-09 | 0.000158 | RA |
|  | rs805292 | 6 | *LY6G6C* | 2.02E-06 | 0.00148 | 1.394E-09 | 0.000147 | RA |
|  | rs453098 | 6 | *C6orf25* | 4.22E-07 | 0.000361 | 6.363E-10 | 2.43E-05 | RA |
|  | rs3117577 | 6 | *MSH5* | 1.24E-10 | 1.98E-07 | 3.273E-14 | 8.18E-09 | CD |
|  | rs3130481 | 6 | *SLC44A4* | 4.86E-09 | 6.63E-06 | 6.275E-08 | 8.68E-07 | RA |
|  | rs3869145 | 6 | *NG36/G9a* | 2.51E-08 | 2.94E-05 | 9.042E-15 | 2.97E-06 | RA |
|  | rs659445 | 6 | *NG36/G9a* | 6.10E-15 | 1.59E-07 | 6.468E-12 | 1.65E-08 | SARC |
|  | rs2734335 | 6 | *C2* | 4.31E-05 | 0.0188 | 1.952E-06 | 0.00109 | PSOR |
|  | rs2072633 | 6 | *CFB* | 0.000174 | 0.0544 | 0.00293 | 0.0031 | PSOR |
|  | rs440454 | 6 | *SKIV2L* | 1.88E-14 | 1.59E-07 | 8.917E-13 | 9.63E-09 | UC |
|  | rs389883 | 6 | *STK19* | 1.22E-15 | 1.59E-07 | 1.917E-15 | 9.63E-09 | UC |
|  | rs9267798 | 6 | *TNXB* | 0.000336 | 0.0888 | 0.0004258 | 0.00538 | PSOR |
|  | rs429150 | 6 | *TNXB* | 8.49E-05 | 0.0324 | 0.001758 | 0.00183 | PSOR |
|  | rs3134603 | 6 | *PPT2* | 9.22E-07 | 0.000805 | 1.771E-06 | 4.52E-05 | RA |
|  | rs3131294 | 6 | *NOTCH4* | 3.37E-06 | 0.00221 | 9.126E-06 | 0.00012 | RA |
|  | rs384247 | 6 | *NOTCH4* | 0.000192 | 0.0643 | 9.826E-05 | 0.00471 | RA |
|  | rs405875 | 6 | *AK123889* | 3.47E-07 | 0.000295 | 1.056E-07 | 1.91E-05 | UC |
|  | rs3130309 | 6 | *AK123889* | 2.09E-11 | 1.59E-07 | 1.464E-09 | 2.74E-08 | RA |
|  | rs9267992 | 6 | *AK123889* | 6.80E-07 | 0.000538 | 1.919E-05 | 3.28E-05 | RA |
|  | rs3115572 | 6 | *AK123889* | 8.92E-11 | 1.59E-07 | 6.599E-09 | 2.74E-08 | RA |
|  | rs3132959 | 6 | *C6orf10* | 0.000182 | 0.0643 | 0.01304 | 0.00471 | RA |
|  | rs2143462 | 6 | *C6orf10* | 4.22E-07 | 0.000361 | 3.659E-07 | 2.37E-05 | UC |
|  | rs3129933 | 6 | *C6orf10* | 7.74E-07 | 0.000658 | 5.819E-06 | 3.83E-05 | RA |
|  | rs9268384 | 6 | *C6orf10* | 1.87E-07 | 0.000195 | 1.335E-09 | 1.51E-05 | RA |
|  | rs9268402 | 6 | *C6orf10* | 0.000199 | 0.0643 | 0.0001928 | 0.00372 | PSOR |
|  | rs6930777 | 6 | *BTNL2* | 0.00022 | 0.0643 | 0.0003911 | 0.00471 | RA |
|  | rs17423649 | 6 | *BTNL2* | 2.38E-05 | 0.0128 | 7.424E-07 | 0.00118 | UC |
|  | rs3129883 | 6 | *HLA** | 0.000216 | 0.0643 | 0.0006115 | 0.00372 | PSOR |
|  | rs3135005 | 6 | *HLA** | 6.27E-07 | 0.000538 | 2.795E-06 | 3.28E-05 | RA |
|  | rs9271366 | 6 | *HLA** | 8.20E-07 | 0.000658 | 2.378E-05 | 3.83E-05 | RA |
|  | rs9272535 | 6 | *HLA** | 8.50E-05 | 0.0324 | 0.0001972 | 0.00216 | RA |
|  | rs9273363 | 6 | *HLA** | 5.22E-05 | 0.0227 | 1.415E-08 | 0.00147 | RA |
|  | rs11752643 | 6 | *HLA** | 8.37E-11 | 1.59E-07 | 3.96E-19 | 2.74E-08 | RA |
|  | rs9275572 | 6 | *HLA** | 9.05E-05 | 0.0386 | 0.002556 | 0.00217 | PSOR |
|  | rs2858331 | 6 | *HLA** | 0.000523 | 0.121 | 0.001079 | 0.00781 | PSOR |
|  | rs3892710 | 6 | *HLA** | 6.88E-05 | 0.0272 | 3.653E-08 | 0.00178 | RA |
|  | rs9276731 | 6 | *HLA** | 1.19E-06 | 0.000985 | 3.136E-06 | 5.41E-05 | RA |
|  | rs4148876 | 6 | *TAP2* | 4.54E-07 | 0.00044 | 2.076E-06 | 2.83E-05 | RA |
|  | rs11756897 | 6 | *HLA-DMB* | 2.19E-05 | 0.0105 | 0.0001684 | 0.000655 | RA |
|  | rs23544 | 6 | *HLA-DMB* | 0.000412 | 0.104 | 1.669E-05 | 0.00649 | PSOR |
| 64 | rs12667443 | 7 | *TYW1B** | 2.05E-05 | 0.0105 | 2.965E-07 | 0.00543 | UC |
|  | rs2909969 | 7 | *TYW1B** | 1.77E-05 | 0.00868 | 1.528E-07 | 0.0053 | T1D |
| 65 | rs13238203* | 7 | *TYW1B** | 5.54E-10 | 7.43E-07 | 3.07E-06 | 9.76E-07 | RA |
|  | rs10248063 | 7 | *FKBP6* | 1.66E-05 | 0.00868 | 2.85E-08 | 0.00397 | RA |
|  | rs42131 | 7 | *FZD9* | 2.57E-25 | 1.59E-07 | 2.419E-37 | 1.69E-07 | RA |
|  | rs2240466 | 7 | *BAZ1B* | 7.87E-57 | 1.59E-07 | 5.27E-90 | 2.27E-08 | CD |
|  | rs1178979 | 7 | *BAZ1B* | 4.01E-58 | 1.59E-07 | 1.97E-97 | 7.96E-09 | CD |
|  | rs2237279 | 7 | *BAZ1B* | 4.64E-14 | 1.59E-07 | 2.567E-21 | 6.11E-08 | CD |
|  | rs2074754 | 7 | *BAZ1B* | 2.18E-12 | 1.59E-07 | 1.17E-17 | 6.11E-08 | CD |
|  | rs17145721 | 7 | *BAZ1B* | 2.04E-05 | 0.0105 | 0.0002046 | 0.00314 | CD |
|  | rs3763432 | 7 | *BCL7B* | 5.70E-11 | 1.59E-07 | 2.227E-18 | 7.51E-08 | CD |
|  | rs17145738 | 7 | *TBL2* | 4.65E-60 | 1.59E-07 | 9.42E-99 | 2.57E-08 | CD |
|  | rs11974409 | 7 | *TBL2* | 9.17E-61 | 1.59E-07 | 1.36E-100 | 8.84E-09 | CD |
|  | rs7808877 | 7 | *MLXIPL** | 1.58E-38 | 1.59E-07 | 3.5E-66 | 2.01E-07 | RA |
|  | rs7785479 | 7 | *MLXIPL** | 1.87E-11 | 1.59E-07 | 1.311E-48 | 2.27E-08 | CD |
|  | rs17145813 | 7 | *MLXIPL** | 4.43E-34 | 1.59E-07 | 1.118E-41 | 1.57E-07 | PSOR |
|  | rs17401675 | 7 | *MLXIPL** | 7.58E-06 | 0.00484 | 8.469E-08 | 0.00185 | CD |
| 68 | rs799161 | 7 | *MLXIPL** | 3.14E-16 | 1.59E-07 | 1.682E-28 | 7.51E-08 | CD |
|  | rs799160 | 7 | *MLXIPL** | 1.15E-18 | 1.59E-07 | 5.459E-30 | 1.12E-07 | CD |
|  | rs1128349 | 7 | *DNAJC30* | 2.11E-05 | 0.0105 | 8.996E-12 | 0.00701 | PSOR |
| 72 | rs2979151 | 8 | *PRAGMIN* | 4.60E-05 | 0.0227 | 0.0004818 | 0.00719 | T1D |
|  | rs2979146 | 8 | *PRAGMIN* | 1.34E-05 | 0.00715 | 4.055E-07 | 0.00519 | PSOR |
|  | rs898137 | 8 | *AK055863* | 4.01E-07 | 0.000361 | 1.942E-07 | 0.000438 | RA |
|  | rs9693857 | 8 | *AK055863* | 7.18E-07 | 0.000658 | 1.691E-08 | 0.000231 | CD |
|  | rs13282752 | 8 | *DM004390* | 1.27E-05 | 0.00715 | 0.0001169 | 0.00842 | RA |
|  | rs583484 | 8 | *DM004390* | 5.39E-07 | 0.00044 | 8.207E-08 | 0.000191 | UC |
|  | rs615171 | 8 | *DM004390* | 4.65E-07 | 0.00044 | 7.269E-08 | 0.00018 | CD |
|  | rs7840785 | 8 | *SOX7* | 2.25E-08 | 2.94E-05 | 4.888E-12 | 1.60E-05 | CD |
|  | rs2271357 | 8 | *SOX7* | 8.47E-09 | 1.02E-05 | 4.197E-11 | 7.43E-06 | CD |
|  | rs11776767* | 8 | *SOX7* | 6.94E-09 | 8.23E-06 | 2.873E-11 | 8.12E-06 | RA |
|  | rs7812865 | 8 | *XKR6* | 1.79E-05 | 0.0105 | 0.0009271 | 0.00989 | CD |
|  | rs11990550 | 8 | *XKR6* | 2.30E-07 | 0.00024 | 2.942E-07 | 0.000171 | T1D |
|  | rs4841504 | 8 | *XKR6* | 9.55E-06 | 0.00588 | 1.242E-07 | 0.000351 | PSOR |
|  | rs7004825 | 8 | *XKR6* | 1.65E-06 | 0.00121 | 0.0003555 | 0.00144 | RA |
|  | rs7813802 | 8 | *XKR6* | 2.58E-06 | 0.00181 | 0.0004996 | 0.000936 | T1D |
|  | rs2618451 | 8 | *BLK* | 5.75E-06 | 0.00398 | 2.599E-07 | 0.00206 | T1D |
|  | rs17744726 | 8 | *BLK* | 0.000144 | 0.0544 | 1.87E-07 | 0.00987 | T1D |
|  | rs12677146 | 8 | *BLK* | 1.10E-05 | 0.00588 | 0.0004702 | 0.00294 | SARC |
|  | rs11787286 | 8 | *GATA4* | 1.59E-05 | 0.00868 | 3.233E-05 | 0.0082 | RA |
|  | rs10903342 | 8 | *GATA4* | 2.24E-06 | 0.00181 | 0.0001399 | 0.000758 | T1D |
|  | rs1004712 | 8 | *C8orf49* | 2.37E-07 | 0.00024 | 5.692E-09 | 0.000118 | CD |
|  | rs1466785 | 8 | *C8orf49* | 2.21E-07 | 0.000195 | 6.89E-06 | 4.04E-05 | T1D |
|  | rs1296023 | 8 | *CTSB* | 0.000116 | 0.046 | 0.0006093 | 0.00504 | UC |
|  | rs4604485 | 8 | *DEFB134* | 8.12E-06 | 0.00484 | 0.0001091 | 0.00429 | SARC |
| 73 | rs13254048 | 8 | *NAT2** | 7.93E-06 | 0.00484 | 0.000659 | 0.00496 | PSOR |
|  | rs6993895 | 8 | *NAT2** | 2.02E-07 | 0.000195 | 1.486E-06 | 0.000236 | RA |
|  | rs6983849 | 8 | *NAT2** | 2.16E-06 | 0.00148 | 1.781E-05 | 0.000956 | CD |
|  | rs10088180 | 8 | *NAT2** | 1.54E-11 | 1.59E-07 | 3.867E-08 | 1.22E-07 | RA |
|  | rs1961456 | 8 | *NAT2** | 1.43E-11 | 1.59E-07 | 5.486E-13 | 2.74E-08 | RA |
|  | rs721399 | 8 | *NAT2** | 2.26E-12 | 1.59E-07 | 7.746E-14 | 2.74E-08 | RA |
|  | rs1495745 | 8 | *NAT2** | 2.55E-12 | 1.59E-07 | 3.178E-12 | 2.74E-08 | RA |
|  | rs13277394 | 8 | *NAT2** | 1.94E-05 | 0.0105 | 3.221E-05 | 0.00905 | RA |
|  | rs2410561 | 8 | *NAT2** | 1.21E-09 | 1.78E-06 | 9.249E-10 | 6.05E-07 | RA |
|  | rs1495743* | 8 | *NAT2** | 1.38E-14 | 1.59E-07 | 2.273E-12 | 2.74E-08 | RA |
|  | rs1466753 | 8 | *NAT2** | 1.99E-06 | 0.00148 | 4.037E-07 | 0.000658 | SARC |
| 75 | rs10085966 | 8 | *CSGALNACT1* | 1.70E-11 | 1.59E-07 | 1.151E-13 | 1.34E-07 | CD |
|  | rs7010610 | 8 | *CSGALNACT1* | 2.73E-10 | 3.85E-07 | 1.56E-18 | 2.63E-07 | CeD |
|  | rs11204072 | 8 | *INTS10* | 9.13E-11 | 1.59E-07 | 1.084E-16 | 6.11E-08 | CD |
|  | rs2278615 | 8 | *INTS10* | 0.000418 | 0.104 | 0.000527 | 0.00834 | RA |
|  | rs4244456 | 8 | *INTS10* | 6.03E-07 | 0.000538 | 1.141E-06 | 0.000201 | SARC |
|  | rs6586872 | 8 | *INTS10* | 1.79E-10 | 3.08E-07 | 4.086E-19 | 2.85E-07 | CD;SARC |
|  | rs1441778 | 8 | *INTS10* | 8.51E-12 | 1.59E-07 | 6.77E-13 | 1.59E-07 | CD |
|  | rs7003579 | 8 | *INTS10* | 4.39E-06 | 0.0027 | 4.716E-10 | 0.00313 | RA |
|  | rs4518684 | 8 | *INTS10* | 4.10E-06 | 0.0027 | 7.583E-08 | 0.0021 | UC |
|  | rs4922113 | 8 | *INTS10* | 1.27E-25 | 1.59E-07 | 9.889E-42 | 4.54E-08 | SARC |
|  | rs6586876 | 8 | *INTS10* | 1.11E-08 | 1.26E-05 | 7.522E-13 | 7.66E-06 | CD |
|  | rs17410407 | 8 | *INTS10* | 9.19E-26 | 1.59E-07 | 7.44E-36 | 1.26E-07 | UC |
|  | rs17482310 | 8 | *INTS10* | 1.40E-27 | 1.59E-07 | 6.46E-44 | 5.15E-08 | SARC |
|  | rs7844579 | 8 | *INTS10* | 9.97E-06 | 0.00588 | 2.998E-10 | 0.00449 | CD |
|  | rs3898938 | 8 | *INTS10* | 3.02E-08 | 3.63E-05 | 1.219E-12 | 1.58E-05 | T1D |
|  | rs10102717 | 8 | *LPL** | 4.91E-14 | 1.59E-07 | 9.662E-20 | 2.01E-07 | RA |
|  | rs1441771 | 8 | *LPL** | 1.76E-30 | 1.59E-07 | 1.605E-50 | 3.97E-08 | SARC |
|  | rs6586879 | 8 | *LPL** | 8.48E-15 | 1.59E-07 | 1.812E-23 | 1.34E-07 | CD |
|  | rs6996383 | 8 | *LPL** | 4.25E-16 | 1.59E-07 | 9.89E-15 | 1.34E-07 | CD |
|  | rs3988302 | 8 | *LPL** | 7.30E-16 | 1.59E-07 | 3.06E-21 | 1.34E-07 | CD |
|  | rs3900537 | 8 | *LPL** | 3.93E-16 | 1.59E-07 | 5.511E-20 | 1.34E-07 | CD |
|  | rs253 | 8 | *LPL** | 1.64E-18 | 1.59E-07 | 1.462E-31 | 1.46E-07 | CD |
|  | rs263 | 8 | *LPL** | 2.06E-43 | 1.59E-07 | 1.075E-87 | 1.12E-07 | CD |
|  | rs268 | 8 | *LPL** | 6.19E-17 | 1.59E-07 | 2.766E-15 | 1.12E-07 | UC |
|  | rs285 | 8 | *LPL** | 4.11E-35 | 1.59E-07 | 4.801E-56 | 2.04E-07 | RA |
|  | rs316 | 8 | *LPL** | 1.84E-12 | 1.59E-07 | 1.804E-12 | 1.34E-07 | CD |
|  | rs10099160 | 8 | *LPL** | 1.58E-05 | 0.00868 | 7.815E-11 | 0.00435 | RA |
|  | rs4922115 | 8 | *LPL** | 4.48E-16 | 1.59E-07 | 1.482E-23 | 1.46E-07 | CD |
|  | rs3289 | 8 | *LPL** | 2.40E-20 | 1.59E-07 | 3.671E-33 | 1.47E-07 | PSOR |
|  | rs9644636 | 8 | *LPL** | 2.46E-19 | 1.59E-07 | 2.02E-27 | 1.34E-07 | CD |
|  | rs17482753 | 8 | *LPL** | 9.42E-114 | 1.59E-07 | 5.79E-193 | 8.78E-08 | T1D |
|  | rs1441777 | 8 | *LPL** | 8.23E-16 | 1.59E-07 | 1.31E-23 | 1.46E-07 | CD |
|  | rs12678919* | 8 | *LPL** | 8.04E-120 | 1.59E-07 | 1.82E-199 | 8.33E-08 | CD |
|  | rs17410962 | 8 | *LPL** | 1.55E-95 | 1.59E-07 | 2.26E-172 | 8.33E-08 | CD |
|  | rs17489268 | 8 | *LPL** | 1.46E-109 | 1.59E-07 | 3.05E-166 | 5.89E-08 | UC |
|  | rs11992311 | 8 | *LPL** | 4.71E-10 | 7.43E-07 | 1.617E-12 | 5.65E-07 | UC |
|  | rs2410622 | 8 | *LPL** | 1.54E-15 | 1.59E-07 | 1.203E-26 | 1.67E-07 | PSOR |
|  | rs1441766 | 8 | *LPL** | 7.17E-76 | 1.59E-07 | 2.551E-70 | 6.11E-08 | CD |
|  | rs1899351 | 8 | *LPL** | 3.06E-08 | 3.63E-05 | 2.045E-12 | 1.06E-05 | UC |
|  | rs1441759 | 8 | *LPL** | 1.15E-08 | 1.56E-05 | 2.85E-11 | 1.92E-05 | RA |
|  | rs894210 | 8 | *LPL** | 3.59E-56 | 1.59E-07 | 2.94E-89 | 1.23E-07 | CD |
|  | rs7005359 | 8 | *LPL** | 3.47E-46 | 1.59E-07 | 1.862E-57 | 7.51E-08 | CD |
|  | rs2410629 | 8 | *LPL** | 3.35E-42 | 1.59E-07 | 4.036E-58 | 1.27E-07 | T1D |
|  | rs2410630 | 8 | *LPL** | 1.01E-50 | 1.59E-07 | 1.025E-88 | 1.27E-07 | T1D |
|  | rs1534650 | 8 | *LPL** | 1.92E-24 | 1.59E-07 | 2.161E-36 | 1.46E-07 | CD |
|  | rs10103634 | 8 | *LPL** | 4.49E-30 | 1.59E-07 | 8.089E-46 | 1.32E-07 | T1D |
|  | rs4557718 | 8 | *LPL** | 7.46E-17 | 1.59E-07 | 3.264E-24 | 1.97E-07 | RA |
|  | rs4244457 | 8 | *LPL** | 2.54E-28 | 1.59E-07 | 7.722E-51 | 1.23E-07 | CD |
|  | rs6586891 | 8 | *SLC18A1* | 1.43E-43 | 1.59E-07 | 9.207E-66 | 1.11E-07 | T1D |
|  | rs4128744 | 8 | *SLC18A1* | 1.01E-78 | 1.59E-07 | 1.19E-71 | 1.12E-07 | CD |
| 81 | rs4871594 | 8 | *TRIB1** | 1.56E-05 | 0.00868 | 9.473E-09 | 0.0074 | RA |
| 82 | rs2385114 | 8 | *TRIB1** | 4.16E-20 | 1.59E-07 | 6.174E-28 | 4.10E-08 | CD |
|  | rs4871598 | 8 | *TRIB1** | 5.79E-17 | 1.59E-07 | 5.12E-28 | 8.12E-08 | SARC |
|  | rs6470355 | 8 | *TRIB1** | 1.43E-13 | 1.59E-07 | 9.862E-17 | 4.56E-08 | UC |
|  | rs4518686 | 8 | *TRIB1** | 7.98E-24 | 1.59E-07 | 1.245E-29 | 8.12E-08 | SARC |
|  | rs12678799 | 8 | *TRIB1** | 6.33E-13 | 1.59E-07 | 2.164E-18 | 3.57E-08 | UC |
|  | rs4871601 | 8 | *TRIB1** | 8.59E-09 | 1.02E-05 | 3.35E-14 | 8.96E-06 | RA |
|  | rs2980885 | 8 | *TRIB1** | 5.35E-15 | 1.59E-07 | 2.997E-40 | 7.51E-08 | CD |
|  | rs2954018 | 8 | *TRIB1** | 1.80E-32 | 1.59E-07 | 6.6E-75 | 2.57E-08 | CD |
|  | rs2001945 | 8 | *TRIB1** | 4.72E-44 | 1.59E-07 | 2.619E-86 | 5.02E-08 | CD |
|  | rs4871603 | 8 | *TRIB1** | 1.80E-29 | 1.59E-07 | 3.13E-64 | 6.11E-08 | CD |
|  | rs2954020 | 8 | *TRIB1** | 4.67E-30 | 1.59E-07 | 1.173E-69 | 3.57E-08 | UC |
|  | rs8180991 | 8 | *TRIB1** | 4.01E-10 | 5.97E-07 | 6.818E-14 | 1.13E-07 | UC |
| 83 | rs7012891 | 8 | *TRIB1** | 1.20E-13 | 1.59E-07 | 9.204E-24 | 1.12E-07 | UC |
|  | rs7832357 | 8 | *TRIB1** | 7.72E-12 | 1.59E-07 | 3.571E-11 | 6.73E-08 | UC |
|  | rs4512391 | 8 | *TRIB1** | 4.30E-13 | 1.59E-07 | 1.886E-17 | 8.33E-08 | CD |
|  | rs1809167 | 8 | *TRIB1** | 1.84E-07 | 0.000195 | 1.077E-11 | 3.47E-05 | CD |
| 84 | rs7004305 | 8 | *TRIB1** | 2.02E-06 | 0.00148 | 3.684E-05 | 0.000693 | T1D |
| 85 | rs4871624 | 8 | *TRIB1** | 2.10E-08 | 2.38E-05 | 1.07E-11 | 1.88E-05 | CD |
| 90 | rs10733788 | 10 | *NRBF2* | 1.33E-06 | 0.000985 | 1.763E-05 | 0.000337 | CeD |
|  | rs6479889 | 10 | *JMJD1C** | 1.09E-05 | 0.00588 | 0.0001967 | 0.00243 | T1D |
|  | rs10761739 | 10 | *JMJD1C** | 3.87E-12 | 1.59E-07 | 1.728E-10 | 1.02E-07 | CD |
|  | rs7083356 | 10 | *JMJD1C** | 9.37E-07 | 0.000805 | 4.431E-05 | 0.000967 | RA |
|  | rs7897379 | 10 | *REEP3* | 3.07E-12 | 1.59E-07 | 1.271E-17 | 1.02E-07 | CD |
|  | rs3847326 | 10 | *REEP3* | 4.29E-11 | 1.59E-07 | 1.401E-10 | 1.12E-07 | CD |
|  | rs12768534 | 10 | *REEP3* | 5.93E-11 | 1.59E-07 | 6.77E-10 | 1.22E-07 | RA |
| 92 | rs2185757 | 10 | *EXOC6* | 5.07E-07 | 0.00044 | 5.489E-05 | 0.000148 | RA |
|  | rs2497337 | 10 | *EXOC6* | 6.02E-07 | 0.000538 | 0.0001388 | 3.95E-05 | UC |
|  | rs10748583 | 10 | *EXOC6* | 7.17E-06 | 0.00484 | 1.755E-05 | 0.00213 | UC |
|  | rs11593751 | 10 | *EXOC6* | 3.99E-05 | 0.0188 | 0.00217 | 0.00345 | UC |
|  | rs1409386 | 10 | *EXOC6* | 9.43E-06 | 0.00588 | 3.635E-06 | 0.00205 | RA |
|  | rs4411227 | 10 | *CYP26A1** | 2.37E-06 | 0.00181 | 7.526E-05 | 0.001 | RA |
|  | rs2068888* | 10 | *CYP26A1** | 1.30E-08 | 1.56E-05 | 1.682E-11 | 6.45E-06 | T1D |
| 97 | rs108499 | 11 | *C11orf9* | 4.23E-17 | 1.59E-07 | 2.045E-16 | 6.49E-09 | CD |
|  | rs509360 | 11 | *C11orf9* | 3.49E-18 | 1.59E-07 | 4.203E-17 | 1.39E-08 | CD |
|  | rs740006 | 11 | *C11orf10* | 2.99E-06 | 0.00221 | 6.968E-05 | 0.00265 | RA |
|  | rs174538 | 11 | *C11orf10* | 3.10E-20 | 1.59E-07 | 3.46E-28 | 4.50E-08 | RA |
|  | rs174556 | 11 | *FADS1** | 8.45E-24 | 1.59E-07 | 1.3E-32 | 6.49E-09 | CD |
|  | rs968567 | 11 | *FADS2** | 3.03E-07 | 0.000295 | 3.397E-09 | 2.09E-05 | RA |
|  | rs174570 | 11 | *FADS2** | 2.84E-10 | 4.80E-07 | 1.611E-18 | 3.04E-08 | CD |
|  | rs2727270 | 11 | *FADS2** | 8.80E-14 | 1.59E-07 | 2.099E-14 | 1.69E-07 | RA |
|  | rs2524299 | 11 | *FADS2** | 4.68E-13 | 1.59E-07 | 1.267E-20 | 1.39E-07 | UC |
|  | rs2072114 | 11 | *FADS2** | 1.43E-12 | 1.59E-07 | 5.254E-20 | 1.56E-08 | CD |
|  | rs174449 | 11 | *FADS3** | 1.21E-10 | 1.98E-07 | 2.463E-17 | 1.74E-08 | CD |
|  | rs7942717 | 11 | *FADS3** | 1.38E-05 | 0.00715 | 1.112E-08 | 0.00161 | CD |
|  | rs174634 | 11 | *FADS3** | 5.04E-07 | 0.00044 | 1.117E-09 | 2.83E-05 | RA |
|  | rs1000778 | 11 | *FADS3** | 6.14E-07 | 0.000538 | 3.751E-09 | 3.57E-05 | RA |
| 105 | rs516226 | 11 | *BUD13* | 8.16E-21 | 1.59E-07 | 6.221E-22 | 9.23E-08 | CD |
|  | rs3016352 | 11 | *BUD13* | 3.35E-12 | 1.59E-07 | 5.767E-10 | 9.23E-08 | CD |
|  | rs12805779 | 11 | *BUD13* | 1.64E-06 | 0.00121 | 1.635E-07 | 0.00148 | RA |
|  | rs545108 | 11 | *BUD13* | 6.30E-13 | 1.59E-07 | 1.959E-10 | 1.97E-07 | RA |
|  | rs486394 | 11 | *BUD13* | 2.73E-32 | 1.59E-07 | 2.817E-60 | 1.39E-07 | T1D |
|  | rs496958 | 11 | *BUD13* | 7.38E-21 | 1.59E-07 | 1.965E-35 | 9.93E-08 | UC |
|  | rs530053 | 11 | *BUD13* | 2.22E-12 | 1.59E-07 | 2.122E-10 | 1.12E-07 | CD |
|  | rs558056 | 11 | *BUD13* | 1.33E-11 | 1.59E-07 | 2.576E-24 | 1.12E-07 | CD |
|  | rs17119878 | 11 | *BUD13* | 1.40E-58 | 1.59E-07 | 8.487E-75 | 1.12E-07 | CD |
|  | rs2155216 | 11 | *BUD13* | 5.29E-15 | 1.59E-07 | 2.114E-34 | 9.93E-08 | UC |
|  | rs2186670 | 11 | *BUD13* | 2.05E-15 | 1.59E-07 | 1.131E-35 | 1.23E-07 | CD |
|  | rs1145187 | 11 | *BUD13* | 1.69E-16 | 1.59E-07 | 1.918E-24 | 1.38E-07 | SARC |
|  | rs1145189 | 11 | *BUD13* | 3.78E-17 | 1.59E-07 | 1.076E-25 | 1.22E-07 | T1D |
|  | rs1240776 | 11 | *BUD13* | 2.29E-16 | 1.59E-07 | 2.184E-29 | 1.93E-07 | UC |
|  | rs1145212 | 11 | *BUD13* | 6.99E-11 | 1.59E-07 | 4.061E-13 | 1.23E-07 | CD |
|  | rs1145195 | 11 | *BUD13* | 1.76E-19 | 1.59E-07 | 1.771E-41 | 1.34E-07 | CD |
|  | rs11601584 | 11 | *BUD13* | 9.96E-24 | 1.59E-07 | 1.374E-40 | 4.56E-08 | UC |
|  | rs1892948 | 11 | *BUD13* | 3.17E-70 | 1.59E-07 | 1.034E-64 | 1.46E-07 | CD |
|  | rs1145196 | 11 | *BUD13* | 5.43E-19 | 1.59E-07 | 2.541E-41 | 1.34E-07 | CD |
|  | rs10892019 | 11 | *BUD13* | 1.64E-23 | 1.59E-07 | 3.293E-29 | 1.69E-07 | RA |
|  | rs7350481 | 11 | *BUD13* | 2.78E-126 | 1.59E-07 | 3.21E-233 | 1.34E-07 | CD |
|  | rs7946299 | 11 | *BUD13* | 4.92E-09 | 6.63E-06 | 1.345E-12 | 6.19E-06 | T1D |
|  | rs180366 | 11 | *BUD13* | 3.06E-24 | 1.59E-07 | 9.074E-32 | 1.46E-07 | CD;PSOR |
|  | rs180360 | 11 | *BUD13* | 7.82E-27 | 1.59E-07 | 1.083E-48 | 9.93E-08 | UC |
|  | rs7123583 | 11 | *BUD13* | 1.47E-15 | 1.59E-07 | 2.14E-20 | 1.15E-07 | CeD |
|  | rs12272004 | 11 | *BUD13* | 8.94E-110 | 1.59E-07 | 1.56E-179 | 7.55E-08 | CeD |
|  | rs17518841 | 11 | *BUD13* | 1.08E-05 | 0.00588 | 9.826E-15 | 0.00352 | RA |
|  | rs12292921 | 11 | *BUD13* | 1.49E-111 | 1.59E-07 | 1.26E-187 | 7.55E-08 | CeD |
|  | rs180326 | 11 | *BUD13* | 1.20E-52 | 1.59E-07 | 6.27E-108 | 1.53E-07 | PSOR |
|  | rs2075295 | 11 | *BUD13* | 2.94E-11 | 1.59E-07 | 1.19E-12 | 1.34E-07 | CD |
|  | rs964184* | 11 | *ZNF259* | 7.70E-249 | 1.59E-07 | 6.55E-224 | 1.34E-07 | CD |
|  | rs6589566 | 11 | *ZNF259* | 2.10E-121 | 1.59E-07 | 1.04E-239 | 1.32E-07 | T1D |
|  | rs3741298 | 11 | *ZNF259* | 1.29E-06 | 0.000985 | 2.49E-124 | 0.000347 | RA |
|  | rs6589567 | 11 | *APOA5** | 6.78E-72 | 1.59E-07 | 6.22E-121 | 1.22E-07 | T1D |
|  | rs1263167 | 11 | *APOA4** | 5.46E-17 | 1.59E-07 | 5.593E-17 | 8.33E-08 | CD |
|  | rs4938315 | 11 | *SIK3* | 3.19E-51 | 1.59E-07 | 2.012E-49 | 4.03E-08 | UC |
|  | rs2080586 | 11 | *SIK3* | 1.34E-18 | 1.59E-07 | 2.416E-15 | 1.46E-07 | CD;PSOR |
|  | rs11216164 | 11 | *SIK3* | 2.06E-09 | 2.75E-06 | 2.124E-17 | 2.19E-06 | RA |
|  | rs548638 | 11 | *QSK* | 2.72E-12 | 1.59E-07 | 7.836E-16 | 2.08E-07 | RA |
|  | rs518547 | 11 | *QSK* | 5.13E-12 | 1.59E-07 | 8.493E-11 | 1.12E-07 | CD |
|  | rs11216169 | 11 | *SIK3* | 1.13E-18 | 1.59E-07 | 1.924E-15 | 1.46E-07 | CD |
|  | rs482371 | 11 | *SIK3* | 1.41E-32 | 1.59E-07 | 2.563E-57 | 8.70E-08 | RA |
|  | rs11216180 | 11 | *SIK3* | 4.77E-20 | 1.59E-07 | 7.237E-17 | 1.97E-07 | RA |
|  | rs7109876 | 11 | *SIK3* | 5.15E-20 | 1.59E-07 | 6.843E-17 | 1.22E-07 | T1D |
|  | rs7115242 | 11 | *SIK3* | 7.76E-47 | 1.59E-07 | 8.097E-48 | 3.57E-08 | UC |
|  | rs17581874 | 11 | *SIK3* | 4.72E-08 | 5.53E-05 | 1.818E-11 | 6.65E-05 | RA |
|  | rs10892074 | 11 | *SIK3* | 7.12E-13 | 1.59E-07 | 3.194E-11 | 6.34E-08 | RA |
|  | rs7120515 | 11 | *AB231710* | 1.96E-28 | 1.59E-07 | 1.508E-25 | 6.11E-08 | RA |
|  | rs10892078 | 11 | *AB231710* | 8.70E-13 | 1.59E-07 | 9.189E-12 | 6.11E-08 | RA |
|  | rs6589598 | 11 | *AB231710* | 9.51E-11 | 1.59E-07 | 1.736E-10 | 6.11E-08 | RA |
|  | rs10892082 | 11 | *PAFAH1B2* | 1.86E-39 | 1.59E-07 | 9.27E-40 | 4.03E-08 | UC |
|  | rs7112577 | 11 | *PAFAH1B2* | 1.98E-07 | 0.000195 | 1.826E-07 | 0.000154 | CD |
|  | rs236911 | 11 | *PCSK7* | 2.71E-24 | 1.59E-07 | 4.89E-25 | 4.56E-08 | UC |
| 111 | rs10783815 | 12 | *NXPH4* | 8.52E-05 | 0.0324 | 1.625E-05 | 0.00941 | UC |
|  | rs11613352* | 12 | *R3HDM2* | 2.09E-10 | 3.08E-07 | 9.397E-14 | 3.84E-07 | RA |
|  | rs3741414 | 12 | *INHBC* | 3.14E-10 | 4.80E-07 | 1.443E-13 | 2.58E-07 | UC |
| 115 | rs4930732 | 12 | *DNAH10* | 2.60E-06 | 0.00181 | 3.355E-05 | 0.0016 | CD |
|  | rs4930731 | 12 | *DNAH10* | 1.43E-06 | 0.00121 | 2.845E-05 | 0.000998 | CD |
|  | rs11057408 | 12 | *ZNF664** | 8.64E-09 | 1.02E-05 | 2.049E-12 | 4.50E-06 | UC |
|  | rs7978610 | 12 | *ZNF664** | 7.68E-09 | 1.02E-05 | 1.87E-08 | 3.96E-06 | CD |
|  | rs7307277 | 12 | *ZNF664** | 2.56E-08 | 2.94E-05 | 3.278E-11 | 8.58E-06 | UC |
|  | rs12310367* | 12 | *ZNF664** | 6.48E-09 | 8.23E-06 | 1.085E-07 | 1.00E-05 | RA |
| 120 | rs2412710* | 15 | *CAPN3** | 1.01E-08 | 1.26E-05 | 1.658E-11 | 6.14E-06 | CD |
| 121 | rs8035689 | 15 | *FRMD5** | 5.57E-11 | 1.59E-07 | 1.783E-08 | 1.53E-07 | RA |
|  | rs492571 | 15 | *FRMD5** | 2.62E-11 | 1.59E-07 | 6.737E-17 | 1.19E-07 | SARC |
|  | rs2918952 | 15 | *FRMD5** | 3.95E-11 | 1.59E-07 | 1.412E-08 | 1.09E-07 | SARC |
|  | rs483800 | 15 | *FRMD5** | 2.94E-11 | 1.59E-07 | 1.146E-08 | 9.93E-08 | SARC |
|  | rs8035382 | 15 | *FRMD5** | 2.69E-11 | 1.59E-07 | 6.283E-16 | 1.19E-07 | SARC |
| 123 | rs12908474 | 15 | *LIPC** | 5.71E-08 | 6.82E-05 | 2.082E-05 | 6.62E-05 | PSOR |
|  | rs4775041 | 15 | *LIPC** | 2.11E-12 | 1.59E-07 | 4.826E-16 | 8.75E-08 | UC |
|  | rs10468017 | 15 | *LIPC** | 3.01E-13 | 1.59E-07 | 7.559E-21 | 9.23E-08 | CD |
|  | rs17821274 | 15 | *LIPC** | 2.57E-06 | 0.00181 | 2.584E-07 | 0.00136 | PSOR |
|  | rs11855284 | 15 | *LIPC** | 1.04E-10 | 1.59E-07 | 1.345E-11 | 1.27E-07 | T1D |
|  | rs415799 | 15 | *LIPC** | 6.78E-08 | 6.82E-05 | 4.184E-07 | 5.33E-05 | CD |
|  | rs12708454 | 15 | *LIPC** | 3.18E-07 | 0.000295 | 4.467E-05 | 0.000235 | PSOR |
|  | rs487766 | 15 | *LIPC** | 7.35E-10 | 1.15E-06 | 1.602E-09 | 8.84E-07 | T1D |
|  | rs13329672 | 15 | *LIPC** | 2.56E-10 | 3.85E-07 | 7.505E-10 | 2.99E-07 | RA |
|  | rs17821316 | 15 | *LIPC** | 3.41E-07 | 0.000295 | 5.306E-07 | 0.000207 | RA |
| 124 | rs11635491 | 15 | *LIPC** | 1.22E-10 | 1.98E-07 | 6.282E-10 | 9.14E-08 | SARC |
|  | rs1077834 | 15 | *LIPC** | 2.09E-12 | 1.59E-07 | 4.84E-28 | 1.06E-07 | CeD |
|  | rs261332 | 15 | *LIPC** | 2.20E-12 | 1.59E-07 | 1.303E-11 | 1.52E-07 | PSOR |
|  | rs588136 | 15 | *LIPC** | 6.82E-13 | 1.59E-07 | 3.365E-30 | 1.53E-07 | PSOR |
| 130 | rs8058961 | 16 | *ZNF629* | 0.000245 | 0.0757 | 0.000649 | 0.00919 | PSOR |
|  | rs11150596 | 16 | *BCL7C* | 9.08E-06 | 0.00588 | 2.883E-05 | 0.00045 | PSOR |
|  | rs11649653* | 16 | *CTF1** | 1.85E-08 | 2.38E-05 | 1.56E-07 | 2.56E-05 | UC |
|  | rs4889603 | 16 | *SETD1A* | 8.83E-08 | 8.42E-05 | 2.56E-07 | 6.98E-06 | PSOR |
|  | rs2303222 | 16 | *ZNF668* | 8.09E-08 | 8.42E-05 | 6.581E-09 | 9.37E-06 | PSOR |
|  | rs749767 | 16 | *BCKDK* | 1.57E-07 | 0.000158 | 5.978E-09 | 1.79E-05 | PSOR |
| 131 | rs9989419 | 16 | *HERPUD1* | 9.95E-06 | 0.00588 | 1.052E-11 | 0.00484 | CD |
|  | rs12448528 | 16 | *HERPUD1* | 2.84E-06 | 0.00221 | 5.512E-10 | 0.00204 | RA |
|  | rs3764261 | 16 | *CETP** | 2.49E-12 | 1.59E-07 | 1.584E-25 | 1.25E-07 | CeD |
|  | rs1800775 | 16 | *CETP** | 1.18E-11 | 1.59E-07 | 1.332E-26 | 1.26E-07 | UC |
|  | rs1864163 | 16 | *CETP** | 4.14E-10 | 5.97E-07 | 1.753E-17 | 5.93E-07 | CeD |
|  | rs7205804* | 16 | *CETP** | 4.38E-13 | 1.59E-07 | 3.431E-25 | 1.12E-07 | UC |
|  | rs1532624 | 16 | *CETP** | 4.84E-13 | 1.59E-07 | 4.044E-25 | 1.12E-07 | UC |
| 132 | rs7499892 | 16 | *CETP** | 1.66E-07 | 0.000158 | 2.994E-16 | 0.000101 | SARC |
| 151 | rs1871047 | 19 | *PVRL2* | 1.40E-06 | 0.000985 | 6.679E-06 | 0.000445 | PSOR |
|  | rs1871046 | 19 | *PVRL2* | 1.01E-06 | 0.000805 | 0.0002033 | 0.000844 | RA |
|  | rs11667640 | 19 | *PVRL2* | 2.44E-10 | 3.85E-07 | 1.104E-15 | 2.77E-07 | CD |
|  | rs11669338 | 19 | *PVRL2* | 7.51E-15 | 1.59E-07 | 6.089E-13 | 1.97E-07 | RA |
|  | rs7254892 | 19 | *PVRL2* | 2.46E-08 | 2.94E-05 | 1.398E-24 | 2.79E-05 | PSOR |
|  | rs6857 | 19 | *PVRL2* | 7.90E-22 | 1.59E-07 | 4.548E-19 | 1.95E-07 | RA |
|  | rs157580 | 19 | *TOMM40* | 2.10E-16 | 1.59E-07 | 3.396E-21 | 1.06E-07 | T1D |
|  | rs2075650 | 19 | *TOMM40* | 2.75E-20 | 1.59E-07 | 2.308E-21 | 8.33E-08 | CD |
|  | rs439401 | 19 | *APOE** | 9.28E-32 | 1.59E-07 | 1.423E-66 | 3.68E-08 | CD |
| 162 | rs435306 | 20 | *PLTP** | 4.62E-06 | 0.00328 | 3.194E-12 | 0.00179 | UC |
|  | rs4810479* | 20 | *PLTP** | 1.12E-18 | 1.59E-07 | 2.067E-34 | 9.68E-08 | RA |
|  | rs6065906 | 20 | *PCIF1* | 6.62E-18 | 1.59E-07 | 2.363E-34 | 7.51E-08 | CD |
|  | rs3848715 | 20 | *PCIF1* | 2.55E-06 | 0.00181 | 1.36E-08 | 0.000949 | CD |
|  | rs3746503 | 20 | *ZNF335* | 9.88E-06 | 0.00588 | 0.0001471 | 0.00373 | CD |
|  | rs3746506 | 20 | *ZNF335* | 7.56E-11 | 1.59E-07 | 7.392E-10 | 8.69E-08 | T1D |
|  | rs11906879 | 20 | *ZNF335* | 2.39E-07 | 0.00024 | 6.064E-11 | 9.69E-05 | RA |
|  | rs6065912 | 20 | *MMP9* | 4.80E-07 | 0.00044 | 1.363E-11 | 0.000165 | CD |
|  | rs3918241 | 20 | *MMP9* | 1.57E-07 | 0.000158 | 1.313E-10 | 0.000101 | RA |
|  | rs2274755 | 20 | *MMP9* | 2.33E-07 | 0.00024 | 3.051E-11 | 7.94E-05 | CD |
|  | rs3918261 | 20 | *MMP9* | 1.23E-06 | 0.000985 | 1.435E-09 | 0.000352 | CD |
| 165 | rs6001027 | 22 | *PLA2G6** | 6.73E-08 | 6.82E-05 | 1.026E-09 | 4.37E-05 | SARC |
|  | rs5756931* | 22 | *PLA2G6** | 2.12E-08 | 2.38E-05 | 2.524E-08 | 2.01E-05 | SARC |
|  | rs6001031 | 22 | *PLA2G6** | 3.97E-08 | 4.48E-05 | 7.82E-10 | 3.24E-05 | SARC |

Independent complex or single gene loci (r^2^ < 0.2) with SNP(s) with a conditional FDR (condFDR) < 0.01 in triglycerides (TG) conditioned on the associated immune-mediated disease. All SNPs with a condFDR value < 0.01 are listed. For TG and immune-mediated disease phenotypes we defined the most significant SNP in each LD block based on the minimum condFDR (min FDR). For comparison, the minimal condFDR values for each identified SNP are listed for the immune-mediated disease(s) on which the FDR was conditioned (Driving phenotype, multiple phenotypes separated by semi-colon). In addition, the chromosome number (Chr), closest gene (Gene symbol), unconditional false discovery rates (FDR) and p values of each SNPs for the corresponding lipids are given. The corresponding p values from the larger sample of TG (TG2 P-value) are also listed. Independent loci which do not contain any known SNPs or genes are listed first consecutively, followed by loci containing SNPs or genes, which are marked by astrices (*).All data were first corrected for genomic inflation. Crohn’s Disease (CD), ulcerative colitis (UC), rheumatoid arthritis (RA), type 1 diabetes (T1D), celiac disease (CeD), psoriasis (PSOR) and sarcoidosis (SARC). NA; not available.

### Table C. All SNPs in low density lipoprotein (LDL) conditioned on immune-mediated diseases (condFDR<0.01).

.

| **# Locus** | **SNP** | **Chr** | **Gene**  **symbol** | **LDL**  **P-value** | **LDL**  **FDR** | **LDL2**  **P-value** | **minFDR** | **Driving**  **phenotype** |
| --- | --- | --- | --- | --- | --- | --- | --- | --- |
| 1 | rs11802413 | 1 | *TMEM57* | 8.87E-11 | 1.53E-07 | 5.279E-17 | 1.11E-07 | RA |
|  | rs10903129 | 1 | *TMEM57* | 7.12E-11 | 1.53E-07 | 3.03E-17 | 1.14E-07 | RA |
| 2 | rs12752833 | 1 | *ARID1A* | 2.09E-06 | 0.00147 | 2.593E-05 | 0.00063 | CD |
|  | rs12735646 | 1 | *ARID1A* | 1.55E-06 | 0.0012 | 4.795E-12 | 0.00113 | RA |
|  | rs12742115 | 1 | *ARID1A* | 2.24E-06 | 0.00179 | 3.455E-05 | 0.00033 | T1D |
| 3 | rs17111483 | 1 | *BSND* | 2.98E-08 | 3.56E-05 | 2.299E-06 | 3.76E-05 | SARC |
| 10 | rs2647281 | 1 | *USP24* | 1.84E-05 | 0.0106 | 2.274E-09 | 0.00777 | T1D |
| 11 | rs6662286 | 1 | *USP24* | 3.33E-15 | 1.53E-07 | 6.299E-36 | 1.38E-07 | CeD |
|  | rs12086676 | 1 | *USP24* | 1.92E-07 | 0.000191 | 6.129E-08 | 0.000171 | SARC |
|  | rs1874775 | 1 | *USP24* | 3.14E-11 | 1.53E-07 | 4.71E-10 | 1.30E-07 | UC |
|  | rs1874776 | 1 | *USP24* | 2.69E-11 | 1.53E-07 | 2.759E-21 | 1.30E-07 | UC;CeD |
|  | rs11206551 | 1 | *USP24* | 8.85E-11 | 1.53E-07 | 7.095E-21 | 1.30E-07 | UC |
|  | rs17111834 | 1 | *USP24* | 2.30E-06 | 0.00179 | 1.493E-05 | 0.00217 | RA |
|  | rs17111847 | 1 | *USP24* | 1.24E-05 | 0.00713 | 7.06E-05 | 0.00592 | CD |
|  | rs207152 | 1 | *USP24* | 1.06E-05 | 0.00585 | 8.832E-06 | 0.0046 | CD |
|  | rs6677134 | 1 | *USP24* | 1.89E-06 | 0.00147 | 1.573E-06 | 0.00129 | CD |
|  | rs17111966 | 1 | *USP24* | 5.66E-07 | 0.000529 | 8.594E-06 | 0.000509 | SARC |
|  | rs207136 | 1 | *USP24* | 1.69E-08 | 1.87E-05 | 1.863E-07 | 1.72E-05 | SARC |
|  | rs207135 | 1 | *USP24* | 9.11E-09 | 1.20E-05 | 1.016E-07 | 1.11E-05 | SARC |
| 12 | rs12065953 | 1 | *AK127270* | 3.69E-06 | 0.00268 | 0.0001075 | 0.00199 | UC |
| 13 | rs631106 | 1 | *USP1* | 6.91E-18 | 1.53E-07 | 1.582E-31 | 7.63E-09 | CD |
|  | rs10158897 | 1 | *USP1* | 6.48E-18 | 1.53E-07 | 1.008E-31 | 7.63E-09 | CD |
|  | rs1168041 | 1 | *DOCK7* | 2.62E-17 | 1.53E-07 | 4.844E-16 | 7.63E-09 | CD |
|  | rs1184865 | 1 | *DOCK7* | 1.95E-18 | 1.53E-07 | 4.757E-32 | 1.57E-07 | T1D |
|  | rs13375691 | 1 | *DOCK7* | 2.27E-06 | 0.00179 | 1.762E-08 | 0.00179 | CeD |
|  | rs1168013 | 1 | *DOCK7* | 6.17E-19 | 1.53E-07 | 5.713E-33 | 7.63E-09 | CD |
|  | rs12048208 | 1 | *DOCK7* | 6.78E-07 | 0.000529 | 4.987E-06 | 0.000184 | CD |
|  | rs2031373 | 1 | *DOCK7* | 1.65E-08 | 1.87E-05 | 2.672E-13 | 4.85E-06 | CD |
|  | rs912540 | 1 | *DOCK7* | 8.17E-11 | 1.53E-07 | 3.756E-19 | 3.10E-08 | CD |
|  | rs9787151 | 1 | *DOCK7* | 5.19E-17 | 1.53E-07 | 1.375E-28 | 7.63E-09 | CD |
| 14 | rs7512480 | 1 | *ATG4C* | 4.26E-05 | 0.0186 | 2.621E-08 | 0.00259 | CD |
| 15 | rs3122323 | 1 | *C1orf146* | 4.92E-07 | 0.000431 | 5.172E-07 | 0.000166 | CD |
|  | rs3131820 | 1 | *C1orf146* | 5.23E-07 | 0.000431 | 8.803E-07 | 0.000166 | CD |
|  | rs2025607 | 1 | *RPAP2* | 1.85E-07 | 0.000191 | 2.869E-07 | 0.000163 | CeD |
|  | rs4970712 | 1 | *EVI5* | 9.15E-08 | 0.000103 | 2.458E-13 | 5.43E-05 | CD |
|  | rs7515577 | 1 | *EVI5* | 7.55E-08 | 8.33E-05 | 1.213E-07 | 4.40E-05 | CD |
| 16 | rs1337247 | 1 | *KIAA1324* | 4.58E-12 | 1.53E-07 | 7.743E-17 | 1.60E-07 | RA |
|  | rs17641977 | 1 | *KIAA1324* | 6.06E-14 | 1.53E-07 | 2.511E-11 | 1.57E-07 | T1D |
|  | rs676385 | 1 | *KIAA1324* | 1.25E-09 | 1.68E-06 | 1.169E-15 | 1.68E-06 | CeD |
|  | rs648673 | 1 | *KIAA1324* | 3.18E-14 | 1.53E-07 | 1.155E-18 | 1.41E-07 | CD |
| 18 | rs4970729 | 1 | *PSMA5* | 6.09E-12 | 1.53E-07 | 1.164E-13 | 1.22E-07 | PSOR |
|  | rs4970766 | 1 | *SYPL2* | 5.00E-11 | 1.53E-07 | 4.72E-13 | 1.26E-07 | CD |
|  | rs10494040 | 1 | *SYPL2* | 5.62E-11 | 1.53E-07 | 3.245E-10 | 1.26E-07 | CD |
|  | rs2781553 | 1 | *ATXN7L2* | 5.29E-11 | 1.53E-07 | 2.548E-11 | 1.30E-07 | UC |
|  | rs529773 | 1 | *AMIGO1* | 6.66E-06 | 0.00397 | 1.402E-07 | 0.00103 | PSOR |
|  | rs532623 | 1 | *GPR61* | 2.55E-06 | 0.00179 | 4.848E-06 | 0.00112 | T1D |
| 19 | rs501163 | 1 | *GPR61* | 7.56E-06 | 0.00481 | 1.272E-05 | 0.00278 | CD |
| 20 | rs865774 | 1 | *AMPD2* | 4.12E-07 | 0.000351 | 2.759E-10 | 0.000331 | CD |
|  | rs17024629 | 1 | *AMPD2* | 3.30E-07 | 0.000287 | 8.589E-08 | 0.000307 | RA |
|  | rs1010167 | 1 | *GSTM4* | 6.74E-07 | 0.000529 | 6.221E-11 | 0.000665 | RA |
| 21 | rs520263 | 1 | *EPS8L3* | 7.16E-07 | 0.000651 | 7.958E-07 | 0.00077 | RA |
|  | rs500117 | 1 | *EPS8L3* | 1.72E-06 | 0.0012 | 1.8E-06 | 0.000897 | UC |
|  | rs484959 | 1 | *EPS8L3* | 2.99E-05 | 0.0155 | 9.889E-07 | 0.00595 | CD |
| 22 | rs7367104 | 1 | *CSF1* | 0.000131 | 0.0466 | 0.005767 | 0.00778 | T1D |
| 23 | rs267733 | 1 | *ANXA9* | 3.13E-08 | 3.56E-05 | 5.285E-09 | 1.34E-05 | SARC |
| 24 | rs10494359 | 1 | *FCGR2A* | 3.51E-05 | 0.0155 | 3.961E-05 | 0.00329 | RA |
|  | rs7551957 | 1 | *FCGR2A* | 1.83E-05 | 0.0106 | 1.926E-05 | 0.00198 | RA |
| 25 | rs11590932 | 1 | *FCRLB* | 1.64E-05 | 0.0087 | 0.001577 | 0.00484 | CeD |
| 26 | rs3738423 | 1 | *NPHS2* | 2.14E-06 | 0.00147 | 4.276E-05 | 0.00054 | CeD |
|  | rs12048994 | 1 | *NPHS2* | 1.81E-06 | 0.00147 | 3.764E-05 | 0.00134 | PSOR |
| 29 | rs661955 | 1 | *CR596412* | 4.17E-11 | 1.53E-07 | 1.302E-10 | 1.67E-07 | RA |
|  | rs6695664 | 1 | *CR596412* | 2.79E-08 | 2.87E-05 | 9.359E-13 | 2.74E-05 | T1D |
|  | rs10910490 | 1 | *CR596412* | 2.59E-08 | 2.87E-05 | 3.367E-13 | 2.77E-05 | CD |
|  | rs2587534 | 1 | *CR596412* | 5.19E-11 | 1.53E-07 | 8.055E-25 | 1.39E-07 | SARC |
|  | rs553427 | 1 | *CR596412* | 1.10E-10 | 1.53E-07 | 3.345E-10 | 1.39E-07 | SARC |
|  | rs558971 | 1 | *CR596412* | 5.66E-11 | 1.53E-07 | 5.555E-24 | 1.39E-07 | SARC |
| 30 | rs7255 | 2 | *C2orf43* | 1.09E-06 | 0.000799 | 5.835E-05 | 0.000659 | PSOR |
| 33 | rs1260326 | 2 | *GCKR* | 0.000164 | 0.0556 | 1.509E-07 | 0.00706 | CD |
| 34 | rs848606 | 2 | *CRIM1* | 1.33E-05 | 0.00713 | 5.306E-06 | 0.00826 | RA |
| 35 | rs10171620 | 2 | *ZFP36L2* | 2.19E-05 | 0.0106 | 0.001447 | 0.00598 | RA |
| 39 | rs17031864 | 2 | *LRPPRC* | 1.50E-05 | 0.0087 | 5.835E-07 | 0.0073 | RA |
| 40 | rs11125936 | 2 | *BC038779* | 7.57E-07 | 0.000651 | 4.069E-08 | 0.000805 | RA |
|  | rs2075375 | 2 | *OTX1* | 1.70E-07 | 0.000156 | 2.557E-07 | 9.80E-05 | RA |
|  | rs10206947 | 2 | *C2orf86* | 2.22E-05 | 0.0106 | 1.892E-05 | 0.0071 | T1D |
|  | rs2421916 | 2 | *C2orf86* | 2.03E-07 | 0.000191 | 1.407E-06 | 0.000124 | RA |
| 41 | rs826681 | 2 | *LIMS1* | 2.31E-06 | 0.00179 | 1.299E-05 | 0.0022 | RA |
|  | rs826682 | 2 | *LIMS1* | 9.02E-06 | 0.00585 | 9.746E-06 | 0.0046 | CD |
| 42 | rs10490632 | 2 | *DDX18* | 3.53E-08 | 3.56E-05 | 8.194E-08 | 3.49E-05 | CD |
|  | rs1052639 | 2 | *DDX18* | 3.16E-08 | 3.56E-05 | 8.064E-08 | 3.49E-05 | CD |
| 43 | rs2030746 | 2 | *FLJ14816* | 1.97E-07 | 0.000191 | 8.605E-09 | 0.000123 | PSOR |
|  | rs7568633 | 2 | *FLJ14816* | 9.99E-07 | 0.000799 | 6.986E-07 | 9.22E-05 | PSOR |
| 44 | rs16831243 | 2 | *YSK4* | 4.26E-07 | 0.000351 | 9.063E-12 | 0.000233 | UC |
|  | rs749873 | 2 | *CXCR4* | 3.22E-07 | 0.000287 | 9.163E-10 | 5.26E-05 | T1D |
| 45 | rs10184004 | 2 | *GRB14* | 7.06E-07 | 0.000529 | 5.805E-08 | 0.000603 | RA |
| 45 | rs10195252 | 2 | *COBLL1* | 1.08E-06 | 0.000799 | 3.812E-08 | 0.000279 | CD |
| 46 | rs2287623 | 2 | *ABCB11* | 5.06E-07 | 0.000431 | 5.399E-08 | 0.000251 | T1D |
| 47 | rs934287 | 2 | *ICA1L* | 3.74E-05 | 0.0186 | 1.214E-07 | 0.00677 | T1D |
| 48 | rs1250259 | 2 | *FN1* | 8.78E-06 | 0.00481 | 1.46E-06 | 0.00372 | RA |
| 49 | rs2741019 | 2 | *UGT1A8* | 6.49E-06 | 0.00397 | 2.269E-05 | 0.00439 | RA |
|  | rs2741021 | 2 | *UGT1A8* | 6.13E-06 | 0.00397 | 2.152E-05 | 0.00433 | UC |
|  | rs2741027 | 2 | *UGT1A8* | 7.06E-06 | 0.00397 | 1.998E-05 | 0.00302 | T1D |
| 50 | rs11563251 | 2 | *UGT1A8* | 2.05E-06 | 0.00147 | 4.499E-08 | 0.000926 | RA |
|  | rs11888492 | 2 | *UGT1A8* | 1.72E-06 | 0.0012 | 2.791E-05 | 0.000725 | RA |
|  | rs7586006 | 2 | *DQ655968* | 1.48E-05 | 0.0087 | 9.695E-06 | 0.00371 | CD |
| 51 | rs4686247 | 3 | *AK126307* | 1.03E-05 | 0.00585 | 0.01141 | 0.00309 | UC |
| 52 | rs7621919 | 3 | *VGLL4* | 1.02E-05 | 0.00585 | 3.457E-05 | 0.00636 | UC |
| 53 | rs7616006 | 3 | *NR_003112* | 1.30E-05 | 0.00713 | 2.535E-10 | 0.00631 | UC |
|  | rs13070844 | 3 | *NR_003112* | 1.15E-05 | 0.00713 | 2.152E-05 | 0.00527 | CD |
|  | rs9875338 | 3 | *NR_003112* | 4.77E-06 | 0.00327 | 2.21E-11 | 0.00255 | CD |
|  | rs4135247 | 3 | *PPARG* | 8.30E-06 | 0.00481 | 3.694E-05 | 0.00372 | RA |
| 54 | rs17819328 | 3 | *PPARG* | 1.46E-05 | 0.0087 | 6.173E-09 | 0.0034 | T1D |
| 55 | rs709162 | 3 | *TSEN2* | 5.07E-06 | 0.00327 | 1.78E-06 | 0.000297 | T1D |
|  | rs11709504 | 3 | *RAF1* | 1.09E-07 | 0.000103 | 4.601E-08 | 2.19E-05 | T1D |
|  | rs904453 | 3 | *RAF1* | 1.70E-06 | 0.0012 | 4.897E-06 | 0.00016 | T1D |
|  | rs7617041 | 3 | *RAF1* | 1.41E-07 | 0.000127 | 3.307E-07 | 5.50E-05 | T1D |
| 56 | rs7640978 | 3 | *CMTM6* | 4.97E-07 | 0.000431 | 9.837E-09 | 0.000406 | CD |
| 57 | rs6769972 | 3 | *HHLA2* | 0.000117 | 0.0466 | 0.0001858 | 0.00512 | T1D |
| 58 | rs13081171 | 3 | *CCDC58* | 5.25E-06 | 0.00327 | 0.003644 | 0.00303 | CD |
|  | rs3762637 | 3 | *KPNA1* | 1.63E-05 | 0.0087 | 4.169E-06 | 0.00483 | UC |
| 59 | rs6762156 | 3 | *KPNA1* | 1.37E-05 | 0.00713 | 0.0002492 | 0.00806 | RA |
| 60 | rs17185135 | 3 | *DNAJC13* | 6.79E-07 | 0.000529 | 2.897E-06 | 0.000657 | RA |
|  | rs10490862 | 3 | *DNAJC13* | 1.54E-06 | 0.0012 | 6.238E-06 | 0.000468 | CD |
|  | rs17404153 | 3 | *DNAJC13* | 7.70E-07 | 0.000651 | 1.832E-09 | 0.000252 | CD |
|  | rs3816529 | 3 | *DNAJC13* | 1.62E-06 | 0.0012 | 3.862E-06 | 0.000425 | CD |
| 61 | rs7652788 | 3 | *PLD1* | 7.21E-06 | 0.00481 | 0.0007357 | 0.00302 | CeD |
| 62 | rs6818397 | 4 | *RGS12* | 9.02E-06 | 0.00585 | 1.677E-08 | 0.00179 | PSOR |
|  | rs13116176 | 4 | *RGS12* | 1.86E-05 | 0.0106 | 6.585E-05 | 0.00633 | CeD |
|  | rs762861 | 4 | *RGS12* | 7.27E-06 | 0.00481 | 5.306E-06 | 0.00559 | RA |
| 63 | rs10019888 | 4 | *C4orf52* | 8.17E-05 | 0.0325 | 0.0003232 | 0.00864 | RA |
| 64 | rs10516443 | 4 | *C4orf17* | 9.08E-06 | 0.00585 | 6.101E-06 | 0.00645 | RA |
|  | rs10026790 | 4 | *RG9MTD2* | 1.50E-05 | 0.0087 | 2.555E-06 | 0.00554 | CD |
| 65 | rs6871769 | 5 | *CTNND2* | 1.86E-05 | 0.0106 | 3.703E-05 | 0.00198 | RA |
| 66 | rs870992 | 5 | *ITGA1* | 7.75E-06 | 0.00481 | 8.504E-06 | 0.00216 | T1D |
| 67 | rs4647102 | 5 | *ERCC8* | 3.16E-05 | 0.0155 | 0.05225 | 0.0078 | UC |
| 69 | rs2974610 | 5 | *TRIM36* | 5.60E-06 | 0.00327 | 0.3229 | 0.00213 | RA |
| 70 | rs6595459 | 5 | *CSNK1G3* | 2.81E-05 | 0.0128 | 2.418E-05 | 0.00987 | PSOR |
| 71 | rs9282763 | 5 | *LOC441108* | 5.60E-05 | 0.0224 | 0.001204 | 0.00244 | CD |
| 72 | rs32074 | 5 | *SGCD* | 1.75E-07 | 0.000156 | 1.398E-07 | 6.59E-05 | CD |
| 74 | rs2163455 | 6 | *TXNDC5* | 4.25E-05 | 0.0186 | 1.278E-05 | 0.00991 | T1D |
| 80 | rs11758426 | 6 | *PACSIN1* | 2.65E-07 | 0.000234 | 9.353E-06 | 0.000107 | CeD |
|  | rs7754038 | 6 | *SPDEF* | 2.72E-07 | 0.000234 | 1.014E-06 | 0.000182 | RA |
|  | rs2814982 | 6 | *C6orf106* | 2.47E-07 | 0.000234 | 3.161E-06 | 0.000174 | RA |
|  | rs3800406 | 6 | *TCP11* | 1.49E-07 | 0.000156 | 9.392E-08 | 8.94E-05 | RA |
| 81 | rs1028427 | 6 | *EFHC1* | 2.23E-06 | 0.00147 | 1.141E-05 | 0.000956 | T1D |
|  | rs2268721 | 6 | *TRAM2* | 2.02E-06 | 0.00147 | 9.63E-06 | 0.00109 | UC |
|  | rs2268719 | 6 | *TRAM2* | 2.11E-06 | 0.00147 | 9.981E-06 | 0.00126 | UC |
|  | rs2268718 | 6 | *TRAM2* | 4.98E-06 | 0.00327 | 1.843E-05 | 0.00232 | T1D |
|  | rs2268712 | 6 | *TRAM2* | 9.66E-07 | 0.000799 | 7.394E-06 | 0.000963 | RA |
| 82 | rs2239620 | 6 | *CR605995* | 3.58E-07 | 0.000351 | 5.589E-07 | 0.000192 | PSOR |
|  | rs6901147 | 6 | *CR605995* | 4.43E-06 | 0.00268 | 7.723E-07 | 0.000709 | CeD |
|  | rs10456661 | 6 | *CR605995* | 1.73E-06 | 0.0012 | 2.932E-05 | 0.000682 | RA |
|  | rs10948714 | 6 | *LOC730101* | 1.18E-06 | 0.000979 | 6.211E-07 | 0.00042 | CD |
|  | rs9370138 | 6 | *LOC730101* | 7.15E-06 | 0.00481 | 9.057E-06 | 0.00233 | CD |
|  | rs10456157 | 6 | *TMEM14A* | 1.21E-05 | 0.00713 | 7.947E-06 | 0.00826 | RA |
|  | rs2670134 | 6 | *TMEM14A* | 1.70E-05 | 0.0087 | 8.537E-06 | 0.00554 | CD |
|  | rs2207953 | 6 | *TMEM14A* | 1.66E-05 | 0.0087 | 1.043E-05 | 0.00599 | CD |
| 84 | rs9456496 | 6 | *IGF2R* | 1.63E-05 | 0.0087 | 1.054E-07 | 0.00284 | CeD |
| 85 | rs3798180 | 6 | *IGF2R* | 3.56E-08 | 4.41E-05 | 2.773E-12 | 3.71E-05 | RA |
|  | rs3798178 | 6 | *IGF2R* | 2.55E-07 | 0.000234 | 1.436E-11 | 7.97E-05 | CD |
|  | rs3798176 | 6 | *IGF2R* | 2.24E-07 | 0.000234 | 1.314E-07 | 7.12E-05 | CD |
| 86 | rs456598 | 6 | *SLC22A1* | 4.14E-15 | 1.53E-07 | 2,00E-16 | 1.11E-07 | RA |
|  | rs3798167 | 6 | *SLC22A1* | 5.46E-08 | 5.45E-05 | 3.799E-12 | 2.01E-05 | CD |
|  | rs622342 | 6 | *SLC22A1* | 9.60E-08 | 0.000103 | 6.169E-14 | 9.83E-05 | CD |
|  | rs650284 | 6 | *SLC22A1* | 2.96E-08 | 3.56E-05 | 1.487E-14 | 3.43E-05 | CD |
| 88 | rs6935921 | 6 | *PLG* | 1.89E-07 | 0.000191 | 3.418E-11 | 0.000139 | PSOR |
| 89 | rs663440 | 6 | *MAP3K4* | 5.26E-06 | 0.00327 | 0.0002427 | 0.00362 | RA |
| 92 | rs976681 | 7 | *MPP6* | 2.90E-05 | 0.0155 | 0.0001911 | 0.00915 | CD |
|  | rs10250643 | 7 | *MPP6* | 4.12E-05 | 0.0186 | 0.0002438 | 0.00826 | T1D |
|  | rs4722551 | 7 | *DM004234* | 5.49E-07 | 0.000431 | 3.949E-14 | 0.000309 | SARC |
| 95 | rs10953259 | 7 | *BAIAP2L1* | 4.33E-05 | 0.0186 | 1.801E-05 | 0.00784 | CeD |
| 96 | rs2911971 | 8 | *AGPAT5* | 1.46E-05 | 0.0087 | 0.0001734 | 0.00918 | RA |
| 97 | rs1961456 | 8 | *NAT2* | 0.000156 | 0.0556 | 6.883E-08 | 0.00758 | RA |
| 98 | rs9298506 | 8 | *SOX17* | 6.03E-07 | 0.000529 | 4.346E-10 | 0.000453 | SARC |
|  | rs10104997 | 8 | *SOX17* | 2.07E-07 | 0.000191 | 8.738E-11 | 0.000163 | SARC |
| 100 | rs2737250 | 8 | *TRPS1* | 2.37E-07 | 0.000234 | 1.902E-07 | 0.000192 | RA |
|  | rs2737252 | 8 | *TRPS1* | 1.88E-07 | 0.000191 | 7.039E-14 | 0.00019 | RA |
| 104 | rs3780181 | 9 | *VLDLR* | 4.85E-06 | 0.00327 | 1.764E-09 | 0.00226 | SARC |
| 105 | rs10810593 | 9 | *BNC2* | 5.45E-06 | 0.00327 | 5.278E-05 | 0.00202 | UC |
|  | rs2297174 | 9 | *BNC2* | 3.97E-06 | 0.00268 | 0.0002869 | 0.00308 | RA |
|  | rs263636 | 9 | *CNTLN* | 1.27E-05 | 0.00713 | 1.798E-05 | 0.00335 | RA |
| 106 | rs10757055 | 9 | *ACER2* | 2.38E-05 | 0.0128 | 8.539E-05 | 0.00146 | T1D |
|  | rs10757056 | 9 | *ACER2* | 9.99E-06 | 0.00585 | 2.139E-06 | 0.00456 | RA |
| 107 | rs1571790 | 9 | *PCSK5* | 7.50E-06 | 0.00481 | 0.0005398 | 0.00432 | CD |
| 108 | rs1929841 | 9 | *ABCA1* | 2.22E-05 | 0.0106 | 0.001293 | 0.00633 | CeD |
|  | rs2000069 | 9 | *ABCA1* | 1.83E-05 | 0.0106 | 0.0002005 | 0.00242 | T1D |
|  | rs4149273 | 9 | *ABCA1* | 1.88E-06 | 0.00147 | 4.726E-05 | 0.00123 | RA |
|  | rs12686004 | 9 | *ABCA1* | 7.91E-07 | 0.000651 | 2.247E-09 | 0.000555 | T1D |
|  | rs1883025 | 9 | *ABCA1* | 5.50E-08 | 5.45E-05 | 6.141E-11 | 4.82E-05 | RA |
|  | rs2777795 | 9 | *ABCA1* | 8.60E-07 | 0.000651 | 4.816E-10 | 0.000579 | SARC |
| 109 | rs6597604 | 9 | *RALGDS* | 5.39E-07 | 0.000431 | 1.385E-10 | 0.000326 | T1D |
|  | rs11795315 | 9 | *OBP2B* | 5.66E-08 | 6.74E-05 | 1.442E-11 | 7.91E-05 | RA |
| 111 | rs3758348 | 9 | *SURF4* | 4.56E-13 | 1.53E-07 | 1.673E-20 | 1.67E-07 | RA |
|  | rs4962153 | 9 | *vWF-CP* | 1.02E-05 | 0.00585 | 2.319E-13 | 0.000811 | T1D |
|  | rs739468 | 9 | *C9orf7* | 3.28E-06 | 0.00219 | 6.767E-14 | 0.00161 | SARC |
| 112 | rs2275774 | 10 | *C10orf18* | 8.58E-05 | 0.0325 | 7.773E-05 | 0.00886 | PSOR |
| 113 | rs12771080 | 10 | *AK056518* | 5.92E-06 | 0.00397 | 4.559E-05 | 0.0033 | T1D |
| 114 | rs11187157 | 10 | *HHEX* | 5.19E-05 | 0.0224 | 1.4E-05 | 0.00584 | UC |
| 116 | rs10128711 | 11 | *SPTY2D1* | 1.48E-07 | 0.000156 | 9.205E-13 | 0.000136 | T1D |
|  | rs4757676 | 11 | *SPTY2D1* | 3.41E-07 | 0.000287 | 3.374E-06 | 0.00017 | CD |
|  | rs10832956 | 11 | *SPTY2D1* | 4.38E-07 | 0.000351 | 4.396E-06 | 0.000169 | CD |
| 117 | rs11031570 | 11 | *RCN1* | 5.80E-05 | 0.027 | 0.002476 | 0.00715 | CD |
| 119 | rs567956 | 11 | *FLJ00225* | 0.00016 | 0.0556 | 0.1245 | 0.00706 | CD |
| 120 | rs2912 | 11 | *GAB2* | 6.01E-06 | 0.00397 | 0.00035 | 0.00256 | UC |
|  | rs1385600 | 11 | *GAB2* | 1.38E-05 | 0.00713 | 9.472E-05 | 0.00428 | UC |
|  | rs4945261 | 11 | *GAB2* | 1.65E-05 | 0.0087 | 0.001147 | 0.00483 | UC |
| 122 | rs10790519 | 11 | *UBASH3B* | 2.32E-06 | 0.00179 | 1.057E-05 | 0.000836 | T1D |
|  | rs11602361 | 11 | *UBASH3B* | 1.50E-06 | 0.0012 | 9.477E-06 | 0.000956 | CD |
| 123 | rs534278 | 11 | *RPUSD4* | 4.39E-07 | 0.000351 | 2.278E-06 | 0.000269 | UC |
|  | rs588361 | 11 | *FAM118B* | 3.26E-05 | 0.0155 | 5.24E-07 | 0.00339 | CeD |
|  | rs10893493 | 11 | *TIRAP* | 2.14E-06 | 0.00147 | 3.174E-09 | 0.000433 | CeD |
|  | rs8177399 | 11 | *TIRAP* | 8.75E-06 | 0.00481 | 1.463E-05 | 0.00432 | CD |
|  | rs8177375 | 11 | *TIRAP* | 1.85E-06 | 0.00147 | 3.437E-06 | 0.000467 | CeD |
|  | rs8177388 | 11 | *TIRAP* | 2.24E-06 | 0.00147 | 4.731E-09 | 0.000505 | PSOR |
|  | rs652534 | 11 | *DCPS* | 3.42E-06 | 0.00219 | 1.316E-06 | 0.00264 | RA |
|  | rs582037 | 11 | *CR602684* | 9.98E-06 | 0.00585 | 1.504E-09 | 0.00312 | T1D |
| 126 | rs3847680 | 12 | *OVCH1* | 3.68E-05 | 0.0186 | 0.0003013 | 0.00991 | T1D |
| 128 | rs2686555 | 12 | *CABP1* | 3.06E-06 | 0.00219 | 9.09E-05 | 0.00109 | T1D |
|  | rs2673617 | 12 | *CABP1* | 2.71E-06 | 0.00179 | 2.874E-05 | 0.00139 | RA |
| 129 | rs11065299 | 12 | *SPPL3* | 6.67E-06 | 0.00397 | 0.002391 | 0.0033 | T1D |
| 131 | rs11065406 | 12 | *OASL* | 6.07E-06 | 0.00397 | 1.173E-05 | 0.00235 | SARC |
| 132 | rs9590896 | 13 | *ZAR1L* | 2.32E-05 | 0.0128 | 0.0006636 | 0.00415 | PSOR |
|  | rs4942486 | 13 | *BRCA2* | 3.65E-08 | 4.41E-05 | 2.261E-11 | 2.65E-05 | T1D |
| 133 | rs10492397 | 13 | *N4BP2L1* | 2.66E-06 | 0.00179 | 1.997E-05 | 0.00146 | CeD |
| 134 | rs17053405 | 13 | *DCLK1* | 1.64E-05 | 0.0087 | 0.0001713 | 0.00783 | CD |
| 135 | rs17829029 | 13 | *SCA8* | 7.67E-06 | 0.00481 | 3.536E-05 | 0.00547 | RA |
|  | rs17086822 | 13 | *SCA8* | 9.67E-06 | 0.00585 | 0.02348 | 0.00339 | T1D |
| 136 | rs9600212 | 13 | *KLF12* | 1.92E-06 | 0.00147 | 0.7161 | 0.00155 | UC |
| 139 | rs2125598 | 14 | *C14orf177* | 0.000214 | 0.066 | 0.004345 | 0.00863 | T1D |
| 140 | rs12897338 | 14 | *WDR25* | 6.93E-06 | 0.00397 | 0.005652 | 0.0033 | T1D |
| 141 | rs8034505 | 15 | *KLF13* | 3.86E-05 | 0.0186 | 0.01708 | 0.00499 | CeD |
| 142 | rs3812945 | 15 | *SCAMP5* | 1.86E-05 | 0.0106 | 1.517E-05 | 0.0015 | CeD |
| 146 | rs11650232 | 17 | *DLG4* | 6.04E-06 | 0.00397 | 3.192E-08 | 0.0019 | CD |
| 146 | rs314253 | 17 | *DLG4* | 2.87E-07 | 0.000287 | 3.436E-10 | 0.000264 | CD |
| 147 | rs7503353 | 17 | *aik2* | 7.46E-07 | 0.000651 | 2.193E-06 | 0.000556 | CD |
|  | rs4791641 | 17 | *PFAS* | 1.33E-07 | 0.000127 | 1.314E-07 | 0.000118 | CD |
| 148 | rs9895443 | 17 | *ERAL1* | 3.51E-05 | 0.0155 | 3.214E-05 | 0.0096 | T1D |
| 150 | rs1801689 | 17 | *APOH* | 1.32E-05 | 0.00713 | 9.809E-12 | 0.008 | UC |
| 151 | rs2886232 | 17 | *ABCA10* | 7.24E-06 | 0.00481 | 3.876E-11 | 0.00456 | UC |
|  | rs4968845 | 17 | *ABCA10* | 4.06E-06 | 0.00268 | 7.798E-07 | 0.00298 | RA |
| 152 | rs9893349 | 17 | *MAP2K6* | 1.19E-05 | 0.00713 | 2.02E-06 | 0.00616 | SARC |
| 153 | rs7209235 | 17 | *GALK1* | 2.65E-05 | 0.0128 | 6.44E-05 | 0.00643 | CD |
|  | rs2125345 | 17 | *UNK* | 2.78E-05 | 0.0128 | 3.318E-07 | 0.00468 | CeD |
| 154 | rs9894524 | 17 | *FLJ45079* | 8.29E-08 | 8.33E-05 | 0.5191 | 8.39E-05 | PSOR |
| 155 | rs4530255 | 18 | *DSEL* | 1.40E-05 | 0.00713 | 0.0002334 | 0.00844 | RA |
| 156 | rs17342920 | 18 | *ZNF516* | 2.77E-05 | 0.0128 | 0.001332 | 0.00506 | CeD |
| 157 | rs2304165 | 19 | *ATG4D* | 1.97E-06 | 0.00147 | 4.592E-06 | 0.000716 | RA |
|  | rs17677316 | 19 | *CDKN2D* | 2.59E-06 | 0.00179 | 2.082E-09 | 0.00122 | CD |
|  | rs10403668 | 19 | *AP1M2* | 6.23E-07 | 0.000529 | 3.099E-15 | 0.000237 | RA |
|  | rs7253253 | 19 | *SLC44A2* | 6.14E-06 | 0.00397 | 0.0002965 | 0.00034 | CD |
|  | rs8106664 | 19 | *SLC44A2* | 3.88E-13 | 1.53E-07 | 1.501E-27 | 7.61E-08 | PSOR |
|  | rs3087969 | 19 | *SLC44A2* | 6.52E-13 | 1.53E-07 | 4.152E-12 | 1.22E-07 | PSOR |
|  | rs13465 | 19 | *ILF3* | 4.72E-15 | 1.53E-07 | 3.968E-30 | 8.73E-08 | PSOR |
|  | rs12974306 | 19 | *DNM2* | 9.60E-05 | 0.039 | 3.38E-08 | 0.00372 | PSOR |
|  | rs2278444 | 19 | *DNM2* | 2.69E-19 | 1.53E-07 | 1.755E-34 | 3.17E-08 | PSOR |
|  | rs11881315 | 19 | *DNM2* | 6.06E-29 | 1.53E-07 | 7.753E-50 | 3.17E-08 | PSOR |
|  | rs17265047 | 19 | *DNM2* | 6.99E-19 | 1.53E-07 | 8.104E-18 | 8.81E-09 | PSOR |
|  | rs11878377 | 19 | *C19orf38* | 7.63E-19 | 1.53E-07 | 4.128E-19 | 8.81E-09 | PSOR |
|  | rs4804546 | 19 | *CARM1* | 9.30E-14 | 1.53E-07 | 1.145E-13 | 1.40E-07 | RA |
|  | rs1529711 | 19 | *CARM1* | 8.28E-07 | 0.000651 | 2.524E-10 | 0.000522 | SARC |
|  | rs2053065 | 19 | *CARM1* | 4.66E-20 | 1.53E-07 | 4.104E-20 | 8.81E-09 | PSOR |
|  | rs11879293 | 19 | *SMARCA4* | 1.56E-17 | 1.53E-07 | 2.173E-17 | 8.81E-09 | PSOR |
|  | rs12610607 | 19 | *SMARCA4* | 1.81E-23 | 1.53E-07 | 2.25E-23 | 8.81E-09 | PSOR |
|  | rs11669133 | 19 | *SMARCA4* | 5.50E-07 | 0.000431 | 4.797E-08 | 0.000251 | T1D |
|  | rs12983316 | 19 | *SMARCA4* | 2.69E-19 | 1.53E-07 | 7.444E-22 | 8.81E-09 | PSOR |
|  | rs12232780 | 19 | *SMARCA4* | 3.61E-25 | 1.53E-07 | 1.381E-24 | 8.81E-09 | PSOR |
|  | rs4804561 | 19 | *SMARCA4* | 5.03E-24 | 1.53E-07 | 6.504E-24 | 8.81E-09 | PSOR |
|  | rs3786721 | 19 | *SMARCA4* | 2.42E-20 | 1.53E-07 | 2.89E-31 | 8.81E-09 | PSOR |
|  | rs12052058 | 19 | *SMARCA4* | 9.18E-35 | 1.53E-07 | 9.664E-62 | 6.58E-08 | PSOR |
|  | rs1529729 | 19 | *SMARCA4* | 1.20E-26 | 1.53E-07 | 1.695E-40 | 4.21E-08 | PSOR |
|  | rs8102273 | 19 | *SMARCA4* | 1.63E-24 | 1.53E-07 | 4.119E-43 | 2.43E-08 | PSOR |
| 160 | rs379309 | 19 | *KANK2* | 9.07E-06 | 0.00585 | 1.391E-13 | 0.00211 | CeD |
| 161 | rs4804160 | 19 | *DOCK6* | 5.63E-08 | 6.74E-05 | 4.926E-07 | 5.39E-05 | RA |
|  | rs376642 | 19 | *TSPAN16* | 2.70E-07 | 0.000234 | 4.666E-10 | 0.000192 | RA |
| 162 | rs7248896 | 19 | *TSPAN16* | 1.33E-05 | 0.00713 | 1.236E-05 | 0.00673 | CeD |
| 163 | rs3890384 | 19 | *MEF2B* | 3.41E-08 | 3.56E-05 | 6.767E-07 | 2.69E-05 | T1D |
|  | rs3819578 | 19 | *LOC729991-MEF2B* | 1.86E-08 | 2.32E-05 | 4.386E-07 | 1.43E-05 | CD |
|  | rs2228603 | 19 | *NCAN* | 1.89E-20 | 1.53E-07 | 4.433E-44 | 1.48E-07 | T1D |
|  | rs2238675 | 19 | *NCAN* | 2.41E-12 | 1.53E-07 | 3.926E-27 | 1.46E-07 | PSOR |
|  | rs10415849 | 19 | *GATAD2A* | 3.73E-16 | 1.53E-07 | 4.245E-36 | 1.50E-07 | T1D |
|  | rs2965185 | 19 | *GATAD2A* | 1.41E-06 | 0.000979 | 5.804E-18 | 0.000212 | RA |
|  | rs873870 | 19 | *LPAR2* | 2.02E-05 | 0.0106 | 7.625E-06 | 0.00456 | CeD |
|  | rs2304130 | 19 | *ZNF101* | 5.84E-14 | 1.53E-07 | 2.239E-32 | 1.35E-07 | CeD |
|  | rs17699261 | 19 | *ZNF14* | 2.42E-06 | 0.00179 | 1.442E-13 | 0.00171 | SARC |
| 164 | rs1594895 | 19 | *ZFP112* | 1.07E-07 | 0.000103 | 4.422E-12 | 9.12E-05 | T1D;PSOR |
| 165 | rs2571167 | 19 | *ZNF229* | 9.11E-10 | 1.35E-06 | 6.488E-09 | 1.29E-06 | UC |
|  | rs204541 | 19 | *ZNF229* | 4.85E-06 | 0.00327 | 1.592E-10 | 0.00242 | UC |
|  | rs7250401 | 19 | *CEACAM20* | 4.96E-11 | 1.53E-07 | 3.088E-10 | 1.53E-07 | RA |
|  | rs7250536 | 19 | *CEACAM20* | 6.71E-11 | 1.53E-07 | 3.577E-10 | 1.44E-07 | SARC |
|  | rs17800760 | 19 | *CEACAM20* | 5.67E-12 | 1.53E-07 | 8.873E-22 | 1.44E-07 | SARC |
|  | rs17800789 | 19 | *CEACAM20* | 1.18E-11 | 1.53E-07 | 1.773E-21 | 1.44E-07 | SARC |
|  | rs17800819 | 19 | *CEACAM20* | 1.43E-11 | 1.53E-07 | 6.544E-21 | 1.38E-07 | SARC |
|  | rs7260180 | 19 | *CEACAM20* | 1.88E-05 | 0.0106 | 2.911E-05 | 0.00777 | T1D |
| 166 | rs12977255 | 19 | *LOC147710* | 2.25E-15 | 1.53E-07 | 1.4E-15 | 1.74E-07 | RA |
|  | rs13344893 | 19 | *LOC147710* | 2.97E-15 | 1.53E-07 | 5.247E-27 | 1.48E-07 | T1D |
|  | rs7255066 | 19 | *PVR* | 1.81E-13 | 1.53E-07 | 1.044E-12 | 1.35E-07 | CeD |
|  | rs1058402 | 19 | *PVR* | 1.38E-23 | 1.53E-07 | 8.318E-22 | 1.41E-07 | SARC |
|  | rs10422182 | 19 | *PVR* | 7.50E-24 | 1.53E-07 | 5.052E-22 | 1.28E-07 | CeD |
|  | rs6509170 | 19 | *PVR* | 2.56E-24 | 1.53E-07 | 1.541E-22 | 1.41E-07 | SARC |
| 167 | rs754340 | 19 | *PVR* | 1.41E-17 | 1.53E-07 | 2.218E-16 | 1.47E-07 | CeD |
|  | rs714948 | 19 | *PVR* | 5.67E-07 | 0.000529 | 2.543E-14 | 0.000222 | RA |
|  | rs2965157 | 19 | *CEACAM19* | 6.89E-31 | 1.53E-07 | 7.292E-62 | 1.29E-07 | RA |
| 168 | rs2965156 | 19 | *CEACAM19* | 3.67E-14 | 1.53E-07 | 6.017E-14 | 1.81E-07 | RA |
|  | rs2965155 | 19 | *CEACAM19* | 6.77E-06 | 0.00397 | 5.479E-06 | 0.00279 | UC |
|  | rs10422616 | 19 | *CEACAM19* | 2.65E-06 | 0.00179 | 4.722E-13 | 0.00164 | UC |
|  | rs16979372 | 19 | *CEACAM16* | 3.63E-25 | 1.53E-07 | 1.402E-47 | 1.53E-07 | RA |
|  | rs12150984 | 19 | *CEACAM16* | 1.83E-09 | 2.60E-06 | 1.281E-12 | 2.81E-06 | PSOR |
| 169 | rs9973286 | 19 | *CEACAM16* | 2.42E-11 | 1.53E-07 | 1.118E-10 | 6.58E-08 | PSOR |
|  | rs9973305 | 19 | *CEACAM16* | 2.37E-10 | 3.62E-07 | 1.008E-09 | 1.04E-07 | PSOR |
| 170 | rs2965109 | 19 | *CEACAM16* | 1.55E-27 | 1.53E-07 | 6.647E-45 | 7.23E-08 | CD |
|  | rs7254776 | 19 | *CEACAM16* | 2.23E-26 | 1.53E-07 | 6.246E-24 | 7.23E-08 | CD |
|  | rs1004165 | 19 | *CEACAM16* | 4.70E-22 | 1.53E-07 | 3.43E-20 | 1.25E-07 | CeD |
|  | rs2965101 | 19 | *BCL3* | 5.68E-36 | 1.53E-07 | 1.069E-60 | 4.12E-08 | CD |
|  | rs1531517 | 19 | *BCL3* | 7.88E-104 | 1.53E-07 | 9.51E-163 | 3.17E-08 | PSOR |
|  | rs2965174 | 19 | *BCL3* | 6.88E-06 | 0.00397 | 5.205E-11 | 0.00238 | RA |
|  | rs4803750 | 19 | *BCL3* | 6.15E-99 | 1.53E-07 | 1.7E-162 | 2.13E-08 | PSOR |
| 171 | rs8103315 | 19 | *BCL3* | 2.44E-09 | 3.22E-06 | 1.623E-22 | 2.63E-06 | RA |
| 172 | rs3208856 | 19 | *CBLC* | 1.51E-60 | 1.53E-07 | 4.032E-56 | 1.11E-07 | PSOR |
|  | rs10419669 | 19 | *CBLC* | 4.93E-09 | 6.21E-06 | 2.355E-09 | 5.94E-06 | CD |
| 174 | rs7259004 | 19 | *EF553526* | 3.07E-43 | 1.53E-07 | 1.37E-110 | 1.74E-07 | RA |
| 175 | rs5158 | 19 | *APOC4* | 9.48E-11 | 1.53E-07 | 2.739E-10 | 1.23E-07 | RA |
|  | rs12721109 | 19 | *APOC4* | 3.88E-76 | 1.53E-07 | 2.99E-122 | 1.90E-07 | RA |
|  | rs760114 | 19 | *CLPTM1* | 2.21E-08 | 2.32E-05 | 4.913E-08 | 1.49E-05 | RA |
|  | rs875255 | 19 | *CLPTM1* | 4.76E-05 | 0.0224 | 0.0001948 | 0.00984 | RA |
|  | rs9193 | 19 | *CLPTM1* | 1.54E-07 | 0.000156 | 3.117E-07 | 5.20E-05 | CD |
|  | rs4803789 | 19 | *RELB* | 1.41E-07 | 0.000127 | 8.905E-08 | 7.11E-05 | RA |
| 176 | rs4802246 | 19 | *SFRS16* | 5.54E-06 | 0.00327 | 4.075E-05 | 0.00169 | CD |
| 177 | rs3745157 | 19 | *GEMIN7* | 2.14E-16 | 1.53E-07 | 1.923E-20 | 1.14E-07 | RA |
|  | rs7251736 | 19 | *LRRC68* | 1.06E-15 | 1.53E-07 | 1.7E-15 | 1.11E-07 | PSOR |
|  | rs11672002 | 19 | *LRRC68* | 1.33E-15 | 1.53E-07 | 1.296E-15 | 1.11E-07 | RA;PSOR |
|  | rs1048699 | 19 | *LRRC68* | 5.67E-07 | 0.000529 | 1.811E-08 | 0.000333 | T1D |
| 179 | rs732841 | 19 | *QPCTL* | 4.25E-07 | 0.000351 | 1.961E-10 | 0.000235 | UC |
| 180 | rs679574 | 19 | *FUT2* | 4.24E-08 | 4.41E-05 | 6.137E-08 | 5.54E-05 | RA |
|  | rs492602 | 19 | *FUT2* | 3.66E-08 | 4.41E-05 | 9.422E-14 | 3.03E-06 | CD |
|  | rs676388 | 19 | *FUT2* | 2.34E-07 | 0.000234 | 1.31E-11 | 1.74E-05 | CD |
| 181 | rs364585 | 20 | *SPTLC3* | 2.48E-06 | 0.00179 | 4.278E-10 | 0.000828 | RA |
|  | rs168622 | 20 | *SPTLC3* | 5.23E-06 | 0.00327 | 1.347E-05 | 0.00144 | RA |
|  | rs680379 | 20 | *SPTLC3* | 6.30E-06 | 0.00397 | 7.963E-10 | 0.0018 | RA |
| 182 | rs6111702 | 20 | *SNX5* | 1.00E-05 | 0.00585 | 1.981E-05 | 0.00663 | RA |
|  | rs4814671 | 20 | *SNX5* | 1.47E-05 | 0.0087 | 0.00018 | 0.00633 | UC |
|  | rs2745865 | 20 | *SNX5* | 8.48E-06 | 0.00481 | 3.192E-08 | 0.00448 | RA |
| 183 | rs6136213 | 20 | *SNX5* | 6.48E-06 | 0.00397 | 3.33E-07 | 0.00181 | T1D;CeD |
| 184 | rs11167186 | 20 | *EFCAB8* | 1.74E-05 | 0.0087 | 3.154E-05 | 0.00809 | CD;UC |
|  | rs12480689 | 20 | *EFCAB8* | 1.15E-06 | 0.000979 | 1.233E-05 | 0.000988 | RA |
| 185 | rs2104417 | 20 | *ERGIC3* | 3.83E-06 | 0.00268 | 1.482E-06 | 0.000951 | UC |
|  | rs2277862 | 20 | *FER1L4* | 3.85E-06 | 0.00268 | 1.301E-06 | 0.000687 | UC |
| 192 | rs6129900 | 20 | *CHD6* | 5.92E-06 | 0.00397 | 6.646E-06 | 0.00463 | RA |
|  | rs2866705 | 20 | *CHD6* | 1.62E-05 | 0.0087 | 0.0001117 | 0.00745 | CeD |
| 193 | rs1800961 | 20 | *HNF4A* | 1.50E-05 | 0.0087 | 6.034E-10 | 0.0051 | UC |
| 194 | rs4823057 | 22 | *ASCC2* | 7.11E-05 | 0.0325 | 2.033E-08 | 0.00297 | T1D |
| 195 | rs41342646 | 22 | *AK128136* | 2.75E-06 | 0.00179 | 4.842E-06 | 0.00161 | T1D |
| 4 | rs2479394 | 1 | *BSND* | 3.32E-10 | 4.50E-07 | 1.584E-19 | 3.86E-07 | CeD |
|  | rs11588151 | 1 | *BSND* | 3.95E-14 | 1.53E-07 | 8.919E-14 | 1.34E-07 | RA |
|  | rs2479393 | 1 | *BSND* | 3.51E-13 | 1.53E-07 | 2.031E-13 | 1.44E-07 | SARC |
|  | rs11206510 | 1 | *PCSK9** | 6.63E-21 | 1.53E-07 | 2.38E-53 | 1.31E-07 | PSOR |
| 5 | rs2479415 | 1 | *PCSK9** | 5.48E-14 | 1.53E-07 | 7.552E-14 | 1.40E-07 | RA |
|  | rs7523242 | 1 | *PCSK9** | 6.69E-27 | 1.53E-07 | 9.647E-41 | 1.14E-07 | RA |
|  | rs2182833 | 1 | *PCSK9** | 8.75E-25 | 1.53E-07 | 2.251E-37 | 1.11E-07 | RA |
| 6 | rs4927193 | 1 | *PCSK9** | 1.31E-12 | 1.53E-07 | 4.269E-11 | 2.13E-08 | PSOR |
|  | rs10888897 | 1 | *PCSK9** | 1.58E-11 | 1.53E-07 | 8.425E-31 | 4.21E-08 | PSOR |
|  | rs11206514 | 1 | *PCSK9** | 9.40E-14 | 1.53E-07 | 9.949E-33 | 4.21E-08 | PSOR |
| 7 | rs572512 | 1 | *PCSK9** | 6.78E-20 | 1.53E-07 | 5.309E-26 | 1.14E-07 | RA |
| 8 | rs7552841 | 1 | *PCSK9** | 1.52E-05 | 0.0087 | 5.396E-15 | 0.00673 | SARC |
| 9 | rs557435 | 1 | *PCSK9** | 1.95E-16 | 1.53E-07 | 6.273E-20 | 1.21E-07 | CeD |
|  | rs584626 | 1 | *PCSK9** | 5.85E-15 | 1.53E-07 | 6.84E-35 | 1.26E-07 | CeD |
|  | rs585131 | 1 | *PCSK9** | 3.17E-14 | 1.53E-07 | 2.699E-35 | 1.35E-07 | UC |
|  | rs505151 | 1 | *PCSK9** | 7.17E-13 | 1.53E-07 | 4.214E-17 | 1.44E-07 | SARC |
|  | rs10493176 | 1 | *USP24* | 1.13E-13 | 1.53E-07 | 2.535E-14 | 1.11E-07 | RA |
|  | rs11583974 | 1 | *USP24* | 7.57E-07 | 0.000651 | 3.951E-09 | 0.000144 | PSOR |
|  | rs1165285 | 1 | *USP24* | 7.64E-14 | 1.53E-07 | 2.209E-14 | 1.40E-07 | RA |
|  | rs17111652 | 1 | *USP24* | 1.08E-12 | 1.53E-07 | 2.263E-11 | 1.28E-07 | CeD |
|  | rs6676563 | 1 | *USP24* | 1.27E-12 | 1.53E-07 | 2.216E-13 | 1.32E-07 | UC |
|  | rs4926670 | 1 | *USP24* | 1.53E-18 | 1.53E-07 | 4.116E-39 | 1.10E-07 | RA |
|  | rs2047422 | 1 | *USP24* | 1.09E-14 | 1.53E-07 | 2.541E-15 | 1.30E-07 | UC |
|  | rs4927207 | 1 | *USP24* | 1.00E-20 | 1.53E-07 | 2.357E-39 | 9.34E-08 | CD |
|  | rs4927208 | 1 | *USP24* | 8.99E-16 | 1.53E-07 | 1.448E-14 | 1.49E-07 | SARC |
|  | rs11206538 | 1 | *USP24* | 5.93E-14 | 1.53E-07 | 1.263E-14 | 1.30E-07 | UC |
|  | rs2647286 | 1 | *USP24* | 4.84E-15 | 1.53E-07 | 6.293E-16 | 1.29E-07 | RA |
|  | rs2802865 | 1 | *USP24* | 6.04E-06 | 0.00397 | 4.797E-05 | 0.00221 | UC |
|  | rs7551981 | 1 | *USP24* | 2.40E-18 | 1.53E-07 | 1.362E-33 | 1.29E-07 | UC |
| 17 | rs3120625 | 1 | *SARS* | 2.15E-05 | 0.0106 | 2.307E-06 | 0.00424 | CD |
|  | rs683182 | 1 | *SARS* | 4.12E-08 | 4.41E-05 | 3.011E-14 | 1.96E-05 | T1D |
|  | rs4268379 | 1 | *SARS* | 8.74E-20 | 1.53E-07 | 1.396E-29 | 1.16E-07 | CD |
|  | rs611060 | 1 | *SARS* | 5.40E-24 | 1.53E-07 | 4.274E-42 | 1.34E-07 | RA |
|  | rs11102964 | 1 | *SARS* | 8.79E-22 | 1.53E-07 | 7.955E-30 | 9.91E-08 | PSOR |
|  | rs585362 | 1 | *CELSR2* | 2.02E-40 | 1.53E-07 | 6.636E-50 | 6.31E-08 | CD |
|  | rs454107 | 1 | *CELSR2* | 5.52E-12 | 1.53E-07 | 9.744E-17 | 1.40E-07 | RA |
|  | rs413380 | 1 | *CELSR2* | 5.69E-12 | 1.53E-07 | 7.622E-17 | 1.40E-07 | RA |
|  | rs437444 | 1 | *CELSR2* | 6.64E-12 | 1.53E-07 | 8.007E-15 | 1.34E-07 | RA |
|  | rs10858082 | 1 | *CELSR2* | 2.43E-27 | 1.53E-07 | 1.294E-55 | 5.48E-08 | CD |
|  | rs4970833 | 1 | *CELSR2* | 8.83E-39 | 1.53E-07 | 7.007E-55 | 1.35E-07 | CD;UC |
|  | rs17035630 | 1 | *CELSR2* | 1.77E-14 | 1.53E-07 | 1.438E-16 | 1.26E-07 | CD |
|  | rs4970834 | 1 | *CELSR2* | 4.85E-132 | 1.53E-07 | 2.02E-208 | 8.25E-08 | CD |
|  | rs611917 | 1 | *CELSR2* | 1.40E-108 | 1.53E-07 | 1.9E-151 | 1.17E-07 | RA |
|  | rs7528419 | 1 | *CELSR2* | 3.29E-62 | 1.53E-07 | 1.55E-165 | 1.34E-07 | RA |
|  | rs658435 | 1 | *CELSR2* | 1.11E-07 | 0.000103 | 3.368E-10 | 8.26E-05 | RA |
|  | rs646776 | 1 | *CELSR2* | 3.38E-177 | 1.53E-07 | 1.63E-272 | 9.95E-08 | CeD |
|  | rs17035949 | 1 | *PSRC1* | 1.58E-18 | 1.53E-07 | 3.332E-16 | 1.32E-07 | CeD |
|  | rs10410 | 1 | *PSRC1* | 6.42E-09 | 7.73E-06 | 6.197E-11 | 6.30E-06 | RA |
|  | rs14000 | 1 | *PSRC1* | 1.02E-07 | 0.000103 | 1.071E-09 | 7.17E-05 | CeD |
|  | rs657420 | 1 | *PSRC1* | 3.87E-40 | 1.53E-07 | 6.963E-60 | 2.43E-08 | PSOR |
|  | rs655246 | 1 | *MYBPHL* | 7.81E-28 | 1.53E-07 | 1.683E-45 | 2.77E-08 | PSOR |
|  | rs17584208 | 1 | *MYBPHL* | 4.11E-29 | 1.53E-07 | 4.386E-47 | 1.29E-07 | UC |
|  | rs12127701 | 1 | *MYBPHL* | 8.27E-26 | 1.53E-07 | 4.542E-36 | 1.60E-07 | RA |
|  | rs629001 | 1 | *MYBPHL* | 1.22E-24 | 1.53E-07 | 2.279E-35 | 1.39E-07 | SARC |
|  | rs11583969 | 1 | *MYBPHL* | 1.64E-06 | 0.0012 | 5.585E-06 | 0.000809 | T1D |
|  | rs1278286 | 1 | *MYBPHL* | 2.87E-13 | 1.53E-07 | 8.824E-14 | 1.81E-07 | RA |
|  | rs17646665 | 1 | *SORT1** | 1.02E-14 | 1.53E-07 | 1.549E-22 | 1.30E-07 | CeD |
|  | rs17646731 | 1 | *SORT1** | 5.22E-17 | 1.53E-07 | 3.969E-16 | 1.10E-07 | RA |
|  | rs4970752 | 1 | *SORT1** | 2.22E-13 | 1.53E-07 | 1.172E-12 | 1.31E-07 | PSOR |
|  | rs10745354 | 1 | *SORT1** | 1.29E-12 | 1.53E-07 | 1.9E-14 | 1.22E-07 | PSOR |
|  | rs17586966 | 1 | *PSMA5* | 1.46E-15 | 1.53E-07 | 7.259E-23 | 1.10E-07 | RA |
|  | rs17647543 | 1 | *PSMA5* | 4.67E-17 | 1.53E-07 | 3.347E-24 | 1.10E-07 | RA |
| 27 | rs12145544 | 1 | *MOSC2* | 9.63E-07 | 0.000799 | 7.246E-06 | 0.000808 | RA |
|  | rs2807834* | 1 | *MOSC1** | 1.93E-11 | 1.53E-07 | 1.19E-15 | 1.50E-07 | T1D |
|  | rs2642442 | 1 | *MOSC1** | 3.93E-11 | 1.53E-07 | 5.268E-11 | 1.47E-07 | RA |
|  | rs2807837 | 1 | *MOSC1** | 6.88E-06 | 0.00397 | 7.635E-06 | 0.00219 | RA |
|  | rs10863565 | 1 | *MOSC1** | 5.15E-11 | 1.53E-07 | 9.977E-09 | 1.41E-07 | SARC |
|  | rs2642420 | 1 | *MOSC1** | 2.98E-10 | 4.50E-07 | 6.168E-09 | 3.29E-07 | RA |
|  | rs17649913 | 1 | *MOSC1** | 2.62E-06 | 0.00179 | 1.116E-08 | 0.00122 | CD |
|  | rs10495152 | 1 | *MOSC1** | 5.86E-09 | 7.73E-06 | 3.45E-07 | 6.97E-06 | RA |
|  | rs7544735 | 1 | *BC045735* | 3.28E-10 | 4.50E-07 | 3.36E-11 | 3.18E-07 | RA |
|  | rs17008917 | 1 | *BC045735* | 9.35E-09 | 1.20E-05 | 2.214E-08 | 9.74E-06 | RA |
| 28 | rs1329125 | 1 | *IRF2BP2** | 2.33E-05 | 0.0128 | 2.145E-06 | 0.00447 | CD |
| 31 | rs12616258 | 2 | *C2orf43* | 7.71E-06 | 0.00481 | 5.151E-06 | 0.0057 | RA |
|  | rs4971548 | 2 | *C2orf43* | 9.96E-06 | 0.00585 | 3.212E-06 | 0.0046 | CD |
|  | rs4971546 | 2 | *C2orf43* | 3.44E-06 | 0.00219 | 1.095E-05 | 0.000563 | PSOR |
|  | rs4971547 | 2 | *C2orf43* | 2.29E-06 | 0.00179 | 2.244E-05 | 0.000403 | PSOR |
|  | rs4971549 | 2 | *C2orf43* | 3.60E-07 | 0.000351 | 3.15E-06 | 0.000441 | RA |
|  | rs12469525 | 2 | *C2orf43* | 4.94E-06 | 0.00327 | 4.559E-06 | 0.00219 | UC |
|  | rs666471 | 2 | *C2orf43* | 7.94E-06 | 0.00481 | 7.344E-06 | 0.00196 | T1D |
|  | rs16988072 | 2 | *C2orf43* | 4.71E-06 | 0.00327 | 1.529E-09 | 0.00259 | UC |
|  | rs492399 | 2 | *C2orf43* | 3.48E-06 | 0.00219 | 1.225E-09 | 0.0018 | RA |
|  | rs6727365 | 2 | *C2orf43* | 3.75E-14 | 1.53E-07 | 2.882E-19 | 1.11E-07 | PSOR |
|  | rs7569093 | 2 | *C2orf43* | 4.25E-11 | 1.53E-07 | 7.583E-10 | 6.31E-08 | CD |
|  | rs7569328 | 2 | *C2orf43* | 1.47E-05 | 0.0087 | 2.86E-05 | 0.00124 | PSOR |
|  | rs6722139 | 2 | *C2orf43* | 4.36E-12 | 1.53E-07 | 1.316E-11 | 8.73E-08 | PSOR |
|  | rs11673889 | 2 | *C2orf43* | 1.77E-30 | 1.53E-07 | 2.113E-27 | 4.75E-08 | CD |
|  | rs12710745 | 2 | *C2orf43* | 2.05E-19 | 1.53E-07 | 2.004E-28 | 7.23E-08 | CD |
|  | rs3903032 | 2 | *C2orf43* | 7.09E-14 | 1.53E-07 | 1.981E-20 | 1.46E-07 | CD |
|  | rs4971538 | 2 | *C2orf43* | 5.40E-16 | 1.53E-07 | 2.375E-20 | 1.41E-07 | CD |
|  | rs10198175 | 2 | *APOB** | 8.12E-22 | 1.53E-07 | 1.449E-32 | 8.73E-08 | PSOR |
|  | rs6734506 | 2 | *APOB** | 8.66E-27 | 1.53E-07 | 3.248E-25 | 1.53E-07 | RA |
|  | rs4341893 | 2 | *APOB** | 6.68E-27 | 1.53E-07 | 3.677E-51 | 1.49E-07 | CD |
|  | rs4362515 | 2 | *APOB** | 4.56E-15 | 1.53E-07 | 2.22E-13 | 1.38E-07 | CeD |
|  | rs4643493 | 2 | *APOB** | 1.05E-21 | 1.53E-07 | 5.538E-30 | 1.49E-07 | CD;SARC |
|  | rs9306897 | 2 | *APOB** | 5.10E-28 | 1.53E-07 | 9.513E-48 | 1.53E-07 | RA;T1D |
|  | rs3935557 | 2 | *APOB** | 1.80E-20 | 1.53E-07 | 1.351E-24 | 1.38E-07 | SARC |
|  | rs3923037 | 2 | *APOB** | 9.03E-15 | 1.53E-07 | 1.648E-28 | 1.05E-07 | CD |
|  | rs4665788 | 2 | *APOB** | 2.15E-29 | 1.53E-07 | 1.117E-52 | 1.40E-07 | UC |
|  | rs10495712 | 2 | *APOB** | 9.40E-29 | 1.53E-07 | 2.306E-50 | 1.35E-07 | CeD |
|  | rs10164442 | 2 | *APOB** | 7.60E-15 | 1.53E-07 | 7.784E-13 | 1.38E-07 | SARC |
|  | rs13392272 | 2 | *APOB** | 1.31E-86 | 1.53E-07 | 1.46E-118 | 1.45E-07 | UC |
|  | rs6756501 | 2 | *APOB** | 1.56E-30 | 1.53E-07 | 5.291E-39 | 1.44E-07 | SARC |
|  | rs1042034 | 2 | *APOB** | 6.20E-26 | 1.53E-07 | 7.435E-39 | 1.67E-07 | RA |
|  | rs1042031 | 2 | *APOB** | 1.58E-24 | 1.53E-07 | 3.61E-24 | 1.44E-07 | SARC |
|  | rs1801701 | 2 | *APOB** | 2.51E-12 | 1.53E-07 | 2.302E-21 | 1.38E-07 | CeD |
|  | rs676210 | 2 | *APOB** | 4.69E-26 | 1.53E-07 | 4.112E-39 | 1.21E-07 | CeD |
|  | rs6413458 | 2 | *APOB** | 1.76E-08 | 1.87E-05 | 2.188E-10 | 1.66E-05 | RA |
|  | rs693 | 2 | *APOB** | 2.00E-93 | 1.53E-07 | 1.2E-131 | 1.30E-07 | CeD |
|  | rs533617 | 2 | *APOB** | 4.00E-21 | 1.53E-07 | 9.628E-45 | 1.23E-07 | RA |
|  | rs673548 | 2 | *APOB** | 4.28E-26 | 1.53E-07 | 2.718E-38 | 1.22E-07 | CeD |
|  | rs2854725 | 2 | *APOB** | 1.92E-11 | 1.53E-07 | 4.989E-19 | 1.29E-07 | RA |
|  | rs12713956 | 2 | *APOB** | 1.17E-17 | 1.53E-07 | 1.322E-28 | 3.64E-08 | PSOR |
|  | rs12691202 | 2 | *APOB** | 1.68E-16 | 1.53E-07 | 8.223E-19 | 1.90E-07 | RA |
| 32 | rs570877 | 2 | *APOB** | 1.77E-23 | 1.53E-07 | 1.044E-40 | 5.67E-08 | PSOR |
|  | rs12720842 | 2 | *APOB** | 5.47E-12 | 1.53E-07 | 1.877E-15 | 1.23E-07 | RA |
|  | rs12720796 | 2 | *APOB** | 1.32E-06 | 0.000979 | 1.68E-10 | 0.00117 | RA |
|  | rs1367117* | 2 | *APOB** | 1.57E-119 | 1.53E-07 | 9.48E-183 | 1.18E-07 | RA |
|  | rs12714264 | 2 | *APOB** | 9.19E-79 | 1.53E-07 | 3.17E-113 | 1.34E-07 | RA |
|  | rs7575840 | 2 | *APOB** | 3.34E-103 | 1.53E-07 | 1.45E-153 | 1.41E-07 | SARC |
|  | rs7567653 | 2 | *APOB** | 8.20E-19 | 1.53E-07 | 3.368E-26 | 1.44E-07 | SARC |
|  | rs540156 | 2 | *APOB** | 4.83E-06 | 0.00327 | 4.318E-05 | 0.00168 | T1D |
|  | rs754524 | 2 | *APOB** | 1.23E-77 | 1.53E-07 | 4.7E-111 | 1.30E-07 | UC |
|  | rs312985 | 2 | *APOB** | 2.96E-85 | 1.53E-07 | 1.36E-118 | 1.16E-07 | CD |
|  | rs12471982 | 2 | *APOB** | 6.41E-08 | 6.74E-05 | 3.931E-11 | 1.55E-05 | CD |
|  | rs538928 | 2 | *APOB** | 4.44E-84 | 1.53E-07 | 1.217E-75 | 1.40E-07 | RA |
|  | rs567071 | 2 | *APOB** | 1.95E-59 | 1.53E-07 | 2.419E-84 | 1.31E-07 | PSOR |
|  | rs1878512 | 2 | *APOB** | 1.15E-80 | 1.53E-07 | 2.94E-122 | 1.26E-07 | CD |
|  | rs7605304 | 2 | *APOB** | 9.05E-39 | 1.53E-07 | 4.752E-49 | 1.32E-07 | UC |
|  | rs386397 | 2 | *APOB** | 2.93E-20 | 1.53E-07 | 4.44E-24 | 1.29E-07 | RA |
|  | rs10198972 | 2 | *APOB** | 9.67E-16 | 1.53E-07 | 5.329E-25 | 1.17E-07 | RA |
|  | rs10166144 | 2 | *APOB** | 2.34E-47 | 1.53E-07 | 1.245E-44 | 1.10E-07 | RA |
|  | rs10166647 | 2 | *APOB** | 1.50E-20 | 1.53E-07 | 3.301E-20 | 1.32E-07 | UC |
|  | rs312028 | 2 | *APOB** | 2.63E-09 | 3.22E-06 | 1.02E-08 | 2.86E-06 | CeD |
|  | rs312046 | 2 | *APOB** | 1.33E-21 | 1.53E-07 | 2.775E-26 | 1.21E-07 | CeD |
|  | rs312049 | 2 | *APOB** | 2.04E-21 | 1.53E-07 | 2.485E-26 | 1.30E-07 | CeD |
|  | rs7578637 | 2 | *APOB** | 2.82E-06 | 0.00179 | 1.204E-06 | 0.00209 | RA |
|  | rs4665662 | 2 | *APOB** | 9.47E-06 | 0.00585 | 1.813E-05 | 0.00227 | CD |
|  | rs13396400 | 2 | *APOB** | 5.85E-15 | 1.53E-07 | 1.01E-18 | 1.15E-07 | CeD |
|  | rs10199484 | 2 | *APOB** | 6.81E-15 | 1.53E-07 | 7.049E-14 | 1.49E-07 | CD |
|  | rs13014768 | 2 | *APOB** | 1.63E-29 | 1.53E-07 | 7.618E-28 | 1.49E-07 | SARC |
|  | rs394167 | 2 | *APOB** | 2.02E-14 | 1.53E-07 | 2.504E-13 | 1.45E-07 | UC |
|  | rs6739502 | 2 | *APOB** | 4.93E-20 | 1.53E-07 | 5.27E-25 | 1.22E-07 | CeD |
|  | rs4665702 | 2 | *APOB** | 5.27E-13 | 1.53E-07 | 1.661E-12 | 1.44E-07 | SARC |
|  | rs219549 | 2 | *APOB** | 1.34E-14 | 1.53E-07 | 2.278E-14 | 2.77E-08 | PSOR |
| 36 | rs1025447 | 2 | *DYNC2LI1* | 1.14E-09 | 1.68E-06 | 3.782E-16 | 1.50E-06 | RA |
|  | rs10208987 | 2 | *ABCG5** | 8.41E-08 | 8.33E-05 | 2.359E-12 | 7.01E-05 | RA |
|  | rs4131228 | 2 | *ABCG5** | 1.32E-08 | 1.50E-05 | 1.206E-07 | 1.69E-05 | RA |
|  | rs6756629 | 2 | *ABCG5** | 1.44E-17 | 1.53E-07 | 1.285E-49 | 4.12E-08 | CD |
|  | rs4299376* | 2 | *ABCG8** | 1.03E-49 | 1.53E-07 | 3.937E-72 | 1.45E-07 | UC |
|  | rs6544713 | 2 | *ABCG8** | 1.43E-49 | 1.53E-07 | 4.843E-83 | 1.46E-07 | CD |
|  | rs4148214 | 2 | *ABCG8** | 7.91E-16 | 1.53E-07 | 3.753E-25 | 1.22E-07 | PSOR |
| 37 | rs4603816 | 2 | *ABCG8** | 3.45E-41 | 1.53E-07 | 1.158E-37 | 1.60E-07 | RA |
|  | rs4148217 | 2 | *ABCG8** | 8.20E-13 | 1.53E-07 | 2.255E-16 | 1.46E-08 | PSOR |
| 38 | rs4148222 | 2 | *ABCG8** | 5.01E-06 | 0.00327 | 6.002E-05 | 0.00226 | CeD |
|  | rs2954804 | 2 | *ABCG8** | 2.36E-05 | 0.0128 | 0.0008279 | 0.00885 | CD |
|  | rs7599981 | 2 | *LRPPRC* | 5.02E-06 | 0.00327 | 2.465E-05 | 0.00267 | RA |
| 68 | rs6453099 | 5 | *GCNT4* | 7.71E-10 | 1.09E-06 | 5.66E-09 | 7.75E-07 | RA |
|  | rs10043960 | 5 | *GCNT4* | 3.20E-10 | 4.50E-07 | 3.257E-10 | 3.29E-07 | RA |
|  | rs4382144 | 5 | *GCNT4* | 9.74E-11 | 1.53E-07 | 3.246E-15 | 1.14E-07 | RA |
|  | rs6889166 | 5 | *GCNT4* | 6.83E-11 | 1.53E-07 | 1.762E-10 | 1.14E-07 | RA |
|  | rs6888013 | 5 | *GCNT4* | 1.90E-05 | 0.0106 | 7.451E-06 | 0.00385 | PSOR |
|  | rs7700719 | 5 | *GCNT4* | 1.53E-08 | 1.87E-05 | 4.071E-08 | 1.27E-05 | RA |
|  | rs3935470 | 5 | *GCNT4* | 1.42E-14 | 1.53E-07 | 1.591E-27 | 1.44E-07 | SARC |
|  | rs4703645 | 5 | *GCNT4* | 3.34E-17 | 1.53E-07 | 1.9E-16 | 1.14E-07 | RA |
|  | rs3923323 | 5 | *GCNT4* | 4.22E-17 | 1.53E-07 | 1.401E-16 | 1.18E-07 | RA |
|  | rs10462511 | 5 | *GCNT4* | 3.65E-17 | 1.53E-07 | 5.213E-17 | 1.49E-07 | SARC |
|  | rs6872586 | 5 | *AK097510* | 7.57E-11 | 1.53E-07 | 1.736E-10 | 1.42E-07 | CeD |
|  | rs9654427 | 5 | *AK097510* | 3.75E-06 | 0.00268 | 1.577E-10 | 0.0019 | RA |
|  | rs2219745 | 5 | *AK097510* | 3.34E-09 | 4.01E-06 | 2.364E-08 | 3.02E-06 | CeD |
|  | rs6866661 | 5 | *AK097510* | 2.59E-19 | 1.53E-07 | 2.636E-18 | 1.30E-07 | CeD |
|  | rs7711235 | 5 | *AK097510* | 6.22E-10 | 8.70E-07 | 4.997E-10 | 8.70E-07 | CD |
|  | rs2006760 | 5 | *AK097510* | 1.45E-14 | 1.53E-07 | 1.668E-13 | 1.31E-07 | PSOR |
|  | rs10062361 | 5 | *AK097510* | 3.11E-27 | 1.53E-07 | 5.406E-52 | 1.31E-07 | UC |
|  | rs1551894 | 5 | *AK097510* | 2.80E-27 | 1.53E-07 | 4.166E-48 | 1.30E-07 | CeD |
|  | rs2335418 | 5 | *HMGCR** | 2.42E-38 | 1.53E-07 | 8.583E-65 | 1.10E-07 | RA |
|  | rs3761738 | 5 | *HMGCR** | 2.84E-14 | 1.53E-07 | 5.017E-13 | 1.49E-07 | SARC |
|  | rs3761739 | 5 | *HMGCR** | 6.30E-15 | 1.53E-07 | 7.162E-20 | 1.14E-07 | RA |
|  | rs11742194 | 5 | *HMGCR** | 4.07E-14 | 1.53E-07 | 8.126E-22 | 1.47E-07 | CeD |
|  | rs12916* | 5 | *HMGCR** | 4.03E-47 | 1.53E-07 | 7.792E-78 | 1.24E-07 | RA |
|  | rs17562686 | 5 | *COL4A3BP* | 7.75E-13 | 1.53E-07 | 1.093E-11 | 1.35E-07 | SARC |
|  | rs10515197 | 5 | *COL4A3BP* | 1.08E-14 | 1.53E-07 | 6.842E-16 | 1.17E-07 | RA |
|  | rs13356670 | 5 | *COL4A3BP* | 6.63E-13 | 1.53E-07 | 8.341E-22 | 1.67E-07 | RA |
|  | rs3761742 | 5 | *COL4A3BP* | 1.89E-21 | 1.53E-07 | 1.36E-39 | 1.23E-07 | CeD |
|  | rs6453133 | 5 | *COL4A3BP* | 3.71E-19 | 1.53E-07 | 1.081E-32 | 1.11E-07 | RA |
|  | rs12659791 | 5 | *COL4A3BP* | 1.88E-14 | 1.53E-07 | 1.422E-18 | 1.14E-07 | RA |
|  | rs5744661 | 5 | *POLK* | 3.80E-07 | 0.000351 | 1.235E-07 | 0.000214 | UC |
|  | rs4045166 | 5 | *AK307154* | 1.81E-16 | 1.53E-07 | 5.675E-32 | 1.41E-07 | SARC |
|  | rs253407 | 5 | *AK307154* | 2.05E-11 | 1.53E-07 | 8.085E-20 | 1.34E-07 | RA |
|  | rs16872670 | 5 | *AK307154* | 1.39E-05 | 0.00713 | 1.796E-09 | 0.00481 | UC |
|  | rs7717355 | 5 | *AK307154* | 3.82E-11 | 1.53E-07 | 2.319E-13 | 1.16E-07 | CD |
|  | rs253414 | 5 | *AK307154* | 2.06E-24 | 1.53E-07 | 8.179E-34 | 5.48E-08 | CD |
|  | rs7715739 | 5 | *AK307154* | 2.50E-10 | 3.62E-07 | 2.763E-11 | 3.52E-07 | CD |
|  | rs40059 | 5 | *AK307154* | 3.54E-14 | 1.53E-07 | 8.994E-22 | 1.10E-07 | RA |
|  | rs10056022 | 5 | *C5orf37* | 1.78E-08 | 2.32E-05 | 1.874E-10 | 1.45E-05 | SARC |
|  | rs888786 | 5 | *C5orf37* | 5.64E-11 | 1.53E-07 | 1.388E-10 | 1.22E-07 | PSOR |
|  | rs10515214 | 5 | *C5orf37* | 5.38E-12 | 1.53E-07 | 3.618E-16 | 1.67E-07 | RA |
|  | rs10057967 | 5 | *C5orf37* | 1.29E-21 | 1.53E-07 | 1.21E-21 | 9.34E-08 | CD |
|  | rs2047059 | 5 | *C5orf37* | 1.52E-15 | 1.53E-07 | 2.992E-21 | 1.11E-07 | RA |
|  | rs17564079 | 5 | *C5orf37* | 8.10E-06 | 0.00481 | 4.526E-06 | 0.00391 | PSOR |
|  | rs7727150 | 5 | *C5orf37* | 1.24E-05 | 0.00713 | 4.966E-09 | 0.00444 | T1D |
|  | rs17564561 | 5 | *C5orf37* | 9.95E-06 | 0.00585 | 2.705E-05 | 0.00679 | RA |
|  | rs10805894 | 5 | *C5orf37* | 1.62E-05 | 0.0087 | 3.872E-05 | 0.00537 | T1D |
| 73 | rs462058 | 5 | *SGCD* | 1.64E-05 | 0.0087 | 2.589E-05 | 0.00399 | CD |
|  | rs172240 | 5 | *SGCD* | 1.16E-05 | 0.00713 | 1.981E-06 | 0.00311 | T1D |
|  | rs1827368 | 5 | *SGCD* | 4.08E-06 | 0.00268 | 5.365E-06 | 0.00283 | RA |
|  | rs4704810 | 5 | *PPP1R2P3* | 2.31E-06 | 0.00179 | 8.279E-09 | 0.00184 | T1D |
|  | rs7717984 | 5 | *TIMD4** | 1.08E-17 | 1.53E-07 | 1.6E-24 | 7.63E-09 | CD |
|  | rs6873053 | 5 | *TIMD4** | 1.10E-08 | 1.20E-05 | 6.683E-11 | 9.74E-06 | RA |
|  | rs7724832 | 5 | *TIMD4** | 3.54E-19 | 1.53E-07 | 2.867E-25 | 7.63E-09 | CD |
|  | rs1363232 | 5 | *TIMD4** | 5.97E-20 | 1.53E-07 | 2.089E-27 | 7.63E-09 | CD |
|  | rs1501908 | 5 | *TIMD4** | 7.86E-21 | 1.53E-07 | 1.116E-28 | 7.63E-09 | CD |
|  | rs4704728 | 5 | *TIMD4** | 2.03E-13 | 1.53E-07 | 2.252E-13 | 1.18E-07 | RA |
|  | rs2277025 | 5 | *hHAVcr-1* | 3.21E-10 | 4.50E-07 | 1.651E-14 | 3.54E-07 | CD |
|  | rs6878732 | 5 | *hHAVcr-1* | 3.65E-08 | 4.41E-05 | 6.459E-12 | 7.25E-06 | CD |
|  | rs1501909 | 5 | *hHAVcr-1* | 6.54E-09 | 7.73E-06 | 9.225E-14 | 5.46E-06 | CD |
|  | rs1553318 | 5 | *hHAVcr-1* | 2.53E-20 | 1.53E-07 | 2.547E-22 | 1.34E-07 | RA |
| 75 | rs9464867 | 6 | *MYLIP** | 1.72E-11 | 1.53E-07 | 1.114E-16 | 1.90E-07 | RA |
|  | rs3757354* | 6 | *MYLIP** | 3.71E-12 | 1.53E-07 | 2.087E-17 | 1.49E-07 | CD |
|  | rs9370867 | 6 | *MYLIP** | 3.49E-06 | 0.00219 | 6.091E-06 | 0.000272 | T1D |
|  | rs2327951 | 6 | *GMPR* | 1.91E-08 | 2.32E-05 | 3.484E-10 | 8.37E-06 | T1D |
| 76 | rs12210098 | 6 | *TRIM38* | 4.54E-05 | 0.0224 | 5.167E-06 | 0.00198 | T1D |
|  | rs12216125 | 6 | *TRIM38* | 0.000208 | 0.066 | 1.458E-05 | 0.00651 | T1D |
|  | rs16891235 | 6 | *HIST1H1A* | 0.000275 | 0.0781 | 0.0002051 | 0.00791 | T1D |
|  | rs1800562* | 6 | *HFE** | 2.34E-10 | 3.62E-07 | 8.253E-14 | 1.84E-07 | CeD |
|  | rs13214703 | 6 | *OR2B6* | 9.82E-05 | 0.039 | 1.202E-06 | 0.00361 | T1D |
| 77 | rs3893464 | 6 | *HLA** | 8.20E-05 | 0.0325 | 0.0002906 | 0.00328 | T1D |
|  | rs9378220 | 6 | *TRIM31* | 2.76E-05 | 0.0128 | 0.0001289 | 0.0018 | CeD |
|  | rs9368624 | 6 | *TRIM15* | 4.82E-05 | 0.0224 | 0.0002417 | 0.0021 | PSOR |
| 78 | rs1264344 | 6 | *C6orf214* | 9.37E-05 | 0.039 | 5.173E-06 | 0.00372 | PSOR |
|  | rs13210132 | 6 | *HCG22* | 3.17E-06 | 0.00219 | 0.0009053 | 0.000175 | PSOR |
|  | rs9264601 | 6 | *HLA** | 1.96E-05 | 0.0106 | nan | 0.00198 | RA |
|  | rs9264603 | 6 | *HLA** | 7.25E-07 | 0.000651 | 0.001464 | 0.000145 | RA |
|  | rs2249742 | 6 | *HLA** | 3.67E-06 | 0.00268 | 2.527E-06 | 0.000215 | PSOR |
|  | rs13191343 | 6 | *HLA** | 4.59E-07 | 0.000431 | 5.319E-05 | 9.96E-05 | RA |
|  | rs2844533 | 6 | *MICA* | 0.000114 | 0.0466 | 3.95E-05 | 0.0044 | T1D |
|  | rs13437088 | 6 | *MICA* | 6.46E-08 | 6.74E-05 | 5.413E-07 | 5.24E-06 | PSOR |
|  | rs7771971 | 6 | *MICA* | 2.80E-08 | 2.87E-05 | 2.383E-07 | 1.21E-05 | RA |
|  | rs2523454 | 6 | *MICA* | 0.000195 | 0.066 | 2.251E-06 | 0.00651 | T1D |
|  | rs2256175 | 6 | *MICA* | 0.000102 | 0.039 | 1.646E-07 | 0.00361 | T1D |
|  | rs9501106 | 6 | *MICA* | 1.03E-07 | 0.000103 | 2.318E-10 | 1.80E-05 | CeD |
|  | rs4413654 | 6 | *HCG26* | 2.83E-05 | 0.0155 | 3.502E-07 | 0.00132 | T1D |
|  | rs2071593 | 6 | *BAT1* | 4.22E-05 | 0.0186 | 3.917E-07 | 0.00162 | T1D |
|  | rs2230365 | 6 | *NFKBIL1* | 2.35E-05 | 0.0128 | 6.136E-05 | 0.00127 | CD |
|  | rs1052248 | 6 | *LST1* | 3.26E-06 | 0.00219 | 3.597E-05 | 0.000175 | PSOR |
|  | rs2857595 | 6 | *NCR3* | 0.000111 | 0.039 | 6.651E-06 | 0.00361 | T1D |
|  | rs2857697 | 6 | *AIF1* | 2.94E-05 | 0.0155 | 0.0006061 | 0.00141 | PSOR |
|  | rs3130617 | 6 | *C6orf47* | 3.73E-05 | 0.0186 | 0.0001336 | 0.00162 | T1D |
|  | rs3117577 | 6 | *MSH5* | 0.000165 | 0.0556 | 1.294E-05 | 0.00541 | PSOR |
|  | rs644045 | 6 | *C2* | 4.27E-08 | 4.41E-05 | 5.043E-07 | 3.40E-06 | PSOR |
|  | rs592229 | 6 | *SKIV2L* | 3.32E-07 | 0.000287 | 1.391E-07 | 2.26E-05 | PSOR |
|  | rs7774197 | 6 | *TNXB* | 4.67E-05 | 0.0224 | 2.972E-10 | 0.00305 | CeD |
|  | rs6930777 | 6 | *BTNL2* | 1.56E-11 | 1.53E-07 | 3.027E-11 | 5.24E-08 | UC |
|  | rs3763313 | 6 | *BTNL2* | 1.79E-11 | 1.53E-07 | 3.143E-14 | 7.63E-09 | CD |
|  | rs2395174 | 6 | *HLA** | 0.000106 | 0.039 | 0.000198 | 0.00372 | PSOR |
|  | rs9268626 | 6 | *HLA** | 2.63E-05 | 0.0128 | 0.000285 | 0.00234 | RA |
|  | rs3129872 | 6 | *HLA** | 5.30E-05 | 0.0224 | 0.000113 | 0.0021 | PSOR |
|  | rs13209234 | 6 | *HLA** | 5.89E-15 | 1.53E-07 | 1.005E-15 | 8.81E-09 | PSOR |
|  | rs2040406 | 6 | *HLA** | 4.61E-05 | 0.0224 | 9.913E-05 | 0.00368 | RA |
|  | rs9272346 | 6 | *HLA** | 1.05E-05 | 0.00585 | 3.106E-05 | 0.000497 | T1D |
|  | rs3891175 | 6 | *HLA** | 3.05E-05 | 0.0155 | 7.533E-06 | 0.00274 | RA |
|  | rs9469220 | 6 | *HLA** | 1.54E-06 | 0.0012 | 3.511E-08 | 9.48E-05 | CD |
|  | rs9275292 | 6 | *HLA** | 6.62E-06 | 0.00397 | 4.885E-05 | 0.000801 | RA |
|  | rs9275427 | 6 | *HLA** | 6.81E-09 | 7.73E-06 | 2.441E-08 | 1.28E-06 | PSOR |
|  | rs17427887 | 6 | *HLA** | 8.25E-05 | 0.0325 | 0.0003678 | 0.0049 | RA |
|  | rs2858331 | 6 | *HLA** | 7.79E-07 | 0.000651 | 2.367E-09 | 4.99E-05 | CD |
|  | rs10807113 | 6 | *HLA** | 4.96E-06 | 0.00327 | 0.0002707 | 0.000275 | CD |
|  | rs1383264 | 6 | *HLA** | 2.05E-06 | 0.00147 | 2.647E-05 | 0.000117 | CD |
|  | rs2071472 | 6 | *HLA** | 2.48E-06 | 0.00179 | 9.335E-07 | 0.000143 | PSOR |
|  | rs2621321 | 6 | *TAP2* | 2.13E-07 | 0.000191 | 4.694E-08 | 1.41E-05 | CD |
|  | rs241447 | 6 | *TAP2* | 2.59E-07 | 0.000234 | 8.194E-08 | 1.74E-05 | CD |
| 79 | rs2281389 | 6 | *HLA** | 0.000118 | 0.0466 | 0.002092 | 0.0044 | T1D |
|  | rs2064473 | 6 | *HLA** | 4.50E-05 | 0.0224 | 0.0009934 | 0.00368 | RA |
| 83 | rs868943 | 6 | *FRK** | 1.40E-09 | 1.68E-06 | 8.439E-11 | 1.44E-06 | SARC |
|  | rs6909746 | 6 | *FRK** | 1.29E-09 | 1.68E-06 | 7.86E-11 | 1.82E-06 | CeD |
| 87 | rs1112073 | 6 | *LPAL2* | 3.07E-07 | 0.000287 | 3.487E-07 | 0.000338 | RA |
|  | rs11751605 | 6 | *LPA** | 3.65E-09 | 4.99E-06 | 5.831E-10 | 3.18E-06 | CD |
|  | rs6415084 | 6 | *LPA** | 3.16E-07 | 0.000287 | 1.349E-10 | 8.63E-05 | PSOR |
|  | rs3798221 | 6 | *LPA** | 3.21E-13 | 1.53E-07 | 1.056E-15 | 1.40E-07 | UC |
|  | rs1367211 | 6 | *LPA** | 1.09E-05 | 0.00585 | 7.226E-09 | 0.00432 | UC |
|  | rs1367210 | 6 | *LPA** | 1.10E-10 | 1.53E-07 | 6.764E-09 | 1.49E-07 | CD |
|  | rs2315065 | 6 | *PLG* | 8.41E-13 | 1.53E-07 | 5.23E-12 | 1.67E-07 | RA |
| 90 | rs2107449 | 7 | *SP4* | 4.09E-06 | 0.00268 | 1.371E-07 | 0.0021 | CD |
|  | rs6461563 | 7 | *SP4* | 5.15E-06 | 0.00327 | 1.37E-06 | 0.000894 | T1D |
|  | rs12669528 | 7 | *SP4* | 3.00E-07 | 0.000287 | 2.572E-07 | 0.00026 | RA |
|  | rs7811417 | 7 | *SP4* | 4.74E-06 | 0.00327 | 1.168E-06 | 0.00137 | T1D |
|  | rs2285950 | 7 | *DNAH11** | 7.07E-06 | 0.00397 | 1.394E-05 | 0.00207 | CD |
|  | rs12670798* | 7 | *DNAH11** | 2.66E-10 | 3.62E-07 | 4.812E-14 | 3.52E-07 | CD |
|  | rs7779983 | 7 | *DNAH11** | 3.53E-07 | 0.000287 | 5.569E-07 | 2.00E-04 | UC |
| 93 | rs217406 | 7 | *NPC1L1** | 1.68E-11 | 1.53E-07 | 4.09E-14 | 1.29E-07 | RA |
|  | rs17725246 | 7 | *NPC1L1** | 3.95E-10 | 5.59E-07 | 1.487E-20 | 5.46E-07 | CeD |
|  | rs217385 | 7 | *DDX56* | 2.46E-11 | 1.53E-07 | 4.395E-19 | 1.81E-07 | RA |
|  | rs217381 | 7 | *DDX56* | 1.58E-11 | 1.53E-07 | 3.053E-18 | 1.49E-07 | SARC |
|  | rs217375 | 7 | *DDX56* | 1.31E-09 | 1.68E-06 | 2.173E-17 | 1.63E-06 | PSOR |
|  | rs7723 | 7 | *TMED4* | 4.96E-06 | 0.00327 | 8.197E-09 | 0.00183 | T1D |
| 96 | rs330096 | 8 | *AK055863* | 1.07E-07 | 0.000103 | 3.38E-07 | 4.29E-05 | CD |
|  | rs713286 | 8 | *AK055863* | 5.83E-06 | 0.00397 | 4.499E-10 | 0.00386 | PSOR |
|  | rs330093 | 8 | *AK055863* | 8.05E-09 | 9.64E-06 | 4.638E-12 | 1.22E-05 | RA |
|  | rs983309 | 8 | *AK055863* | 4.72E-14 | 1.53E-07 | 1.325E-22 | 1.32E-07 | CeD |
|  | rs12543276 | 8 | *AK055863* | 4.04E-08 | 4.41E-05 | 4.74E-10 | 2.10E-05 | CD |
|  | rs17716118 | 8 | *AK055863* | 1.60E-07 | 0.000156 | 1.141E-12 | 0.000152 | CD |
|  | rs2126259* | 8 | *AK055863* | 1.67E-15 | 1.53E-07 | 1.348E-22 | 8.25E-08 | CD |
|  | rs1461729 | 8 | *AK055863* | 3.62E-13 | 1.53E-07 | 6.697E-13 | 7.23E-08 | CD |
|  | rs7004769 | 8 | *AK055863* | 1.80E-08 | 2.32E-05 | 5.203E-08 | 3.04E-06 | CD |
|  | rs11781511 | 8 | *AK055863* | 1.44E-11 | 1.53E-07 | 1.222E-14 | 1.35E-07 | CD;CeD |
|  | rs4332138 | 8 | *AK055863* | 9.96E-11 | 1.53E-07 | 7.889E-14 | 1.46E-07 | CD |
|  | rs7846549 | 8 | *AK055863* | 4.85E-06 | 0.00327 | 5.621E-05 | 0.00342 | PSOR |
| 99 | rs1030431* | 8 | *UBXN2B* | 1.62E-09 | 2.09E-06 | 5.968E-17 | 2.53E-06 | RA |
|  | rs7007181 | 8 | *UBXN2B* | 2.88E-09 | 4.01E-06 | 1.739E-08 | 3.14E-06 | CD |
|  | rs13277801 | 8 | *UBXN2B* | 2.89E-09 | 4.01E-06 | 3.994E-17 | 3.14E-06 | CD |
|  | rs4738679 | 8 | *UBXN2B* | 6.59E-09 | 7.73E-06 | 9.219E-08 | 6.46E-06 | CD |
| 101 | rs2385114 | 8 | *TRIB1** | 5.73E-13 | 1.53E-07 | 1.054E-19 | 5.48E-08 | CD |
|  | rs4871598 | 8 | *TRIB1** | 3.48E-12 | 1.53E-07 | 7.396E-21 | 1.40E-07 | UC |
|  | rs4518686 | 8 | *TRIB1** | 2.58E-18 | 1.53E-07 | 1.394E-23 | 1.47E-07 | CeD |
|  | rs10956248 | 8 | *TRIB1** | 1.14E-13 | 1.53E-07 | 1.417E-16 | 1.30E-07 | UC |
|  | rs2954018 | 8 | *TRIB1** | 6.70E-12 | 1.53E-07 | 5.236E-20 | 3.10E-08 | CD |
|  | rs2001945 | 8 | *TRIB1** | 5.67E-24 | 1.53E-07 | 2.296E-35 | 7.23E-08 | CD |
|  | rs2001845 | 8 | *TRIB1** | 9.14E-11 | 1.53E-07 | 6.323E-19 | 4.12E-08 | CD |
|  | rs2954020 | 8 | *TRIB1** | 3.12E-12 | 1.53E-07 | 2.647E-20 | 5.48E-08 | CD |
|  | rs17406109 | 8 | *TRIB1** | 9.30E-11 | 1.53E-07 | 2.87E-17 | 5.24E-08 | UC |
|  | rs2980862 | 8 | *TRIB1** | 3.32E-12 | 1.53E-07 | 2.966E-19 | 4.75E-08 | CD |
|  | rs8180991 | 8 | *TRIB1** | 3.11E-10 | 4.50E-07 | 1.799E-22 | 3.45E-07 | UC |
| 103 | rs11784762 | 8 | *PLEC1** | 1.47E-11 | 1.53E-07 | 4.695E-10 | 1.45E-07 | UC |
|  | rs7832643 | 8 | *PLEC1** | 1.71E-12 | 1.53E-07 | 2.671E-17 | 1.40E-07 | UC |
|  | rs7010330 | 8 | *PLEC1** | 4.68E-12 | 1.53E-07 | 2.112E-10 | 1.32E-07 | UC |
|  | rs11136341* | 8 | *PLEC1** | 1.21E-13 | 1.53E-07 | 7.112E-12 | 1.60E-07 | RA |
|  | rs11136343 | 8 | *PARP10* | 4.22E-11 | 1.53E-07 | 2.267E-09 | 1.30E-07 | UC |
| 110 | rs4489379 | 9 | *OBP2B* | 2.77E-07 | 0.000234 | 4.318E-15 | 0.000216 | CD |
|  | rs8176731 | 9 | *ABO** | 1.29E-10 | 1.90E-07 | 3.6E-15 | 1.99E-07 | RA |
|  | rs2073824 | 9 | *ABO** | 1.13E-10 | 1.90E-07 | 4.063E-15 | 1.64E-07 | CeD |
|  | rs8176720 | 9 | *ABO** | 1.92E-10 | 2.92E-07 | 1.589E-17 | 2.86E-07 | CD |
|  | rs687621 | 9 | *ABO** | 6.26E-17 | 1.53E-07 | 2.926E-23 | 1.47E-07 | RA |
|  | rs657152 | 9 | *ABO** | 1.55E-12 | 1.53E-07 | 3.981E-18 | 2.07E-08 | CD |
|  | rs505922 | 9 | *ABO** | 1.11E-16 | 1.53E-07 | 2.723E-24 | 7.23E-08 | CD |
|  | rs630014 | 9 | *ABO** | 3.29E-10 | 4.50E-07 | 2.103E-16 | 1.66E-07 | CD |
|  | rs579459 | 9 | *ABO** | 2.58E-22 | 1.53E-07 | 2.419E-44 | 1.05E-07 | CD |
|  | rs495828 | 9 | *ABO** | 2.56E-22 | 1.53E-07 | 1.296E-40 | 1.05E-07 | CD |
| 115 | rs1129555* | 10 | *GPAM** | 8.74E-10 | 1.09E-06 | 1.538E-13 | 9.27E-07 | CD |
|  | rs2419604 | 10 | *GPAM** | 1.62E-09 | 2.09E-06 | 7.49E-14 | 7.97E-07 | CeD |
| 118 | rs108499 | 11 | *C11orf9* | 6.78E-19 | 1.53E-07 | 1.166E-18 | 7.63E-09 | CD |
|  | rs174532 | 11 | *C11orf9* | 6.92E-12 | 1.53E-07 | 3.13E-16 | 8.25E-08 | CD |
|  | rs174537 | 11 | *C11orf9* | 6.74E-22 | 1.53E-07 | 3.942E-39 | 7.63E-09 | CD |
|  | rs102275 | 11 | *C11orf10* | 6.21E-22 | 1.53E-07 | 7.605E-40 | 7.63E-09 | CD |
|  | rs412334 | 11 | *FEN1* | 1.26E-05 | 0.00713 | 1.074E-06 | 0.00254 | T1D |
|  | rs4246215 | 11 | *FEN1* | 8.46E-22 | 1.53E-07 | 4.474E-31 | 7.63E-09 | CD |
|  | rs174556 | 11 | *FADS1** | 8.67E-21 | 1.53E-07 | 2.536E-37 | 7.63E-09 | CD |
|  | rs174570 | 11 | *FADS2** | 7.88E-14 | 1.53E-07 | 4.418E-21 | 1.16E-08 | CD |
|  | rs2845573 | 11 | *FADS2** | 1.12E-10 | 1.53E-07 | 1.235E-17 | 4.75E-08 | CD |
|  | rs174576 | 11 | *FADS2** | 9.78E-22 | 1.53E-07 | 4.415E-40 | 7.63E-09 | CD |
|  | rs174585 | 11 | *FADS2** | 8.98E-11 | 1.53E-07 | 1.297E-13 | 8.76E-08 | RA |
|  | rs2851682 | 11 | *FADS2** | 8.70E-12 | 1.53E-07 | 3.919E-17 | 8.25E-08 | CD |
|  | rs174450 | 11 | *FADS3** | 2.59E-12 | 1.53E-07 | 4.058E-16 | 1.34E-07 | RA |
|  | rs174456 | 11 | *FADS3** | 1.21E-05 | 0.00713 | 1.107E-10 | 0.00153 | RA |
|  | rs174478 | 11 | *RAB3IL1* | 2.57E-07 | 0.000234 | 8.858E-11 | 0.000236 | CeD |
| 121 | rs481843 | 11 | *BUD13* | 5.77E-07 | 0.000529 | 1.004E-10 | 4.00E-04 | UC |
|  | rs11820504 | 11 | *BUD13* | 1.49E-05 | 0.0087 | 1.56E-05 | 0.00982 | RA |
|  | rs2155216 | 11 | *BUD13* | 3.30E-06 | 0.00219 | 6.291E-07 | 0.00147 | UC |
|  | rs2186670 | 11 | *BUD13* | 4.89E-06 | 0.00327 | 1.596E-06 | 0.00293 | CD |
|  | rs7350481 | 11 | *BUD13* | 4.75E-15 | 1.53E-07 | 3.264E-20 | 1.49E-07 | SARC |
|  | rs12272004 | 11 | *BUD13* | 7.66E-12 | 1.53E-07 | 8.243E-19 | 1.19E-07 | CeD |
|  | rs12280724 | 11 | *BUD13* | 3.70E-11 | 1.53E-07 | 1.183E-19 | 1.46E-07 | PSOR |
|  | rs12280753 | 11 | *BUD13* | 3.31E-11 | 1.53E-07 | 1.446E-17 | 1.46E-07 | PSOR |
|  | rs12292921 | 11 | *BUD13* | 1.08E-11 | 1.53E-07 | 7.214E-20 | 1.19E-07 | CeD |
|  | rs180327 | 11 | *BUD13* | 1.20E-07 | 0.000127 | 1.369E-07 | 0.000158 | RA |
|  | rs180326 | 11 | *BUD13* | 3.94E-07 | 0.000351 | 3.022E-11 | 0.000192 | PSOR |
|  | rs6589565 | 11 | *BUD13* | 1.06E-16 | 1.53E-07 | 8.725E-16 | 1.11E-07 | RA |
|  | rs964184* | 11 | *ZNF259* | 9.04E-28 | 1.53E-07 | 2.008E-26 | 1.35E-07 | CeD |
|  | rs1942478 | 11 | *ZNF259* | 4.29E-09 | 4.99E-06 | 2.022E-10 | 4.06E-06 | CeD |
|  | rs4417316 | 11 | *ZNF259* | 3.53E-10 | 4.50E-07 | 3.029E-11 | 4.44E-07 | CD |
|  | rs6589566 | 11 | *ZNF259* | 3.54E-15 | 1.53E-07 | 1.727E-23 | 1.34E-07 | RA |
|  | rs2266788 | 11 | *APOA5** | 7.19E-15 | 1.53E-07 | 3.774E-23 | 1.34E-07 | RA |
|  | rs6589567 | 11 | *APOA5** | 5.07E-12 | 1.53E-07 | 3.117E-14 | 1.40E-07 | UC |
|  | rs1263167 | 11 | *APOA4** | 1.60E-05 | 0.0087 | 0.00172 | 0.00604 | PSOR |
|  | rs482371 | 11 | *SIK3* | 8.56E-06 | 0.00481 | 8.101E-05 | 0.000565 | T1D |
|  | rs579890 | 11 | *SIK3* | 7.54E-07 | 0.000651 | 2.183E-07 | 0.000353 | RA |
|  | rs7122944 | 11 | *PAFAH1B2* | 1.55E-06 | 0.0012 | 1.013E-05 | 0.000356 | T1D |
|  | rs90192 | 11 | *SIDT2* | 3.10E-06 | 0.00219 | 0.0001441 | 0.000709 | T1D |
|  | rs641620 | 11 | *TAGLN* | 4.77E-07 | 0.000431 | 0.0001223 | 0.000221 | RA |
|  | rs508487 | 11 | *TAGLN* | 2.41E-10 | 3.62E-07 | 1.773E-10 | 2.66E-07 | RA |
| 124 | rs17135399 | 11 | *CR602684* | 1.43E-11 | 1.53E-07 | 2.165E-20 | 4.88E-08 | PSOR |
|  | rs4937122 | 11 | *ST3GAL4** | 1.68E-12 | 1.53E-07 | 1.812E-20 | 4.21E-08 | PSOR |
|  | rs10893499 | 11 | *ST3GAL4** | 1.22E-15 | 1.53E-07 | 3.861E-21 | 1.41E-07 | SARC |
|  | rs3862628 | 11 | *ST3GAL4** | 1.03E-10 | 1.53E-07 | 3.44E-11 | 1.29E-07 | RA |
|  | rs3862629 | 11 | *ST3GAL4** | 2.10E-12 | 1.53E-07 | 9.16E-14 | 1.29E-07 | RA |
|  | rs10893502 | 11 | *ST3GAL4** | 6.68E-07 | 0.000529 | 4.99E-05 | 0.000508 | RA |
| 125 | rs10893505 | 11 | *ST3GAL4** | 1.62E-06 | 0.0012 | 1.387E-08 | 0.0012 | RA |
|  | rs11220472 | 11 | *ST3GAL4** | 3.61E-06 | 0.00268 | 1.947E-08 | 0.00128 | T1D |
| 127 | rs3184504 | 12 | *SH2B3* | 7.00E-10 | 8.70E-07 | 4.203E-12 | 3.51E-07 | CeD |
|  | rs630512 | 12 | *ATXN2* | 2.18E-07 | 0.000191 | 4.072E-08 | 3.38E-05 | T1D |
|  | rs11065987* | 12 | *BRAP** | 6.05E-10 | 8.70E-07 | 1.204E-11 | 3.51E-07 | CeD |
|  | rs11066028 | 12 | *ALDH2* | 2.32E-09 | 3.22E-06 | 7.661E-10 | 1.88E-06 | CeD |
|  | rs11066320 | 12 | *PTPN11* | 6.82E-09 | 7.73E-06 | 1.568E-09 | 2.00E-06 | CeD |
| 130 | rs1186380 | 12 | *C12orf27* | 3.86E-06 | 0.00268 | 8.463E-08 | 0.00155 | T1D |
|  | rs2650000 | 12 | *C12orf27* | 7.05E-15 | 1.53E-07 | 2.396E-16 | 1.41E-07 | SARC |
|  | rs11065374 | 12 | *C12orf27* | 2.55E-07 | 0.000234 | 1.664E-07 | 0.000121 | SARC |
|  | rs11065376 | 12 | *C12orf27* | 2.97E-06 | 0.00219 | 1.134E-05 | 0.00259 | RA |
|  | rs7953249 | 12 | *C12orf27* | 1.26E-11 | 1.53E-07 | 8.471E-14 | 1.30E-07 | UC |
|  | rs1169286 | 12 | *HNF1A** | 9.81E-12 | 1.53E-07 | 2.266E-12 | 1.30E-07 | UC |
|  | rs1169302 | 12 | *HNF1A** | 9.06E-10 | 1.35E-06 | 1.433E-09 | 1.26E-06 | SARC |
|  | rs2259816 | 12 | *HNF1A** | 2.74E-13 | 1.53E-07 | 3.659E-17 | 5.79E-08 | UC |
|  | rs1169307 | 12 | *HNF1A** | 1.80E-05 | 0.0106 | 3.264E-05 | 0.00571 | CD |
|  | rs735396 | 12 | *HNF1A** | 1.94E-13 | 1.53E-07 | 5.364E-16 | 5.24E-08 | UC |
|  | rs10849829 | 12 | *OASL* | 1.47E-10 | 2.36E-07 | 3.034E-11 | 1.95E-07 | UC |
|  | rs3213545 | 12 | *OASL* | 5.95E-15 | 1.53E-07 | 5.764E-17 | 1.30E-07 | UC |
|  | rs2708101 | 12 | *OASL* | 6.49E-08 | 6.74E-05 | 4.452E-09 | 6.00E-05 | UC |
| 137 | rs11629005 | 14 | *KIAA1305** | 5.13E-06 | 0.00327 | 6.339E-06 | 0.00272 | SARC |
| 138 | rs6573778 | 14 | *KIAA1305** | 3.78E-11 | 1.53E-07 | 3.332E-14 | 1.48E-07 | SARC |
|  | rs8017377 | 14 | *KIAA1305** | 1.66E-11 | 1.53E-07 | 2.516E-15 | 1.31E-07 | PSOR |
| 143 | rs12448528 | 16 | *HERPUD1* | 2.36E-06 | 0.00179 | 1.057E-12 | 0.00178 | RA |
|  | rs173539 | 16 | *CETP** | 3.14E-13 | 1.53E-07 | 2.241E-35 | 1.81E-07 | RA |
|  | rs3764261 | 16 | *CETP** | 4.75E-13 | 1.53E-07 | 2.22E-34 | 1.28E-07 | CeD |
|  | rs1864163 | 16 | *CETP** | 2.06E-08 | 2.32E-05 | 7.968E-21 | 2.02E-05 | CeD |
|  | rs9939224 | 16 | *CETP** | 5.42E-09 | 6.21E-06 | 4.648E-08 | 7.04E-06 | RA |
|  | rs11076174 | 16 | *CETP** | 1.17E-05 | 0.00713 | 3.224E-06 | 0.00744 | CeD |
|  | rs7205804 | 16 | *CETP** | 3.83E-11 | 1.53E-07 | 3.833E-26 | 1.32E-07 | UC |
|  | rs1532624 | 16 | *CETP** | 3.76E-11 | 1.53E-07 | 1.844E-26 | 1.32E-07 | UC |
|  | rs7499892 | 16 | *CETP** | 4.16E-08 | 4.41E-05 | 2.157E-18 | 3.14E-05 | SARC |
| 144 | rs289719 | 16 | *CETP** | 1.59E-05 | 0.0087 | 6.774E-07 | 0.00866 | RA |
|  | rs289742 | 16 | *CETP** | 2.12E-05 | 0.0106 | 3.389E-05 | 0.00833 | CD |
| 145 | rs17286411 | 16 | *PKD1L3* | 5.41E-06 | 0.00327 | 3.301E-06 | 0.00213 | SARC |
|  | rs12708919 | 16 | *PKD1L3* | 2.55E-08 | 2.87E-05 | 2.684E-08 | 1.88E-05 | SARC |
|  | rs9925462 | 16 | *PKD1L3* | 4.25E-12 | 1.53E-07 | 1.268E-11 | 1.51E-07 | T1D |
|  | rs12921355 | 16 | *PKD1L3* | 1.98E-15 | 1.53E-07 | 1.493E-14 | 1.53E-07 | RA |
|  | rs10492825 | 16 | *PKD1L3* | 2.84E-15 | 1.53E-07 | 3.665E-25 | 1.32E-07 | UC |
|  | rs4788589 | 16 | *PKD1L3* | 4.17E-07 | 0.000351 | 1.07E-09 | 0.000248 | CD |
|  | rs4788591 | 16 | *PKD1L3* | 9.01E-07 | 0.000799 | 3.025E-09 | 0.000493 | CeD |
|  | rs17664900 | 16 | *PKD1L3* | 3.11E-06 | 0.00219 | 1.861E-10 | 0.000427 | T1D |
|  | rs3213422 | 16 | *DHODH* | 5.14E-06 | 0.00327 | 4.458E-11 | 0.00203 | CD |
|  | rs2000999* | 16 | *TXNL4B* | 1.69E-23 | 1.53E-07 | 4.219E-41 | 1.41E-07 | T1D |
|  | rs217181 | 16 | *TXNL4B* | 2.20E-11 | 1.53E-07 | 1.131E-25 | 1.16E-08 | CD |
|  | rs9302635 | 16 | *DHX38* | 3.07E-10 | 4.50E-07 | 2.033E-15 | 3.62E-07 | UC |
|  | rs17666993 | 16 | *DHX38* | 1.15E-06 | 0.000979 | 3.207E-06 | 0.000681 | T1D |
|  | rs12926250 | 16 | *PMFBP1* | 2.25E-06 | 0.00179 | 1.446E-05 | 0.00112 | T1D |
|  | rs10492816 | 16 | *PMFBP1* | 1.46E-06 | 0.0012 | 1.611E-07 | 0.00116 | UC |
|  | rs2023929 | 16 | *PMFBP1* | 3.34E-06 | 0.00219 | 2.242E-06 | 0.000656 | CD |
|  | rs8060878 | 16 | *PMFBP1* | 4.81E-10 | 6.97E-07 | 8.601E-12 | 5.49E-07 | CD |
|  | rs16970719 | 16 | *PMFBP1* | 5.38E-07 | 0.000431 | 8.472E-06 | 0.000535 | RA |
| 149 | rs11652772 | 17 | *MYL4* | 3.24E-05 | 0.0155 | 2.07E-07 | 0.0056 | CeD |
|  | rs7225700* | 17 | *ITGB3* | 1.65E-09 | 2.09E-06 | 3.558E-13 | 2.04E-06 | CD |
|  | rs6504833 | 17 | *ITGB3* | 2.42E-09 | 3.22E-06 | 1.375E-07 | 1.40E-06 | T1D |
|  | rs11652957 | 17 | *ITGB3* | 3.54E-06 | 0.00219 | 2.284E-06 | 0.00132 | T1D |
| 158 | rs9305020 | 19 | *LDLR** | 7.36E-70 | 1.53E-07 | 4.91E-151 | 1.11E-07 | PSOR |
|  | rs7249753 | 19 | *LDLR** | 5.90E-06 | 0.00397 | 2.828E-07 | 0.00181 | T1D |
|  | rs11668477 | 19 | *LDLR** | 1.07E-71 | 1.53E-07 | 3.8E-143 | 1.35E-07 | CeD |
|  | rs6511720* | 19 | *LDLR** | 1.07E-122 | 1.53E-07 | 3.85E-262 | 7.61E-08 | PSOR |
|  | rs2228671 | 19 | *LDLR** | 5.17E-84 | 1.53E-07 | 1.74E-171 | 1.31E-07 | PSOR |
| 159 | rs12983082 | 19 | *LDLR** | 1.40E-19 | 1.53E-07 | 1.522E-18 | 1.11E-07 | RA |
|  | rs5930 | 19 | *LDLR** | 7.57E-35 | 1.53E-07 | 3.708E-38 | 4.12E-08 | CD |
|  | rs1799898 | 19 | *LDLR** | 3.08E-08 | 3.56E-05 | 1.962E-09 | 2.29E-05 | RA |
|  | rs5927 | 19 | *LDLR** | 5.72E-11 | 1.53E-07 | 2.767E-13 | 1.14E-07 | RA |
|  | rs2569537 | 19 | *LDLR** | 6.83E-11 | 1.53E-07 | 1.348E-10 | 1.41E-07 | CD |
|  | rs2116897 | 19 | *LDLR** | 9.98E-11 | 1.53E-07 | 5.48E-11 | 1.35E-07 | CD |
|  | rs1433099 | 19 | *LDLR** | 6.72E-12 | 1.53E-07 | 2.51E-16 | 1.14E-07 | RA |
|  | rs4804147 | 19 | *SPC24* | 3.04E-22 | 1.53E-07 | 5.31E-29 | 1.23E-07 | RA |
|  | rs892115 | 19 | *SPC24* | 1.58E-08 | 1.87E-05 | 2.209E-10 | 1.14E-05 | T1D |
|  | rs7251031 | 19 | *SPC24* | 1.79E-13 | 1.53E-07 | 6.236E-23 | 8.76E-08 | RA |
|  | rs7188 | 19 | *KANK2* | 8.75E-17 | 1.53E-07 | 9.389E-31 | 8.76E-08 | RA |
| 173 | rs4803759 | 19 | *BCAM* | 6.17E-34 | 1.53E-07 | 9.503E-33 | 1.31E-07 | PSOR |
|  | rs11668536 | 19 | *BCAM* | 4.24E-14 | 1.53E-07 | 4.302E-26 | 1.44E-07 | SARC |
|  | rs10402271 | 19 | *BCAM* | 6.58E-66 | 1.53E-07 | 2.6E-118 | 1.47E-07 | CeD |
|  | rs4803760 | 19 | *BCAM* | 3.62E-77 | 1.53E-07 | 2.47E-123 | 8.81E-09 | PSOR |
|  | rs2972564 | 19 | *PVRL2* | 3.25E-14 | 1.53E-07 | 2.765E-22 | 1.10E-07 | RA |
|  | rs3852856 | 19 | *PVRL2* | 1.05E-16 | 1.53E-07 | 1.775E-15 | 2.43E-08 | PSOR |
|  | rs4803767 | 19 | *PVRL2* | 7.69E-20 | 1.53E-07 | 4.11E-18 | 1.45E-07 | UC |
|  | rs519113 | 19 | *PVRL2* | 1.52E-48 | 1.53E-07 | 1.607E-49 | 1.11E-07 | PSOR |
|  | rs387976 | 19 | *PVRL2* | 4.65E-46 | 1.53E-07 | 7.952E-45 | 5.67E-08 | PSOR |
|  | rs6859 | 19 | *PVRL2* | 1.19E-38 | 1.53E-07 | 4.646E-88 | 1.35E-07 | CD |
|  | rs3852861 | 19 | *PVRL2* | 7.81E-08 | 8.33E-05 | 8.48E-17 | 1.93E-05 | PSOR |
|  | rs283813 | 19 | *PVRL2* | 7.56E-51 | 1.53E-07 | 1.26E-93 | 1.47E-07 | RA |
|  | rs7254892 | 19 | *PVRL2* | 1.79E-93 | 1.53E-07 | 0 | 1.58E-07 | UC |
|  | rs6857 | 19 | *PVRL2* | 8.46E-116 | 1.53E-07 | 5.12E-110 | 1.47E-07 | RA |
|  | rs157580 | 19 | *TOMM40* | 1.25E-84 | 1.53E-07 | 9.24E-119 | 1.38E-07 | SARC |
|  | rs2075650 | 19 | *TOMM40* | 2.04E-115 | 1.53E-07 | 1.72E-214 | 1.26E-07 | CD |
|  | rs8106922 | 19 | *TOMM40* | 6.33E-18 | 1.53E-07 | 2.97E-22 | 1.29E-07 | RA |
|  | rs405509 | 19 | *APOE** | 3.66E-33 | 1.53E-07 | 2.843E-84 | 9.91E-08 | PSOR |
|  | rs439401 | 19 | *APOE** | 2.84E-07 | 0.000287 | 1.044E-12 | 9.82E-05 | CD |
|  | rs445925 | 19 | *APOC1** | 1.02E-62 | 1.53E-07 | 0 | 1.29E-07 | UC |
| 186 | rs11697014 | 20 | *MAFB** | 1.54E-05 | 0.0087 | 6.329E-06 | 0.00605 | UC |
| 187 | rs6029123 | 20 | *MAFB** | 3.24E-07 | 0.000287 | 5.195E-08 | 7.86E-05 | CD |
|  | rs2902941* | 20 | *MAFB** | 4.90E-09 | 6.21E-06 | 4.205E-11 | 5.34E-06 | RA |
|  | rs2143877 | 20 | *MAFB** | 4.01E-08 | 4.41E-05 | 6.12E-11 | 4.10E-05 | RA |
|  | rs16989081 | 20 | *MAFB** | 1.20E-06 | 0.000979 | 5.073E-05 | 0.000888 | RA |
|  | rs6029143 | 20 | *MAFB** | 1.38E-06 | 0.000979 | 4.144E-05 | 0.000687 | CeD |
| 188 | rs6016373 | 20 | *MAFB** | 6.47E-08 | 6.74E-05 | 7.947E-19 | 5.48E-05 | CeD |
|  | rs6016382 | 20 | *MAFB** | 5.32E-08 | 5.45E-05 | 1.426E-07 | 5.97E-05 | RA |
| 189 | rs2865507 | 20 | *MAFB** | 7.69E-06 | 0.00481 | 3.201E-08 | 0.00547 | RA |
|  | rs6029214 | 20 | *MAFB** | 7.08E-06 | 0.00397 | 2.214E-05 | 0.00248 | CD |
|  | rs6029228 | 20 | *MAFB** | 3.83E-05 | 0.0186 | 7.955E-05 | 0.00953 | CD |
|  | rs6016399 | 20 | *MAFB** | 5.41E-07 | 0.000431 | 9.575E-07 | 0.000524 | RA |
|  | rs6129653 | 20 | *MAFB** | 6.45E-07 | 0.000529 | 2.553E-11 | 4.00E-04 | UC |
|  | rs6072090 | 20 | *MAFB** | 3.27E-06 | 0.00219 | 5.27E-06 | 0.00165 | T1D |
| 190 | rs2425421 | 20 | *MAFB** | 8.36E-06 | 0.00481 | 1.088E-06 | 0.00302 | CeD |
|  | rs6029315 | 20 | *MAFB** | 6.37E-06 | 0.00397 | 1.486E-05 | 0.00472 | RA |
| 191 | rs6029516 | 20 | *CR612573* | 3.85E-08 | 4.41E-05 | 1.749E-07 | 3.21E-05 | RA |
|  | rs6016501 | 20 | *CR612573* | 9.74E-09 | 1.20E-05 | 6.053E-08 | 8.88E-06 | RA |
|  | rs12625035 | 20 | *TOP1** | 4.43E-09 | 4.99E-06 | 2.895E-11 | 3.18E-06 | CD |
|  | rs6016505 | 20 | *TOP1** | 5.84E-17 | 1.53E-07 | 1.963E-15 | 7.23E-08 | CD |
|  | rs2076574 | 20 | *TOP1** | 1.97E-07 | 0.000191 | 6.572E-08 | 0.000136 | CD |
|  | rs2866370 | 20 | *PLCG1* | 1.75E-05 | 0.0087 | 4.04E-05 | 0.00751 | PSOR |
|  | rs753381 | 20 | *PLCG1* | 1.33E-15 | 1.53E-07 | 3.571E-25 | 9.34E-08 | CD |
|  | rs2235366 | 20 | *ZHX3* | 1.18E-10 | 1.90E-07 | 1.665E-15 | 1.81E-08 | CD |
|  | rs4297946 | 20 | *ZHX3* | 6.85E-20 | 1.53E-07 | 8.954E-18 | 1.38E-07 | SARC |
|  | rs6129778 | 20 | *ZHX3* | 1.63E-10 | 2.36E-07 | 3.145E-17 | 1.81E-07 | RA |
|  | rs4812491 | 20 | *ZHX3* | 4.55E-11 | 1.53E-07 | 3.562E-17 | 1.83E-08 | CD |
|  | rs4812492 | 20 | *ZHX3* | 4.59E-14 | 1.53E-07 | 1.421E-12 | 1.35E-07 | UC |
|  | rs6029636 | 20 | *LPIN3* | 3.72E-12 | 1.53E-07 | 2.302E-11 | 1.11E-07 | RA |
|  | rs6124342 | 20 | *EMILIN3* | 1.25E-05 | 0.00713 | 6.226E-09 | 0.00806 | RA |
|  | rs6102385 | 20 | *EMILIN3* | 1.93E-05 | 0.0106 | 0.004892 | 0.00982 | PSOR |
|  | rs4810316 | 20 | *KIAA1335* | 4.69E-07 | 0.000431 | 7.559E-07 | 0.000378 | CeD |
|  | rs4812505 | 20 | *KIAA1335* | 1.32E-07 | 0.000127 | 1.269E-06 | 0.000103 | SARC |
|  | rs4142393 | 20 | *CHD6* | 7.09E-08 | 8.33E-05 | 1.347E-09 | 6.32E-05 | SARC |
|  | rs6129859 | 20 | *CHD6* | 8.19E-08 | 8.33E-05 | 6.757E-06 | 7.33E-05 | CD |
|  | rs2425463 | 20 | *CHD6* | 3.34E-06 | 0.00219 | 1.028E-07 | 0.000388 | PSOR |

Independent complex or single gene loci (r^2^ < 0.2) with SNP(s) with a conditional FDR (condFDR) < 0.01 in low density lipoprotein (LDL) condition on the associated immune mediated disease. All SNPs with a condFDR value < 0.01 are listed. For LDL and immune-mediated disease phenotype we defined the most significant SNP in each LD block based on the minimum condFDR (min FDR). For comparison, the minimal condFDR values for each identified SNP are listed for the phenotypes based on which this value was obtained (Driving phenotype, multiple phenotypes separated by semi-colon). In addition, the chromosome number (Chr), closest gene (Gene symbol), unconditional false discovery rates (FDR) and p values of each SNPs for the corresponding lipids are given. The corresponding p values from the larger sample of LDL (LDL2 P-value) are also listed. Independent loci which do not contain any known SNPs or genes are listed first consecutively, followed by loci containing SNPs or genes, which were marked by stars (*). Crohn’s Disease (CD), ulcerative colitis (UC), rheumatoid arthritis (RA), type 1 diabetes (T1D), celiac disease (CeD), psoriasis (PSOR) and sarcoidosis (SARC). Chromosome (Chr). All data were first corrected for genomic inflation. NA; not available.

### Table D. SNPs in high density lipoprotein (HDL) conditioned on immune-mediated diseases (condFDR<0.01).

| **# Locus** | **SNP** | **Chr** | **Gene**  **Symbol** | **HDL**  **P-value** | **HDL**  **FDR** | **HDL2**  **P-value** | **minFDR** | **Driving**  **phenotype** |
| --- | --- | --- | --- | --- | --- | --- | --- | --- |
| 1 | rs11247963 | 1 | *RPS6KA1* | 3.79E-06 | 0.00227 | 8.998E-06 | 0.0014 | CD |
|  | rs6666757 | 1 | *RPS6KA1* | 2.27E-07 | 0.000196 | 2.751E-07 | 0.000111 | CD |
| 2 | rs17162333 | 1 | *NR0B2* | 8.09E-08 | 7.01E-05 | 1.51E-07 | 2.45E-05 | RA |
|  | rs12742376 | 1 | *C1orf172* | 1.68E-07 | 0.00013 | 2.003E-15 | 4.35E-05 | T1D |
| 4 | rs2806425 | 1 | *NFIA* | 8.88E-05 | 0.0295 | 0.001586 | 0.00975 | CD |
| 5 | rs12118262 | 1 | *CCDC18* | 2.47E-07 | 0.000196 | 3.558E-10 | 2.25E-05 | CD |
|  | rs4240966 | 1 | *CR609342* | 9.57E-07 | 0.000666 | 1.093E-06 | 8.17E-05 | CD |
| 6 | rs12740374 | 1 | *CELSR2* | 3.49E-08 | 3.04E-05 | 1.687E-15 | 2.54E-05 | RA |
|  | rs629301 | 1 | *CELSR2* | 3.48E-08 | 3.04E-05 | 7.608E-13 | 1.81E-05 | CD |
|  | rs646776 | 1 | *CELSR2* | 3.63E-08 | 3.75E-05 | 2.722E-15 | 1.18E-05 | T1D |
| 7 | rs333947 | 1 | *CSF1* | 5.49E-05 | 0.0203 | 3.166E-09 | 0.00932 | CeD |
| 8 | rs390923 | 1 | *CSF1* | 5.22E-05 | 0.0203 | 1.045E-08 | 0.00542 | CD |
| 9 | rs4650993 | 1 | *DKFZp564J047* | 8.65E-06 | 0.00418 | 0.0001215 | 0.00135 | T1D |
|  | rs4650994 | 1 | *DKFZp564J047* | 6.26E-06 | 0.00341 | 6.696E-09 | 0.00113 | T1D |
|  | rs6660445 | 1 | *C1orf220* | 3.84E-06 | 0.00227 | 6.751E-05 | 0.00184 | RA |
|  | rs2248666 | 1 | *RALGPS2* | 9.60E-06 | 0.00511 | 1.899E-06 | 0.00467 | CD |
| 11 | rs3761916 | 1 | *DKFZp666D0110* | 3.30E-06 | 0.00185 | 1.906E-05 | 0.000917 | T1D |
|  | rs7529037 | 1 | *SLC45A3* | 1.25E-06 | 0.000819 | 1.346E-05 | 0.000491 | RA |
|  | rs3902968 | 1 | *SLC45A3* | 1.54E-06 | 0.00101 | 7.092E-06 | 0.000745 | RA |
| 12 | rs9242 | 1 | *SRGAP2* | 3.04E-05 | 0.0138 | 0.0001164 | 0.0086 | RA |
|  | rs17433769 | 1 | *SRGAP2* | 7.42E-05 | 0.0295 | 0.0002162 | 0.00316 | UC |
| 13 | rs12040334 | 1 | *RD3* | 3.39E-05 | 0.0138 | 4.432E-05 | 0.00613 | RA |
| 14 | rs4846300 | 1 | *LYPLAL1* | 1.35E-05 | 0.00623 | 0.02158 | 0.00473 | RA |
| 15 | rs2642438 | 1 | *MOSC1* | 9.22E-06 | 0.00511 | 7.781E-14 | 0.0053 | RA |
|  | rs2807834 | 1 | *MOSC1* | 1.80E-05 | 0.00927 | 7.761E-12 | 0.00716 | T1D |
|  | rs2642442 | 1 | *MOSC1* | 1.38E-05 | 0.00623 | 7.04E-06 | 0.00571 | CD |
| 18 | rs17799872 | 2 | *ADCY3* | 8.18E-05 | 0.0295 | 0.001937 | 0.00758 | T1D |
| 19 | rs4832163 | 2 | *TCF7L1* | 8.51E-08 | 7.01E-05 | 5.275E-07 | 1.66E-05 | RA |
|  | rs1053560 | 2 | *TGOLN2* | 5.76E-08 | 5.70E-05 | 6.038E-05 | 1.04E-05 | RA |
|  | rs4459734 | 2 | *TGOLN2* | 8.29E-08 | 7.01E-05 | 6.616E-07 | 1.23E-05 | RA |
|  | rs10460586 | 2 | *TGOLN2* | 6.05E-08 | 5.70E-05 | 2.113E-05 | 9.17E-06 | RA |
| 20 | rs952249 | 2 | *ACOXL* | 4.96E-06 | 0.00278 | 2.088E-05 | 0.000911 | T1D |
| 21 | rs2704531 | 2 | *LOC389033* | 5.90E-06 | 0.00341 | 1.887E-05 | 0.00178 | RA |
|  | rs2704532 | 2 | *LOC389033* | 3.97E-06 | 0.00227 | 1.291E-05 | 0.0011 | RA |
|  | rs2521920 | 2 | *LOC389033* | 3.04E-06 | 0.00185 | 0.0002716 | 0.00112 | RA |
| 22 | rs11904278 | 2 | *TMEM163* | 5.06E-06 | 0.00278 | 0.002126 | 0.00208 | T1D |
| 24 | rs16846841 | 2 | *HECW2* | 4.70E-06 | 0.00278 | 7.766E-06 | 0.00253 | CD |
| 25 | rs12468557 | 2 | *CPS1* | 3.52E-05 | 0.0138 | 5.303E-05 | 0.00672 | T1D |
|  | rs6750325 | 2 | *CPS1* | 3.20E-05 | 0.0138 | 2.824E-06 | 0.00985 | CD |
| 26 | rs4480977 | 2 | *VWC2L* | 4.93E-05 | 0.0203 | 8.986E-05 | 0.00887 | UC |
| 28 | rs4135280 | 3 | *PPARG* | 6.86E-05 | 0.0245 | 2.518E-07 | 0.0098 | CD |
| 29 | rs7613875 | 3 | *MON1A* | 8.83E-07 | 0.000543 | 1.792E-11 | 4.73E-05 | UC |
|  | rs6765484 | 3 | *RBM6* | 5.80E-06 | 0.00341 | 6.938E-11 | 0.000302 | UC |
| 30 | rs13326165 | 3 | *STAB1* | 3.43E-07 | 0.00024 | 9.042E-11 | 7.67E-05 | T1D |
| 31 | rs2581799 | 3 | *RFT1* | 5.66E-05 | 0.0245 | 3.258E-06 | 0.00254 | CD |
| 32 | rs645040 | 3 | *MSL2* | 2.12E-06 | 0.00124 | 1.525E-12 | 0.00121 | UC |
|  | rs684773 | 3 | *PCCB* | 2.60E-06 | 0.00151 | 1.37E-05 | 0.00137 | CD;UC |
|  | rs9854084 | 3 | *STAG1* | 2.51E-06 | 0.00151 | 1.111E-05 | 0.000813 | RA |
|  | rs7621025 | 3 | *STAG1* | 2.68E-06 | 0.00151 | 2.239E-12 | 0.000854 | RA |
| 33 | rs2869433 | 4 | *MAPK10* | 1.02E-05 | 0.00511 | 8.243E-06 | 0.00336 | RA |
|  | rs17417758 | 4 | *MAPK10* | 8.25E-06 | 0.00418 | 6.591E-06 | 0.00275 | RA |
| 34 | rs13149938 | 4 | *SLC10A6* | 3.22E-06 | 0.00185 | 2.191E-07 | 0.00157 | UC |
|  | rs10516787 | 4 | *AFF1* | 1.35E-06 | 0.000819 | 1.294E-05 | 0.000845 | RA |
|  | rs3775230 | 4 | *AFF1* | 1.42E-06 | 0.00101 | 2.58E-06 | 0.000746 | CD |
|  | rs1471251 | 4 | *AFF1* | 3.35E-07 | 0.00024 | 2.186E-06 | 0.000124 | UC |
|  | rs236996 | 4 | *AFF1* | 1.31E-06 | 0.000819 | 1.244E-08 | 0.000717 | PSOR |
|  | rs442177 | 4 | *AFF1* | 1.63E-07 | 0.00013 | 2.193E-09 | 0.000162 | RA |
| 35 | rs7698923 | 4 | *KLHL8* | 9.28E-06 | 0.00511 | 7.77E-06 | 0.00263 | CD |
| 36 | rs987469 | 4 | *FAM13A* | 3.86E-06 | 0.00227 | 4.272E-06 | 0.0011 | RA |
|  | rs2290782 | 4 | *FAM13A* | 6.92E-06 | 0.00341 | 7.468E-06 | 0.00168 | RA |
|  | rs13131633 | 4 | *FAM13A* | 4.17E-06 | 0.00227 | 3.315E-06 | 0.0011 | RA |
|  | rs13133548 | 4 | *FAM13A* | 1.17E-06 | 0.000819 | 5.207E-12 | 0.000389 | RA |
| 38 | rs6838128 | 4 | *NDST3* | 6.33E-06 | 0.00341 | 0.002549 | 0.000811 | CD |
| 39 | rs12645965 | 4 | *USP53* | 7.85E-06 | 0.00418 | 2.102E-05 | 0.00292 | UC |
| 41 | rs3843467 | 5 | *MAP3K1* | 5.09E-06 | 0.00278 | 7.183E-08 | 0.00122 | CD |
|  | rs9686661 | 5 | *MAP3K1* | 4.64E-07 | 0.00036 | 1.365E-08 | 0.000439 | RA |
| 42 | rs4976033 | 5 | *PIK3R1* | 2.25E-05 | 0.0113 | 6.421E-08 | 0.00882 | CD |
| 43 | rs4242231 | 5 | *AK056485* | 6.01E-06 | 0.00341 | 0.008095 | 0.00101 | CD |
| 44 | rs13207673 | 6 | *SLC17A2* | 5.78E-07 | 0.000442 | 1.957E-06 | 0.000271 | RA |
|  | rs9461222 | 6 | *SLC17A2* | 6.18E-07 | 0.000442 | 8.321E-07 | 0.000255 | RA |
|  | rs17526722 | 6 | *SLC17A2* | 1.05E-06 | 0.000666 | 1.42E-07 | 0.000235 | RA |
|  | rs3734523 | 6 | *SLC17A2* | 6.63E-07 | 0.000442 | 1.027E-06 | 0.000213 | T1D |
| 45 | rs3823417 | 6 | *PSORS1C1* | 0.000225 | 0.0723 | 2.073E-11 | 0.00817 | CD |
| 46 | rs2844571 | 6 | *HLA* | 0.000153 | 0.0508 | 5.728E-06 | 0.00941 | CD |
|  | rs2844513 | 6 | *MICA* | 6.29E-06 | 0.00341 | 2.465E-09 | 0.000429 | CD |
| 47 | rs3117583 | 6 | *BAT3* | 1.93E-05 | 0.00927 | 8.552E-08 | 0.00214 | RA |
|  | rs396960 | 6 | *NOTCH4* | 9.60E-05 | 0.0353 | 0.0001391 | 0.00904 | RA |
|  | rs3135353 | 6 | *HLA* | 6.80E-05 | 0.0245 | 1.729E-05 | 0.00229 | UC |
|  | rs12194148 | 6 | *HLA* | 3.47E-06 | 0.00185 | 4.251E-07 | 0.000363 | RA |
|  | rs7774434 | 6 | *HLA* | 0.000128 | 0.0423 | 2.074E-06 | 0.00724 | UC |
|  | rs11752643 | 6 | *HLA* | 1.42E-05 | 0.0076 | 3.134E-07 | 0.00172 | RA |
|  | rs9275572 | 6 | *HLA* | 1.47E-05 | 0.0076 | 4.448E-08 | 0.00172 | RA |
| 49 | rs998584 | 6 | *VEGFA* | 1.19E-05 | 0.00623 | 2.269E-11 | 0.00344 | RA |
| 50 | rs2055445 | 6 | *GFRAL* | 1.24E-05 | 0.00623 | 0.03013 | 0.00377 | UC |
| 51 | rs884366 | 6 | *AK094715* | 5.13E-05 | 0.0203 | 1.671E-07 | 0.00644 | T1D |
| 52 | rs9375486 | 6 | *AK127472* | 0.000159 | 0.0508 | 2.611E-06 | 0.00726 | CD |
|  | rs2800703 | 6 | *AK127472* | 3.20E-07 | 0.00024 | 6.476E-10 | 0.00016 | CD |
|  | rs1936797 | 6 | *AK127472* | 6.17E-07 | 0.000442 | 1.028E-09 | 0.000175 | T1D |
|  | rs1936806 | 6 | *RSPO3* | 3.43E-05 | 0.0138 | 2.368E-09 | 0.0014 | CD |
| 54 | rs851977 | 6 | *ESR1* | 2.01E-06 | 0.00124 | 9.118E-06 | 0.00116 | RA |
|  | rs12525163 | 6 | *ESR1* | 2.66E-06 | 0.00151 | 1.524E-07 | 0.000902 | T1D |
| 55 | rs9340788 | 6 | *ESR1* | 2.51E-05 | 0.0113 | 6.082E-05 | 0.00629 | RA |
| 56 | rs9457931 | 6 | *LPAL2* | 2.32E-07 | 0.000196 | 7.297E-13 | 0.000114 | RA |
| 58 | rs9458009 | 6 | *PLG* | 7.02E-06 | 0.00341 | 7.723E-06 | 0.00401 | RA |
| 59 | rs10951983 | 7 | *RAC1* | 1.67E-06 | 0.00101 | 3.598E-10 | 8.99E-05 | UC |
|  | rs836556 | 7 | *DAGLB* | 2.10E-06 | 0.00124 | 1.59E-05 | 0.000324 | UC |
|  | rs702485 | 7 | *DAGLB* | 8.66E-07 | 0.000543 | 6.45E-12 | 0.000138 | UC |
|  | rs12538142 | 7 | *DAGLB* | 1.59E-06 | 0.00101 | 6.246E-05 | 0.000477 | RA |
| 60 | rs38179 | 7 | *MEOX2* | 2.66E-05 | 0.0113 | 5.626E-05 | 0.00803 | T1D |
| 61 | rs10282707 | 7 | *SNX13* | 9.07E-07 | 0.000666 | 1.025E-11 | 0.000443 | CD |
|  | rs10242866 | 7 | *SNX13* | 2.08E-06 | 0.00124 | 2.457E-06 | 0.000756 | CD |
| 62 | rs4917014 | 7 | *IKZF1* | 1.66E-05 | 0.0076 | 1.026E-08 | 0.000761 | CD |
|  | rs11185603 | 7 | *IKZF1* | 1.23E-05 | 0.00623 | 7.889E-05 | 0.00236 | T1D |
|  | rs876038 | 7 | *IKZF1* | 1.27E-05 | 0.00623 | 8.008E-05 | 0.00268 | RA |
| 64 | rs2714467 | 7 | *GNAI1* | 1.28E-06 | 0.000819 | 6.221E-06 | 0.000863 | UC |
|  | rs2714449 | 7 | *GNAI1* | 1.24E-05 | 0.00623 | 0.001419 | 0.00523 | CD |
| 65 | rs10276981 | 7 | *GNAI1* | 2.32E-05 | 0.0113 | 0.0002237 | 0.00761 | UC |
| 66 | rs17232680 | 7 | *GNAI1* | 1.14E-05 | 0.00623 | 0.0001032 | 0.00507 | RA |
| 67 | rs2237717 | 7 | *MET* | 3.62E-05 | 0.0168 | 5.184E-05 | 0.0055 | CD |
|  | rs42336 | 7 | *MET* | 3.94E-05 | 0.0168 | 7.008E-05 | 0.0055 | CD |
| 69 | rs12533255 | 7 | *ABP1* | 8.00E-05 | 0.0295 | 0.001362 | 0.00842 | UC |
|  | rs2968856 | 7 | *KCNH2* | 1.58E-05 | 0.0076 | 0.00136 | 0.00908 | RA |
|  | rs2968854 | 7 | *KCNH2* | 4.34E-05 | 0.0168 | 0.001518 | 0.00668 | T1D |
| 70 | rs330096 | 8 | *AK055863* | 2.28E-08 | 2.47E-05 | 6.019E-08 | 9.07E-06 | CD |
|  | rs330093 | 8 | *AK055863* | 6.93E-10 | 8.50E-07 | 6.135E-16 | 1.07E-06 | RA |
|  | rs983309 | 8 | *AK055863* | 6.59E-20 | 1.53E-07 | 1.983E-37 | 1.22E-07 | CD |
|  | rs10099512 | 8 | *AK055863* | 1.13E-10 | 1.91E-07 | 6.752E-18 | 2.57E-07 | RA |
|  | rs12543276 | 8 | *AK055863* | 1.17E-08 | 1.32E-05 | 6.564E-20 | 5.34E-06 | CD |
|  | rs17716118 | 8 | *AK055863* | 2.52E-10 | 3.66E-07 | 5.9E-19 | 3.19E-07 | UC |
|  | rs2126259 | 8 | *AK055863* | 2.04E-23 | 1.53E-07 | 1.526E-42 | 8.09E-08 | CD |
|  | rs1461729 | 8 | *AK055863* | 1.83E-20 | 1.53E-07 | 1.358E-19 | 7.64E-08 | CD |
|  | rs7004769 | 8 | *AK055863* | 1.20E-09 | 1.61E-06 | 7.015E-09 | 3.28E-07 | CD |
|  | rs11781511 | 8 | *AK055863* | 2.81E-17 | 1.53E-07 | 1.508E-30 | 1.03E-07 | CD |
|  | rs4332138 | 8 | *AK055863* | 8.54E-15 | 1.53E-07 | 3.575E-29 | 1.14E-07 | CD |
|  | rs930991 | 8 | *AK055863* | 4.30E-05 | 0.0168 | 3.75E-10 | 0.00673 | UC |
|  | rs2169385 | 8 | *AK055863* | 1.27E-11 | 1.53E-07 | 5.007E-22 | 1.39E-07 | UC |
|  | rs7846549 | 8 | *AK055863* | 1.18E-05 | 0.00623 | 3.977E-06 | 0.00642 | RA |
| 71 | rs10903312 | 8 | *AK055863* | 1.85E-05 | 0.00927 | 6.481E-05 | 0.00924 | PSOR |
| 72 | rs7014168 | 8 | *SOX7* | 6.92E-06 | 0.00341 | 9.203E-10 | 0.00401 | RA |
|  | rs10099021 | 8 | *SOX7* | 1.67E-05 | 0.0076 | 1.272E-08 | 0.00641 | CD |
| 74 | rs13263508 | 8 | *SLC18A1* | 4.78E-22 | 1.53E-07 | 9.531E-46 | 1.50E-07 | UC |
| 75 | rs7003526 | 8 | *SLC18A1* | 5.60E-06 | 0.00278 | 6.664E-10 | 0.00129 | UC |
| 76 | rs16842 | 8 | *SLC18A1* | 1.11E-08 | 1.07E-05 | 3.822E-14 | 7.02E-06 | CD |
| 77 | rs13263568 | 8 | *BC048982* | 6.01E-06 | 0.00341 | 0.0001421 | 0.00212 | CD |
| 79 | rs4871137 | 8 | *SNTB1* | 3.74E-06 | 0.00227 | 1.926E-07 | 0.0014 | CD |
| 83 | rs7815797 | 8 | *GPIHBP1* | 3.18E-05 | 0.0138 | 4.584E-06 | 0.00744 | T1D |
| 90 | rs4979373 | 9 | *AKNA* | 4.36E-06 | 0.00227 | 1.467E-05 | 0.00194 | PSOR |
| 91 | rs970548 | 10 | *MARCH8* | 9.11E-08 | 8.61E-05 | 1.706E-10 | 7.12E-05 | CD |
|  | rs10900223 | 10 | *MARCH8* | 7.95E-08 | 7.01E-05 | 1.451E-07 | 8.89E-05 | RA |
| 92 | rs10761731 | 10 | *JMJD1C* | 1.49E-07 | 0.00013 | 3.75E-07 | 0.000162 | RA |
|  | rs10761741 | 10 | *JMJD1C* | 3.28E-07 | 0.00024 | 5.362E-08 | 0.000146 | CD |
|  | rs7923609 | 10 | *JMJD1C* | 2.37E-06 | 0.00151 | 2.556E-08 | 0.00102 | CD |
| 93 | rs1129555 | 10 | *GPAM* | 2.49E-07 | 0.000196 | 5.192E-17 | 0.000105 | CD |
|  | rs2250802 | 10 | *GPAM* | 1.27E-07 | 0.000106 | 2.022E-17 | 5.71E-05 | CD |
|  | rs2792751 | 10 | *GPAM* | 2.43E-07 | 0.000196 | 3.829E-16 | 0.000105 | CD |
| 94 | rs6585151 | 10 | *GUCY2G* | 3.99E-05 | 0.0168 | 0.0001315 | 0.00969 | CD |
| 95 | rs951585 | 10 | *TRUB1* | 8.55E-05 | 0.0295 | 0.0006534 | 0.00487 | CD |
| 97 | rs12288723 | 11 | *EF537580* | 3.10E-06 | 0.00185 | 3.873E-06 | 0.000802 | UC |
| 98 | rs11038668 | 11 | *SLC35C1* | 1.04E-05 | 0.00511 | 1.49E-06 | 0.0011 | CD |
|  | rs10838519 | 11 | *SLC35C1* | 1.30E-06 | 0.000819 | 8.619E-07 | 0.000981 | RA |
|  | rs7121775 | 11 | *CRY2* | 1.69E-05 | 0.0076 | 6.738E-06 | 0.00132 | CD |
| 99 | rs6485672 | 11 | *CREB3L1* | 3.31E-06 | 0.00185 | 3.418E-10 | 0.000647 | T1D |
| 100 | rs10838612 | 11 | *ARHGAP1* | 5.40E-13 | 1.53E-07 | 2.05E-20 | 1.14E-07 | CD |
|  | rs11038977 | 11 | *F2* | 3.99E-17 | 1.53E-07 | 6.088E-27 | 8.66E-08 | T1D |
|  | rs1007738 | 11 | *CKAP5* | 2.14E-06 | 0.00124 | 2.482E-08 | 0.00102 | CD |
|  | rs2306026 | 11 | *CR612190* | 1.88E-07 | 0.00016 | 5.971E-09 | 0.000131 | CD |
|  | rs2290883 | 11 | *CR612190* | 1.25E-15 | 1.53E-07 | 3.103E-24 | 7.51E-08 | T1D |
|  | rs17787966 | 11 | *C11orf49* | 3.84E-07 | 0.000294 | 1.078E-10 | 0.00026 | UC |
|  | rs7117404 | 11 | *C11orf49* | 2.46E-05 | 0.0113 | 5.026E-07 | 0.00964 | CD |
|  | rs7120118 | 11 | *NR1H3* | 4.11E-15 | 1.53E-07 | 1.555E-32 | 1.14E-07 | CD |
|  | rs10501321 | 11 | *MADD* | 3.78E-16 | 1.53E-07 | 3.541E-38 | 1.03E-07 | CD |
|  | rs326214 | 11 | *MADD* | 3.43E-13 | 1.53E-07 | 2.174E-36 | 1.03E-07 | CD |
|  | rs2697920 | 11 | *MYBPC3* | 3.44E-14 | 1.53E-07 | 1.003E-22 | 1.41E-07 | CD |
|  | rs12419342 | 11 | *RAPSN* | 6.22E-08 | 5.70E-05 | 4.857E-20 | 6.97E-05 | RA |
|  | rs7124681 | 11 | *CUGBP1* | 1.36E-10 | 1.91E-07 | 6.164E-22 | 7.30E-08 | T1D |
|  | rs17791016 | 11 | *AGBL2* | 2.47E-14 | 1.53E-07 | 5.534E-26 | 9.51E-08 | CD |
|  | rs10838757 | 11 | *FNBP4* | 1.09E-10 | 1.53E-07 | 5.447E-10 | 1.46E-07 | RA |
|  | rs2290850 | 11 | *NUP160* | 4.10E-11 | 1.53E-07 | 4.679E-17 | 9.06E-08 | CD |
|  | rs2305983 | 11 | *NUP160* | 4.39E-11 | 1.53E-07 | 1.29E-19 | 9.51E-08 | CD |
|  | rs6485788 | 11 | *NUP160* | 3.87E-11 | 1.53E-07 | 1.188E-19 | 9.51E-08 | CD |
|  | rs7924699 | 11 | *NUP160* | 5.78E-12 | 1.53E-07 | 2.669E-17 | 1.28E-07 | UC |
|  | rs4752894 | 11 | *PTPRJ* | 1.67E-05 | 0.0076 | 1.885E-09 | 0.00258 | CD |
|  | rs2270994 | 11 | *PTPRJ* | 8.84E-06 | 0.00418 | 3.483E-10 | 0.00157 | CD |
|  | rs10838833 | 11 | *OR4B1* | 1.05E-10 | 1.53E-07 | 2.694E-09 | 1.22E-07 | CD |
|  | rs1316604 | 11 | *OR4X1* | 2.30E-11 | 1.53E-07 | 9.731E-19 | 1.14E-07 | CD |
|  | rs7929225 | 11 | *FOLH1* | 1.27E-10 | 1.91E-07 | 3.618E-09 | 2.64E-07 | RA |
|  | rs11040208 | 11 | *FOLH1* | 1.06E-09 | 1.30E-06 | 5.638E-08 | 1.21E-06 | T1D |
|  | rs1819409 | 11 | *FOLH1* | 1.55E-09 | 1.99E-06 | 4.575E-10 | 1.15E-06 | CD |
|  | rs7117025 | 11 | *FOLH1* | 6.82E-09 | 7.10E-06 | 1.122E-07 | 4.19E-06 | CD |
|  | rs7103246 | 11 | *LOC646813* | 5.49E-08 | 4.63E-05 | 3.538E-07 | 2.91E-05 | UC |
|  | rs11246602 | 11 | *OR4C46* | 3.88E-08 | 3.75E-05 | 1.681E-10 | 1.71E-05 | CD |
|  | rs11246610 | 11 | *OR4C46* | 4.02E-07 | 0.000294 | 2.528E-08 | 0.000229 | T1D |
| 101 | rs11229165 | 11 | *OR4A16* | 2.15E-07 | 0.00016 | 1.661E-06 | 0.000189 | RA |
|  | rs11229606 | 11 | *OR4A15* | 3.43E-07 | 0.00024 | 3.117E-10 | 8.92E-05 | CD |
|  | rs11230983 | 11 | *OR5D13* | 5.58E-07 | 0.00036 | 1.875E-09 | 0.000124 | CD |
|  | rs6591243 | 11 | *OR8U8* | 5.08E-07 | 0.00036 | 1.729E-06 | 0.000102 | CD |
|  | rs684156 | 11 | *OR8U8* | 6.34E-06 | 0.00341 | 1.332E-05 | 0.000811 | CD |
|  | rs1945244 | 11 | *OR8U8* | 3.72E-06 | 0.00227 | 9.403E-06 | 0.000489 | CD |
| 102 | rs518385 | 11 | *AB231742* | 2.51E-05 | 0.0113 | 2.486E-05 | 0.00735 | CD |
| 104 | rs12801636 | 11 | *PCNXL3* | 4.97E-06 | 0.00278 | 3.147E-08 | 0.00209 | CD |
| 105 | rs10793126 | 11 | *MOGAT2* | 9.73E-06 | 0.00511 | 7.251E-05 | 0.00306 | UC |
| 107 | rs11216322 | 11 | *PCSK7* | 6.83E-08 | 5.70E-05 | 1.799E-07 | 4.30E-05 | T1D |
| 108 | rs552498 | 11 | *CEP164* | 2.63E-06 | 0.00151 | 6.193E-05 | 0.00132 | PSOR |
| 112 | rs1038164 | 12 | *MDM1* | 1.18E-05 | 0.00623 | 0.02175 | 0.00476 | T1D |
| 113 | rs2589266 | 12 | *RIC8B* | 3.47E-05 | 0.0138 | 3.449E-07 | 0.00502 | T1D |
| 115 | rs653178 | 12 | *ATXN2* | 1.01E-06 | 0.000666 | 1.064E-12 | 0.00013 | RA |
| 116 | rs895953 | 12 | *SETD1B* | 4.33E-05 | 0.0168 | 2.544E-06 | 0.00911 | CD |
| 117 | rs2454722 | 12 | *GPR81* | 1.91E-07 | 0.00016 | 3.308E-14 | 0.000145 | CD |
| 118 | rs1798192 | 12 | *GPR81* | 3.94E-05 | 0.0168 | 2.908E-07 | 0.00911 | CD |
| 119 | rs3817094 | 12 | *HIP1R* | 9.16E-08 | 8.61E-05 | 2.372E-07 | 0.000105 | RA |
|  | rs12313006 | 12 | *HIP1R* | 1.37E-06 | 0.000819 | 3.667E-06 | 0.000463 | RA |
|  | rs2271051 | 12 | *HIP1R* | 8.91E-07 | 0.000543 | 1.596E-11 | 0.000295 | RA |
| 127 | rs7298751 | 12 | *UBC* | 1.51E-09 | 1.99E-06 | 2.455E-16 | 1.33E-06 | CD |
| 128 | rs9805284 | 13 | *B3GALTL* | 1.83E-05 | 0.00927 | 6.042E-05 | 0.0097 | CeD |
| 129 | rs9600212 | 13 | *KLF12* | 6.15E-09 | 7.10E-06 | 0.7632 | 6.36E-06 | UC |
| 130 | rs4983559 | 14 | *INF2* | 6.45E-06 | 0.00341 | 9.565E-09 | 0.00113 | CD;T1D |
| 131 | rs3743031 | 15 | *RPAP1* | 4.41E-05 | 0.0168 | 5.13E-05 | 0.00149 | UC |
|  | rs2297381 | 15 | *RPAP1* | 1.33E-05 | 0.00623 | 2.846E-07 | 0.00258 | CD |
|  | rs721772 | 15 | *RPAP1* | 1.18E-05 | 0.00623 | 2.358E-07 | 0.00238 | CD |
| 132 | rs2929282 | 15 | *FRMD5* | 1.85E-06 | 0.00124 | 1.41E-06 | 0.000986 | RA |
|  | rs2929275 | 15 | *FRMD5* | 2.84E-06 | 0.00185 | 2.112E-11 | 0.00136 | UC |
| 133 | rs10444840 | 15 | *ALDH1A2* | 2.75E-05 | 0.0113 | 2.048E-08 | 0.00882 | CD |
| 134 | rs2414553 | 15 | *ALDH1A2* | 5.05E-06 | 0.00278 | 6.847E-11 | 0.00136 | UC |
|  | rs4774291 | 15 | *ALDH1A2* | 1.56E-05 | 0.0076 | 1.52E-05 | 0.00335 | UC |
|  | rs4774292 | 15 | *ALDH1A2* | 2.98E-06 | 0.00185 | 1.523E-08 | 0.000649 | CD |
|  | rs1123294 | 15 | *ALDH1A2* | 5.11E-19 | 1.53E-07 | 1.094E-18 | 1.64E-07 | RA |
|  | rs7165301 | 15 | *ALDH1A2* | 9.73E-19 | 1.53E-07 | 3.835E-28 | 2.80E-08 | UC |
|  | rs935214 | 15 | *ALDH1A2* | 2.95E-06 | 0.00185 | 2.278E-06 | 0.0015 | PSOR |
|  | rs17821159 | 15 | *ALDH1A2* | 2.29E-12 | 1.53E-07 | 6.33E-12 | 5.73E-08 | CD |
|  | rs12910051 | 15 | *ALDH1A2* | 1.91E-10 | 2.95E-07 | 3.996E-20 | 2.47E-07 | CD |
|  | rs1973688 | 15 | *ALDH1A2* | 2.33E-16 | 1.53E-07 | 9.277E-16 | 7.05E-08 | UC |
| 135 | rs1444939 | 15 | *ALDH1A2* | 4.34E-07 | 0.000294 | 1.05E-06 | 0.000132 | UC |
|  | rs11633876 | 15 | *ALDH1A2* | 2.74E-12 | 1.53E-07 | 1.862E-11 | 6.90E-08 | CD |
|  | rs261267 | 15 | *ALDH1A2* | 8.81E-12 | 1.53E-07 | 5.912E-11 | 1.73E-07 | T1D |
|  | rs1816879 | 15 | *ALDH1A2* | 3.17E-13 | 1.53E-07 | 2.141E-13 | 6.61E-08 | CD |
|  | rs1122208 | 15 | *ALDH1A2* | 5.81E-11 | 1.53E-07 | 1.168E-10 | 8.58E-08 | CD |
|  | rs11637094 | 15 | *ALDH1A2* | 7.91E-14 | 1.53E-07 | 4.176E-14 | 6.90E-08 | CD |
| 136 | rs12910827 | 15 | *ALDH1A2* | 1.31E-07 | 0.000106 | 9.146E-11 | 4.44E-05 | UC |
| 144 | rs424346 | 15 | *ADAM10* | 3.26E-06 | 0.00185 | 4.84E-08 | 0.00181 | UC |
| 146 | rs8023580 | 15 | *AK307134* | 5.40E-06 | 0.00278 | 2.258E-05 | 0.00312 | RA |
| 147 | rs8050872 | 16 | *IQCK* | 4.92E-05 | 0.0203 | 1.756E-05 | 0.00633 | T1D |
|  | rs4782291 | 16 | *IQCK* | 3.50E-05 | 0.0138 | 4.586E-05 | 0.00842 | CD |
| 148 | rs7498491 | 16 | *NPIPL2* | 6.65E-05 | 0.0245 | 8.682E-06 | 0.00254 | CD |
|  | rs9937676 | 16 | *NPIPL2* | 5.48E-05 | 0.0203 | 0.0005946 | 0.00233 | CD |
| 149 | rs1121980 | 16 | *FTO* | 1.34E-06 | 0.000819 | 6.791E-09 | 0.000462 | CD |
|  | rs9941349 | 16 | *FTO* | 2.57E-06 | 0.00151 | 1.245E-08 | 0.000811 | CD |
| 150 | rs3790106 | 16 | *GNAO1* | 6.22E-06 | 0.00341 | 3.268E-11 | 0.00401 | RA |
|  | rs9935936 | 16 | *GNAO1* | 1.06E-05 | 0.00511 | 4.896E-05 | 0.00571 | RA |
|  | rs4784659 | 16 | *GNAO1* | 1.33E-05 | 0.00623 | 1.018E-08 | 0.00523 | CD |
|  | rs3790116 | 16 | *GNAO1* | 1.22E-05 | 0.00623 | 4.126E-06 | 0.00519 | T1D |
| 151 | rs2011186 | 16 | *MT1H* | 0.000101 | 0.0353 | 0.0002604 | 0.0072 | CD |
| 152 | rs12928598 | 16 | *MT1X* | 1.74E-10 | 2.38E-07 | 2.482E-17 | 1.64E-07 | CD |
|  | rs4784724 | 16 | *NUP93* | 1.35E-12 | 1.53E-07 | 9.559E-12 | 1.74E-07 | RA |
|  | rs8049693 | 16 | *NUP93* | 1.70E-19 | 1.53E-07 | 6.113E-18 | 1.03E-07 | CD |
|  | rs12599585 | 16 | *NUP93* | 7.16E-12 | 1.53E-07 | 2.873E-11 | 6.63E-08 | T1D |
|  | rs2164514 | 16 | *NUP93* | 1.96E-07 | 0.00016 | 1.941E-07 | 9.61E-05 | UC |
|  | rs10852553 | 16 | *NUP93* | 7.26E-19 | 1.53E-07 | 2.673E-17 | 1.03E-07 | CD |
|  | rs7187512 | 16 | *NUP93* | 1.37E-21 | 1.53E-07 | 4.997E-20 | 6.33E-08 | CD |
|  | rs16962399 | 16 | *NUP93* | 6.31E-32 | 1.53E-07 | 2.336E-30 | 7.51E-08 | T1D |
|  | rs1529929 | 16 | *NUP93* | 2.03E-21 | 1.53E-07 | 7.202E-20 | 6.33E-08 | CD |
|  | rs3764266 | 16 | *NUP93* | 7.02E-33 | 1.53E-07 | 4.158E-31 | 1.31E-07 | UC |
|  | rs1138295 | 16 | *NUP93* | 6.96E-18 | 1.53E-07 | 2.05E-16 | 9.92E-08 | CD |
|  | rs4784733 | 16 | *SLC12A3* | 4.99E-14 | 1.53E-07 | 1.888E-13 | 1.03E-07 | CD |
|  | rs4329913 | 16 | *SLC12A3* | 2.27E-12 | 1.53E-07 | 1.405E-11 | 5.96E-08 | T1D |
| 157 | rs1566439 | 16 | *NLRC5* | 1.30E-08 | 1.32E-05 | 3.532E-15 | 1.09E-05 | RA |
|  | rs289748 | 16 | *NLRC5* | 5.68E-19 | 1.53E-07 | 6.629E-17 | 1.56E-07 | RA |
|  | rs289751 | 16 | *NLRC5* | 1.49E-13 | 1.53E-07 | 8.814E-25 | 1.84E-07 | T1D |
|  | rs17369578 | 16 | *NLRC5* | 1.47E-07 | 0.00013 | 7.786E-08 | 0.000112 | T1D |
|  | rs17310296 | 16 | *NLRC5* | 1.41E-08 | 1.32E-05 | 4.37E-08 | 1.63E-05 | RA |
|  | rs7198642 | 16 | *NLRC5* | 5.07E-08 | 4.63E-05 | 3.062E-14 | 2.24E-05 | RA |
|  | rs1875236 | 16 | *NLRC5* | 3.63E-11 | 1.53E-07 | 1.445E-18 | 2.08E-07 | RA |
|  | rs1991515 | 16 | *NLRC5* | 6.80E-09 | 7.10E-06 | 5.622E-10 | 6.36E-06 | UC |
| 158 | rs17370142 | 16 | *NLRC5* | 2.34E-08 | 2.47E-05 | 1.188E-11 | 7.72E-06 | CD |
|  | rs7185561 | 16 | *NLRC5* | 3.12E-13 | 1.53E-07 | 3.114E-15 | 1.84E-07 | RA |
|  | rs13335668 | 16 | *NLRC5* | 2.45E-08 | 2.47E-05 | 2.616E-11 | 9.87E-06 | RA |
|  | rs12446867 | 16 | *NLRC5* | 1.24E-12 | 1.53E-07 | 1.831E-16 | 1.31E-07 | CD |
|  | rs291040 | 16 | *NLRC5* | 2.11E-11 | 1.53E-07 | 8.21E-17 | 1.31E-07 | CD |
|  | rs289754 | 16 | *NLRC5* | 4.31E-10 | 5.59E-07 | 2.346E-15 | 2.60E-07 | T1D |
|  | rs289726 | 16 | *NLRC5* | 5.06E-13 | 1.53E-07 | 1.27E-22 | 9.51E-08 | CD |
| 159 | rs289723 | 16 | *NLRC5* | 4.87E-07 | 0.00036 | 4.079E-09 | 0.000218 | CD |
| 160 | rs150348 | 16 | *NLRC5* | 1.00E-05 | 0.00511 | 7.408E-10 | 0.0051 | UC |
| 161 | rs8051360 | 16 | *NLRC5* | 1.58E-05 | 0.0076 | 1.664E-05 | 0.00909 | RA |
| 162 | rs4783972 | 16 | *CPNE2* | 5.57E-06 | 0.00278 | 1.43E-09 | 0.0017 | T1D |
| 164 | rs1542825 | 16 | *AK057218* | 5.40E-06 | 0.00278 | 0.0002548 | 0.0016 | CD |
|  | rs1542824 | 16 | *AK057218* | 5.59E-06 | 0.00278 | 0.0001202 | 0.00105 | T1D |
| 166 | rs11652527 | 17 | *NLK* | 4.28E-08 | 3.75E-05 | 8.702E-08 | 3.12E-05 | UC |
| 168 | rs4793040 | 17 | *CD300LG* | 6.99E-05 | 0.0245 | 4.429E-07 | 0.00983 | T1D |
| 169 | rs231471 | 17 | *PPY* | 5.44E-06 | 0.00278 | 0.005938 | 0.00154 | T1D |
|  | rs151196 | 17 | *PPY* | 2.44E-06 | 0.00151 | 2.732E-05 | 0.00168 | RA |
|  | rs1642599 | 17 | *PYY* | 2.39E-06 | 0.00151 | 0.0002404 | 0.00102 | CD |
| 170 | rs9911967 | 17 | *GOSR2* | 6.55E-05 | 0.0245 | 7.011E-05 | 0.0091 | CD |
| 171 | rs10491182 | 17 | *NFE2L1* | 2.86E-05 | 0.0138 | 0.0001368 | 0.00487 | CeD |
|  | rs10491183 | 17 | *NFE2L1* | 2.81E-05 | 0.0113 | 4.134E-05 | 0.00458 | UC |
| 174 | rs12327104 | 18 | *CABLES1* | 8.50E-06 | 0.00418 | 3.152E-05 | 0.00348 | CD |
|  | rs1966656 | 18 | *CABLES1* | 6.90E-06 | 0.00341 | 1.474E-05 | 0.00232 | T1D |
|  | rs750071 | 18 | *CABLES1* | 3.85E-06 | 0.00227 | 0.0001399 | 0.00208 | UC |
| 177 | rs1787328 | 18 | *MYO5B* | 4.46E-18 | 1.53E-07 | 2.304E-26 | 1.24E-07 | T1D |
|  | rs12457612 | 18 | *MYO5B* | 1.01E-06 | 0.000666 | 5.439E-06 | 0.000519 | UC |
|  | rs13381727 | 18 | *MYO5B* | 1.54E-07 | 0.00013 | 2.094E-09 | 0.000107 | CD |
| 181 | rs737204 | 19 | *EEF2* | 5.00E-05 | 0.0203 | 0.0005156 | 0.00957 | CD |
| 182 | rs8101064 | 19 | *INSR* | 1.41E-05 | 0.0076 | 2.225E-05 | 0.00755 | PSOR |
| 184 | rs4804311 | 19 | *MYO1F* | 2.29E-05 | 0.0113 | 3.739E-14 | 0.00983 | RA |
| 185 | rs7254425 | 19 | *DNM2* | 3.16E-06 | 0.00185 | 5.385E-06 | 0.00221 | RA |
|  | rs2278444 | 19 | *DNM2* | 4.85E-06 | 0.00278 | 0.0001424 | 0.00253 | CD |
| 186 | rs755237 | 19 | *KANK2* | 4.19E-05 | 0.0168 | 0.0005002 | 0.00969 | CD |
|  | rs754528 | 19 | *KANK2* | 3.88E-05 | 0.0168 | 0.0001978 | 0.00911 | CD |
|  | rs12976810 | 19 | *KANK2* | 1.05E-05 | 0.00511 | 5.393E-05 | 0.00601 | RA |
| 188 | rs4805755 | 19 | *ZNF507* | 7.97E-06 | 0.00418 | 2.526E-06 | 0.00315 | T1D |
|  | rs2012353 | 19 | *DPY19L3* | 3.52E-06 | 0.00185 | 2.649E-06 | 0.00153 | CD |
|  | rs2111504 | 19 | *DPY19L3* | 4.00E-06 | 0.00227 | 1.808E-05 | 0.00188 | CD |
| 189 | rs8182584 | 19 | *PEPD* | 1.91E-07 | 0.00016 | 1.515E-08 | 0.000131 | CD |
| 190 | rs10402271 | 19 | *BCAM* | 1.25E-05 | 0.00623 | 6.148E-09 | 0.00561 | T1D |
|  | rs4803763 | 19 | *PVRL2* | 4.60E-07 | 0.00036 | 1.724E-06 | 0.000427 | RA |
| 191 | rs6857 | 19 | *PVRL2* | 1.30E-18 | 1.53E-07 | 2.625E-17 | 1.94E-07 | RA |
|  | rs157580 | 19 | *TOMM40* | 1.44E-07 | 0.00013 | 9.875E-12 | 5.25E-05 | T1D |
|  | rs2075650 | 19 | *TOMM40* | 2.92E-17 | 1.53E-07 | 9.716E-26 | 6.07E-08 | T1D |
| 193 | rs5167 | 19 | *APOC4* | 9.54E-07 | 0.000666 | 4.879E-16 | 0.00048 | UC |
| 194 | rs11670462 | 19 | *EML2* | 6.77E-05 | 0.0245 | 3.792E-05 | 0.0049 | CD |
| 195 | rs2303108 | 19 | *ZC3H4* | 3.04E-05 | 0.0138 | 6.318E-07 | 0.00207 | UC |
| 196 | rs2974225 | 19 | *GLTSCR1* | 4.71E-06 | 0.00278 | 2.988E-05 | 0.00104 | CD |
|  | rs2974224 | 19 | *GLTSCR1* | 4.11E-06 | 0.00227 | 1.558E-05 | 0.000634 | CD |
| 197 | rs10422101 | 19 | *FPR3* | 3.51E-08 | 3.04E-05 | 3.827E-08 | 2.79E-05 | CD |
|  | rs17695224 | 19 | *FPR3* | 9.86E-08 | 8.61E-05 | 2.417E-13 | 7.62E-05 | UC |
| 198 | rs8112458 | 19 | *ZNF614* | 2.76E-05 | 0.0113 | 7.472E-05 | 0.00605 | CD |
| 199 | rs6509859 | 19 | *LILRA6* | 1.71E-05 | 0.0076 | 8.952E-11 | 0.00198 | RA |
|  | rs383369 | 19 | *LILRA6* | 5.85E-13 | 1.53E-07 | 3.699E-12 | 1.49E-07 | RA |
|  | rs798887 | 19 | *LILRA6* | 2.03E-14 | 1.53E-07 | 2.334E-18 | 7.71E-08 | UC |
|  | rs427366 | 19 | *LILRA6* | 1.43E-07 | 0.00013 | 4.364E-11 | 9.07E-05 | T1D |
|  | rs103294 | 19 | *LILRA6* | 4.02E-16 | 1.53E-07 | 3.995E-30 | 8.40E-08 | UC |
|  | rs410852 | 19 | *LILRA6* | 8.47E-13 | 1.53E-07 | 1.894E-22 | 1.35E-07 | UC |
| 200 | rs4806741 | 19 | *LILRA6* | 3.46E-06 | 0.00185 | 1.72E-10 | 0.00138 | RA |
| 201 | rs651279 | 19 | *LILRA5* | 2.22E-13 | 1.53E-07 | 2.191E-12 | 6.90E-08 | CD |
|  | rs741584 | 19 | *LILRA5* | 5.29E-13 | 1.53E-07 | 5.139E-12 | 7.24E-08 | CD |
|  | rs1616661 | 19 | *LILRA5* | 3.42E-11 | 1.53E-07 | 3.106E-10 | 8.09E-08 | CD |
|  | rs1645784 | 19 | *LILRA5* | 2.36E-11 | 1.53E-07 | 6.219E-11 | 1.31E-07 | UC |
| 202 | rs1205340 | 20 | *AHCY* | 3.38E-05 | 0.0138 | 0.0001179 | 0.0023 | UC |
| 203 | rs6098467 | 20 | *KIAA1755* | 1.37E-05 | 0.00623 | 5.83E-05 | 0.00476 | CD |
| 206 | rs6074009 | 20 | *KIAA1637* | 8.64E-06 | 0.00418 | 3.567E-05 | 0.00213 | CD |
|  | rs6065921 | 20 | *NCOA5* | 7.48E-06 | 0.00418 | 7.619E-10 | 0.00213 | CD |
| 208 | rs713875 | 22 | *HORMAD2* | 0.000275 | 0.0723 | 0.000883 | 0.0077 | CD |
| 209 | rs5750784 | 22 | *PDGFB* | 5.03E-06 | 0.00278 | 5.358E-05 | 0.0016 | RA |
|  | rs968451 | 22 | *PDGFB* | 1.00E-05 | 0.00511 | 0.0001789 | 0.000494 | UC |
|  | rs1569501 | 22 | *RPL3* | 1.22E-05 | 0.00623 | 0.0001375 | 0.000535 | UC |
| 3 | rs11580989 | 1 | *MACF1* | 2.98E-05 | 0.0138 | 3.566E-07 | 0.00744 | CD |
|  | rs4660293* | 1 | *PABPC4** | 1.86E-10 | 2.95E-07 | 2.863E-18 | 1.17E-07 | T1D |
| 10 | rs16858559 | 1 | *ZNF648** | 1.24E-06 | 0.000819 | 1.83E-06 | 0.000526 | T1D |
|  | rs1689797 | 1 | *ZNF648** | 4.34E-10 | 5.59E-07 | 2.852E-21 | 3.31E-07 | T1D |
|  | rs1689800* | 1 | *ZNF648** | 1.47E-10 | 2.38E-07 | 4.771E-20 | 2.00E-07 | CD |
|  | rs1779823 | 1 | *ZNF648** | 3.28E-10 | 4.53E-07 | 2.377E-09 | 2.53E-07 | T1D |
| 16 | rs4846908 | 1 | *GALNT2** | 1.89E-09 | 2.47E-06 | 3.236E-09 | 2.07E-06 | UC |
|  | rs2144300 | 1 | *GALNT2** | 1.03E-20 | 1.53E-07 | 3.997E-40 | 8.04E-08 | T1D |
|  | rs2281719 | 1 | *GALNT2** | 2.02E-20 | 1.53E-07 | 1.15E-39 | 7.24E-08 | CD |
|  | rs10779835 | 1 | *GALNT2** | 1.15E-20 | 1.53E-07 | 1.239E-39 | 7.24E-08 | CD |
|  | rs4846918 | 1 | *GALNT2** | 9.73E-14 | 1.53E-07 | 5.518E-12 | 1.22E-07 | CD |
|  | rs2296065 | 1 | *GALNT2** | 1.42E-14 | 1.53E-07 | 9.75E-13 | 1.14E-07 | CD |
| 17 | rs12710745 | 2 | *C2orf43* | 4.75E-06 | 0.00278 | 2.909E-08 | 0.00104 | CD |
|  | rs6711016 | 2 | *C2orf43* | 1.97E-24 | 1.53E-07 | 9.315E-43 | 1.02E-07 | T1D |
|  | rs4341893 | 2 | *APOB** | 1.89E-05 | 0.00927 | 1.788E-07 | 0.00717 | CD |
|  | rs9306897 | 2 | *APOB** | 7.50E-06 | 0.00418 | 2.68E-07 | 0.00346 | T1D |
|  | rs4533439 | 2 | *APOB** | 3.01E-07 | 0.00024 | 2.597E-13 | 0.000178 | CD |
|  | rs13414987 | 2 | *APOB** | 6.52E-06 | 0.00341 | 1.092E-08 | 0.00311 | CD |
|  | rs3923037 | 2 | *APOB** | 2.03E-12 | 1.53E-07 | 1.142E-21 | 9.06E-08 | CD |
|  | rs1344063 | 2 | *APOB** | 2.49E-07 | 0.000196 | 3.575E-12 | 9.61E-05 | UC |
|  | rs17041662 | 2 | *APOB** | 4.07E-09 | 4.67E-06 | 1.248E-15 | 2.77E-06 | UC |
|  | rs6728178 | 2 | *APOB** | 4.84E-29 | 1.53E-07 | 2.091E-47 | 1.22E-07 | CD |
|  | rs10495712 | 2 | *APOB** | 1.32E-05 | 0.00623 | 7.743E-08 | 0.00445 | UC |
|  | rs11902417 | 2 | *APOB** | 1.65E-29 | 1.53E-07 | 1.175E-47 | 1.14E-07 | CD |
|  | rs10172650 | 2 | *APOB** | 1.57E-05 | 0.0076 | 9.393E-08 | 0.00529 | CD |
|  | rs9789416 | 2 | *APOB** | 3.23E-09 | 3.78E-06 | 1.241E-15 | 2.28E-06 | UC |
|  | rs13392272 | 2 | *APOB** | 2.12E-13 | 1.53E-07 | 1.839E-17 | 1.31E-07 | CD |
|  | rs952275 | 2 | *APOB** | 3.25E-14 | 1.53E-07 | 7.493E-21 | 2.03E-07 | RA |
|  | rs533617 | 2 | *APOB** | 2.81E-07 | 0.000196 | 8.91E-21 | 0.00014 | RA |
|  | rs673548 | 2 | *APOB** | 3.34E-31 | 1.53E-07 | 2.116E-53 | 9.38E-08 | T1D |
|  | rs10199768 | 2 | *APOB** | 2.47E-10 | 3.66E-07 | 2.449E-14 | 3.34E-07 | CD |
|  | rs11676704 | 2 | *APOB** | 3.06E-06 | 0.00185 | 8.899E-08 | 0.00115 | UC |
|  | rs1469513 | 2 | *APOB** | 3.99E-08 | 3.75E-05 | 2.181E-12 | 3.09E-05 | RA |
|  | rs12471982 | 2 | *APOB** | 6.35E-05 | 0.0245 | 0.0001548 | 0.00658 | CD |
|  | rs4635554 | 2 | *APOB** | 1.75E-07 | 0.00013 | 5.552E-08 | 0.000124 | RA |
| 23 | rs12328675* | 2 | *COBLL1** | 1.25E-10 | 1.91E-07 | 2.133E-15 | 1.33E-07 | CD |
|  | rs7607980 | 2 | *COBLL1** | 1.81E-10 | 2.95E-07 | 1.807E-15 | 2.02E-07 | CD |
| 27 | rs2943634 | 2 | *BC017935* | 1.16E-09 | 1.61E-06 | 2.022E-16 | 5.57E-07 | T1D |
|  | rs1515100* | 2 | *BC017935* | 9.95E-10 | 1.30E-06 | 8.57E-18 | 8.94E-07 | UC |
|  | rs2713539 | 2 | *BC017935* | 1.23E-09 | 1.61E-06 | 1.605E-17 | 5.71E-07 | T1D |
| 37 | rs151390 | 4 | *SLC39A8** | 5.50E-05 | 0.0203 | 8.887E-08 | 0.00208 | CD |
|  | rs13107325* | 4 | *SLC39A8** | 3.15E-11 | 1.53E-07 | 1.065E-15 | 1.65E-07 | CeD |
|  | rs13114738 | 4 | *SLC39A8** | 6.86E-09 | 7.10E-06 | 2.002E-10 | 1.72E-06 | CD |
| 40 | rs6450176* | 5 | *ARL15** | 2.78E-08 | 2.47E-05 | 6.875E-10 | 7.65E-06 | UC |
|  | rs4311394 | 5 | *ARL15** | 7.32E-08 | 7.01E-05 | 4.968E-09 | 2.04E-05 | UC |
| 48 | rs11758426 | 6 | *PACSIN1* | 7.90E-07 | 0.000543 | 6.467E-10 | 0.00033 | RA |
|  | rs2814944* | 6 | *C6orf106** | 1.93E-09 | 2.47E-06 | 3.952E-13 | 1.88E-06 | RA |
|  | rs2744937 | 6 | *C6orf106** | 6.58E-09 | 7.10E-06 | 1.821E-12 | 2.18E-06 | CD |
|  | rs205262 | 6 | *C6orf106** | 1.49E-08 | 1.62E-05 | 3.878E-13 | 8.65E-06 | CD |
|  | rs2744972 | 6 | *C6orf106** | 6.64E-09 | 7.10E-06 | 3.253E-08 | 2.31E-06 | CD |
|  | rs12204265 | 6 | *ANKS1A* | 5.17E-05 | 0.0203 | 0.001057 | 0.00619 | UC |
|  | rs7742443 | 6 | *ANKS1A* | 4.18E-06 | 0.00227 | 1.346E-05 | 0.00267 | RA |
| 53 | rs605066* | 6 | *AK123801* | 1.39E-08 | 1.32E-05 | 2.786E-08 | 1.63E-05 | RA |
|  | rs634869 | 6 | *AK123801* | 2.75E-08 | 2.47E-05 | 1.003E-10 | 1.22E-05 | CD |
|  | rs668459 | 6 | *AK123801* | 5.77E-08 | 5.70E-05 | 1.423E-10 | 2.94E-05 | CD |
|  | rs628751 | 6 | *AK123801* | 2.36E-08 | 2.47E-05 | 2.538E-10 | 1.38E-05 | CD |
| 57 | rs9355296 | 6 | *LPA** | 2.03E-07 | 0.00016 | 7.041E-08 | 8.87E-05 | UC |
|  | rs1367211 | 6 | *LPA** | 7.37E-06 | 0.00418 | 2.892E-06 | 0.00248 | UC |
|  | rs783149 | 6 | *LPA** | 5.69E-08 | 5.70E-05 | 2.946E-11 | 4.79E-05 | T1D |
|  | rs1084651* | 6 | *LPA** | 1.63E-08 | 1.62E-05 | 1.347E-11 | 2.00E-05 | RA |
| 63 | rs714052 | 7 | *BAZ1B* | 2.02E-09 | 2.47E-06 | 2.212E-12 | 7.30E-07 | CD |
|  | rs13244268 | 7 | *BAZ1B* | 6.57E-10 | 8.50E-07 | 8.079E-09 | 1.07E-06 | RA |
|  | rs17145738* | 7 | *TBL2* | 5.77E-10 | 8.50E-07 | 4.952E-13 | 2.95E-07 | CD |
| 68 | rs13234269 | 7 | *KLF14** | 1.07E-15 | 1.53E-07 | 8.876E-17 | 1.22E-07 | CD |
|  | rs1596972 | 7 | *KLF14** | 1.30E-13 | 1.53E-07 | 2.064E-13 | 1.41E-07 | CD |
|  | rs10954284 | 7 | *KLF14** | 1.02E-15 | 1.53E-07 | 5.804E-17 | 1.14E-07 | CD |
| 73 | rs4921667 | 8 | *CSGALNACT1* | 8.61E-09 | 8.72E-06 | 6.764E-08 | 1.09E-05 | RA |
|  | rs1967103 | 8 | *CSGALNACT1* | 2.27E-08 | 2.47E-05 | 1.708E-07 | 1.81E-05 | UC |
|  | rs1907800 | 8 | *CSGALNACT1* | 9.48E-06 | 0.00511 | 2.702E-05 | 0.00387 | CD |
|  | rs10085966 | 8 | *CSGALNACT1* | 1.77E-07 | 0.00013 | 9.799E-09 | 0.000107 | CD |
|  | rs7010610 | 8 | *CSGALNACT1* | 1.11E-05 | 0.00511 | 2.674E-12 | 0.00506 | PSOR |
|  | rs4551376 | 8 | *CSGALNACT1* | 5.23E-06 | 0.00278 | 1.689E-05 | 0.00278 | CD |
|  | rs11204072 | 8 | *INTS10* | 8.30E-09 | 8.72E-06 | 1.281E-13 | 4.19E-06 | CD |
|  | rs6586872 | 8 | *INTS10* | 3.62E-11 | 1.53E-07 | 4.76E-21 | 1.39E-07 | UC |
|  | rs11986182 | 8 | *INTS10* | 1.13E-21 | 1.53E-07 | 4.36E-21 | 2.08E-07 | RA |
|  | rs7821631 | 8 | *INTS10* | 1.33E-06 | 0.000819 | 3.89E-06 | 0.000543 | UC |
|  | rs4922113 | 8 | *INTS10* | 1.47E-21 | 1.53E-07 | 5.317E-37 | 1.08E-07 | CD |
|  | rs6586876 | 8 | *INTS10* | 1.94E-09 | 2.47E-06 | 1.002E-12 | 1.56E-06 | CD |
|  | rs17410407 | 8 | *INTS10* | 1.45E-21 | 1.53E-07 | 9.315E-31 | 1.29E-07 | UC |
|  | rs1866956 | 8 | *INTS10* | 2.32E-08 | 2.47E-05 | 7.964E-10 | 1.64E-05 | T1D |
|  | rs3898938 | 8 | *INTS10* | 1.54E-08 | 1.62E-05 | 7.814E-13 | 5.17E-06 | T1D |
|  | rs1441773 | 8 | *INTS10* | 9.90E-08 | 8.61E-05 | 7.38E-09 | 7.34E-05 | T1D |
|  | rs1441771 | 8 | *LPL** | 9.98E-25 | 1.53E-07 | 2.326E-46 | 9.92E-08 | CD |
|  | rs1441770 | 8 | *LPL** | 1.89E-13 | 1.53E-07 | 8.058E-20 | 1.41E-07 | CD |
|  | rs6586879 | 8 | *LPL** | 9.54E-21 | 1.53E-07 | 6.774E-33 | 1.31E-07 | CD |
|  | rs6996383 | 8 | *LPL** | 1.48E-22 | 1.53E-07 | 5.139E-19 | 1.31E-07 | CD |
|  | rs3988302 | 8 | *LPL** | 3.68E-22 | 1.53E-07 | 4.366E-31 | 1.31E-07 | CD |
|  | rs3900537 | 8 | *LPL** | 5.34E-21 | 1.53E-07 | 2.459E-27 | 1.31E-07 | CD |
|  | rs7000460 | 8 | *LPL** | 3.86E-23 | 1.53E-07 | 1.313E-31 | 1.31E-07 | CD |
|  | rs253 | 8 | *LPL** | 6.31E-18 | 1.53E-07 | 8.139E-30 | 1.41E-07 | CD |
|  | rs263 | 8 | *LPL** | 2.42E-43 | 1.53E-07 | 5.858E-81 | 1.14E-07 | CD |
|  | rs264 | 8 | *LPL** | 1.23E-49 | 1.53E-07 | 8.033E-77 | 1.14E-07 | CD |
|  | rs268 | 8 | *LPL** | 7.29E-14 | 1.53E-07 | 1.625E-12 | 1.28E-07 | UC |
|  | rs285 | 8 | *LPL** | 5.69E-36 | 1.53E-07 | 4.999E-48 | 2.03E-07 | RA |
|  | rs312 | 8 | *LPL** | 3.12E-13 | 1.53E-07 | 8.32E-19 | 2.03E-07 | RA |
|  | rs316 | 8 | *LPL** | 4.74E-12 | 1.53E-07 | 4.422E-11 | 1.31E-07 | CD |
|  | rs331 | 8 | *LPL** | 2.12E-97 | 1.53E-07 | 3.95E-150 | 9.38E-08 | T1D |
|  | rs3289 | 8 | *LPL** | 2.09E-28 | 1.53E-07 | 6.444E-46 | 1.39E-07 | UC |
|  | rs9644636 | 8 | *LPL** | 2.12E-19 | 1.53E-07 | 8.375E-27 | 1.31E-07 | CD |
|  | rs17482753 | 8 | *LPL** | 7.22E-98 | 1.53E-07 | 2.8E-149 | 6.11E-08 | T1D |
|  | rs12678919* | 8 | *LPL** | 2.16E-101 | 1.53E-07 | 1.38E-149 | 6.11E-08 | T1D |
|  | rs17489268 | 8 | *LPL** | 1.85E-90 | 1.53E-07 | 2.54E-137 | 9.51E-08 | CD |
|  | rs1372345 | 8 | *LPL** | 1.51E-13 | 1.53E-07 | 1.123E-24 | 1.22E-07 | CD |
|  | rs11992311 | 8 | *LPL** | 1.20E-13 | 1.53E-07 | 1.898E-16 | 1.12E-07 | T1D |
|  | rs1441766 | 8 | *LPL** | 9.35E-57 | 1.53E-07 | 6.794E-52 | 8.58E-08 | CD |
|  | rs1899351 | 8 | *LPL** | 6.00E-10 | 8.50E-07 | 4.024E-18 | 4.26E-07 | UC |
|  | rs1441759 | 8 | *LPL** | 2.05E-10 | 2.95E-07 | 2.425E-16 | 3.79E-07 | RA |
|  | rs894210 | 8 | *LPL** | 1.24E-55 | 1.53E-07 | 1.683E-84 | 1.22E-07 | CD |
|  | rs7005359 | 8 | *LPL** | 1.73E-33 | 1.53E-07 | 1.218E-40 | 9.51E-08 | CD |
|  | rs2410629 | 8 | *LPL** | 9.37E-32 | 1.53E-07 | 3.511E-49 | 9.38E-08 | T1D |
|  | rs1534650 | 8 | *LPL** | 1.61E-20 | 1.53E-07 | 8.125E-31 | 1.24E-07 | T1D |
|  | rs10103634 | 8 | *LPL** | 1.89E-29 | 1.53E-07 | 3.647E-38 | 1.02E-07 | T1D |
|  | rs4557718 | 8 | *LPL** | 1.28E-13 | 1.53E-07 | 5.738E-18 | 2.03E-07 | RA |
|  | rs4244457 | 8 | *LPL** | 1.16E-24 | 1.53E-07 | 1.094E-43 | 1.22E-07 | CD |
|  | rs6586891 | 8 | *SLC18A1* | 1.37E-41 | 1.53E-07 | 4.061E-61 | 7.51E-08 | T1D |
|  | rs4128744 | 8 | *SLC18A1* | 2.90E-71 | 1.53E-07 | 1.031E-64 | 9.44E-08 | T1D |
| 78 | rs11989423 | 8 | *TRPS1** | 7.49E-11 | 1.53E-07 | 4.054E-10 | 2.03E-07 | RA |
|  | rs13271228 | 8 | *TRPS1** | 8.67E-11 | 1.53E-07 | 5.229E-10 | 9.14E-08 | UC |
|  | rs2293889* | 8 | *TRPS1** | 2.50E-11 | 1.53E-07 | 4.271E-17 | 4.07E-08 | UC |
|  | rs7357472 | 8 | *TRPS1** | 9.70E-11 | 1.53E-07 | 4.97E-10 | 2.80E-08 | UC |
|  | rs2737203 | 8 | *TRPS1** | 3.37E-11 | 1.53E-07 | 1.786E-10 | 9.14E-08 | UC |
|  | rs2737205 | 8 | *TRPS1** | 1.17E-10 | 1.91E-07 | 6.296E-10 | 1.84E-07 | UC |
| 80 | rs2385114 | 8 | *TRIB1** | 7.91E-06 | 0.00418 | 1.127E-06 | 0.00138 | CD |
|  | rs2980856 | 8 | *TRIB1** | 5.74E-17 | 1.53E-07 | 9.698E-28 | 1.03E-07 | CD |
|  | rs2954018 | 8 | *TRIB1** | 6.77E-14 | 1.53E-07 | 4.805E-27 | 5.73E-08 | CD |
|  | rs2001845 | 8 | *TRIB1** | 1.57E-14 | 1.53E-07 | 1.646E-27 | 6.33E-08 | CD |
|  | rs2954020 | 8 | *TRIB1** | 1.13E-12 | 1.53E-07 | 2.973E-24 | 6.90E-08 | CD |
|  | rs2980862 | 8 | *TRIB1** | 7.27E-15 | 1.53E-07 | 6.56E-27 | 6.61E-08 | CD |
|  | rs2954032 | 8 | *TRIB1** | 1.30E-14 | 1.53E-07 | 1.089E-27 | 6.33E-08 | CD |
|  | rs10808546* | 8 | *TRIB1** | 1.38E-19 | 1.53E-07 | 4.106E-30 | 8.09E-08 | CD |
| 81 | rs7832357 | 8 | *TRIB1** | 2.11E-05 | 0.00927 | 5.492E-05 | 0.00409 | UC |
|  | rs4512391 | 8 | *TRIB1** | 2.54E-05 | 0.0113 | 4.151E-08 | 0.00643 | CD |
|  | rs4360309 | 8 | *TRIB1** | 1.40E-05 | 0.00623 | 4.787E-08 | 0.0073 | RA |
| 82 | rs2124036 | 8 | *TRIB1** | 4.48E-06 | 0.00278 | 1.681E-06 | 0.00218 | PSOR |
| 84 | rs3933785 | 9 | *TTC39B** | 9.33E-12 | 1.53E-07 | 4.127E-21 | 1.08E-07 | CD |
|  | rs10810369 | 9 | *TTC39B** | 4.70E-13 | 1.53E-07 | 6.05E-18 | 1.24E-07 | T1D |
|  | rs471364 | 9 | *TTC39B** | 1.85E-11 | 1.53E-07 | 1.839E-20 | 1.14E-07 | CD |
|  | rs585002 | 9 | *TTC39B** | 7.81E-12 | 1.53E-07 | 1.048E-19 | 1.67E-07 | UC |
|  | rs2849049 | 9 | *TTC39B** | 4.29E-06 | 0.00227 | 1.478E-07 | 0.0014 | CD |
| 85 | rs2777799 | 9 | *ABCA1** | 4.75E-07 | 0.00036 | 1.625E-18 | 0.000125 | RA |
|  | rs2777800 | 9 | *ABCA1** | 1.33E-07 | 0.000106 | 3.066E-07 | 2.08E-05 | RA |
|  | rs2066716 | 9 | *ABCA1** | 8.02E-06 | 0.00418 | 3.274E-05 | 0.00257 | RA |
|  | rs2740476 | 9 | *ABCA1** | 9.88E-06 | 0.00511 | 2.413E-05 | 0.00158 | RA |
| 86 | rs3780543 | 9 | *ABCA1** | 8.65E-11 | 1.53E-07 | 1.419E-19 | 6.04E-08 | CD |
|  | rs4743762 | 9 | *ABCA1** | 3.67E-11 | 1.53E-07 | 1.595E-10 | 5.73E-08 | CD |
|  | rs4149281 | 9 | *ABCA1** | 1.39E-10 | 1.91E-07 | 4.552E-12 | 1.86E-07 | T1D |
| 87 | rs4743764 | 9 | *ABCA1** | 3.75E-17 | 1.53E-07 | 1.603E-16 | 8.40E-08 | UC |
|  | rs2000069 | 9 | *ABCA1** | 2.24E-14 | 1.53E-07 | 5.593E-12 | 5.90E-08 | T1D |
|  | rs4149274 | 9 | *ABCA1** | 2.10E-13 | 1.53E-07 | 1.833E-23 | 1.63E-07 | CeD |
|  | rs11789603 | 9 | *ABCA1** | 4.38E-15 | 1.53E-07 | 3.695E-21 | 2.08E-07 | RA |
|  | rs3890182 | 9 | *ABCA1** | 5.89E-34 | 1.53E-07 | 6.128E-58 | 7.04E-08 | T1D |
|  | rs3847303 | 9 | *ABCA1** | 7.99E-34 | 1.53E-07 | 9.483E-58 | 7.04E-08 | T1D |
|  | rs2275543 | 9 | *ABCA1** | 2.05E-33 | 1.53E-07 | 5.307E-53 | 1.94E-07 | RA |
|  | rs12686004 | 9 | *ABCA1** | 8.89E-11 | 1.53E-07 | 1.992E-18 | 1.12E-07 | T1D |
|  | rs3905000 | 9 | *ABCA1** | 4.10E-33 | 1.53E-07 | 8.673E-57 | 6.11E-08 | T1D |
|  | rs3847305 | 9 | *ABCA1** | 8.75E-27 | 1.53E-07 | 2.158E-53 | 9.51E-08 | CD |
|  | rs2777784 | 9 | *ABCA1** | 9.06E-16 | 1.53E-07 | 1.793E-31 | 1.49E-07 | RA |
|  | rs2777793 | 9 | *ABCA1** | 1.23E-22 | 1.53E-07 | 7.624E-37 | 1.14E-07 | CD |
|  | rs2482424 | 9 | *ABCA1** | 6.05E-06 | 0.00341 | 9.926E-09 | 0.00357 | PSOR |
|  | rs2740486 | 9 | *ABCA1** | 6.13E-24 | 1.53E-07 | 7.04E-38 | 1.14E-07 | CD |
|  | rs3905001 | 9 | *ABCA1** | 8.57E-11 | 1.53E-07 | 4.37E-12 | 1.77E-07 | CeD |
| 88 | rs4100654 | 9 | *ABCA1** | 4.23E-28 | 1.53E-07 | 1.757E-25 | 7.71E-08 | UC |
| 89 | rs2515618 | 9 | *ABCA1** | 3.03E-06 | 0.00185 | 3.606E-06 | 0.00128 | RA |
|  | rs2515616 | 9 | *ABCA1** | 4.99E-07 | 0.00036 | 1.386E-06 | 0.000342 | PSOR |
|  | rs2472508 | 9 | *ABCA1** | 1.27E-07 | 0.000106 | 1.727E-05 | 9.40E-05 | RA |
|  | rs1800977 | 9 | *AK311445* | 1.76E-07 | 0.00013 | 1.308E-07 | 8.56E-05 | UC |
| 96 | rs2923084* | 11 | *EF537580* | 2.57E-08 | 2.47E-05 | 5.02E-08 | 1.22E-05 | CD |
| 103 | rs108499 | 11 | *C11orf9* | 3.75E-17 | 1.53E-07 | 4.849E-17 | 1.96E-08 | CD |
|  | rs509360 | 11 | *C11orf9* | 2.92E-10 | 4.53E-07 | 1.84E-09 | 1.05E-07 | CD |
|  | rs174535 | 11 | *C11orf9* | 1.59E-22 | 1.53E-07 | 9.036E-28 | 1.96E-08 | CD |
|  | rs102275 | 11 | *C11orf10* | 3.63E-23 | 1.53E-07 | 6.404E-28 | 1.96E-08 | CD |
|  | rs174538 | 11 | *C11orf10* | 2.84E-19 | 1.53E-07 | 7.897E-20 | 1.01E-07 | RA |
|  | rs174556 | 11 | *FADS1** | 1.12E-20 | 1.53E-07 | 9.647E-23 | 1.96E-08 | CD |
|  | rs174570 | 11 | *FADS2** | 5.87E-15 | 1.53E-07 | 2.414E-17 | 2.86E-08 | CD |
|  | rs2845573 | 11 | *FADS2** | 2.12E-12 | 1.53E-07 | 3.448E-11 | 6.61E-08 | CD |
|  | rs2727270 | 11 | *FADS2** | 1.80E-14 | 1.53E-07 | 3.476E-14 | 1.74E-07 | RA |
|  | rs2072114 | 11 | *FADS2** | 3.89E-15 | 1.53E-07 | 1.441E-16 | 4.20E-08 | CD |
|  | rs174589 | 11 | *FADS2** | 2.74E-07 | 0.000196 | 5.665E-07 | 2.45E-05 | CD |
|  | rs2851682 | 11 | *FADS2** | 3.52E-13 | 1.53E-07 | 2.762E-09 | 8.09E-08 | CD |
|  | rs174602 | 11 | *FADS2** | 1.42E-06 | 0.00101 | 5.722E-11 | 0.000866 | PSOR |
|  | rs174449 | 11 | *FADS3** | 4.13E-15 | 1.53E-07 | 4.997E-15 | 3.83E-08 | CD |
|  | rs7942717 | 11 | *FADS3** | 4.67E-07 | 0.00036 | 1.283E-08 | 0.000102 | CD |
|  | rs174634 | 11 | *FADS3** | 7.78E-09 | 8.72E-06 | 1.075E-08 | 2.06E-06 | RA |
|  | rs1000778 | 11 | *FADS3** | 8.10E-09 | 8.72E-06 | 7.905E-08 | 2.17E-06 | RA |
|  | rs174455 | 11 | *FADS3** | 4.38E-12 | 1.53E-07 | 2.163E-11 | 4.20E-08 | CD |
| 106 | rs480823 | 11 | *BUD13* | 2.06E-09 | 2.47E-06 | 9.605E-21 | 1.69E-06 | UC |
|  | rs17119878 | 11 | *BUD13* | 7.53E-16 | 1.53E-07 | 8.916E-19 | 1.14E-07 | CD |
|  | rs1145189 | 11 | *BUD13* | 1.27E-11 | 1.53E-07 | 2.58E-11 | 8.66E-08 | T1D |
|  | rs12805061 | 11 | *BUD13* | 2.22E-12 | 1.53E-07 | 4.128E-20 | 1.28E-07 | UC |
|  | rs1942479 | 11 | *BUD13* | 2.97E-12 | 1.53E-07 | 3.803E-14 | 1.39E-07 | UC |
|  | rs1892948 | 11 | *BUD13* | 1.02E-16 | 1.53E-07 | 1.95E-16 | 1.41E-07 | CD |
|  | rs10892019 | 11 | *BUD13* | 5.72E-15 | 1.53E-07 | 2.232E-14 | 1.74E-07 | RA |
|  | rs7350481 | 11 | *BUD13* | 2.83E-19 | 1.53E-07 | 3.826E-36 | 1.31E-07 | CD |
|  | rs180360 | 11 | *BUD13* | 4.16E-21 | 1.53E-07 | 5.737E-32 | 1.24E-07 | T1D |
|  | rs10466588 | 11 | *BUD13* | 2.32E-20 | 1.53E-07 | 5.675E-34 | 1.31E-07 | CD |
|  | rs918144 | 11 | *BUD13* | 4.82E-07 | 0.00036 | 6.465E-09 | 0.00032 | UC |
|  | rs10488698 | 11 | *BUD13* | 1.38E-07 | 0.000106 | 1.405E-15 | 4.08E-05 | UC |
|  | rs2187126 | 11 | *BUD13* | 7.29E-08 | 7.01E-05 | 2.221E-15 | 5.98E-05 | RA |
|  | rs6589565 | 11 | *BUD13* | 3.69E-22 | 1.53E-07 | 1.219E-21 | 1.43E-07 | RA |
|  | rs964184* | 11 | *ZNF259* | 9.82E-49 | 1.53E-07 | 6.086E-48 | 1.31E-07 | CD |
|  | rs6589566 | 11 | *ZNF259* | 2.45E-17 | 1.53E-07 | 2.715E-38 | 1.02E-07 | T1D |
|  | rs618923 | 11 | *ZNF259* | 5.12E-19 | 1.53E-07 | 2.737E-21 | 1.08E-07 | CD |
|  | rs2266788 | 11 | *APOA5** | 7.42E-17 | 1.53E-07 | 1.185E-35 | 1.31E-07 | CD;UC |
|  | rs6589567 | 11 | *APOA5** | 1.18E-05 | 0.00623 | 2.181E-09 | 0.00361 | T1D |
|  | rs1729409 | 11 | *APOA5** | 1.54E-08 | 1.62E-05 | 2.823E-08 | 1.49E-05 | CD |
|  | rs625145 | 11 | *SIK3* | 4.17E-17 | 1.53E-07 | 5.921E-16 | 1.31E-07 | CD |
|  | rs11216162 | 11 | *SIK3* | 2.85E-16 | 1.53E-07 | 3.046E-25 | 6.31E-08 | T1D |
|  | rs11216164 | 11 | *SIK3* | 1.09E-10 | 1.53E-07 | 1.156E-09 | 1.50E-07 | UC |
|  | rs7130160 | 11 | *SIK3* | 8.32E-10 | 1.05E-06 | 2.318E-09 | 7.18E-07 | T1D |
|  | rs10502221 | 11 | *SIK3* | 6.28E-09 | 7.10E-06 | 1.379E-08 | 2.93E-06 | T1D |
|  | rs7938466 | 11 | *SIK3* | 1.60E-09 | 1.99E-06 | 4.469E-09 | 2.33E-06 | CeD |
|  | rs7943171 | 11 | *SIK3* | 2.78E-09 | 3.06E-06 | 7.595E-09 | 1.18E-06 | CD |
|  | rs17120244 | 11 | *SIK3* | 9.11E-16 | 1.53E-07 | 1.172E-21 | 1.39E-07 | UC |
|  | rs1871756 | 11 | *SIK3* | 3.68E-12 | 1.53E-07 | 2.882E-13 | 6.63E-08 | T1D |
|  | rs4938343 | 11 | *AB231710* | 2.99E-08 | 3.04E-05 | 5.766E-07 | 1.45E-05 | RA |
|  | rs12291885 | 11 | *PAFAH1B2* | 6.44E-10 | 8.50E-07 | 2.402E-09 | 1.09E-06 | RA |
|  | rs1871757 | 11 | *PAFAH1B2* | 7.41E-07 | 0.000543 | 8.519E-06 | 0.000214 | RA |
|  | rs7112577 | 11 | *PAFAH1B2* | 4.72E-11 | 1.53E-07 | 2.34E-10 | 1.22E-07 | CD |
|  | rs4938353 | 11 | *PAFAH1B2* | 1.06E-06 | 0.000666 | 7.77E-11 | 2.00E-04 | T1D |
| 109 | rs7941030 | 11 | *UBASH3B** | 1.45E-08 | 1.62E-05 | 1.12E-14 | 8.65E-06 | CD |
|  | rs7127978 | 11 | *UBASH3B** | 2.22E-08 | 2.00E-05 | 3.507E-07 | 6.65E-06 | T1D |
|  | rs7117842 | 11 | *UBASH3B** | 1.52E-08 | 1.62E-05 | 1.057E-14 | 8.09E-06 | CD |
|  | rs10892873 | 11 | *UBASH3B** | 2.09E-08 | 2.00E-05 | 3.307E-07 | 6.65E-06 | T1D |
|  | rs7117189 | 11 | *UBASH3B** | 2.02E-05 | 0.00927 | 9.432E-05 | 0.00459 | CD |
|  | rs12270851 | 11 | *UBASH3B** | 3.98E-06 | 0.00227 | 2.474E-05 | 0.00271 | RA |
|  | rs11218749 | 11 | *UBASH3B** | 1.26E-05 | 0.00623 | 5.836E-05 | 0.00302 | CD |
| 110 | rs7134375* | 12 | *PDE3A** | 2.12E-08 | 2.00E-05 | 1.045E-08 | 1.78E-05 | UC |
| 111 | rs7978567 | 12 | *LRP1** | 0.000105 | 0.0353 | 0.0005654 | 0.00864 | UC |
|  | rs567895 | 12 | *R3HDM2* | 2.12E-05 | 0.00927 | 1.426E-05 | 0.000796 | UC |
|  | rs3741414* | 12 | *INHBC* | 8.76E-09 | 8.72E-06 | 6.095E-14 | 4.76E-06 | UC |
|  | rs473465 | 12 | *INHBE* | 1.39E-05 | 0.00623 | 9.715E-08 | 0.000535 | UC |
| 114 | rs1992136 | 12 | *FOXN4* | 2.51E-05 | 0.0113 | 4.913E-05 | 0.00499 | UC |
|  | rs12582463 | 12 | *FOXN4* | 3.25E-09 | 3.78E-06 | 1.532E-06 | 2.51E-06 | UC |
|  | rs10850058 | 12 | *FOXN4* | 6.08E-09 | 7.10E-06 | 1.777E-06 | 4.51E-06 | UC |
|  | rs7968387 | 12 | *MYO1H* | 8.52E-09 | 8.72E-06 | 3.421E-06 | 6.18E-06 | UC |
|  | rs9943753 | 12 | *MYO1H* | 4.88E-12 | 1.53E-07 | 1.311E-11 | 1.35E-07 | UC |
|  | rs6663 | 12 | *KCTD10* | 2.71E-07 | 0.000196 | 1.026E-05 | 0.000214 | PSOR |
|  | rs888193 | 12 | *MMAB* | 6.43E-09 | 7.10E-06 | 1.438E-08 | 4.51E-06 | UC |
|  | rs11067231 | 12 | *MMAB* | 4.27E-15 | 1.53E-07 | 2.16E-19 | 1.51E-07 | PSOR |
|  | rs10850379 | 12 | *MMAB* | 1.18E-13 | 1.53E-07 | 4.045E-14 | 1.33E-07 | UC |
|  | rs12314392 | 12 | *MMAB* | 1.43E-13 | 1.53E-07 | 4.264E-13 | 1.57E-07 | PSOR |
|  | rs3759387 | 12 | *MVK** | 1.80E-08 | 2.00E-05 | 3.066E-08 | 1.93E-05 | PSOR |
|  | rs11067376 | 12 | *MVK** | 1.67E-12 | 1.53E-07 | 6.031E-11 | 1.56E-07 | RA |
|  | rs10161126 | 12 | *MVK** | 3.05E-13 | 1.53E-07 | 1.441E-16 | 1.52E-07 | PSOR |
|  | rs1861679 | 12 | *MVK** | 1.04E-06 | 0.000666 | 3.769E-08 | 0.000318 | T1D |
|  | rs10774803 | 12 | *MVK** | 2.01E-08 | 2.00E-05 | 7.16E-08 | 2.65E-05 | RA |
| 120 | rs10847980 | 12 | *VPS37B* | 8.75E-05 | 0.0295 | 3.158E-07 | 0.00976 | RA |
|  | rs10773003 | 12 | *mop-3* | 4.59E-07 | 0.00036 | 1.455E-13 | 0.000189 | T1D |
|  | rs4759375* | 12 | *SBNO1** | 3.90E-09 | 4.67E-06 | 3.011E-08 | 5.94E-06 | RA |
|  | rs10846513 | 12 | *SBNO1** | 6.06E-07 | 0.000442 | 6.435E-11 | 0.000325 | CD |
| 121 | rs4930732 | 12 | *DNAH10* | 1.26E-05 | 0.00623 | 1.84E-06 | 0.00523 | CD |
|  | rs4930731 | 12 | *DNAH10* | 2.81E-06 | 0.00151 | 7.027E-07 | 0.00113 | CD |
|  | rs7973103 | 12 | *limkain* | 1.24E-06 | 0.000819 | 9.251E-06 | 0.000543 | UC |
|  | rs7973683 | 12 | *CCDC92* | 1.60E-10 | 2.38E-07 | 5.259E-14 | 1.33E-07 | CD |
|  | rs4765127* | 12 | *ZNF664** | 1.33E-10 | 1.91E-07 | 7.792E-10 | 1.00E-07 | CD |
| 122 | rs863750 | 12 | *ZNF664** | 3.72E-10 | 5.59E-07 | 4.714E-13 | 4.68E-07 | CD |
| 123 | rs838873 | 12 | *SCARB1** | 1.13E-06 | 0.000819 | 1.04E-06 | 0.000325 | RA |
| 124 | rs838876 | 12 | *SCARB1** | 4.62E-14 | 1.53E-07 | 7.325E-33 | 2.11E-07 | RA |
|  | rs838878 | 12 | *SCARB1** | 2.17E-14 | 1.53E-07 | 3.963E-30 | 1.22E-07 | CD |
|  | rs838882 | 12 | *SCARB1** | 2.03E-14 | 1.53E-07 | 1.631E-32 | 1.31E-07 | CD |
| 125 | rs10773105 | 12 | *SCARB1** | 4.49E-09 | 5.76E-06 | 3.202E-24 | 6.83E-06 | T1D |
|  | rs989892 | 12 | *SCARB1** | 1.64E-08 | 1.62E-05 | 2.251E-21 | 8.65E-06 | CD |
|  | rs3782287 | 12 | *SCARB1** | 8.67E-08 | 7.01E-05 | 3.634E-07 | 3.60E-05 | CD |
| 126 | rs7137797 | 12 | *SCARB1** | 5.54E-11 | 1.53E-07 | 1.078E-10 | 1.22E-07 | CD |
|  | rs3924313 | 12 | *SCARB1** | 3.05E-12 | 1.53E-07 | 1.747E-11 | 1.31E-07 | CD |
| 137 | rs4775031 | 15 | *ALDH1A2* | 2.16E-10 | 2.95E-07 | 2.297E-13 | 2.47E-07 | CD |
|  | rs4622454 | 15 | *LIPC** | 3.77E-11 | 1.53E-07 | 1.932E-19 | 7.05E-08 | UC |
|  | rs16940126 | 15 | *LIPC** | 5.44E-11 | 1.53E-07 | 2.226E-18 | 2.11E-07 | RA |
| 138 | rs473422 | 15 | *LIPC** | 2.53E-11 | 1.53E-07 | 2.627E-18 | 8.58E-08 | CD |
| 139 | rs4775039 | 15 | *LIPC** | 5.36E-35 | 1.53E-07 | 1.67E-33 | 2.08E-07 | RA |
|  | rs12908474 | 15 | *LIPC** | 3.67E-61 | 1.53E-07 | 1.007E-55 | 1.51E-07 | PSOR |
|  | rs4775041 | 15 | *LIPC** | 7.44E-83 | 1.53E-07 | 4.92E-147 | 1.23E-07 | UC |
|  | rs16940147 | 15 | *LIPC** | 4.13E-08 | 3.75E-05 | 2.447E-10 | 2.74E-05 | UC |
|  | rs10468017 | 15 | *LIPC** | 1.32E-96 | 1.53E-07 | 1.21E-188 | 9.38E-08 | T1D |
|  | rs1825955 | 15 | *LIPC** | 2.11E-24 | 1.53E-07 | 2.593E-34 | 2.03E-07 | RA |
|  | rs1532085* | 15 | *LIPC** | 7.39E-100 | 1.53E-07 | 1.24E-188 | 1.37E-07 | T1D |
|  | rs12185072 | 15 | *LIPC** | 7.55E-30 | 1.53E-07 | 6.887E-44 | 1.41E-07 | CD |
|  | rs16940167 | 15 | *LIPC** | 5.39E-30 | 1.53E-07 | 1.097E-44 | 1.41E-07 | CD |
|  | rs16940170 | 15 | *LIPC** | 1.29E-25 | 1.53E-07 | 6.465E-46 | 1.41E-07 | CD |
|  | rs17821274 | 15 | *LIPC** | 4.76E-28 | 1.53E-07 | 2.407E-50 | 1.12E-07 | T1D |
|  | rs8042174 | 15 | *LIPC** | 2.82E-12 | 1.53E-07 | 7.299E-12 | 2.03E-07 | RA |
|  | rs7164909 | 15 | *LIPC** | 3.09E-13 | 1.53E-07 | 5.058E-24 | 1.33E-07 | UC |
|  | rs7165077 | 15 | *LIPC** | 1.75E-10 | 2.38E-07 | 2.224E-23 | 2.18E-07 | UC |
|  | rs11855284 | 15 | *LIPC** | 2.97E-47 | 1.53E-07 | 3.02E-95 | 9.38E-08 | T1D |
|  | rs415799 | 15 | *LIPC** | 7.70E-66 | 1.53E-07 | 2.718E-63 | 1.22E-07 | CD |
|  | rs440183 | 15 | *LIPC** | 2.91E-11 | 1.53E-07 | 8.599E-13 | 1.33E-07 | UC |
|  | rs16940204 | 15 | *LIPC** | 8.84E-46 | 1.53E-07 | 1.213E-43 | 1.41E-07 | CD |
|  | rs487766 | 15 | *LIPC** | 1.90E-47 | 1.53E-07 | 1.87E-93 | 8.66E-08 | T1D |
|  | rs16940212 | 15 | *LIPC** | 8.34E-13 | 1.53E-07 | 6.079E-21 | 1.02E-07 | T1D |
|  | rs16940213 | 15 | *LIPC** | 4.69E-15 | 1.53E-07 | 3.681E-29 | 8.66E-08 | T1D |
|  | rs12440032 | 15 | *LIPC** | 2.88E-41 | 1.53E-07 | 2.884E-39 | 1.43E-07 | RA |
|  | rs11071380 | 15 | *LIPC** | 3.91E-44 | 1.53E-07 | 4.092E-86 | 1.45E-07 | RA |
|  | rs436965 | 15 | *LIPC** | 7.72E-11 | 1.53E-07 | 3.257E-24 | 1.24E-07 | T1D |
| 140 | rs7163052 | 15 | *LIPC** | 1.68E-17 | 1.53E-07 | 2.038E-16 | 1.46E-07 | RA |
|  | rs4774297 | 15 | *LIPC** | 1.08E-21 | 1.53E-07 | 3.64E-20 | 1.31E-07 | CD |
|  | rs11632618 | 15 | *LIPC** | 5.66E-32 | 1.53E-07 | 3.459E-29 | 1.39E-07 | UC |
|  | rs633695 | 15 | *LIPC** | 1.52E-62 | 1.53E-07 | 7.82E-58 | 1.37E-07 | T1D |
|  | rs17190510 | 15 | *LIPC** | 8.73E-11 | 1.53E-07 | 7.27E-10 | 1.30E-07 | RA |
|  | rs261333 | 15 | *LIPC** | 8.36E-28 | 1.53E-07 | 2.822E-26 | 1.31E-07 | CD |
|  | rs17190517 | 15 | *LIPC** | 1.14E-26 | 1.53E-07 | 3.651E-25 | 1.43E-07 | RA |
|  | rs12914035 | 15 | *LIPC** | 1.45E-11 | 1.53E-07 | 3.863E-22 | 1.14E-07 | CD |
|  | rs17301746 | 15 | *LIPC** | 1.52E-06 | 0.00101 | 1.343E-15 | 0.000568 | UC |
| 141 | rs473224 | 15 | *LIPC** | 7.01E-55 | 1.53E-07 | 1.222E-50 | 1.08E-07 | CD |
|  | rs573922 | 15 | *LIPC** | 2.27E-51 | 1.53E-07 | 2.917E-47 | 1.14E-07 | CD |
|  | rs8041059 | 15 | *LIPC** | 8.77E-29 | 1.53E-07 | 5.652E-27 | 1.50E-07 | T1D |
| 142 | rs4775048 | 15 | *LIPC** | 2.45E-07 | 0.000196 | 5.188E-06 | 0.000139 | UC |
|  | rs936960 | 15 | *LIPC** | 2.14E-07 | 0.00016 | 1.42E-10 | 0.000131 | CD;UC |
| 143 | rs7162855 | 15 | *LIPC** | 1.04E-05 | 0.00511 | 7.039E-05 | 0.00321 | T1D |
| 145 | rs2652840 | 15 | *LACTB** | 9.75E-09 | 1.07E-05 | 1.088E-10 | 8.09E-06 | RA |
|  | rs8032104 | 15 | *LACTB** | 1.05E-08 | 1.07E-05 | 1.279E-07 | 3.22E-06 | CD |
|  | rs2729787 | 15 | *LACTB** | 5.15E-09 | 5.76E-06 | 6.542E-08 | 4.46E-06 | RA |
|  | rs4774474 | 15 | *LACTB** | 1.10E-08 | 1.07E-05 | 2.307E-07 | 3.72E-06 | CD |
|  | rs16946801 | 15 | *APH1B* | 2.66E-05 | 0.0113 | 5.621E-06 | 0.00605 | CD |
| 153 | rs2289119 | 16 | *SLC12A3* | 2.05E-19 | 1.53E-07 | 4.063E-17 | 1.22E-07 | CD |
|  | rs13306677 | 16 | *SLC12A3* | 4.70E-34 | 1.53E-07 | 1.352E-50 | 7.06E-08 | T1D |
|  | rs12445698 | 16 | *SLC12A3* | 3.65E-19 | 1.53E-07 | 2.803E-18 | 7.51E-08 | T1D |
|  | rs7187932 | 16 | *SLC12A3* | 2.03E-18 | 1.53E-07 | 3.182E-39 | 7.04E-08 | T1D |
|  | rs12446689 | 16 | *SLC12A3* | 8.59E-24 | 1.53E-07 | 3.411E-22 | 1.33E-07 | UC |
|  | rs2289114 | 16 | *SLC12A3* | 1.28E-17 | 1.53E-07 | 1.184E-16 | 7.04E-08 | T1D |
|  | rs8044243 | 16 | *SLC12A3* | 2.13E-25 | 1.53E-07 | 3.758E-22 | 8.73E-08 | T1D |
|  | rs8049280 | 16 | *SLC12A3* | 9.47E-25 | 1.53E-07 | 1.561E-21 | 9.51E-08 | CD |
|  | rs7204044 | 16 | *SLC12A3* | 6.58E-14 | 1.53E-07 | 6.461E-20 | 1.53E-07 | CD |
|  | rs1138429 | 16 | *SLC12A3* | 4.02E-44 | 1.53E-07 | 9.522E-66 | 2.12E-07 | RA |
|  | rs7204290 | 16 | *HERPUD1* | 1.86E-10 | 2.95E-07 | 1.479E-14 | 2.31E-07 | UC |
|  | rs2217332 | 16 | *HERPUD1* | 3.57E-27 | 1.53E-07 | 3.335E-25 | 1.14E-07 | CD |
|  | rs247615 | 16 | *HERPUD1* | 5.99E-42 | 1.53E-07 | 2.935E-62 | 1.14E-07 | CD |
|  | rs9989419 | 16 | *HERPUD1* | 2.31E-168 | 1.53E-07 | 0 | 1.22E-07 | CD |
|  | rs247617 | 16 | *CETP** | 7.11E-53 | 1.53E-07 | 0 | 1.57E-07 | PSOR |
|  | rs6499861 | 16 | *CETP** | 3.06E-31 | 1.53E-07 | 2.563E-28 | 1.73E-07 | T1D |
|  | rs6499863 | 16 | *CETP** | 2.72E-33 | 1.53E-07 | 9.745E-63 | 1.51E-07 | PSOR |
|  | rs3764261* | 16 | *CETP** | 4.13E-312 | 1.53E-07 | 0 | 1.31E-07 | CD |
|  | rs12447924 | 16 | *CETP** | 3.95E-41 | 1.53E-07 | 1.041E-53 | 5.37E-08 | UC |
|  | rs1800775 | 16 | *CETP** | 4.13e-312 | 1.53E-07 | 0 | 1.29E-07 | UC |
|  | rs1864163 | 16 | *CETP** | 8.50E-292 | 1.53E-07 | 0 | 1.50E-07 | T1D |
|  | rs7203984 | 16 | *CETP** | 1.62E-251 | 1.53E-07 | 0 | 1.41E-07 | CD |
|  | rs12597002 | 16 | *CETP** | 5.85E-59 | 1.53E-07 | 1.13E-102 | 7.71E-08 | UC |
|  | rs11076174 | 16 | *CETP** | 4.20E-75 | 1.53E-07 | 2.28E-127 | 1.72E-07 | CeD |
|  | rs1532625 | 16 | *CETP** | 1.67E-42 | 1.53E-07 | 0 | 1.29E-07 | UC |
|  | rs7499892 | 16 | *CETP** | 6.20E-282 | 1.53E-07 | 0 | 1.35E-07 | UC |
|  | rs5883 | 16 | *CETP** | 4.88E-06 | 0.00278 | 1.757E-31 | 0.00127 | T1D |
|  | rs289714 | 16 | *CETP** | 5.24E-159 | 1.53E-07 | 0 | 1.41E-07 | CD |
| 154 | rs289715 | 16 | *CETP** | 1.47E-56 | 1.53E-07 | 4.15E-98 | 1.57E-07 | PSOR |
|  | rs4784744 | 16 | *CETP** | 1.29E-52 | 1.53E-07 | 5.685E-50 | 1.31E-07 | CD |
|  | rs4784745 | 16 | *CETP** | 1.63E-48 | 1.53E-07 | 3.501E-46 | 1.45E-07 | RA |
|  | rs5880 | 16 | *CETP** | 2.24E-103 | 1.53E-07 | 1.37E-233 | 2.08E-07 | RA |
|  | rs5882 | 16 | *CETP** | 2.14E-62 | 1.53E-07 | 2.211E-58 | 8.66E-08 | T1D |
|  | rs9923854 | 16 | *CETP** | 1.56E-16 | 1.53E-07 | 1.532E-15 | 1.12E-07 | T1D |
|  | rs1801706 | 16 | *CETP** | 9.41E-17 | 1.53E-07 | 1.091E-15 | 1.41E-07 | CD |
|  | rs289742 | 16 | *CETP** | 2.68E-64 | 1.53E-07 | 1.968E-61 | 1.03E-07 | CD |
|  | rs289744 | 16 | *CETP** | 1.95E-69 | 1.53E-07 | 6.9E-138 | 8.66E-08 | T1D |
| 155 | rs12720917 | 16 | *CETP** | 9.43E-37 | 1.53E-07 | 5.89E-68 | 6.45E-08 | UC |
| 156 | rs289745 | 16 | *CETP** | 7.30E-40 | 1.53E-07 | 2.28E-20 | 5.90E-08 | T1D |
|  | rs17369163 | 16 | *CETP** | 1.22E-10 | 1.91E-07 | 6.143E-07 | 1.53E-07 | UC |
|  | rs12934552 | 16 | *NLRC5* | 2.01E-16 | 1.53E-07 | 8.707E-24 | 1.22E-07 | CD |
|  | rs17290922 | 16 | *NLRC5* | 1.30E-14 | 1.53E-07 | 3.445E-13 | 1.08E-07 | CD |
| 163 | rs11639620 | 16 | *CBFB* | 7.06E-06 | 0.00341 | 0.0001998 | 0.0015 | CD |
|  | rs2233455 | 16 | *NOL3* | 7.54E-10 | 1.05E-06 | 2.273E-14 | 4.03E-07 | T1D |
|  | rs10852437 | 16 | *KCTD19* | 2.72E-07 | 0.000196 | 1.189E-10 | 0.00012 | CD |
|  | rs1471143 | 16 | *LRRC36* | 1.10E-05 | 0.00511 | 7.668E-06 | 0.0032 | CD |
|  | rs8047080 | 16 | *LRRC36* | 1.93E-05 | 0.00927 | 1.199E-05 | 0.00596 | CD |
|  | rs9934827 | 16 | *LRRC36* | 2.16E-06 | 0.00124 | 1.259E-06 | 0.00106 | RA |
|  | rs7203742 | 16 | *CTCF* | 6.67E-13 | 1.53E-07 | 1.126E-17 | 5.73E-08 | CD |
|  | rs12449157 | 16 | *AX747090* | 2.26E-24 | 1.53E-07 | 7.847E-37 | 5.94E-08 | T1D |
|  | rs7202185 | 16 | *GFOD2* | 3.01E-10 | 4.53E-07 | 1.111E-15 | 2.90E-07 | T1D |
|  | rs8057184 | 16 | *RANBP10* | 7.45E-08 | 7.01E-05 | 6.008E-07 | 6.55E-05 | T1D |
|  | rs3743733 | 16 | *CENPT* | 5.07E-09 | 5.76E-06 | 8.653E-13 | 4.86E-06 | CD |
|  | rs7198357 | 16 | *NUTF2* | 5.98E-26 | 1.53E-07 | 4.277E-40 | 5.90E-08 | T1D |
|  | rs2271293 | 16 | *NUTF2* | 3.41E-33 | 1.53E-07 | 6.162E-31 | 9.44E-08 | T1D |
|  | rs4986970 | 16 | *LCAT** | 3.78E-08 | 3.75E-05 | 1.09E-15 | 4.40E-05 | RA |
|  | rs7201742 | 16 | *DUS2L* | 8.65E-28 | 1.53E-07 | 5.129E-46 | 9.92E-08 | CD |
|  | rs7190307 | 16 | *NFATC3* | 2.69E-06 | 0.00151 | 7.208E-11 | 0.00119 | PSOR |
|  | rs7199588 | 16 | *NFATC3* | 2.65E-12 | 1.53E-07 | 2.195E-31 | 9.51E-08 | CD |
|  | rs2305811 | 16 | *SLC7A6* | 6.65E-06 | 0.00341 | 1.153E-05 | 0.00363 | UC |
|  | rs11862968 | 16 | *SMPD3* | 2.21E-10 | 2.95E-07 | 4.465E-10 | 1.09E-07 | CD |
|  | rs8050499 | 16 | *SMPD3* | 2.52E-11 | 1.53E-07 | 1.094E-10 | 6.33E-08 | CD |
| 165 | rs2925979* | 16 | *CMIP** | 8.70E-12 | 1.53E-07 | 1.321E-19 | 1.22E-07 | CD |
| 167 | rs8070695 | 17 | *FBXL20* | 4.74E-12 | 1.53E-07 | 1.158E-18 | 6.61E-08 | CD |
|  | rs9889354 | 17 | *NEUROD2* | 2.27E-05 | 0.0113 | 7.754E-08 | 0.00801 | CD |
|  | rs12453682 | 17 | *NEUROD2* | 8.44E-13 | 1.53E-07 | 9.863E-12 | 5.96E-08 | T1D |
|  | rs9972882 | 17 | *STARD3** | 1.29E-11 | 1.53E-07 | 1.062E-17 | 1.96E-08 | CD |
|  | rs2952151 | 17 | *CAB2* | 2.43E-11 | 1.53E-07 | 6.469E-15 | 1.96E-08 | CD |
|  | rs1565922 | 17 | *CAB2* | 3.17E-11 | 1.53E-07 | 6.859E-14 | 1.96E-08 | CD |
|  | rs907091 | 17 | *IKZF3* | 8.92E-11 | 1.53E-07 | 1.608E-13 | 1.96E-08 | CD |
|  | rs1008723 | 17 | *GSDMB* | 4.51E-10 | 6.89E-07 | 1.47E-12 | 7.74E-08 | CD |
|  | rs4795405 | 17 | *ORMDL3* | 3.16E-07 | 0.00024 | 1.464E-06 | 2.00E-05 | UC |
|  | rs3894194 | 17 | *GSDMA* | 2.22E-07 | 0.00016 | 4.403E-07 | 1.31E-05 | UC |
|  | rs2302776 | 17 | *MED24* | 0.000167 | 0.0508 | 0.00019 | 0.00542 | UC |
|  | rs868150 | 17 | *MED24* | 0.000171 | 0.0508 | 0.001643 | 0.00603 | CD |
| 172 | rs12948339 | 17 | *ABCA8** | 1.21E-05 | 0.00623 | 1.307E-05 | 0.00584 | PSOR |
|  | rs4148008* | 17 | *ABCA8** | 8.10E-11 | 1.53E-07 | 1.125E-12 | 1.56E-07 | RA |
|  | rs4148005 | 17 | *ABCA8** | 8.81E-11 | 1.53E-07 | 5.743E-14 | 1.03E-07 | CD |
| 173 | rs4082919* | 17 | *PGS1** | 2.55E-09 | 3.06E-06 | 5.737E-11 | 1.42E-06 | UC |
|  | rs2292641 | 17 | *PGS1** | 7.66E-05 | 0.0295 | 4.88E-05 | 0.00862 | CD |
| 175 | rs2156498 | 18 | *DYM* | 1.06E-07 | 8.61E-05 | 2.175E-07 | 8.95E-05 | PSOR |
|  | rs2156499 | 18 | *C18orf32* | 1.36E-08 | 1.32E-05 | 4.749E-06 | 4.25E-06 | T1D |
|  | rs6507928 | 18 | *LIPG** | 1.75E-11 | 1.53E-07 | 4.561E-09 | 1.14E-07 | CD |
|  | rs11662909 | 18 | *LIPG** | 1.75E-11 | 1.53E-07 | 4.628E-11 | 1.22E-07 | CD |
|  | rs4556888 | 18 | *LIPG** | 2.16E-06 | 0.00124 | 2.08E-07 | 0.000702 | CD |
|  | rs2000813 | 18 | *LIPG** | 6.15E-15 | 1.53E-07 | 1.078E-23 | 1.31E-07 | CD |
|  | rs8093249 | 18 | *LIPG** | 2.87E-07 | 0.00024 | 1.799E-13 | 0.000185 | RA |
|  | rs11875600 | 18 | *LIPG** | 6.86E-09 | 7.10E-06 | 2.293E-12 | 4.76E-06 | T1D |
|  | rs3819166 | 18 | *LIPG** | 1.91E-09 | 2.47E-06 | 1.922E-08 | 9.39E-07 | T1D |
|  | rs3786248 | 18 | *LIPG** | 5.71E-08 | 5.70E-05 | 2.852E-14 | 2.76E-05 | CD |
|  | rs8092791 | 18 | *LIPG** | 5.49E-14 | 1.53E-07 | 7.131E-13 | 2.11E-07 | RA |
|  | rs17712928 | 18 | *LIPG** | 7.21E-12 | 1.53E-07 | 3.137E-16 | 1.26E-07 | UC |
| 176 | rs9954848 | 18 | *LIPG** | 1.50E-11 | 1.53E-07 | 5.093E-16 | 1.23E-07 | UC |
|  | rs8090363 | 18 | *LIPG** | 1.05E-24 | 1.53E-07 | 6.23E-31 | 9.92E-08 | CD |
|  | rs11872886 | 18 | *LIPG** | 3.96E-10 | 5.59E-07 | 2.076E-13 | 7.14E-07 | RA |
|  | rs6507932 | 18 | *LIPG** | 5.77E-10 | 8.50E-07 | 5.079E-09 | 6.73E-07 | UC |
|  | rs11664342 | 18 | *LIPG** | 4.92E-12 | 1.53E-07 | 6.683E-20 | 8.09E-08 | CD |
|  | rs6507934 | 18 | *LIPG** | 4.83E-28 | 1.53E-07 | 1.844E-38 | 1.08E-07 | CD |
|  | rs7229377 | 18 | *LIPG** | 4.80E-15 | 1.53E-07 | 2.668E-21 | 1.13E-07 | UC |
|  | rs7235005 | 18 | *LIPG** | 7.66E-15 | 1.53E-07 | 1.107E-18 | 9.38E-08 | T1D |
|  | rs7244811 | 18 | *LIPG** | 8.95E-33 | 1.53E-07 | 3.777E-43 | 7.51E-08 | T1D |
|  | rs7244595 | 18 | *LIPG** | 2.86E-15 | 1.53E-07 | 2.09E-19 | 8.09E-08 | CD |
|  | rs6507935 | 18 | *LIPG** | 4.50E-07 | 0.00036 | 3.474E-07 | 0.000416 | RA |
|  | rs7242671 | 18 | *LIPG** | 2.30E-06 | 0.00151 | 3.984E-07 | 0.000609 | UC |
|  | rs4939881 | 18 | *LIPG** | 2.33E-20 | 1.53E-07 | 3.592E-16 | 6.07E-08 | T1D |
|  | rs11659960 | 18 | *LIPG** | 3.84E-08 | 3.75E-05 | 1.292E-10 | 4.86E-05 | RA |
|  | rs4939883 | 18 | *LIPG** | 6.83E-51 | 1.53E-07 | 1.796E-66 | 1.02E-07 | T1D |
|  | rs12963082 | 18 | *LIPG** | 1.28E-07 | 0.000106 | 1.455E-10 | 6.53E-05 | CD |
|  | rs948937 | 18 | *LIPG** | 2.62E-14 | 1.53E-07 | 2.549E-18 | 9.51E-08 | CD |
|  | rs1540038 | 18 | *LIPG** | 1.56E-05 | 0.0076 | 6.888E-08 | 0.00529 | CD |
|  | rs1943969 | 18 | *LIPG** | 6.97E-11 | 1.53E-07 | 2.524E-13 | 1.31E-07 | CD |
|  | rs11662691 | 18 | *LIPG** | 7.19E-07 | 0.000543 | 2.519E-06 | 0.000656 | RA |
|  | rs7233748 | 18 | *LIPG** | 2.75E-12 | 1.53E-07 | 1.711E-10 | 8.09E-08 | CD |
|  | rs1943979 | 18 | *LIPG** | 4.54E-12 | 1.53E-07 | 1.308E-17 | 9.06E-08 | CD |
|  | rs882617 | 18 | *LIPG** | 1.39E-05 | 0.00623 | 3.265E-08 | 0.00343 | CD |
|  | rs12970803 | 18 | *LIPG** | 3.40E-09 | 3.78E-06 | 3.295E-07 | 2.71E-06 | CD |
|  | rs10502900 | 18 | *LIPG** | 2.34E-08 | 2.47E-05 | 2.166E-06 | 1.59E-05 | CD |
|  | rs11875988 | 18 | *LIPG** | 1.11E-12 | 1.53E-07 | 4.413E-10 | 1.03E-07 | CD |
|  | rs2000827 | 18 | *ACAA2* | 1.64E-12 | 1.53E-07 | 2.257E-15 | 1.94E-07 | RA |
|  | rs6507945 | 18 | *ACAA2* | 9.35E-22 | 1.53E-07 | 1.327E-34 | 1.02E-07 | T1D |
|  | rs8088428 | 18 | *ACAA2* | 2.33E-06 | 0.00151 | 4.563E-13 | 0.00113 | CD |
|  | rs7234826 | 18 | *ACAA2* | 3.04E-06 | 0.00185 | 5.568E-06 | 0.00165 | T1D |
|  | rs11082777 | 18 | *ACAA2* | 2.74E-06 | 0.00151 | 1.028E-05 | 0.00177 | RA |
|  | rs4939890 | 18 | *ACAA2* | 4.45E-13 | 1.53E-07 | 5.089E-19 | 1.14E-07 | CD |
|  | rs3892138 | 18 | *ACAA2* | 9.50E-14 | 1.53E-07 | 3.159E-21 | 1.45E-07 | RA |
|  | rs7241641 | 18 | *ACAA2* | 1.02E-14 | 1.53E-07 | 5.369E-26 | 1.31E-07 | CD |
|  | rs6507952 | 18 | *ACAA2* | 1.08E-14 | 1.53E-07 | 7.435E-26 | 1.31E-07 | CD |
|  | rs9964067 | 18 | *ACAA2* | 1.53E-14 | 1.53E-07 | 4.596E-14 | 1.31E-07 | CD |
|  | rs488191 | 18 | *MYO5B* | 6.87E-10 | 8.50E-07 | 5.15E-15 | 4.99E-07 | T1D |
|  | rs578211 | 18 | *MYO5B* | 1.84E-07 | 0.00016 | 1.479E-08 | 0.000123 | UC |
|  | rs3826579 | 18 | *MYO5B* | 2.67E-11 | 1.53E-07 | 2.478E-10 | 1.03E-07 | CD |
|  | rs4939903 | 18 | *MYO5B* | 3.96E-11 | 1.53E-07 | 3.595E-10 | 9.92E-08 | CD |
| 178 | rs12967135* | 18 | *MC4R** | 3.40E-09 | 3.78E-06 | 3.574E-08 | 4.79E-06 | RA |
|  | rs17782313 | 18 | *MC4R** | 6.14E-09 | 7.10E-06 | 1.698E-07 | 2.99E-06 | CD |
|  | rs476828 | 18 | *MC4R** | 6.15E-09 | 7.10E-06 | 5.773E-09 | 2.99E-06 | CD |
| 179 | rs17773235 | 18 | *MC4R** | 4.59E-06 | 0.00278 | 6.092E-07 | 0.00141 | CD |
|  | rs17700633 | 18 | *MC4R** | 2.29E-06 | 0.00151 | 5.151E-07 | 0.000765 | CD |
| 180 | rs1943226 | 18 | *MC4R** | 6.84E-06 | 0.00341 | 0.0001806 | 0.00376 | T1D |
| 183 | rs2278236 | 19 | *ANGPTL4** | 2.24E-08 | 2.00E-05 | 3.185E-18 | 1.41E-05 | CD |
|  | rs2913972 | 19 | *RAB11B* | 1.02E-05 | 0.00511 | 7.768E-05 | 0.00601 | RA |
|  | rs2913968 | 19 | *RAB11B* | 1.06E-05 | 0.00511 | 1.815E-12 | 0.00424 | T1D |
|  | rs2230876 | 19 | *RAB11B* | 1.10E-05 | 0.00511 | 1.005E-10 | 0.00437 | PSOR |
| 187 | rs737337* | 19 | *DOCK6* | 1.56E-09 | 1.99E-06 | 4.564E-17 | 1.27E-06 | UC |
| 193 | rs445925 | 19 | *APOC1** | 2.26E-07 | 0.000196 | 1.898E-10 | 6.94E-05 | UC |
| 204 | rs1800961* | 20 | *HNF4A** | 3.03E-16 | 1.53E-07 | 1.639E-34 | 9.88E-08 | UC |
| 205 | rs12480887 | 20 | *DNTTIP1* | 9.01E-07 | 0.000666 | 4.434E-07 | 0.000373 | UC |
|  | rs3817731 | 20 | *NEURL2* | 3.15E-06 | 0.00185 | 2.44E-05 | 0.000592 | T1D |
|  | rs6065904 | 20 | *PLTP** | 1.89E-19 | 1.53E-07 | 1.949E-18 | 1.14E-07 | CD |
|  | rs378114 | 20 | *PLTP** | 5.97E-11 | 1.53E-07 | 1.29E-13 | 1.26E-07 | UC |
|  | rs435306 | 20 | *PLTP** | 1.08E-10 | 1.53E-07 | 1.942E-13 | 1.23E-07 | UC |
|  | rs4810479 | 20 | *PLTP** | 6.15E-22 | 1.53E-07 | 1.21E-33 | 1.26E-07 | UC |
|  | rs6065906* | 20 | *PCIF1* | 3.05E-23 | 1.53E-07 | 5.336E-40 | 9.51E-08 | CD |
|  | rs3746506 | 20 | *ZNF335* | 5.46E-12 | 1.53E-07 | 7.717E-11 | 5.96E-08 | T1D |
|  | rs6065912 | 20 | *MMP9* | 6.17E-08 | 5.70E-05 | 1.282E-14 | 2.40E-05 | CD |
|  | rs3918261 | 20 | *MMP9* | 2.84E-06 | 0.00185 | 4.335E-12 | 0.000695 | CD |
| 207 | rs181360 | 22 | *UBE2L3** | 8.55E-09 | 8.72E-06 | 9.238E-18 | 8.77E-07 | CD |
|  | rs181362* | 22 | *UBE2L3** | 5.87E-09 | 7.10E-06 | 4.303E-18 | 7.16E-07 | CD |
|  | rs5754217 | 22 | *UBE2L3** | 1.46E-08 | 1.62E-05 | 4.359E-08 | 1.55E-06 | UC |

Independent complex or single gene loci (r^2^ < 0.2) with SNP(s) with a conditional FDR (condFDR) < 0.01 in high density lipoprotein (HDL) condition on the associated immune mediated disease. All SNPs with a condFDR value < 0.01 are listed. For HDL and immune-mediated disease phenotype we defined the most significant SNP in each LD block based on the minimum condFDR (min FDR). For comparison, the minimal condFDR values for each identified SNP are listed for the phenotypes based on which this value was obtained (Driving phenotype, multiple phenotypes separated by semi-colon). In addition, the chromosome number (Chr), closest gene (Gene symbol), unconditional false discovery rates (FDR) and p values of each SNPs for the corresponding lipids are given. The corresponding p values from the larger sample of HDL (HDL2 P-value) are also listed. Independent loci which do not contain any known SNPs or genes are listed first consecutively, followed by loci containing SNPs or genes, which were marked by stars (*). Crohn’s Disease (CD), ulcerative colitis (UC), rheumatoid arthritis (RA), type 1 diabetes (T1D), celiac disease (CeD), psoriasis (PSOR) and sarcoidosis (SARC). Chromosome (Chr). All data were first corrected for genomic inflation. NA; not available.

**Table E. Non-MHC pleiotropic SNPs in triglycerides (TG) and immune-mediated diseases (conj FDR<0.01)**

| **locus** | **SNP** | **gene** | **MIM** | **chr** | **Min conjFDR** | **TG & CD** | **TG & UC** | **TG & RA** | **TG & T1D** | **TG & CeD** | **TG & PSOR** | **TG & SARC** |
| --- | --- | --- | --- | --- | --- | --- | --- | --- | --- | --- | --- | --- |
| 12 | rs4409689 | *DOCK7* | - | 1 | 5.34E-03 | 5.34E-03 | 9.08E-01 | 9.87E-01 | NA | 2.74E-01 | 9.18E-01 | 9.33E-01 |
| 42 | rs1260326 | *GCKR* | 600842 | 2 | 9.76E-03 | 9.76E-03 | 4.45E-02 | 8.08E-01 | 2.85E-01 | 7.39E-01 | 9.03E-01 | 9.56E-01 |
|  | rs2068834 | *ZNF512* | - | 2 | 2.25E-03 | 3.28E-01 | 2.25E-03 | 8.08E-01 | 3.75E-01 | 8.71E-01 | 9.80E-01 | 9.93E-01 |
|  | rs898031 | *BRE* | 610497 | 2 | 3.19E-03 | 6.10E-01 | 3.19E-03 | 9.56E-01 | 2.17E-01 | 9.83E-01 | 5.37E-01 | 7.42E-01 |
| 44 | rs2338437 | *FOSL2* | 601575 | 2 | 4.74E-03 | 4.74E-03 | 4.46E-01 | 9.56E-01 | 2.85E-01 | 9.06E-01 | 9.80E-01 | 9.53E-01 |
| 75 | rs310751 | *NR_003112* | - | 3 | 7.26E-03 | 8.81E-01 | 9.68E-01 | 3.11E-01 | 7.26E-03 | NA | 9.03E-01 | 9.56E-01 |
| 139 | rs272869 | *SLC22A4* | 604190 | 5 | 3.64E-03 | 3.64E-03 | 9.68E-01 | 9.80E-01 | NA | NA | 9.18E-01 | 9.56E-01 |
| 142 | rs7724832 | *TIMD4* | 610096 | 5 | 4.17E-03 | 4.17E-03 | 5.83E-01 | 8.83E-01 | 4.80E-01 | NA | 8.71E-01 | 8.46E-01 |
| 166 | rs2503322 | *RSPO3* | 610574 | 6 | 2.28E-03 | 2.28E-03 | 8.69E-01 | 9.72E-01 | 4.26E-01 | 5.62E-01 | 9.03E-01 | 9.93E-01 |
| 205 | rs3021494 | *XKR6* | - | 8 | 1.53E-03 | 6.73E-01 | 8.40E-01 | 9.72E-01 | NA | NA | 1.53E-03 | 9.56E-01 |
|  | rs9644737 | *C8orf12* | - | 8 | 9.79E-03 | NA | 8.87E-01 | 9.56E-01 | 9.79E-03 | 1.00E+00 | 9.80E-01 | 9.93E-01 |
|  | rs998683 | *BLK* | 191305 | 8 | 8.31E-03 | 5.52E-01 | 7.97E-01 | 8.31E-03 | 2.85E-01 | NA | 9.31E-01 | 8.60E-01 |
|  | rs1296023 | *CTSB* | 116810 | 8 | 5.14E-03 | 1.00E+00 | 2.79E-02 | 9.87E-01 | 5.14E-03 | NA | 9.80E-01 | 9.93E-01 |
| 207 | rs1961456 | *NAT2* | 612182 | 8 | 3.40E-03 | 7.34E-01 | 8.87E-01 | 3.40E-03 | NA | NA | 6.58E-01 | 8.21E-01 |
| 211 | rs2278615 | *INTS10* | 611353 | 8 | 8.34E-03 | NA | NA | 8.34E-03 | NA | NA | NA | NA |
| 277 | rs102275 | *TMEM258(C11orf10)* | - | 11 | 2.07E-04 | 2.07E-04 | 7.97E-01 | 3.62E-01 | 4.26E-01 | 2.07E-01 | 8.71E-01 | 8.69E-01 |
|  | rs968567 | *FADS2* | 606149 | 11 | 5.69E-04 | 1.46E-01 | 9.08E-01 | 5.69E-04 | 2.17E-01 | 2.07E-01 | 9.31E-01 | 8.69E-01 |
| 311 | rs7398833 | *CUX2* | 610648 | 12 | 6.12E-03 | NA | 8.87E-01 | 5.60E-01 | 6.12E-03 | NA | 1.00E+00 | 1.00E+00 |
| 363 | rs8062719 | *STX1B* | 601485 | 16 | 6.29E-04 | 8.37E-01 | 6.24E-01 | 1.00E+00 | NA | NA | 6.29E-04 | 9.56E-01 |
| 391 | rs7221651 | *BPTF* | 601819 | 17 | 7.85E-03 | 8.81E-01 | 1.00E+00 | 9.87E-01 | 7.85E-03 | NA | 9.80E-01 | 1.00E+00 |
| 409 | rs2965185 | *GATAD2A* | 614997 | 19 | 2.18E-03 | 8.37E-01 | 9.68E-01 | 2.18E-03 | NA | NA | 9.03E-01 | 9.33E-01 |
| 425 | rs676388 | *FUT2* | 182100 | 19 | 2.00E-03 | 2.00E-03 | 1.00E+00 | 1.00E+00 | 2.57E-03 | 1.07E-01 | 1.23E-01 | 6.18E-01 |

Independent complex or single gene loci (r^2^ < 0.2) with SNP(s) with a conjunctional FDR (conjFDR) < 0.01 in both Triglycerides (TG) and in the associated immune-mediated disease. All SNPs with a conjFDR value < 0.01 (bidirectional association, i.e. association with lipid phenotype given the immune-mediated disease (condFDR< 0.01) and association with immune mediated disease given lipid phenotypes (condFDR<0.01)) are listed and sorted in each LD block. For TG and immune mediated disease phenotype we defined the most significant SNP in each LD block based on the minimum conjFDR (min conjFDR). For comparison, the conjFDR values for each identified SNP are listed for all phenotypes, and the minimum (min) conjFDR across all phenotypes. All independent loci are listed consecutively by chromosomal location, and one SNP is listed for MCH region on chr 6. In addition, the closest gene and MIM number are listed for each SNP when available from NCBI gene database. Chromosome (Chr). Not available (NA). All data were first corrected for genomic inflation. Loci with conjFDR < 0.05 are shown in Table E, and z-scores are shown in Table H, using same locus number.

**Table F. Non-MHC pleiotropic SNPs in low density lipoprotein (LDL) and immune-mediated diseases (conj FDR<0.01).**

| **locus** | **SNP** | **gene** | **MIMI** | **chr** | **Min conjFDR** | **LDL & CD** | **LDL & UC** | **LDL & RA** | **LDL & T1D** | **LDL & CeD** | **LDL & PSOR** | **LDL & SARC** |
| --- | --- | --- | --- | --- | --- | --- | --- | --- | --- | --- | --- | --- |
| 23 | rs4409689 | *DOCK7* | - | 1 | 6.11E-03 | 6.11E-03 | 9.29E-01 | 8.85E-01 | NA | 2.72E-01 | 9.50E-01 | 9.61E-01 |
| 38 | rs2938616 | *EPS8L3* | 614989 | 1 | 4.76E-03 | 4.94E-01 | 4.76E-03 | 9.60E-01 | 9.68E-01 | 8.57E-01 | 9.60E-01 | 9.56E-01 |
| 44 | rs7551957 | *FCGR2A* | 146790 | 1 | 4.31E-03 | NA | NA | 4.31E-03 | NA | NA | NA | NA |
| 82 | rs932206 | *CXCR4* | 162643 | 2 | 4.31E-03 | 9.12E-01 | 5.08E-01 | 5.63E-01 | 2.70E-01 | 4.31E-03 | 9.17E-01 | 9.99E-01 |
| 99 | rs299639 | *TSEN2* | 608753 | 3 | 5.96E-03 | 5.36E-01 | 1.00E+00 | 3.41E-01 | 5.96E-03 | 1.00E+00 | 9.87E-01 | 9.99E-01 |
| 136 | rs6878664 | *CTNND2* | 604275 | 5 | 7.58E-03 | 8.97E-01 | 9.86E-01 | 7.58E-03 | 5.38E-01 | 2.37E-01 | 1.00E+00 | 9.99E-01 |
| 151 | rs9282763 | *LOC441108* | - | 5 | 2.44E-03 | 2.44E-03 | 3.92E-01 | 6.60E-01 | NA | NA | 9.87E-01 | 9.62E-01 |
| 155 | rs7724832 | *TIMD4* | 610096 | 5 | 4.32E-03 | 4.32E-03 | 5.08E-01 | 7.75E-01 | 5.38E-01 | NA | 9.58E-01 | 8.46E-01 |
| 234 | rs1961456 | *NAT2* | 612182 | 8 | 7.58E-03 | 8.63E-01 | 9.00E-01 | 7.58E-03 | NA | NA | 8.49E-01 | 8.11E-01 |
| 288 | rs11187157 | *HHEX* | 604420 | 10 | 5.86E-03 | NA | 5.86E-03 | 9.60E-01 | NA | NA | 9.60E-01 | 9.57E-01 |
| 300 | rs102275 | *TMEM258(C11orf10)* | - | 11 | 1.13E-04 | 1.13E-04 | 7.66E-01 | 3.41E-01 | 4.89E-01 | 2.06E-01 | 9.58E-01 | 9.02E-01 |
|  | rs968567 | *FADS2* | 606149 | 11 | 9.17E-04 | 1.93E-01 | 9.29E-01 | 9.17E-04 | 2.70E-01 | 2.06E-01 | 9.47E-01 | 9.02E-01 |
| 331 | rs3184504 | *SH2B3* | 605093 | 12 | 3.50E-07 | NA | NA | 9.76E-02 | 4.87E-07 | 3.50E-07 | NA | NA |
|  | rs630512 | *ATXN2* | 601517 | 12 | 3.37E-05 | NA | 8.65E-01 | 6.10E-01 | 3.37E-05 | 2.06E-01 | 6.30E-01 | 9.62E-01 |
|  | rs11066320 | *PTPN11* | 176876 | 12 | 2.66E-06 | NA | 2.50E-01 | 1.16E-01 | 2.66E-06 | 9.30E-06 | 7.56E-01 | 9.57E-01 |
| 365 | rs2472304 | *CYP1A2* | 124060 | 15 | 8.39E-03 | 9.12E-01 | 9.00E-01 | 9.06E-01 | 1.00E+00 | 8.39E-03 | NA | 9.57E-01 |
| 424 | rs7253253 | *SLC44A2* | 606106 | 19 | 4.32E-03 | 4.32E-03 | 1.77E-01 | 6.60E-01 | NA | NA | 2.66E-01 | 9.62E-01 |
|  | rs12974306 | *DNM2* | 602378 | 19 | 4.86E-03 | 7.56E-01 | 1.00E+00 | 5.63E-01 | 8.00E-01 | 1.00E+00 | 4.86E-03 | 7.70E-01 |
|  | rs11085749 | *C19orf38* | - | 19 | 1.34E-03 | 8.87E-01 | 1.00E+00 | 9.17E-01 | 6.40E-01 | 7.68E-01 | 1.34E-03 | 9.02E-01 |
|  | rs12983316 | *SMARCA4* | 603254 | 19 | 9.07E-04 | NA | 9.86E-01 | 9.17E-01 | NA | NA | 9.07E-04 | 5.96E-01 |
| 427 | rs7188 | *KANK2* | 614610 | 19 | 4.31E-03 | 6.93E-01 | 9.29E-01 | 4.31E-03 | 8.89E-01 | 7.68E-01 | 9.47E-01 | 8.77E-01 |
| 432 | rs2965185 | *GATAD2A* | 614997 | 19 | 3.47E-03 | 8.97E-01 | 9.86E-01 | 3.47E-03 | NA | NA | 9.60E-01 | 9.61E-01 |
| 454 | rs516246 | *FUT2* | 182100 | 19 | 9.37E-06 | 9.37E-06 | 1.00E+00 | 1.00E+00 | NA | NA | 8.36E-02 | 5.74E-01 |
| 483 | rs4820821 | *ASCC2* | 614216 | 22 | 6.50E-03 | 1.46E-01 | 9.00E-01 | 8.38E-01 | 6.50E-03 | NA | 6.30E-01 | 9.61E-01 |

Independent complex or single gene loci (r^2^ < 0.2) with SNP(s) with a conjunctional FDR (conjFDR) < 0.01 in both Low density lipoproteins (LDL) and in the associated immune-mediated disease. All SNPs with a conjFDR value < 0.01 (bidirectional association, i.e. association with lipid phenotype given the immune-mediated disease (condFDR< 0.01) and association with immune mediated disease given lipid phenotypes (condFDR<0.01)) are listed and sorted in each LD block. For LDL and immune mediated disease phenotype we defined the most significant SNP in each LD block based on the minimum conjFDR (min conjFDR). For comparison, the conjFDR values for each identified SNP are listed for all phenotypes, and the minimum (min) conjFDR across all phenotypes. All independent loci are listed consecutively by chromosomal location, and one SNP is listed for MCH region on chr 6. In addition, the closest gene and MIM number are listed for each SNP when available from NCBI gene database. Chromosome (Chr). Not available (NA). All data were first corrected for genomic inflation. Loci with conjFDR < 0.05 are shown in Table F, and z-scores are shown in Table I, using same locus number.

**Table G. Non-MHC pleiotropic SNPs in high density lipoprotein (HDL) and immune-mediated diseases (conj FDR<0.01).**

| **locus** | **SNP** | **gene** | **MIM** | **chr** | **Min conjFDR** | **HDL & CD** | **HDL & UC** | **HDL & RA** | **HDL & T1D** | **HDL & CeD** | **HDL & PSOR** | **HDL & SARC** |
| --- | --- | --- | --- | --- | --- | --- | --- | --- | --- | --- | --- | --- |
| 31 | rs3813982 | *SRGAP2* | 606524 | 1 | 8.04E-03 | 1.00E+00 | 8.04E-03 | 7.62E-02 | 7.59E-01 | 8.56E-01 | 9.11E-01 | 9.57E-01 |
| 45 | rs17799872 | *ADCY3* | 600291 | 2 | 9.09E-03 | 6.74E-01 | 7.87E-01 | 9.28E-01 | 9.09E-03 | 8.66E-01 | 9.09E-01 | 8.01E-01 |
| 77 | rs7613875 | *MON1A* | 611464 | 3 | 5.14E-05 | 5.14E-05 | 6.38E-04 | 9.89E-01 | NA | NA | 9.09E-01 | 9.92E-01 |
|  | rs12496973 | *RBM6* | 606886 | 3 | 1.52E-04 | 1.52E-04 | 1.59E-04 | 1.00E+00 | NA | NA | 9.03E-01 | 1.00E+00 |
| 79 | rs2336725 | *RFT1* | 611908 | 3 | 3.72E-03 | 3.72E-03 | 8.86E-01 | 1.00E+00 | 5.33E-01 | 8.71E-01 | 8.94E-01 | 1.00E+00 |
| 141 | rs2489623 | *RSPO3* | 610574 | 6 | 2.53E-03 | 2.53E-03 | 9.58E-01 | 9.64E-01 | 3.89E-01 | NA | 9.09E-01 | 1.00E+00 |
| 152 | rs836472 | *RAC1* | 602048 | 7 | 2.12E-03 | 4.90E-01 | 2.12E-03 | 5.72E-01 | 7.59E-01 | 8.36E-01 | 9.11E-01 | 9.57E-01 |
| 162 | rs4917014 | *IKZF1* | 603023 | 7 | 6.06E-03 | 6.06E-03 | 1.46E-01 | 3.69E-01 | 5.35E-02 | 8.36E-01 | 9.76E-01 | 9.92E-01 |
| 200 | rs2223103 | *TRPS1* | 604386 | 8 | 5.77E-03 | 9.02E-01 | 5.77E-03 | 1.00E+00 | 9.63E-01 | NA | 9.11E-01 | 9.11E-01 |
| 266 | rs102275 | *TMEM258*  *(C11orf10)* | - | 11 | 5.53E-05 | 5.53E-05 | 7.39E-01 | 4.85E-01 | 4.83E-01 | 3.62E-01 | 9.09E-01 | 1.00E+00 |
|  | rs968567 | *FADS2* | 606149 | 11 | 6.12E-03 | 1.49E-01 | 8.86E-01 | 6.12E-03 | 2.81E-01 | 3.62E-01 | 9.22E-01 | 1.00E+00 |
| 285 | rs473465 | *INHBE* | 612031 | 12 | 5.35E-04 | 1.00E+00 | 5.35E-04 | 6.08E-01 | NA | NA | 9.76E-01 | 1.00E+00 |
| 291 | rs653178 | *ATXN2* | 601517 | 12 | 3.17E-04 | NA | 2.76E-01 | 1.58E-01 | 3.17E-04 | 4.88E-04 | 7.95E-01 | 1.00E+00 |
| 319 | rs2899624 | *ALDH1A2* | 603687 | 15 | 4.88E-03 | 9.02E-01 | 4.88E-03 | 8.85E-01 | 9.63E-01 | 8.78E-01 | 9.76E-01 | 9.85E-01 |
| 344 | rs12448482 | *NPIPB15(NPIPL2)* | - | 16 | 6.06E-03 | 6.06E-03 | 3.42E-01 | 4.48E-01 | NA | NA | 9.76E-01 | 1.00E+00 |
| 377 | rs879606 | *PPP1R1B* | 604399 | 17 | 8.96E-03 | 8.96E-03 | 1.46E-01 | 6.54E-02 | 2.81E-01 | 9.82E-01 | 8.87E-01 | 9.92E-01 |
|  | rs907092 | *IKZF3* | 606221 | 17 | 8.36E-06 | 1.59E-05 | 8.36E-06 | 4.65E-03 | 6.53E-04 | 1.00E+00 | 9.76E-01 | 1.00E+00 |
|  | rs2872507 | *ZPBP2* | 608499 | 17 | 3.19E-06 | 3.19E-06 | 3.20E-06 | 7.04E-03 | 5.23E-05 | 9.82E-01 | 9.22E-01 | 1.00E+00 |
|  | rs1008723 | *GSDMB* | 611221 | 17 | 2.26E-05 | 1.77E-04 | 7.05E-05 | 1.89E-02 | 2.26E-05 | NA | 1.00E+00 | 1.00E+00 |
|  | rs11078936 | *MED24(THRAP4)* | 607000 | 17 | 9.69E-03 | 2.94E-01 | 9.69E-03 | 1.00E+00 | 1.99E-02 | 8.66E-01 | 9.11E-01 | 9.62E-01 |
| 383 | rs10491182 | *NFE2L1* | 163260 | 17 | 8.10E-03 | 7.31E-01 | 3.07E-01 | 8.85E-01 | 7.59E-01 | 8.10E-03 | 9.03E-01 | 1.00E+00 |
| 448 | rs181362 | *UBE2L3* | 603721 | 22 | 7.15E-07 | 7.15E-07 | 8.28E-03 | 4.77E-02 | NA | NA | 5.66E-01 | 1.00E+00 |
|  | rs713875 | *HORMAD2* | - | 22 | 7.70E-03 | 7.70E-03 | 1.05E-01 | 3.29E-01 | NA | NA | 9.11E-01 | 1.00E+00 |
| 454 | rs1569501 | *RPL3* | 604163 | 22 | 5.35E-04 | 1.34E-02 | 5.35E-04 | 4.09E-01 | NA | NA | 9.76E-01 | 1.00E+00 |

Independent complex or single gene loci (r^2^ < 0.2) with SNP(s) with a conjunctional FDR (conjFDR) < 0.01 in both high density lipoproteins (HDL) and in the associated immune-mediated disease. All SNPs with a conjFDR value < 0.01 (bidirectional association, i.e. association with lipid phenotype given the immune-mediated disease (condFDR< 0.01) and association with immune mediated disease given lipid phenotypes (condFDR<0.01)) are listed and sorted in each LD block. For HDL and immune mediated disease phenotype we defined the most significant SNP in each LD block based on the minimum conjFDR (min conjFDR). For comparison, the conjFDR values for each identified SNP are listed for all phenotypes, and the minimum (min) conjFDR across all phenotypes. All independent loci are listed consecutively by chromosomal location, and one SNP is listed for MCH region on chr 6. In addition, the closest gene and MIM number are listed for each SNP when available from NCBI gene database. Chromosome (Chr). Not available (NA). All data were first corrected for genomic inflation. Loci with conjFDR < 0.05 are shown in Table G, and z-scores are shown in Table J, using same locus number.

### Table H. Pleiotropic SNPs in triglycerides (TG) and immune mediated diseases (conjFDR<0.05) .

| **#Locus** | **SNP** | **gene** | **chr** | **Min conjFDR** | **TG & CD** | **TG & UC** | **TG & RA** | **TG & T1D** | **TG & CeD** | **TG & PSOR** | **TG & SARC** |
| --- | --- | --- | --- | --- | --- | --- | --- | --- | --- | --- | --- |
| 4 | rs10903118 | *AML2* | 1 | 2.02E-02 | 2.40E-01 | 9.68E-01 | 6.72E-01 | NA | 2.02E-02 | 4.21E-01 | 8.46E-01 |
| 6 | rs502621 | *EPHA10* | 1 | 3.57E-02 | 8.85E-01 | 8.41E-01 | 3.57E-02 | 8.74E-01 | 3.72E-01 | 9.09E-01 | 9.91E-01 |
| 7 | rs2293476 | *PABPC4* | 1 | 2.32E-02 | 2.14E-01 | 8.40E-01 | 1.00E+00 | 2.32E-02 | NA | 7.17E-01 | 1.00E+00 |
| 12 | rs4409689 | *DOCK7* | 1 | 5.34E-03 | 5.34E-03 | 9.08E-01 | 9.87E-01 | NA | 2.74E-01 | 9.18E-01 | 9.33E-01 |
|  | rs7512480 | *ATG4C* | 1 | 4.42E-02 | 4.42E-02 | 8.69E-01 | 9.80E-01 | 6.91E-02 | 9.06E-01 | 9.18E-01 | 8.21E-01 |
| 19 | rs12128454 | *MAGI3* | 1 | 3.57E-02 | NA | NA | 3.57E-02 | NA | NA | NA | NA |
| 27 | rs2797257 | *USH2A* | 1 | 4.24E-02 | 8.09E-01 | 6.66E-01 | 9.74E-01 | 4.24E-02 | NA | 3.13E-01 | 9.89E-01 |
| 35 | rs7590536 | *ASAP2* | 2 | 3.22E-02 | 9.59E-01 | 7.94E-01 | 9.80E-01 | NA | NA | 3.22E-02 | 9.64E-01 |
| 42 | rs11684134 | *GTF3C2* | 2 | 1.05E-02 | 3.63E-01 | 1.05E-02 | 9.56E-01 | 5.99E-01 | 9.06E-01 | 5.97E-01 | 8.60E-01 |
|  | rs1260326 | *GCKR* | 2 | 9.76E-03 | 9.76E-03 | 4.45E-02 | 8.08E-01 | 2.85E-01 | 7.39E-01 | 9.03E-01 | 9.56E-01 |
|  | rs2068834 | *ZNF512* | 2 | 2.25E-03 | 3.28E-01 | 2.25E-03 | 8.08E-01 | 3.75E-01 | 8.71E-01 | 9.80E-01 | 9.93E-01 |
|  | rs13013484 | *MRPL33* | 2 | 4.77E-02 | 4.48E-01 | 9.68E-01 | 9.56E-01 | 7.70E-01 | 4.77E-02 | 9.31E-01 | 9.56E-01 |
|  | rs898031 | *BRE* | 2 | 3.19E-03 | 6.10E-01 | 3.19E-03 | 9.56E-01 | 2.17E-01 | 9.83E-01 | 5.37E-01 | 7.42E-01 |
|  | rs4401177 | *BRE* | 2 | 6.72E-03 | 6.72E-03 | 8.40E-01 | 9.80E-01 | 5.99E-01 | 7.88E-01 | 1.02E-01 | 9.06E-01 |
|  | rs4665408 | *BRE* | 2 | 4.45E-02 | 9.08E-02 | 4.45E-02 | 8.08E-01 | 9.56E-01 | 8.50E-01 | 7.74E-01 | 9.06E-01 |
|  | rs12617913 | *BRE* | 2 | 6.72E-03 | 6.72E-03 | 7.05E-02 | 8.83E-01 | NA | NA | 6.58E-01 | 9.56E-01 |
| 42 | rs10184619 | *BRE* | 2 | 4.75E-02 | 5.32E-02 | 3.27E-01 | 8.83E-01 | NA | NA | 4.75E-02 | 9.33E-01 |
| 43 | rs925255 | *AK055918* | 2 | 1.14E-02 | 1.14E-02 | 8.35E-02 | 9.51E-01 | 2.48E-01 | 7.39E-01 | 9.03E-01 | 9.33E-01 |
|  | rs7590263 | *FOSL2* | 2 | 3.46E-02 | NA | 3.46E-02 | 1.00E+00 | 1.08E-01 | NA | 8.26E-01 | 9.93E-01 |
| 44 | rs2338437 | *FOSL2* | 2 | 4.74E-03 | 4.74E-03 | 4.46E-01 | 9.56E-01 | 2.85E-01 | 9.06E-01 | 9.80E-01 | 9.53E-01 |
| 51 | rs7557569 | *SPRED2* | 2 | 4.23E-02 | 9.59E-01 | 8.64E-01 | 4.23E-02 | 5.94E-01 | 5.84E-01 | 6.14E-01 | 7.45E-01 |
| 53 | rs2521932 | *LOC389033* | 2 | 4.11E-02 | NA | NA | 4.11E-02 | NA | NA | NA | NA |
| 67 | rs7596727 | *ICOS* | 2 | 2.53E-02 | 9.56E-01 | 8.89E-01 | 2.53E-02 | 3.13E-02 | NA | 9.81E-01 | 4.50E-01 |
|  | rs1978594 | *ICOS* | 2 | 2.11E-02 | NA | NA | 2.11E-02 | NA | NA | NA | NA |
| 68 | rs2115591 | *DYTN* | 2 | 3.76E-02 | 7.55E-01 | 7.71E-01 | 9.99E-01 | 7.98E-01 | 9.23E-01 | 3.76E-02 | 6.17E-01 |
| 75 | rs310751 | *NR_003112* | 3 | 7.26E-03 | 8.81E-01 | 9.68E-01 | 3.11E-01 | 7.26E-03 | NA | 9.03E-01 | 9.56E-01 |
| 88 | rs540722 | *AK097161* | 3 | 3.93E-02 | 6.96E-01 | 2.70E-01 | 7.72E-01 | 7.46E-01 | 3.93E-02 | 9.41E-01 | 7.00E-01 |
| 93 | rs10018612 | *EVC* | 4 | 4.73E-02 | NA | 1.12E-01 | 9.76E-01 | NA | 9.78E-01 | 4.73E-02 | 5.54E-01 |
| 95 | rs6846421 | *C4orf52* | 4 | 2.33E-02 | 3.63E-01 | 8.40E-01 | 2.33E-02 | NA | NA | 1.00E+00 | 9.93E-01 |
|  | rs4692059 | *C4orf52* | 4 | 3.47E-02 | 2.67E-01 | 5.83E-01 | 4.11E-02 | 3.47E-02 | NA | 8.26E-01 | 9.56E-01 |
| 96 | rs10939100 | *RBPJ* | 4 | 4.98E-02 | 6.73E-01 | 8.40E-01 | 1.00E+00 | 4.98E-02 | 6.82E-01 | 1.00E+00 | 9.93E-01 |
| 103 | rs170869 | *SLC39A8* | 4 | 1.14E-02 | 1.14E-02 | 7.97E-01 | 8.83E-01 | 5.99E-01 | 7.88E-01 | 9.80E-01 | 9.56E-01 |
| 134 | rs7734159 | *EDIL3* | 5 | 2.22E-02 | 1.00E+00 | 9.11E-01 | 7.72E-01 | 9.60E-01 | 8.90E-01 | 2.22E-02 | 9.57E-01 |
| 139 | rs272869 | *SLC22A4* | 5 | 3.64E-03 | 3.64E-03 | 9.68E-01 | 9.80E-01 | NA | NA | 9.18E-01 | 9.56E-01 |
| 142 | rs7724832 | *TIMD4* | 5 | 4.17E-03 | 4.17E-03 | 5.83E-01 | 8.83E-01 | 4.80E-01 | NA | 8.71E-01 | 8.46E-01 |
| 148* | rs1324082 | *SLC17A1* | 6 | 4.40E-02 | 1.00E+00 | 8.97E-01 | 9.75E-01 | 4.40E-02 | 4.66E-02 | 9.79E-01 | 1.00E+00 |
|  | rs942378 | *SLC17A1* | 6 | 3.93E-02 | NA | NA | 9.74E-01 | NA | 3.93E-02 | NA | NA |
| 149* | rs6904596 | *ZNF184* | 6 | 5.38E-03 | 2.67E-01 | 4.91E-01 | 7.38E-01 | 1.02E-02 | 1.07E-02 | 5.38E-03 | 9.56E-01 |
|  | rs13194781 | *HIST1H2BN* | 6 | 4.32E-03 | 2.40E-01 | 6.63E-01 | 6.72E-01 | 4.32E-03 | 4.48E-03 | 1.82E-02 | 9.56E-01 |
|  | rs7766356 | *ZSCAN23* | 6 | 1.93E-02 | 6.10E-01 | 5.83E-01 | 1.93E-02 | NA | NA | 1.23E-01 | 8.83E-01 |
|  | rs2746150 | *OR2H1* | 6 | 5.14E-03 | 2.97E-01 | 8.87E-01 | 3.62E-01 | 5.14E-03 | 5.39E-03 | 6.97E-03 | 9.33E-01 |
|  | rs1233493 | *MAS1L* | 6 | 4.73E-03 | 2.97E-01 | 8.69E-01 | 4.68E-01 | 6.12E-03 | NA | 4.73E-03 | 8.83E-01 |
|  | rs1235162 | *GABBR1* | 6 | 2.15E-03 | 3.28E-01 | 8.69E-01 | 7.38E-01 | 2.15E-03 | NA | 2.43E-01 | 8.69E-01 |
|  | rs2735024 | *HLA-G* | 6 | 3.01E-02 | NA | NA | 3.01E-02 | NA | NA | NA | NA |
|  | rs9295826 | *HLA-G* | 6 | 4.23E-02 | NA | NA | 4.23E-02 | NA | NA | NA | NA |
|  | rs9393986 | *HLA-G* | 6 | 3.22E-02 | 8.71E-01 | 8.25E-02 | 4.23E-02 | NA | NA | 3.22E-02 | 9.28E-01 |
|  | rs2523992 | *TRIM31* | 6 | 1.32E-02 | 1.07E-01 | 6.63E-01 | 1.76E-02 | NA | NA | 1.32E-02 | 9.06E-01 |
|  | rs2844762 | *HLA-B* | 6 | 5.71E-03 | NA | 7.97E-01 | 5.71E-03 | 8.62E-03 | NA | 5.74E-03 | NA |
|  | rs3130380 | *HCG18* | 6 | 1.20E-02 | 1.99E-02 | 8.69E-01 | 5.60E-01 | 1.20E-02 | NA | 1.24E-02 | 8.21E-01 |
|  | rs3094694 | *HLA-E* | 6 | 1.02E-02 | NA | 8.87E-01 | 9.72E-01 | 1.02E-02 | 1.07E-02 | NA | NA |
|  | rs3095326 | *IER3* | 6 | 3.90E-03 | 1.67E-01 | 8.87E-01 | 6.72E-01 | NA | NA | 3.90E-03 | 9.56E-01 |
|  | rs9468830 | *C6orf214* | 6 | 5.38E-03 | 3.28E-01 | 8.87E-01 | 6.91E-03 | NA | NA | 5.38E-03 | 8.21E-01 |
|  | rs7752959 | *C6orf214* | 6 | 5.71E-03 | 4.48E-01 | 9.08E-01 | 5.71E-03 | NA | NA | 3.69E-01 | 6.67E-01 |
|  | rs13201769 | *C6orf214* | 6 | 6.49E-03 | 3.63E-01 | 8.69E-01 | 8.34E-03 | 1.20E-02 | 1.26E-02 | 6.49E-03 | 8.46E-01 |
|  | rs1264344 | *C6orf214* | 6 | 9.10E-04 | 1.90E-01 | 9.68E-01 | 9.94E-04 | 9.56E-01 | 1.66E-03 | 9.10E-04 | 1.00E+00 |
|  | rs2530710 | *MUC21* | 6 | 1.32E-02 | 8.81E-01 | 9.08E-01 | 1.76E-02 | 2.27E-02 | NA | 1.32E-02 | 8.83E-01 |
|  | rs1634731 | *MUC21* | 6 | 1.57E-02 | 9.56E-01 | 8.88E-01 | 2.11E-02 | 2.66E-02 | 2.81E-02 | 1.57E-02 | 8.75E-01 |
|  | rs3869098 | *HCG22* | 6 | 1.82E-04 | NA | NA | 1.82E-04 | NA | NA | NA | NA |
|  | rs2248386 | *HCG22* | 6 | 2.87E-04 | 2.97E-01 | 8.40E-01 | 5.60E-01 | 5.60E-04 | 5.03E-04 | 2.87E-04 | 8.60E-01 |
|  | rs3130544 | *C6orf15* | 6 | 2.77E-07 | 2.67E-01 | 9.68E-01 | 3.11E-01 | 2.77E-07 | NA | 3.91E-02 | 8.60E-01 |
|  | rs9263565 | *C6orf15* | 6 | 9.75E-05 | NA | NA | 9.75E-05 | NA | NA | NA | NA |
|  | rs1265048 | *C6orf15* | 6 | 3.10E-03 | 1.46E-01 | 4.46E-01 | 3.88E-03 | 6.12E-03 | 6.46E-03 | 3.10E-03 | 9.93E-01 |
|  | rs3132550 | *PSORS1C1* | 6 | 2.50E-06 | 6.10E-01 | 8.87E-01 | 2.50E-06 | NA | NA | 8.44E-02 | 9.56E-01 |
|  | rs3095324 | *PSORS1C1* | 6 | 5.67E-05 | 7.89E-01 | 8.87E-01 | 5.14E-01 | 5.67E-05 | NA | 3.90E-03 | 9.93E-01 |
|  | rs3130991 | *PSORS1C1* | 6 | 2.10E-06 | 4.03E-01 | 2.79E-02 | 7.38E-01 | 4.80E-01 | NA | 2.10E-06 | 9.06E-01 |
|  | rs3094205 | *PSORS1C1* | 6 | 2.10E-06 | 3.63E-01 | 2.50E-02 | 7.38E-01 | 4.80E-01 | 4.66E-06 | 2.10E-06 | 9.06E-01 |
|  | rs3094204 | *PSORS1C1* | 6 | 3.71E-03 | 1.67E-01 | 7.57E-03 | 1.29E-02 | 5.88E-02 | 7.68E-03 | 3.71E-03 | 8.46E-01 |
|  | rs3778639 | *PSORS1C1* | 6 | 6.49E-03 | 1.16E-02 | 1.94E-01 | 8.34E-03 | NA | NA | 6.49E-03 | 6.18E-01 |
|  | rs1265098 | *PSORS1C1* | 6 | 1.51E-05 | 1.67E-01 | 7.49E-01 | 1.51E-05 | 4.03E-05 | 3.09E-05 | 4.06E-05 | 9.53E-01 |
|  | rs1966 | *PSORS1C1* | 6 | 6.42E-07 | 8.37E-01 | 2.57E-01 | 1.62E-06 | 2.71E-05 | 1.52E-06 | 6.42E-07 | 9.93E-01 |
|  | rs1265110 | *CCHCR1* | 6 | 9.10E-04 | 2.14E-01 | 1.47E-02 | 9.94E-04 | 1.79E-03 | 3.09E-02 | 9.10E-04 | 9.56E-01 |
|  | rs2073717 | *CCHCR1* | 6 | 1.09E-03 | 6.10E-01 | 5.37E-01 | 4.16E-01 | NA | NA | 1.09E-03 | 9.56E-01 |
|  | rs2239524 | *CCHCR1* | 6 | 1.53E-03 | 5.52E-01 | 4.91E-01 | 3.62E-01 | 3.06E-03 | 3.04E-03 | 1.53E-03 | 9.56E-01 |
|  | rs879882 | *POU5F1* | 6 | 1.82E-03 | 1.26E-01 | 4.46E-01 | 2.16E-03 | 3.64E-03 | 3.70E-03 | 1.82E-03 | 9.56E-01 |
|  | rs9263871 | *HCG27* | 6 | 1.93E-04 | 1.14E-02 | 1.00E+00 | 4.11E-02 | 3.84E-04 | 3.39E-04 | 1.93E-04 | 9.56E-01 |
|  | rs2853950 | *HLA-C* | 6 | 1.39E-03 | NA | NA | 1.39E-03 | NA | NA | NA | NA |
|  | rs9264601 | *HLA-C* | 6 | 1.93E-02 | NA | NA | 1.93E-02 | NA | NA | NA | NA |
|  | rs9264603 | *HLA-C* | 6 | 1.29E-02 | NA | NA | 1.29E-02 | NA | NA | NA | NA |
|  | rs2074489 | *HLA-B* | 6 | 3.54E-06 | NA | NA | 3.54E-06 | NA | NA | NA | NA |
|  | rs2395471 | *HLA-B* | 6 | 9.35E-03 | 9.08E-02 | 3.65E-01 | 1.21E-02 | 1.66E-02 | 1.73E-02 | 9.35E-03 | 7.42E-01 |
|  | rs13200569 | *HLA-B* | 6 | 1.62E-06 | NA | NA | 1.62E-06 | NA | NA | NA | NA |
|  | rs2524074 | *HLA-B* | 6 | 7.55E-08 | 1.92E-03 | 7.49E-01 | 2.28E-01 | NA | NA | 7.55E-08 | 4.92E-02 |
|  | rs2894207 | *HLA-B* | 6 | 2.66E-02 | 5.43E-02 | 7.41E-02 | 3.57E-02 | 4.40E-02 | 4.66E-02 | 2.66E-02 | 8.39E-01 |
|  | rs9366778 | *HLA-B* | 6 | 2.78E-07 | 2.97E-01 | 7.03E-01 | 2.66E-01 | 4.26E-01 | 5.62E-07 | 2.78E-07 | 9.53E-01 |
|  | rs6931332 | *HLA-B* | 6 | 3.71E-03 | 5.52E-01 | 8.87E-01 | 4.71E-03 | 3.27E-01 | NA | 3.71E-03 | 6.67E-01 |
|  | rs2442719 | *HLA-B* | 6 | 1.86E-02 | 6.10E-02 | 1.00E+00 | 6.38E-01 | 3.13E-02 | 3.32E-02 | 1.86E-02 | 8.62E-01 |
|  | rs2596501 | *HLA-B* | 6 | 1.46E-02 | 1.67E-01 | 8.40E-01 | 1.46E-02 | 1.94E-02 | 2.02E-02 | 2.67E-02 | 9.56E-01 |
|  | rs9378249 | *HLA-B* | 6 | 2.63E-03 | NA | NA | 2.63E-03 | NA | NA | NA | NA |
|  | rs2523554 | *HLA-B* | 6 | 2.98E-03 | 2.98E-03 | 7.49E-01 | 8.83E-01 | 3.64E-03 | 3.70E-03 | 2.08E-01 | 5.03E-01 |
|  | rs2844575 | *HLA-B* | 6 | 1.21E-03 | 6.38E-02 | 5.83E-01 | 1.21E-03 | 4.80E-01 | 2.04E-03 | 3.69E-01 | 6.39E-01 |
|  | rs9266406 | *HLA-B* | 6 | 4.73E-02 | 9.69E-02 | 9.10E-01 | 5.78E-01 | 7.36E-02 | NA | 4.73E-02 | 8.48E-01 |
|  | rs2244020 | *MICA* | 6 | 1.11E-02 | 2.05E-02 | 8.69E-01 | 1.46E-02 | 2.17E-01 | NA | 1.11E-02 | 8.60E-01 |
|  | rs1131896 | *MICA* | 6 | 4.40E-02 | NA | NA | 1.00E+00 | 4.40E-02 | 4.66E-02 | NA | NA |
|  | rs2284178 | *HCP5* | 6 | 2.66E-02 | 3.91E-02 | 1.27E-01 | 9.95E-01 | 2.66E-02 | 2.81E-02 | 9.34E-01 | 8.87E-01 |
|  | rs2516440 | *HCG26* | 6 | 2.78E-04 | NA | NA | 2.78E-04 | 5.60E-04 | 5.03E-04 | NA | NA |
|  | rs2844503 | *HCG26* | 6 | 3.88E-03 | NA | NA | 3.88E-03 | NA | NA | NA | NA |
|  | rs12660382 | *HCG26* | 6 | 2.66E-02 | 3.77E-01 | 9.14E-01 | 3.57E-02 | 4.40E-02 | 4.66E-02 | 2.66E-02 | 9.65E-01 |
|  | rs3749946 | *HCG26* | 6 | 1.00E-02 | 1.40E-02 | 3.46E-02 | 1.00E-02 | 1.41E-02 | 1.47E-02 | 1.23E-01 | 1.00E+00 |
|  | rs3130637 | *MCCD1* | 6 | 1.46E-02 | 4.48E-01 | 2.61E-02 | 1.46E-02 | 1.94E-02 | 2.02E-02 | 1.50E-02 | 8.60E-01 |
|  | rs2259435 | *MCCD1* | 6 | 2.82E-05 | 1.63E-02 | 5.37E-01 | 2.82E-05 | 7.74E-05 | 5.92E-05 | 3.02E-05 | 9.33E-01 |
|  | rs2516478 | *BAT1* | 6 | 1.06E-05 | 1.99E-02 | 4.91E-01 | 1.06E-05 | 2.77E-05 | NA | 1.73E-05 | 9.33E-01 |
|  | rs2071592 | *NFKBIL1* | 6 | 4.71E-03 | NA | NA | 4.71E-03 | NA | NA | NA | NA |
|  | rs2230365 | *NFKBIL1* | 6 | 4.26E-04 | 5.94E-04 | 7.39E-03 | 4.26E-04 | 6.60E-01 | 7.46E-04 | 8.26E-01 | 9.33E-01 |
|  | rs9469027 | *LST1* | 6 | 2.16E-03 | NA | NA | 2.16E-03 | NA | NA | NA | NA |
|  | rs2844480 | *NCR3* | 6 | 6.56E-03 | 6.56E-03 | 9.68E-01 | 7.38E-01 | 1.22E-02 | 7.68E-03 | 6.98E-02 | 8.69E-01 |
|  | rs2857700 | *AIF1* | 6 | 1.21E-03 | 2.44E-02 | 1.00E+00 | 1.21E-03 | NA | NA | 2.67E-02 | 2.38E-01 |
|  | rs2261033 | *BAT2* | 6 | 1.86E-02 | 9.56E-01 | 4.76E-02 | 2.53E-02 | NA | NA | 1.86E-02 | 4.50E-01 |
|  | rs1077393 | *BAT3* | 6 | 1.32E-02 | 1.46E-01 | 9.08E-01 | 1.76E-02 | 2.27E-02 | 2.38E-02 | 1.32E-02 | 8.83E-01 |
|  | rs2844463 | *BAT3* | 6 | 9.94E-04 | 9.76E-03 | 7.03E-01 | 9.94E-04 | NA | 8.25E-01 | 1.51E-03 | 9.33E-01 |
|  | rs2142234 | *CSNK2B* | 6 | 2.09E-05 | 8.81E-01 | 8.69E-01 | 2.09E-05 | NA | NA | 2.12E-05 | 9.56E-01 |
|  | rs9267546 | *BAT5* | 6 | 3.54E-06 | NA | NA | 3.54E-06 | 8.20E-06 | 9.06E-01 | NA | NA |
|  | rs385306 | *BAT5* | 6 | 7.46E-04 | NA | NA | 8.91E-04 | 8.27E-04 | 7.46E-04 | NA | NA |
|  | rs1065356 | *LY6G6C* | 6 | 1.89E-04 | 3.63E-01 | 4.04E-01 | 6.13E-02 | 2.25E-04 | 1.89E-04 | 9.03E-01 | 7.42E-01 |
|  | rs480092 | *LSM2* | 6 | 7.80E-03 | 8.37E-01 | 4.04E-01 | 5.14E-01 | 1.41E-02 | 1.47E-02 | 7.80E-03 | 9.56E-01 |
|  | rs2227956 | *HSPA1L* | 6 | 6.29E-04 | 8.81E-01 | 6.24E-01 | 6.55E-04 | 1.22E-03 | 1.11E-03 | 6.29E-04 | 5.82E-01 |
|  | rs2075799 | *HSPA1L* | 6 | 2.22E-02 | 1.51E-01 | 5.84E-01 | 3.01E-02 | NA | NA | 2.22E-02 | 6.78E-01 |
|  | rs605203 | *SLC44A4* | 6 | 2.75E-08 | 1.67E-01 | 9.68E-01 | 2.75E-08 | NA | NA | 3.69E-01 | 1.59E-01 |
|  | rs659445 | *NG36/G9a* | 6 | 2.75E-08 | 2.67E-01 | 9.08E-01 | 2.75E-08 | 5.99E-01 | 8.03E-08 | 3.23E-01 | 1.38E-01 |
|  | rs7887 | *NG36/G9a* | 6 | 6.49E-03 | 5.52E-01 | 5.37E-01 | 8.34E-03 | NA | NA | 6.49E-03 | 9.06E-01 |
|  | rs7746553 | *C2* | 6 | 2.53E-02 | NA | NA | 2.53E-02 | NA | NA | NA | NA |
|  | rs440454 | *SKIV2L* | 6 | 2.75E-08 | 2.97E-01 | 5.39E-08 | 2.75E-08 | NA | NA | 6.19E-06 | 1.59E-01 |
|  | rs1150755 | *TNXB* | 6 | 1.11E-03 | 1.11E-03 | 4.04E-01 | 9.51E-01 | NA | NA | 6.97E-03 | 8.60E-01 |
|  | rs9267798 | *TNXB* | 6 | 6.91E-03 | 3.65E-02 | 1.14E-02 | 6.91E-03 | NA | NA | 1.02E-02 | 9.56E-01 |
|  | rs1150752 | *TNXB* | 6 | 1.87E-06 | 1.92E-03 | 6.24E-01 | 1.83E-04 | 1.87E-06 | NA | 2.20E-02 | 7.01E-01 |
|  | rs429150 | *TNXB* | 6 | 1.82E-03 | 5.52E-01 | 3.65E-01 | 9.51E-01 | NA | NA | 1.82E-03 | 7.42E-01 |
|  | rs3807039 | *ATF6B* | 6 | 1.76E-02 | 2.48E-02 | 4.91E-01 | 1.76E-02 | NA | NA | 8.44E-02 | 8.60E-01 |
|  | rs9296009 | *PRRT1* | 6 | 2.66E-02 | 5.43E-02 | 1.32E-01 | 3.57E-02 | 6.07E-02 | NA | 2.66E-02 | 8.86E-01 |
|  | rs3134603 | *PPT2* | 6 | 4.52E-05 | 9.56E-01 | 3.89E-02 | 4.52E-05 | 1.20E-04 | 9.40E-05 | 2.20E-02 | NA |
|  | rs204993 | *PBX2* | 6 | 1.57E-02 | 3.01E-02 | 3.91E-02 | 9.59E-01 | NA | NA | 1.57E-02 | 9.91E-01 |
|  | rs204991 | *GPSM3* | 6 | 3.01E-02 | 1.00E+00 | 5.77E-02 | 3.01E-02 | 3.71E-02 | NA | 3.11E-02 | 9.57E-01 |
|  | rs204989 | *GPSM3* | 6 | 1.46E-02 | 8.81E-01 | 2.61E-02 | 1.46E-02 | 1.94E-02 | NA | 3.91E-02 | 9.53E-01 |
|  | rs384247 | *NOTCH4* | 6 | 4.71E-03 | 6.56E-03 | 3.27E-01 | 4.71E-03 | NA | NA | 3.23E-01 | 1.00E+00 |
|  | rs438475 | *NOTCH4* | 6 | 3.57E-02 | 5.43E-02 | 6.94E-02 | 3.57E-02 | 4.40E-02 | NA | 2.46E-01 | 1.00E+00 |
|  | rs6906128 | *NOTCH4* | 6 | 1.11E-02 | 2.97E-01 | 8.69E-01 | 1.46E-02 | NA | NA | 1.11E-02 | 8.69E-01 |
|  | rs17576984 | *AK123889* | 6 | 1.76E-02 | 6.73E-01 | 9.08E-01 | 1.76E-02 | 4.98E-02 | 2.38E-02 | 4.77E-01 | 8.46E-01 |
|  | rs9267956 | *AK123889* | 6 | 1.27E-05 | 7.34E-01 | 4.04E-01 | 1.27E-05 | NA | NA | 3.69E-01 | 9.06E-01 |
|  | rs405875 | *AK123889* | 6 | 2.09E-05 | 3.28E-01 | 3.46E-02 | 2.09E-05 | 5.67E-05 | 4.33E-05 | 4.77E-01 | 9.06E-01 |
|  | rs9357138 | *AK123889* | 6 | 3.90E-02 | 8.82E-01 | 2.44E-01 | 5.00E-02 | NA | NA | 3.90E-02 | 9.88E-01 |
|  | rs9267992 | *AK123889* | 6 | 3.28E-05 | 4.98E-01 | 1.71E-02 | 3.28E-05 | 8.98E-05 | 6.89E-05 | 4.21E-01 | 6.39E-01 |
|  | rs3130320 | *AK123889* | 6 | 2.75E-08 | 7.34E-01 | 8.87E-01 | 2.75E-08 | 1.50E-07 | 8.03E-08 | 4.21E-01 | 3.84E-01 |
|  | rs9268220 | *C6orf10* | 6 | 3.83E-05 | 5.52E-01 | 1.25E-02 | 3.83E-05 | NA | NA | 4.29E-05 | 9.06E-01 |
|  | rs3129927 | *C6orf10* | 6 | 1.01E-07 | NA | NA | 1.01E-07 | NA | NA | NA | NA |
|  | rs2143462 | *C6orf10* | 6 | 2.43E-05 | 7.89E-01 | 1.47E-02 | 2.43E-05 | NA | NA | 2.53E-05 | 9.06E-01 |
|  | rs9268384 | *C6orf10* | 6 | 1.51E-05 | 1.00E+00 | 7.97E-01 | 1.51E-05 | NA | 3.09E-05 | 1.76E-01 | 9.06E-01 |
|  | rs9268402 | *C6orf10* | 6 | 3.71E-03 | 7.34E-01 | 7.57E-03 | 4.71E-03 | NA | NA | 3.71E-03 | 1.51E-02 |
|  | rs6930777 | *BTNL2* | 6 | 4.71E-03 | NA | 7.57E-03 | 4.71E-03 | 7.26E-03 | NA | NA | NA |
|  | rs17423649 | *BTNL2* | 6 | 1.18E-03 | 4.48E-01 | 1.18E-03 | 5.14E-01 | 1.49E-03 | NA | 5.76E-02 | 3.44E-01 |
|  | rs2294881 | *BTNL2* | 6 | 1.02E-02 | 7.63E-02 | 2.13E-02 | 7.38E-01 | NA | NA | 1.02E-02 | 7.01E-01 |
|  | rs5007265 | *BTNL2* | 6 | 5.38E-03 | 9.60E-03 | 1.14E-02 | 6.91E-03 | NA | NA | 5.38E-03 | 2.07E-02 |
|  | rs7759742 | *BTNL2* | 6 | 1.21E-03 | NA | NA | 1.21E-03 | NA | NA | NA | NA |
|  | rs3129883 | *HLA-DRA* | 6 | 3.71E-03 | 6.56E-03 | 7.57E-03 | 4.71E-03 | NA | NA | 3.71E-03 | 5.61E-01 |
|  | rs7192 | *HLA-DRA* | 6 | 1.82E-03 | 1.00E+00 | 5.14E-02 | 2.16E-03 | NA | 3.70E-03 | 1.82E-03 | 7.91E-03 |
|  | rs2858870 | *HLA-DRB5* | 6 | 1.11E-02 | NA | 2.61E-02 | NA | 1.94E-02 | NA | 1.11E-02 | 6.18E-01 |
|  | rs9272535 | *HLA-DQA1* | 6 | 2.16E-03 | NA | 5.99E-02 | 2.16E-03 | NA | NA | NA | NA |
|  | rs9273363 | *HLA-DQB1* | 6 | 1.47E-03 | NA | NA | 1.47E-03 | NA | NA | NA | NA |
|  | rs9469220 | *HLA-DQB1* | 6 | 1.86E-02 | 3.65E-02 | 4.76E-02 | 2.53E-02 | NA | NA | 1.86E-02 | 6.48E-01 |
|  | rs10484561 | *HLA-DQB1* | 6 | 8.34E-03 | 6.73E-01 | 1.41E-02 | 8.34E-03 | 1.20E-02 | 1.26E-02 | 2.08E-01 | 1.01E-01 |
|  | rs11752643 | *HLA-DQB1* | 6 | 2.75E-08 | NA | NA | 2.75E-08 | 1.50E-07 | 8.03E-08 | NA | NA |
|  | rs9275572 | *HLA-DQA2* | 6 | 2.17E-03 | 4.03E-01 | 6.63E-01 | 2.63E-03 | NA | 4.48E-03 | 2.17E-03 | 9.38E-03 |
|  | rs2858331 | *HLA-DQA2* | 6 | 7.80E-03 | 1.40E-02 | 1.73E-02 | 1.00E-02 | 1.41E-02 | 1.47E-02 | 7.80E-03 | 1.38E-01 |
|  | rs3892710 | *HLA-DQA2* | 6 | 1.78E-03 | 9.56E-01 | 5.83E-01 | 1.78E-03 | NA | 3.04E-03 | 9.31E-01 | 9.33E-01 |
|  | rs10947340 | *HLA-DQA2* | 6 | 3.57E-02 | NA | NA | 3.57E-02 | NA | NA | NA | NA |
|  | rs10807113 | *HLA-DQB2* | 6 | 3.57E-02 | 5.43E-02 | 6.94E-02 | 3.57E-02 | 4.40E-02 | 4.66E-02 | 1.50E-01 | 8.04E-01 |
|  | rs4148876 | *TAP2* | 6 | 2.82E-05 | 6.38E-02 | 4.51E-05 | 2.82E-05 | NA | 5.92E-05 | 4.77E-01 | 8.83E-01 |
|  | rs11756897 | *HLA-DMB* | 6 | 6.55E-04 | 1.00E+00 | 8.69E-01 | 6.55E-04 | NA | NA | 8.26E-01 | 9.56E-01 |
|  | rs23544 | *HLA-DMB* | 6 | 6.49E-03 | 6.10E-01 | 7.05E-02 | 8.34E-03 | NA | NA | 6.49E-03 | 9.93E-01 |
|  | rs1049414 | *BRD2* | 6 | 1.21E-02 | NA | NA | 1.21E-02 | NA | NA | NA | NA |
|  | rs9500927 | *HLA-DOA* | 6 | 3.01E-02 | 3.63E-01 | 5.77E-02 | 3.01E-02 | 3.71E-02 | NA | 4.55E-02 | 8.66E-01 |
| 150* | rs2076312 | *COL11A2* | 6 | 4.23E-02 | 1.00E+00 | 6.19E-01 | 4.23E-02 | NA | NA | 7.29E-01 | 8.72E-01 |
|  | rs3762013 | *COL11A2* | 6 | 2.66E-02 | 7.97E-01 | 9.66E-01 | 9.95E-01 | 2.66E-02 | 6.19E-02 | 9.24E-01 | 9.07E-01 |
|  | rs9277936 | *RING1* | 6 | 2.16E-03 | NA | NA | 2.16E-03 | NA | NA | NA | NA |
| 151* | rs213220 | *VPS52* | 6 | 3.13E-02 | 5.19E-01 | 6.51E-01 | 2.83E-01 | 3.13E-02 | 3.32E-02 | 9.31E-01 | 9.55E-01 |
|  | rs462093 | *WDR46* | 6 | 1.78E-03 | NA | NA | 1.78E-03 | NA | NA | NA | NA |
|  | rs3106192 | *LYPLA2* | 6 | 3.13E-02 | 5.71E-01 | 9.64E-01 | 7.60E-01 | 3.13E-02 | 3.32E-02 | 9.31E-01 | 1.00E+00 |
| 162* | rs13196377 | *TRAF3IP2* | 6 | 3.22E-02 | 2.21E-01 | 8.25E-02 | 8.57E-01 | NA | NA | 3.22E-02 | 8.90E-01 |
| 163 | rs13203813 | *SART-2* | 6 | 4.26E-02 | 8.03E-01 | 7.62E-01 | 9.65E-01 | 8.80E-01 | 4.26E-02 | 9.17E-01 | 9.33E-01 |
| 165 | rs7748718 | *TCBA1* | 6 | 2.66E-02 | NA | 1.00E+00 | 5.67E-01 | 9.62E-01 | 1.00E+00 | 2.66E-02 | 9.02E-01 |
| 166 | rs2503322 | *RSPO3* | 6 | 2.28E-03 | 2.28E-03 | 8.69E-01 | 9.72E-01 | 4.26E-01 | 5.62E-01 | 9.03E-01 | 9.93E-01 |
| 167 | rs10499166 | *C6orf191* | 6 | 4.40E-02 | 8.61E-01 | 7.15E-01 | 9.75E-01 | 4.40E-02 | 3.29E-01 | 9.44E-01 | 8.86E-01 |
| 168 | rs6902109 | *L3MBTL3* | 6 | 2.02E-02 | 4.98E-01 | 7.03E-01 | 4.16E-01 | 6.60E-01 | 2.02E-02 | 9.03E-01 | 9.93E-01 |
| 169 | rs9494868 | *AK124173* | 6 | 4.23E-02 | 9.59E-01 | 7.94E-01 | 4.23E-02 | 8.17E-01 | 5.33E-01 | 7.81E-01 | 9.72E-01 |
| 192 | rs1178979 | *BAZ1B* | 7 | 3.65E-02 | 3.65E-02 | 7.97E-01 | 9.72E-01 | NA | NA | 9.80E-01 | 9.56E-01 |
| 203 | rs10275417 | *GIMAP2* | 7 | 2.22E-02 | 1.97E-01 | 9.11E-01 | 9.70E-01 | 9.60E-01 | 9.85E-01 | 2.22E-02 | 8.95E-01 |
| 205 | rs4841042 | *MFHAS1* | 8 | 1.88E-02 | 9.56E-01 | 7.03E-01 | 9.56E-01 | 1.88E-02 | NA | 1.00E+00 | 9.93E-01 |
|  | rs430949 | *AK055863* | 8 | 4.30E-02 | NA | 6.87E-01 | 9.65E-01 | 8.80E-01 | NA | 4.30E-02 | 9.58E-01 |
|  | rs3021494 | *XKR6* | 8 | 1.53E-03 | 6.73E-01 | 8.40E-01 | 9.72E-01 | NA | NA | 1.53E-03 | 9.56E-01 |
|  | rs9644737 | *C8orf12* | 8 | 9.79E-03 | NA | 8.87E-01 | 9.56E-01 | 9.79E-03 | 1.00E+00 | 9.80E-01 | 9.93E-01 |
|  | rs998683 | *BLK* | 8 | 8.31E-03 | 5.52E-01 | 7.97E-01 | 8.31E-03 | 2.85E-01 | NA | 9.31E-01 | 8.60E-01 |
|  | rs2029969 | *GATA4* | 8 | 1.66E-02 | 8.37E-01 | 8.87E-01 | 8.83E-01 | 1.66E-02 | 8.71E-01 | 9.18E-01 | 9.33E-01 |
|  | rs1466785 | *C8orf49* | 8 | 1.52E-02 | 6.73E-01 | 6.24E-01 | 9.72E-01 | 1.52E-02 | 9.83E-01 | 9.80E-01 | 9.93E-01 |
|  | rs1296023 | *CTSB* | 8 | 5.14E-03 | 1.00E+00 | 2.79E-02 | 9.87E-01 | 5.14E-03 | NA | 9.80E-01 | 9.93E-01 |
|  | rs4841645 | *DEFB136* | 8 | 3.47E-02 | 7.89E-01 | 8.69E-01 | 9.56E-01 | 3.47E-02 | NA | 7.74E-01 | 9.06E-01 |
| 207 | rs1961456 | *NAT2* | 8 | 3.40E-03 | 7.34E-01 | 8.87E-01 | 3.40E-03 | NA | NA | 6.58E-01 | 8.21E-01 |
| 211 | rs2278615 | *INTS10* | 8 | 8.34E-03 | NA | NA | 8.34E-03 | NA | NA | NA | NA |
| 212 | rs4128744 | *SLC18A1* | 8 | 3.47E-02 | 7.89E-01 | 1.00E+00 | 5.60E-01 | 3.47E-02 | 8.50E-01 | 9.31E-01 | 9.33E-01 |
| 224 | rs10216900 | *CR590356* | 8 | 4.40E-02 | 1.00E+00 | 6.82E-01 | 9.75E-01 | 4.40E-02 | 8.99E-01 | 9.79E-01 | 1.00E+00 |
| 225 | rs2288310 | *KIAA0196* | 8 | 1.52E-02 | NA | NA | 9.87E-01 | 1.52E-02 | 9.83E-01 | NA | NA |
| 227 | rs17406109 | *TRIB1* | 8 | 3.89E-02 | 8.37E-01 | 3.89E-02 | 9.56E-01 | 1.00E+00 | 8.50E-01 | 1.00E+00 | 7.42E-01 |
| 228 | rs7002027 | *TRIB1* | 8 | 1.99E-02 | 1.99E-02 | 6.24E-01 | 9.72E-01 | NA | NA | 9.18E-01 | 6.67E-01 |
| 229 | rs4871618 | *TRIB1* | 8 | 2.96E-02 | NA | NA | 2.96E-02 | NA | NA | NA | NA |
| 233 | rs953586 | *BNC2* | 9 | 3.93E-02 | 8.91E-01 | 9.11E-01 | 8.36E-01 | 8.41E-01 | 3.93E-02 | 1.00E+00 | 7.27E-01 |
| 236 | rs13284229 | *KIF24* | 9 | 1.66E-02 | 8.37E-01 | 1.00E+00 | 9.87E-01 | 1.66E-02 | NA | 5.97E-01 | 1.00E+00 |
| 238 | rs1006280 | *PCSK5* | 9 | 4.45E-02 | 4.45E-02 | 7.34E-01 | 9.99E-01 | 3.56E-01 | NA | 9.26E-01 | 9.89E-01 |
| 247 | rs3812591 | *SEC16A* | 9 | 2.05E-02 | 2.05E-02 | 2.61E-02 | 9.80E-01 | 8.16E-01 | NA | 5.97E-01 | 9.93E-01 |
| 248 | rs7906894 | *AKR1C4* | 10 | 3.09E-02 | 1.00E+00 | 8.87E-01 | 9.87E-01 | 4.26E-01 | 3.09E-02 | 1.00E+00 | 9.56E-01 |
| 251 | rs11255317 | *ITIH2* | 10 | 3.90E-02 | 8.82E-01 | 9.63E-01 | 9.75E-01 | NA | NA | 3.90E-02 | 9.88E-01 |
| 257 | rs11101342 | *ARHGAP22* | 10 | 3.91E-02 | NA | 9.68E-01 | 9.72E-01 | 6.60E-01 | 6.21E-01 | 3.91E-02 | 1.00E+00 |
| 258 | rs10821944 | *ARID5B* | 10 | 3.57E-02 | 8.61E-01 | 2.50E-01 | 3.57E-02 | 2.88E-01 | 7.99E-01 | 5.37E-01 | 9.65E-01 |
|  | rs12357548 | *ARID5B* | 10 | 3.57E-02 | 9.57E-01 | 7.15E-01 | 3.57E-02 | 4.40E-02 | 4.69E-01 | 8.64E-02 | 9.88E-01 |
| 261 | rs11187157 | *HHEX* | 10 | 1.14E-02 | NA | 1.14E-02 | 9.72E-01 | NA | NA | 9.03E-01 | 9.56E-01 |
| 262 | rs10509646 | *HHEX* | 10 | 1.47E-02 | 2.40E-01 | 1.47E-02 | 6.13E-01 | 5.99E-01 | 9.83E-01 | 7.74E-01 | 9.56E-01 |
| 274 | rs1060573 | *C11orf49* | 11 | 4.98E-02 | 8.81E-01 | 9.08E-01 | 8.08E-01 | 4.98E-02 | 8.71E-01 | 9.80E-01 | 9.53E-01 |
| 277 | rs102275 | *C11orf10* | 11 | 2.07E-04 | 2.07E-04 | 7.97E-01 | 3.62E-01 | 4.26E-01 | 2.07E-01 | 8.71E-01 | 8.69E-01 |
|  | rs968567 | *FADS2* | 11 | 5.69E-04 | 1.46E-01 | 9.08E-01 | 5.69E-04 | 2.17E-01 | 2.07E-01 | 9.31E-01 | 8.69E-01 |
|  | rs174634 | *FADS3* | 11 | 1.93E-02 | NA | NA | 1.93E-02 | NA | NA | NA | NA |
| 280 | rs7479857 | *FGF3* | 11 | 3.85E-02 | 7.34E-01 | 9.68E-01 | 9.56E-01 | 6.60E-01 | 3.85E-02 | 1.76E-01 | 8.60E-01 |
| 281 | rs11607061 | *TMEM135* | 11 | 3.39E-02 | NA | NA | 3.39E-02 | NA | NA | NA | NA |
| 284 | rs11605293 | *BUD13* | 11 | 2.23E-02 | NA | 2.23E-02 | 9.87E-01 | 6.60E-01 | 4.03E-01 | 9.18E-01 | 8.69E-01 |
| 288 | rs7120515 | *AB231710* | 11 | 2.85E-02 | 9.56E-01 | 7.49E-01 | 1.64E-01 | 2.85E-02 | NA | 8.71E-01 | 9.56E-01 |
| 296 | rs10842564 | *IFLTD1* | 12 | 4.11E-02 | 1.00E+00 | 8.69E-01 | 4.11E-02 | 8.16E-01 | 8.50E-01 | 6.58E-01 | 9.56E-01 |
| 299 | rs447649 | *NELL2* | 12 | 2.66E-02 | NA | 6.02E-01 | 6.70E-01 | NA | NA | 2.66E-02 | 9.44E-01 |
| 303 | rs3809114 | *INHBE* | 12 | 2.50E-02 | 1.00E+00 | 2.50E-02 | 6.13E-01 | 4.98E-02 | 8.84E-02 | 1.00E+00 | 9.56E-01 |
| 307 | rs3847814 | *PLXNC1* | 12 | 4.55E-02 | 8.54E-01 | 9.11E-01 | 1.00E+00 | NA | NA | 4.55E-02 | 1.00E+00 |
| 311 | rs7297186 | *CUX2* | 12 | 8.62E-03 | NA | 8.87E-01 | 6.13E-01 | 8.62E-03 | 1.98E-02 | 9.80E-01 | 1.00E+00 |
|  | rs7398833 | *CUX2* | 12 | 6.12E-03 | NA | 8.87E-01 | 5.60E-01 | 6.12E-03 | NA | 1.00E+00 | 1.00E+00 |
| 323 | rs34872609 | *LIG4* | 13 | 4.73E-02 | NA | 9.10E-01 | 9.38E-01 | 9.08E-01 | NA | 4.73E-02 | 9.84E-01 |
| 339 | rs4983425 | *C14orf80* | 14 | 4.77E-02 | NA | 3.19E-01 | 9.84E-01 | 1.00E+00 | NA | 4.77E-02 | 1.00E+00 |
| 345 | rs2696089 | *FRMD5* | 15 | 3.39E-02 | 7.34E-01 | 9.08E-01 | 3.39E-02 | NA | NA | 9.31E-01 | 9.56E-01 |
| 347 | rs7178779 | *ALDH1A2* | 15 | 4.11E-02 | NA | NA | 4.11E-02 | NA | NA | NA | NA |
| 352 | rs11071759 | *HERC1* | 15 | 3.47E-02 | 4.03E-01 | 8.40E-01 | 9.72E-01 | 3.47E-02 | 3.13E-01 | 1.00E+00 | 9.33E-01 |
|  | rs7180823 | *HERC1* | 15 | 1.47E-02 | 8.37E-01 | 9.08E-01 | 1.00E+00 | 4.26E-01 | 1.47E-02 | 9.18E-01 | 8.46E-01 |
| 363 | rs7197475 | *PRR14* | 16 | 3.11E-02 | 3.99E-01 | 7.71E-01 | 8.69E-02 | 6.27E-01 | 8.68E-01 | 3.11E-02 | 9.89E-01 |
|  | rs8062719 | *STX1B* | 16 | 6.29E-04 | 8.37E-01 | 6.24E-01 | 1.00E+00 | NA | NA | 6.29E-04 | 9.56E-01 |
| 364 | rs11643718 | *SLC12A3* | 16 | 3.63E-02 | 5.62E-01 | 8.00E-01 | 9.87E-01 | 3.63E-02 | 8.83E-01 | 9.09E-01 | 9.56E-01 |
| 368 | rs2000999 | *TXNL4B* | 16 | 2.85E-02 | 8.81E-01 | 8.69E-01 | 9.56E-01 | 2.85E-02 | 9.83E-01 | 9.80E-01 | 9.53E-01 |
| 369 | rs4788706 | *HTA* | 16 | 4.73E-02 | 9.63E-01 | 8.18E-01 | 1.00E+00 | NA | NA | 4.73E-02 | 9.23E-01 |
| 375 | rs8045991 | *JPH3* | 16 | 4.98E-02 | 6.73E-01 | 4.91E-01 | 7.38E-01 | 4.98E-02 | 9.83E-01 | 7.74E-01 | 9.56E-01 |
| 391 | rs7221651 | *BPTF* | 17 | 7.85E-03 | 8.81E-01 | 1.00E+00 | 9.87E-01 | 7.85E-03 | NA | 9.80E-01 | 1.00E+00 |
| 393 | rs523927 | *PTPRM* | 18 | 3.23E-02 | NA | 8.40E-01 | 1.00E+00 | 7.70E-01 | 6.82E-01 | 3.23E-02 | NA |
| 402 | rs12955986 | *DSEL* | 18 | 2.80E-02 | 7.34E-01 | 4.46E-01 | 2.80E-02 | 8.69E-01 | 8.25E-01 | 7.74E-01 | 9.56E-01 |
| 404 | rs17253937 | *INSR* | 19 | 2.32E-02 | 2.40E-01 | 1.00E+00 | 3.62E-01 | 2.32E-02 | 6.21E-01 | 9.80E-01 | 9.53E-01 |
| 406 | rs3826803 | *DNM2* | 19 | 3.22E-02 | 9.59E-01 | 1.00E+00 | 9.75E-01 | NA | NA | 3.22E-02 | 9.28E-01 |
| 409 | rs2965185 | *GATAD2A* | 19 | 2.18E-03 | 8.37E-01 | 9.68E-01 | 2.18E-03 | NA | NA | 9.03E-01 | 9.33E-01 |
| 413 | rs2285515 | *FXYD5* | 19 | 3.13E-02 | 8.49E-01 | 9.64E-01 | 9.94E-01 | 3.13E-02 | 5.40E-01 | 9.81E-01 | 9.56E-01 |
| 421 | rs307896 | *SAE1* | 19 | 4.17E-02 | NA | 6.63E-01 | 9.56E-01 | 4.17E-02 | 9.06E-01 | 9.80E-01 | NA |
| 423 | rs918434 | *CCDC9* | 19 | 3.57E-02 | NA | NA | 3.57E-02 | NA | NA | NA | NA |
| 425 | rs676388 | *FUT2* | 19 | 2.00E-03 | 2.00E-03 | 1.00E+00 | 1.00E+00 | 2.57E-03 | 1.07E-01 | 1.23E-01 | 6.18E-01 |
|  | rs838133 | *FGF21* | 19 | 1.32E-02 | 4.98E-01 | 9.08E-01 | 9.80E-01 | 2.85E-02 | 5.06E-01 | 1.32E-02 | 8.69E-01 |
| 432 | rs6060491 | *FER1L4* | 20 | 4.66E-02 | NA | NA | 9.75E-01 | 9.62E-01 | 4.66E-02 | NA | NA |
| 436 | rs6073246 | *TOX2* | 20 | 3.13E-02 | 5.19E-01 | 8.71E-01 | 9.73E-01 | 3.13E-02 | NA | 8.89E-01 | 8.62E-01 |
| 437 | rs4810482 | *MMP9* | 20 | 4.42E-02 | 4.42E-02 | 8.69E-01 | 5.60E-01 | 8.16E-01 | 9.83E-01 | 9.80E-01 | 9.93E-01 |
| 442 | rs2836753 | *ETS2* | 21 | 2.99E-02 | 2.99E-02 | 5.37E-01 | 6.13E-01 | 8.69E-01 | NA | 2.81E-01 | 1.00E+00 |
| 444 | rs1946990 | *DMC1* | 22 | 1.59E-02 | 1.26E-01 | 9.68E-01 | 1.59E-02 | 4.80E-01 | NA | 1.00E+00 | 8.21E-01 |
| 445 | rs5769941 | *BC037788* | 22 | 2.20E-02 | 7.34E-01 | 8.87E-01 | 1.00E+00 | 1.00E+00 | 8.71E-01 | 2.20E-02 | 9.53E-01 |

Independent complex or single gene loci (r^2^ < 0.2) with SNP(s) with a conjunctional FDR (conjFDR) < 0.05 in *both* triglycerides (TG) *and* in the associated immune-mediated disease. All SNPs with a conjFDR value < 0.05 (bidirectional association, i.e. association with the lipid phenotype given the immune mediated disease (condFDR< 0.05) and association with immune-mediated disease given the lipid phenotype (condFDR<0.05)) are listed and sorted in each LD block. For TG and the immune-mediated disease phenotype we defined the most significant SNP in each LD block based on the minimum conjFDR (min conjFDR). For comparison, the conjFDR values for each identified SNP are listed for all phenotypes, and the minimum (min) conjFDR across all phenotypes. All independent loci are listed consecutively, and Major Histocomaptibility Complex loci are marked (*).All data were first corrected for genomic inflation. Crohn’s Disease (CD), ulcerative colitis (UC), rheumatoid arthritis (RA), type 1 diabetes (T1D), celiac disease (CeD), psoriasis (PSOR) and sarcoidosis (SARC). Chromosome (Chr). NA; not available.

### Table I. Pleiotropic SNPs in low density lipoprotein (LDL) and immune mediated diseases (conjFDR<0.05).

| **#Locus** | **SNP** | ***gene*** | **chr** | **Min conjFDR** | **LDL & CD** | **LDL & UC** | **LDL & RA** | **LDL & T1D** | **LDL & CeD** | **LDL & PSOR** | **LDL & SARC** |
| --- | --- | --- | --- | --- | --- | --- | --- | --- | --- | --- | --- |
| 23 | rs4409689 | DOCK7 | 1 | 6.11E-03 | 6.11E-03 | 9.29E-01 | 8.85E-01 | NA | 2.72E-01 | 9.50E-01 | 9.61E-01 |
| 26 | rs11209050 | IL12RB2 | 1 | 1.59E-02 | 1.59E-02 | 1.77E-01 | 5.16E-01 | NA | NA | 9.50E-01 | 9.99E-01 |
| 27 | rs7528377 | RPAP2 | 1 | 4.89E-02 | 4.89E-02 | 7.66E-01 | 7.75E-01 | NA | NA | 9.60E-01 | 1.00E+00 |
| 33 | rs10494097 | STXBP3 | 1 | 2.31E-02 | 8.97E-01 | 9.86E-01 | 8.85E-01 | 2.31E-02 | 8.57E-01 | 9.60E-01 | 8.77E-01 |
| 34 | rs679224 | KIAA1324 | 1 | 2.39E-02 | NA | 5.48E-01 | 6.10E-01 | 2.39E-02 | 2.37E-01 | 9.43E-01 | 9.99E-01 |
| 35 | rs646776 | CELSR2 | 1 | 3.03E-02 | 8.63E-01 | 8.65E-01 | 5.63E-01 | 1.26E-01 | 3.03E-02 | 9.47E-01 | 9.25E-01 |
| 38 | rs2938616 | EPS8L3 | 1 | 4.76E-03 | 4.94E-01 | 4.76E-03 | 9.60E-01 | 9.68E-01 | 8.57E-01 | 9.60E-01 | 9.56E-01 |
| 44 | rs12746613 | FCGR2A | 1 | 1.92E-02 | 4.56E-01 | 7.06E-01 | 1.92E-02 | 1.00E+00 | NA | 9.87E-01 | 9.61E-01 |
|  | rs7551957 | FCGR2A | 1 | 4.31E-03 | NA | NA | 4.31E-03 | NA | NA | NA | NA |
|  | rs1801274 | FCGR2A | 1 | 8.82E-03 | 2.87E-01 | 1.98E-02 | 8.82E-03 | 1.00E+00 | 8.96E-01 | 5.61E-01 | 9.56E-01 |
| 48 | rs4652420 | TDRD5 | 1 | 4.31E-02 | 9.04E-01 | 1.00E+00 | 9.31E-01 | 7.85E-01 | NA | 4.31E-02 | 9.70E-01 |
| 49 | rs2297909 | KIF21B | 1 | 4.87E-02 | 6.43E-02 | 1.04E-01 | 3.89E-01 | 2.01E-01 | 4.87E-02 | 1.00E+00 | 8.95E-01 |
| 50 | rs6679953 | SNRPE | 1 | 4.58E-02 | NA | NA | 4.58E-02 | NA | NA | NA | NA |
| 58 | rs6738346 | RHOB | 2 | 1.64E-02 | 9.71E-01 | 8.20E-01 | 8.85E-01 | NA | NA | 1.64E-02 | 9.56E-01 |
| 64 | rs1260326 | GCKR | 2 | 1.24E-02 | 1.24E-02 | 7.83E-02 | 7.13E-01 | 3.50E-01 | 7.22E-01 | 9.60E-01 | 9.57E-01 |
| 80 | rs4309580 | INSIG2 | 2 | 4.15E-02 | 6.35E-01 | 9.86E-01 | 4.65E-02 | 4.15E-02 | 7.22E-01 | 1.00E+00 | 9.61E-01 |
|  | rs4405778 | INSIG2 | 2 | 3.31E-02 | NA | NA | 3.31E-02 | NA | NA | NA | NA |
| 82 | rs2011946 | CXCR4 | 2 | 1.35E-02 | 8.97E-01 | 8.20E-01 | 4.24E-01 | 1.35E-02 | 2.47E-02 | NA | 9.56E-01 |
|  | rs932206 | CXCR4 | 2 | 4.31E-03 | 9.12E-01 | 5.08E-01 | 5.63E-01 | 2.70E-01 | 4.31E-03 | 9.17E-01 | 9.99E-01 |
| 99 | rs9809905 | PPARG | 3 | 4.04E-02 | 3.34E-01 | 7.73E-01 | 5.99E-01 | 4.04E-02 | 4.83E-01 | 9.86E-01 | 9.66E-01 |
|  | rs299639 | TSEN2 | 3 | 5.96E-03 | 5.36E-01 | 1.00E+00 | 3.41E-01 | 5.96E-03 | 1.00E+00 | 9.87E-01 | 9.99E-01 |
|  | rs9809501 | RAF1 | 3 | 3.66E-02 | 9.13E-01 | 9.77E-01 | 8.50E-01 | 3.66E-02 | 8.53E-01 | 1.00E+00 | 9.63E-01 |
|  | rs2055312 | RAF1 | 3 | 1.03E-02 | NA | NA | 1.03E-02 | NA | NA | NA | NA |
| 108 | rs6779094 | HHLA2 | 3 | 2.99E-02 | 8.88E-01 | 6.47E-01 | 9.07E-01 | 2.99E-02 | 8.61E-01 | 9.62E-01 | 9.60E-01 |
| 124 | rs10517086 | C4orf52 | 4 | 2.70E-02 | 4.57E-01 | 4.30E-01 | 2.94E-02 | 2.70E-02 | 8.61E-01 | 9.49E-01 | 1.00E+00 |
|  | rs7441808 | C4orf52 | 4 | 1.88E-02 | NA | NA | 1.88E-02 | NA | NA | NA | NA |
| 129 | rs974801 | TET2 | 4 | 1.30E-02 | 1.30E-02 | 7.83E-02 | 9.60E-01 | 5.38E-01 | 8.33E-01 | 9.58E-01 | 9.99E-01 |
| 135 | rs11134149 | KIAA1519 | 5 | 3.34E-02 | 6.35E-01 | 3.34E-02 | 5.63E-01 | NA | NA | 3.62E-01 | 9.62E-01 |
| 136 | rs6878664 | CTNND2 | 5 | 7.58E-03 | 8.97E-01 | 9.86E-01 | 7.58E-03 | 5.38E-01 | 2.37E-01 | 1.00E+00 | 9.99E-01 |
| 138 | rs1553577 | PTGER4 | 5 | 1.07E-02 | 1.07E-02 | 3.32E-01 | 9.60E-01 | 5.38E-01 | NA | 9.17E-01 | 9.62E-01 |
| 151 | rs162889 | BC030525 | 5 | 3.90E-02 | 3.90E-02 | 8.19E-01 | 9.07E-01 | 6.49E-01 | 9.76E-01 | 8.86E-01 | 9.64E-01 |
|  | rs273909 | SLC22A4 | 5 | 1.07E-02 | 1.07E-02 | 7.06E-01 | 9.17E-01 | 8.43E-01 | 1.00E+00 | 9.43E-01 | 9.62E-01 |
|  | rs9282763 | LOC441108 | 5 | 2.44E-03 | 2.44E-03 | 3.92E-01 | 6.60E-01 | NA | NA | 9.87E-01 | 9.62E-01 |
|  | rs4705952 | IRF1 | 5 | 4.31E-02 | 2.63E-01 | 7.84E-01 | 8.97E-01 | NA | NA | 4.31E-02 | 1.00E+00 |
| 155 | rs7724832 | TIMD4 | 5 | 4.32E-03 | 4.32E-03 | 5.08E-01 | 7.75E-01 | 5.38E-01 | NA | 9.58E-01 | 8.46E-01 |
| 162 | rs2038037 | MYLIP | 6 | 1.35E-02 | 7.56E-01 | 9.29E-01 | 1.00E+00 | 1.35E-02 | 8.33E-01 | 6.30E-01 | 9.61E-01 |
| 163 | rs10498691 | AK097585 | 6 | 3.15E-02 | 1.00E+00 | 7.66E-01 | 9.58E-01 | 3.15E-02 | 9.74E-01 | 8.53E-01 | 9.62E-01 |
| 166* | rs12216125 | TRIM38 | 6 | 6.50E-03 | 8.18E-01 | 9.29E-01 | 7.13E-01 | 6.50E-03 | 8.86E-03 | 9.43E-01 | 9.46E-01 |
|  | rs16891235 | HIST1H1A | 6 | 7.90E-03 | 9.71E-01 | 9.00E-01 | 5.63E-01 | 7.90E-03 | 1.05E-02 | 9.50E-01 | 1.00E+00 |
|  | rs13194984 | BTN1A1 | 6 | 1.08E-03 | 9.12E-01 | 5.48E-01 | 8.85E-01 | 1.08E-03 | 4.51E-02 | 9.60E-01 | 8.77E-01 |
|  | rs13194491 | C6orf41 | 6 | 3.61E-03 | NA | NA | 1.00E+00 | 3.61E-03 | 5.19E-03 | NA | NA |
|  | rs13214703 | OR2B6 | 6 | 3.61E-03 | NA | NA | 1.00E+00 | 3.61E-03 | 6.73E-03 | NA | NA |
|  | rs11757000 | GPX6 | 6 | 3.66E-02 | 2.59E-01 | 3.20E-01 | 8.00E-01 | 3.66E-02 | 4.20E-02 | 8.91E-01 | 9.95E-01 |
| 167* | rs17508548 | GABBR1 | 6 | 3.15E-02 | 1.00E+00 | 5.98E-01 | 3.41E-02 | 3.15E-02 | NA | 3.18E-01 | 9.96E-01 |
|  | rs29267 | GABBR1 | 6 | 2.54E-02 | 1.00E+00 | 5.93E-01 | 2.54E-02 | NA | NA | 2.30E-01 | 9.99E-01 |
|  | rs3130253 | MOG | 6 | 4.25E-02 | 6.02E-01 | 3.80E-01 | 2.14E-01 | 4.25E-02 | 2.67E-01 | 9.67E-01 | 9.70E-01 |
|  | rs16896081 | HLA-G | 6 | 3.66E-02 | 6.76E-02 | 9.77E-01 | 4.56E-01 | 3.66E-02 | 4.20E-02 | 7.68E-01 | 9.63E-01 |
|  | rs4084096 | HLA-G | 6 | 1.40E-02 | NA | NA | 1.40E-02 | NA | NA | NA | NA |
|  | rs3893464 | HLA-G | 6 | 4.35E-03 | NA | NA | 4.90E-03 | 2.88E-02 | 4.35E-03 | NA | NA |
|  | rs7382061 | RNF39 | 6 | 1.95E-02 | 2.77E-02 | 5.60E-02 | 2.19E-02 | NA | NA | 1.95E-02 | 9.99E-01 |
|  | rs6909253 | RNF39 | 6 | 2.70E-02 | 3.90E-02 | 6.84E-02 | 2.94E-02 | 3.18E-01 | 3.13E-02 | 2.70E-02 | 9.98E-01 |
|  | rs9378220 | TRIM31 | 6 | 1.79E-03 | NA | NA | 4.65E-02 | 5.87E-01 | 1.79E-03 | NA | NA |
|  | rs1264616 | HCG18 | 6 | 2.30E-02 | 8.87E-01 | 9.00E-01 | 2.54E-02 | 2.31E-02 | 2.70E-02 | 2.30E-02 | 1.00E+00 |
|  | rs6912495 | HCG18 | 6 | 2.19E-02 | NA | NA | 2.19E-02 | NA | NA | NA | NA |
|  | rs2240058 | TRIM39 | 6 | 1.39E-02 | 8.87E-01 | 9.86E-01 | 6.71E-02 | 1.39E-02 | 1.72E-02 | 1.98E-01 | 9.62E-01 |
| 168* | rs2523726 | TRIM39 | 6 | 4.91E-02 | 3.04E-01 | 5.93E-01 | 5.28E-02 | 4.91E-02 | NA | 8.94E-01 | 9.94E-01 |
|  | rs2524222 | GNL1 | 6 | 1.15E-02 | 9.71E-01 | 9.86E-01 | 1.40E-02 | NA | NA | 1.15E-02 | 1.00E+00 |
|  | rs2844713 | GNL1 | 6 | 2.70E-02 | 8.88E-01 | 1.00E+00 | 6.93E-02 | 2.70E-02 | 3.13E-02 | 2.70E-02 | 9.60E-01 |
|  | rs2516647 | PRR3 | 6 | 2.30E-02 | 6.93E-01 | 1.00E+00 | 8.11E-02 | NA | NA | 2.30E-02 | 9.57E-01 |
|  | rs3132612 | ABCF1 | 6 | 3.95E-02 | NA | NA | 3.95E-02 | NA | NA | NA | NA |
|  | rs3095326 | IER3 | 6 | 3.16E-02 | 2.25E-01 | 9.00E-01 | 6.32E-01 | NA | NA | 3.16E-02 | 9.62E-01 |
|  | rs4713366 | C6orf214 | 6 | 2.70E-02 | NA | NA | 2.94E-02 | 2.70E-02 | 3.13E-02 | NA | NA |
|  | rs4713367 | C6orf214 | 6 | 2.30E-02 | 4.94E-01 | 9.00E-01 | 2.54E-02 | NA | NA | 2.30E-02 | 8.46E-01 |
|  | rs11758688 | C6orf214 | 6 | 2.30E-02 | 4.94E-01 | 9.00E-01 | 2.54E-02 | 2.31E-02 | NA | 2.30E-02 | 8.46E-01 |
|  | rs1264344 | C6orf214 | 6 | 3.72E-03 | 2.51E-01 | 9.86E-01 | 5.66E-03 | 9.68E-01 | 5.19E-03 | 3.72E-03 | 1.00E+00 |
|  | rs885905 | VARS2 | 6 | 3.16E-02 | 9.12E-01 | 5.98E-01 | 3.41E-02 | NA | NA | 3.16E-02 | 8.87E-01 |
|  | rs2532934 | VARS2 | 6 | 1.16E-02 | 8.87E-01 | 8.20E-01 | 1.40E-02 | 1.16E-02 | 1.47E-02 | 3.24E-02 | 8.77E-01 |
|  | rs13210132 | HCG22 | 6 | 1.75E-04 | 6.01E-02 | 1.95E-01 | 9.76E-02 | 2.17E-04 | 3.20E-04 | 1.75E-04 | 9.62E-01 |
|  | rs3094205 | PSORS1C1 | 6 | 3.69E-02 | 5.05E-01 | 9.00E-02 | 6.97E-01 | 5.66E-01 | 4.20E-02 | 3.69E-02 | 9.46E-01 |
|  | rs3778639 | PSORS1C1 | 6 | 1.73E-03 | 3.58E-03 | 2.32E-01 | 3.18E-03 | NA | NA | 1.73E-03 | 5.74E-01 |
|  | rs3130573 | PSORS1C1 | 6 | 2.30E-02 | 9.12E-01 | 9.29E-01 | 2.54E-02 | 2.31E-02 | 2.70E-02 | 2.30E-02 | 9.61E-01 |
|  | rs879882 | POU5F1 | 6 | 2.70E-02 | 1.71E-01 | 3.93E-01 | 2.94E-02 | 2.70E-02 | 3.13E-02 | 2.70E-02 | 9.60E-01 |
|  | rs2853950 | HLA-C | 6 | 1.03E-02 | NA | NA | 1.03E-02 | NA | NA | NA | NA |
|  | rs9264601 | HLA-C | 6 | 2.78E-02 | NA | NA | 2.78E-02 | NA | NA | NA | NA |
|  | rs9264603 | HLA-C | 6 | 1.92E-02 | NA | NA | 1.92E-02 | NA | NA | NA | NA |
|  | rs2249742 | HLA-B | 6 | 2.15E-04 | 2.22E-04 | 5.48E-01 | 1.92E-02 | 2.53E-04 | 3.89E-04 | 2.15E-04 | 2.80E-01 |
|  | rs13191343 | HLA-B | 6 | 9.98E-05 | NA | NA | 9.98E-05 | NA | NA | NA | NA |
|  | rs9295986 | HLA-B | 6 | 1.32E-03 | NA | NA | 2.79E-03 | 1.32E-03 | 2.14E-03 | NA | NA |
|  | rs2844533 | MICA | 6 | 4.39E-03 | 6.93E-01 | 7.06E-01 | 6.54E-03 | 4.39E-03 | NA | 9.87E-01 | 8.46E-01 |
|  | rs13437088 | MICA | 6 | 5.24E-06 | 5.82E-01 | 8.65E-01 | 1.24E-04 | NA | NA | 5.24E-06 | 9.56E-01 |
|  | rs2523454 | MICA | 6 | 6.50E-03 | 9.12E-01 | 9.86E-01 | 8.82E-03 | 6.50E-03 | 8.86E-03 | 6.54E-03 | 9.61E-01 |
|  | rs2256175 | MICA | 6 | 3.61E-03 | 2.51E-01 | 3.08E-01 | 5.66E-03 | 3.61E-03 | 5.19E-03 | 5.76E-03 | 9.99E-01 |
|  | rs6934187 | MICA | 6 | 1.41E-05 | NA | NA | 1.41E-05 | NA | NA | NA | NA |
|  | rs9501106 | MICA | 6 | 1.79E-05 | NA | NA | 3.03E-05 | 2.18E-05 | 1.79E-05 | NA | NA |
|  | rs2516440 | HCG26 | 6 | 1.39E-02 | NA | NA | 1.62E-02 | 1.39E-02 | 1.72E-02 | NA | NA |
|  | rs4413654 | HCG26 | 6 | 1.32E-03 | 3.73E-01 | 1.00E+00 | 6.10E-01 | 1.32E-03 | 2.14E-03 | 4.71E-02 | 9.57E-01 |
|  | rs12660382 | HCG26 | 6 | 2.96E-03 | 4.56E-01 | 9.29E-01 | 4.90E-03 | 2.96E-03 | 4.35E-03 | 3.08E-03 | 9.56E-01 |
|  | rs16899682 | HCG26 | 6 | 3.95E-02 | 8.91E-01 | 9.28E-01 | 3.95E-02 | NA | NA | 9.55E-01 | 9.68E-01 |
|  | rs4959077 | MICB | 6 | 1.40E-02 | NA | NA | 1.40E-02 | NA | NA | NA | NA |
|  | rs3093995 | MCCD1 | 6 | 4.31E-02 | 6.43E-02 | 3.53E-01 | 9.61E-01 | NA | NA | 4.31E-02 | 5.75E-01 |
|  | rs2071593 | BAT1 | 6 | 1.62E-03 | 6.35E-01 | 2.32E-01 | 3.18E-03 | 1.62E-03 | 2.56E-03 | 6.30E-01 | 6.31E-01 |
|  | rs2230365 | NFKBIL1 | 6 | 1.27E-03 | 1.27E-03 | 6.75E-03 | 2.34E-03 | 6.94E-01 | 1.79E-03 | 9.43E-01 | 9.61E-01 |
|  | rs1799964 | LTA | 6 | 1.65E-02 | 2.32E-02 | 1.00E+00 | 2.70E-01 | 1.65E-02 | NA | 1.44E-01 | 7.23E-01 |
|  | rs1052248 | LST1 | 6 | 1.79E-04 | 1.79E-04 | 8.65E-01 | 8.11E-02 | 8.43E-01 | NA | 1.13E-02 | 6.31E-01 |
|  | rs2857595 | NCR3 | 6 | 3.61E-03 | 6.93E-01 | 3.32E-01 | 1.03E-02 | 3.61E-03 | NA | 2.66E-01 | 9.56E-01 |
|  | rs2857700 | AIF1 | 6 | 1.20E-02 | 3.19E-02 | 1.00E+00 | 1.20E-02 | NA | NA | 4.71E-02 | 2.53E-01 |
|  | rs2857697 | AIF1 | 6 | 2.74E-03 | 1.73E-02 | 5.08E-01 | 2.74E-03 | 1.75E-01 | NA | 1.33E-02 | 9.62E-01 |
|  | rs2736172 | C6orf21 | 6 | 2.70E-02 | 4.17E-01 | 3.61E-01 | 2.94E-02 | 2.70E-02 | NA | 2.70E-02 | 9.60E-01 |
|  | rs2736171 | C6orf21 | 6 | 3.68E-03 | 1.73E-02 | 5.08E-01 | 3.68E-03 | NA | NA | 1.13E-02 | 9.62E-01 |
|  | rs2261033 | BAT2 | 6 | 3.08E-03 | 9.71E-01 | 8.87E-03 | 4.90E-03 | NA | NA | 3.08E-03 | 3.83E-01 |
|  | rs3132453 | BAT2 | 6 | 3.15E-02 | NA | NA | 3.41E-02 | 3.15E-02 | 3.62E-02 | NA | NA |
|  | rs3130617 | C6orf47 | 6 | 1.62E-03 | 7.56E-01 | 5.93E-01 | 3.18E-03 | 1.62E-03 | 2.56E-03 | 4.71E-02 | 6.31E-01 |
|  | rs805292 | LY6G6C | 6 | 4.91E-02 | 5.21E-01 | 4.68E-01 | 1.18E-01 | 4.91E-02 | NA | 9.66E-01 | 8.97E-01 |
|  | rs3117577 | MSH5 | 6 | 7.06E-03 | 7.06E-03 | 7.66E-01 | 8.28E-03 | NA | NA | 1.58E-02 | 5.74E-01 |
|  | rs660594 | SLC44A4 | 6 | 1.40E-02 | 1.06E-01 | 9.86E-01 | 1.40E-02 | NA | 1.47E-02 | 9.87E-01 | 6.75E-01 |
|  | rs644827 | SLC44A4 | 6 | 1.96E-02 | 1.46E-01 | 1.00E+00 | 2.19E-02 | 1.96E-02 | 2.33E-02 | 9.87E-01 | 6.75E-01 |
|  | rs3869145 | NG36/G9a | 6 | 1.88E-02 | 5.36E-01 | 5.48E-01 | 1.88E-02 | NA | NA | 2.30E-01 | 9.61E-01 |
|  | rs644045 | C2 | 6 | 3.39E-06 | 4.56E-01 | 4.29E-01 | 1.64E-05 | 4.15E-02 | NA | 3.39E-06 | 2.09E-02 |
|  | rs7746553 | C2 | 6 | 3.95E-02 | NA | NA | 3.95E-02 | NA | NA | NA | NA |
|  | rs592229 | SKIV2L | 6 | 2.26E-05 | 9.12E-01 | 7.76E-05 | 6.99E-05 | 4.89E-01 | 4.46E-05 | 2.26E-05 | 3.10E-01 |
|  | rs7774197 | TNXB | 6 | 3.05E-03 | 8.18E-01 | 9.86E-01 | 6.10E-01 | 3.95E-01 | 3.05E-03 | 1.01E-01 | 9.62E-01 |
|  | rs12663103 | GPSM3 | 6 | 3.16E-02 | 4.61E-02 | 7.84E-02 | 3.41E-02 | NA | 3.62E-02 | 3.16E-02 | 9.60E-01 |
|  | rs8192583 | GPSM3 | 6 | 2.30E-02 | 3.29E-02 | 6.64E-02 | 2.54E-02 | NA | NA | 2.30E-02 | 9.62E-01 |
| 169* | rs411326 | AK123889 | 6 | 2.31E-02 | 9.71E-01 | 5.97E-02 | 2.54E-02 | 2.31E-02 | 2.70E-02 | 1.00E+00 | 5.96E-01 |
|  | rs2022544 | C6orf10 | 6 | 1.15E-02 | 8.97E-01 | 9.29E-01 | 6.60E-01 | NA | NA | 1.15E-02 | 9.56E-01 |
|  | rs6930777 | BTNL2 | 6 | 5.34E-08 | NA | 5.34E-08 | 8.87E-08 | 1.37E-07 | NA | NA | NA |
|  | rs9268480 | BTNL2 | 6 | 1.62E-02 | 2.20E-01 | 3.90E-02 | 1.62E-02 | NA | NA | 7.56E-01 | 2.53E-01 |
|  | rs3793126 | BTNL2 | 6 | 1.40E-02 | NA | NA | 1.40E-02 | NA | NA | NA | NA |
|  | rs3763313 | BTNL2 | 6 | 9.71E-08 | 1.29E-05 | 1.11E-03 | 9.17E-01 | 1.37E-07 | 9.71E-08 | 1.88E-02 | 5.32E-01 |
|  | rs9268528 | BTNL2 | 6 | 2.94E-02 | 2.22E-01 | 1.25E-01 | 2.94E-02 | NA | 3.13E-02 | 9.53E-01 | 9.98E-01 |
|  | rs2395174 | HLA-DRA | 6 | 3.72E-03 | 6.35E-01 | 2.14E-01 | 5.66E-03 | NA | 5.19E-03 | 3.72E-03 | 9.57E-01 |
|  | rs9268626 | HLA-DRA | 6 | 2.34E-03 | NA | NA | 2.34E-03 | NA | NA | NA | NA |
|  | rs3129872 | HLA-DRA | 6 | 2.10E-03 | 6.35E-01 | 6.46E-01 | 3.68E-03 | NA | NA | 2.10E-03 | 9.57E-01 |
|  | rs2040406 | HLA-DQA1 | 6 | 3.68E-03 | NA | NA | 3.68E-03 | NA | NA | NA | NA |
|  | rs9272346 | HLA-DQA1 | 6 | 4.96E-04 | 7.56E-01 | 1.39E-03 | 1.16E-03 | 4.96E-04 | NA | 5.61E-01 | 9.99E-01 |
|  | rs3891175 | HLA-DQB1 | 6 | 2.74E-03 | 4.16E-01 | 3.88E-03 | 2.74E-03 | NA | NA | 9.50E-01 | 9.56E-01 |
|  | rs9275522 | HLA-DQA2 | 6 | 4.90E-03 | 2.51E-01 | 4.69E-01 | 4.90E-03 | NA | NA | 4.91E-01 | 2.50E-02 |
|  | rs17427887 | HLA-DQA2 | 6 | 4.90E-03 | NA | NA | 4.90E-03 | NA | NA | NA | NA |
|  | rs2858331 | HLA-DQA2 | 6 | 4.99E-05 | 4.99E-05 | 1.61E-04 | 1.45E-04 | 8.39E-05 | 9.65E-05 | 5.12E-05 | 1.70E-01 |
|  | rs3104405 | HLA-DQA2 | 6 | 1.64E-02 | 8.87E-01 | 4.52E-02 | 1.88E-02 | 1.65E-02 | 2.00E-02 | 1.64E-02 | 1.00E+00 |
|  | rs4548006 | HLA-DQA2 | 6 | 3.95E-02 | NA | NA | 3.95E-02 | NA | NA | NA | NA |
|  | rs10947340 | HLA-DQA2 | 6 | 5.51E-04 | NA | NA | 5.51E-04 | NA | NA | NA | NA |
|  | rs10807113 | HLA-DQB2 | 6 | 2.97E-04 | 1.73E-02 | 1.35E-03 | 6.65E-04 | 2.97E-04 | 4.74E-04 | 1.98E-01 | 7.70E-01 |
|  | rs1015575 | HLA-DQB2 | 6 | 7.58E-03 | 1.25E-01 | 3.92E-01 | 7.58E-03 | NA | NA | 1.58E-02 | 8.46E-01 |
|  | rs1383264 | HLA-DQB2 | 6 | 1.17E-04 | 1.17E-04 | 3.44E-04 | 3.31E-02 | NA | NA | 4.71E-02 | 8.11E-01 |
|  | rs2621321 | TAP2 | 6 | 4.98E-05 | 5.81E-04 | 1.47E-02 | 4.98E-05 | NA | NA | 1.63E-03 | 5.57E-01 |
|  | rs241454 | TAP2 | 6 | 5.89E-05 | 1.65E-04 | 2.17E-02 | 5.89E-05 | NA | NA | 9.81E-05 | 5.57E-01 |
|  | rs241447 | TAP2 | 6 | 3.70E-05 | 1.65E-04 | 2.64E-02 | 5.89E-05 | 3.90E-05 | 3.70E-05 | 1.21E-04 | 5.57E-01 |
| 170* | rs2281389 | HLA-DPB1 | 6 | 4.39E-03 | 8.63E-01 | 2.50E-01 | 6.54E-03 | 4.39E-03 | 8.96E-01 | 2.30E-01 | 5.57E-01 |
|  | rs2064473 | HLA-DPB1 | 6 | 3.68E-03 | NA | NA | 3.68E-03 | NA | NA | NA | NA |
|  | rs3117230 | HLA-DPB1 | 6 | 3.15E-02 | 8.64E-01 | 1.28E-01 | 3.41E-02 | 3.15E-02 | 3.62E-02 | 7.04E-01 | 7.59E-02 |
| 171* | rs9277771 | HLA-DPB1 | 6 | 2.54E-02 | NA | NA | 2.54E-02 | NA | NA | NA | NA |
| 172* | rs1799908 | COL11A2 | 6 | 1.03E-02 | NA | NA | 1.03E-02 | NA | NA | NA | NA |
| 176 | rs9463802 | CR605995 | 6 | 1.05E-02 | 8.63E-01 | 4.69E-01 | 5.16E-01 | 8.77E-02 | 1.05E-02 | 5.61E-01 | 9.62E-01 |
| 190 | rs212803 | EYA4 | 6 | 4.23E-02 | 4.23E-02 | 9.82E-01 | 9.59E-01 | NA | NA | 5.65E-01 | 9.64E-01 |
| 198 | rs11965350 | PNLDC1 | 6 | 4.31E-02 | 8.19E-01 | 9.09E-01 | 9.61E-01 | 9.05E-01 | 1.00E+00 | 4.31E-02 | 1.00E+00 |
| 220 | rs8179 | CDK6 | 7 | 1.62E-02 | NA | 8.65E-01 | 1.62E-02 | 8.43E-01 | 8.96E-01 | 9.58E-01 | 9.56E-01 |
| 221 | rs6964803 | CDK6 | 7 | 4.45E-02 | NA | 8.33E-01 | 6.60E-01 | 4.45E-02 | 9.05E-01 | NA | NA |
| 222 | rs43041 | PON2 | 7 | 2.70E-02 | 9.13E-01 | 8.64E-01 | 6.21E-01 | 2.70E-02 | 8.97E-01 | 7.59E-01 | 9.60E-01 |
| 227 | rs205774 | FLJ43663 | 7 | 4.71E-02 | 8.18E-01 | 5.08E-01 | 9.17E-01 | 8.43E-01 | 8.96E-01 | 4.71E-02 | 9.57E-01 |
| 234 | rs1961456 | NAT2 | 8 | 7.58E-03 | 8.63E-01 | 9.00E-01 | 7.58E-03 | NA | NA | 8.49E-01 | 8.11E-01 |
| 263 | rs1961992 | DENND4C | 9 | 4.15E-02 | 9.12E-01 | 9.29E-01 | 7.75E-01 | 4.15E-02 | 8.96E-01 | 9.58E-01 | 9.56E-01 |
| 265 | rs7019909 | B4GALT1 | 9 | 4.91E-02 | 8.67E-01 | 1.00E+00 | 8.65E-01 | 4.91E-02 | 8.47E-01 | 9.76E-01 | 9.79E-01 |
| 268 | rs10781385 | PRUNE2 | 9 | 4.58E-02 | NA | 9.31E-01 | 4.58E-02 | NA | NA | NA | NA |
| 270 | rs10512111 | TLE1 | 9 | 4.25E-02 | NA | NA | 8.97E-01 | 4.25E-02 | 8.63E-01 | NA | NA |
| 283 | rs16916931 | ARID5B | 10 | 2.19E-02 | NA | NA | 2.19E-02 | NA | NA | NA | NA |
| 283 | rs7922857 | ARID5B | 10 | 4.25E-02 | 9.04E-01 | 7.84E-01 | 4.58E-02 | 4.25E-02 | NA | 7.14E-01 | 9.70E-01 |
| 286 | rs10881600 | TSPAN14 | 10 | 3.31E-02 | 3.73E-01 | 8.65E-01 | 3.31E-02 | NA | NA | 9.50E-01 | 6.31E-01 |
| 288 | rs11187157 | HHEX | 10 | 5.86E-03 | NA | 5.86E-03 | 9.60E-01 | NA | NA | 9.60E-01 | 9.57E-01 |
| 293 | rs2792751 | GPAM | 10 | 4.51E-02 | 7.56E-01 | 9.86E-01 | 1.00E+00 | 6.94E-01 | 4.51E-02 | 9.87E-01 | 1.00E+00 |
| 299 | rs12283172 | AMBRA1 | 11 | 2.70E-02 | 6.37E-01 | 9.28E-01 | 7.83E-01 | 2.70E-02 | 9.76E-01 | 5.65E-01 | 9.64E-01 |
| 300 | rs102275 | C11orf10 | 11 | 1.13E-04 | 1.13E-04 | 7.66E-01 | 3.41E-01 | 4.89E-01 | 2.06E-01 | 9.58E-01 | 9.02E-01 |
|  | rs968567 | FADS2 | 11 | 9.17E-04 | 1.93E-01 | 9.29E-01 | 9.17E-04 | 2.70E-01 | 2.06E-01 | 9.47E-01 | 9.02E-01 |
|  | rs174634 | FADS3 | 11 | 2.78E-02 | NA | NA | 2.78E-02 | NA | NA | NA | NA |
| 303 | rs567956 | FLJ00225 | 11 | 1.24E-02 | 1.24E-02 | 9.29E-01 | 1.00E+00 | NA | NA | 1.00E+00 | 9.99E-01 |
| 307 | rs7120515 | AB231710 | 11 | 2.88E-02 | 9.71E-01 | 7.06E-01 | 1.84E-01 | 2.88E-02 | NA | 9.58E-01 | 9.57E-01 |
| 308 | rs7116375 | AMICA1 | 11 | 1.96E-02 | 4.56E-01 | 8.65E-01 | 9.60E-01 | 1.96E-02 | NA | 1.00E+00 | 9.99E-01 |
| 310 | rs573905 | TREH | 11 | 1.88E-02 | 8.97E-01 | 8.65E-01 | 1.88E-02 | 9.68E-01 | 8.57E-01 | 9.58E-01 | 9.61E-01 |
| 312 | rs12422102 | UBASH3B | 11 | 4.04E-02 | 6.94E-01 | 9.82E-01 | 9.59E-01 | 8.93E-01 | 5.16E-01 | 4.04E-02 | 9.64E-01 |
| 313 | rs512577 | FAM118B | 11 | 4.51E-02 | 8.87E-01 | 8.65E-01 | 7.13E-01 | 1.00E+00 | 4.51E-02 | 8.49E-01 | 9.57E-01 |
| 317 | rs2276437 | SNX19 | 11 | 4.96E-02 | 1.00E+00 | 9.79E-01 | 4.96E-02 | 8.57E-01 | 3.29E-01 | 1.00E+00 | 9.60E-01 |
| 325 | rs2253736 | SLC2A13 | 12 | 3.29E-02 | 3.29E-02 | 2.14E-01 | 9.17E-01 | NA | NA | 9.43E-01 | 1.00E+00 |
| 331 | rs7970490 | CUX2 | 12 | 3.66E-02 | NA | 9.77E-01 | 6.97E-01 | 3.66E-02 | 4.20E-02 | 9.62E-01 | 9.95E-01 |
|  | rs3847953 | CUX2 | 12 | 2.70E-02 | NA | 9.00E-01 | 6.10E-01 | NA | 2.70E-02 | 9.43E-01 | 9.99E-01 |
|  | rs3184504 | SH2B3 | 12 | 3.50E-07 | NA | NA | 9.76E-02 | 4.87E-07 | 3.50E-07 | NA | NA |
|  | rs630512 | ATXN2 | 12 | 3.37E-05 | NA | 8.65E-01 | 6.10E-01 | 3.37E-05 | 2.06E-01 | 6.30E-01 | 9.62E-01 |
|  | rs11066320 | PTPN11 | 12 | 2.66E-06 | NA | 2.50E-01 | 1.16E-01 | 2.66E-06 | 9.30E-06 | 7.56E-01 | 9.57E-01 |
|  | rs11066344 | RPH3A | 12 | 4.25E-02 | NA | 8.75E-01 | 9.61E-01 | 4.25E-02 | NA | 9.66E-01 | 9.94E-01 |
| 332 | rs7315519 | RPH3A | 12 | 3.66E-02 | NA | 8.25E-01 | 9.59E-01 | 3.66E-02 | 7.44E-01 | 9.20E-01 | 9.68E-01 |
| 344 | rs2028809 | C13orf31 | 13 | 4.63E-02 | 8.18E-01 | 9.28E-01 | 9.17E-01 | 5.97E-01 | 4.63E-02 | 9.49E-01 | 9.25E-01 |
| 345 | rs1198329 | KPNA3 | 13 | 1.92E-02 | 8.63E-01 | 3.92E-01 | 1.92E-02 | 7.50E-01 | 8.96E-01 | 9.47E-01 | 9.56E-01 |
| 350 | rs2590544 | GPC6 | 13 | 4.58E-02 | 9.65E-01 | 9.09E-01 | 4.58E-02 | 7.85E-01 | NA | 9.86E-01 | 9.72E-01 |
| 356 | rs2125598 | C14orf177 | 14 | 4.99E-02 | 1.00E+00 | 3.92E-01 | 7.75E-01 | 4.99E-02 | NA | 9.87E-01 | 9.57E-01 |
| 358 | rs3742384 | WARS | 14 | 1.11E-02 | NA | NA | 9.17E-01 | 1.11E-02 | 7.68E-01 | NA | NA |
| 365 | rs2472304 | CYP1A2 | 15 | 8.39E-03 | 9.12E-01 | 9.00E-01 | 9.06E-01 | 1.00E+00 | 8.39E-03 | NA | 9.57E-01 |
|  | rs4886649 | PPCDC | 15 | 4.51E-02 | 9.71E-01 | 9.86E-01 | 9.60E-01 | 9.68E-01 | 4.51E-02 | 1.00E+00 | 9.57E-01 |
| 367 | rs248848 | CLEC16A | 16 | 3.45E-02 | 9.71E-01 | 9.86E-01 | 8.38E-01 | 3.45E-02 | NA | 9.50E-01 | 9.99E-01 |
| 371 | rs7342689 | RBBP6 | 16 | 3.16E-02 | 8.99E-01 | 8.64E-01 | 9.08E-01 | 6.58E-01 | 9.74E-01 | 3.16E-02 | 9.61E-01 |
| 377 | rs3764310 | DHODH | 16 | 3.19E-02 | 3.19E-02 | 8.20E-01 | 9.60E-01 | 8.43E-01 | 9.77E-01 | 9.60E-01 | 9.99E-01 |
|  | rs2000999 | TXNL4B | 16 | 2.88E-02 | 9.12E-01 | 8.65E-01 | 9.17E-01 | 2.88E-02 | 9.77E-01 | 9.87E-01 | 9.62E-01 |
|  | rs11648622 | PMFBP1 | 16 | 1.99E-02 | 6.35E-01 | 9.29E-01 | 7.75E-01 | 1.99E-02 | NA | 9.60E-01 | 8.77E-01 |
| 384 | rs781848 | ZZEF1 | 17 | 2.70E-02 | 9.69E-01 | 6.47E-01 | 8.38E-02 | 2.70E-02 | NA | 7.59E-01 | 9.60E-01 |
| 386 | rs3744647 | ARHGEF15 | 17 | 4.51E-02 | 9.71E-01 | 1.00E+00 | 5.16E-01 | 8.89E-01 | 4.51E-02 | 8.06E-01 | 8.46E-01 |
| 387 | rs4791858 | USP43 | 17 | 4.89E-02 | 4.89E-02 | 1.00E+00 | 9.60E-01 | 6.94E-01 | NA | 9.87E-01 | 8.77E-01 |
| 394 | rs11651246 | FAM134C | 17 | 3.88E-02 | 8.86E-02 | 3.88E-02 | 9.60E-01 | NA | NA | 9.43E-01 | 9.25E-01 |
| 397 | rs8080583 | KIAA1267 | 17 | 4.91E-02 | 9.08E-02 | 9.36E-01 | 9.64E-01 | 4.91E-02 | NA | 5.83E-01 | 9.70E-01 |
| 398 | rs6504833 | ITGB3 | 17 | 2.39E-02 | 9.12E-01 | 1.00E+00 | 8.38E-01 | 2.39E-02 | 8.05E-01 | 9.60E-01 | 9.56E-01 |
|  | rs3760371 | NPEPPS | 17 | 4.51E-02 | 8.87E-01 | 6.46E-01 | 9.06E-01 | 6.02E-02 | 4.51E-02 | 9.43E-01 | 9.62E-01 |
| 399 | rs4141183 | OSBPL7 | 17 | 4.62E-02 | 6.98E-01 | 9.03E-01 | 9.23E-01 | 4.62E-02 | 7.44E-01 | 9.86E-01 | 8.66E-01 |
| 412 | rs7232532 | DKFZp762F112 | 18 | 3.90E-02 | 3.90E-02 | 3.61E-01 | 9.17E-01 | 9.68E-01 | NA | 9.87E-01 | 7.82E-01 |
| 416 | rs1378528 | RAB31 | 18 | 3.62E-02 | 8.89E-01 | 9.27E-01 | 9.58E-01 | 9.68E-01 | 3.62E-02 | 1.00E+00 | 9.96E-01 |
| 423 | rs8112157 | CLEC4GP1 | 19 | 3.92E-02 | 9.71E-01 | 9.29E-01 | 3.92E-02 | 9.68E-01 | 5.07E-01 | NA | 5.96E-01 |
| 424 | rs7253253 | SLC44A2 | 19 | 4.32E-03 | 4.32E-03 | 1.77E-01 | 6.60E-01 | NA | NA | 2.66E-01 | 9.62E-01 |
|  | rs10418550 | SLC44A2 | 19 | 1.73E-02 | 1.73E-02 | 9.00E-01 | 7.13E-01 | 8.89E-01 | NA | 1.00E+00 | 9.56E-01 |
|  | rs6511708 | ILF3 | 19 | 3.31E-02 | 9.12E-01 | 9.29E-01 | 3.31E-02 | NA | NA | 1.98E-01 | 9.56E-01 |
|  | rs12974306 | DNM2 | 19 | 4.86E-03 | 7.56E-01 | 1.00E+00 | 5.63E-01 | 8.00E-01 | 1.00E+00 | 4.86E-03 | 7.70E-01 |
|  | rs11085749 | C19orf38 | 19 | 1.34E-03 | 8.87E-01 | 1.00E+00 | 9.17E-01 | 6.40E-01 | 7.68E-01 | 1.34E-03 | 9.02E-01 |
|  | rs12983316 | SMARCA4 | 19 | 9.07E-04 | NA | 9.86E-01 | 9.17E-01 | NA | NA | 9.07E-04 | 5.96E-01 |
| 427 | rs7188 | KANK2 | 19 | 4.31E-03 | 6.93E-01 | 9.29E-01 | 4.31E-03 | 8.89E-01 | 7.68E-01 | 9.47E-01 | 8.77E-01 |
| 429 | rs7252293 | DOCK6 | 19 | 3.92E-02 | 9.71E-01 | 8.65E-01 | 3.92E-02 | 8.89E-01 | 8.05E-01 | 9.43E-01 | 1.00E+00 |
| 432 | rs2965185 | GATAD2A | 19 | 3.47E-03 | 8.97E-01 | 9.86E-01 | 3.47E-03 | NA | NA | 9.60E-01 | 9.61E-01 |
| 445 | rs4803760 | BCAM | 19 | 1.58E-02 | 9.12E-01 | 3.32E-01 | 7.75E-01 | 8.89E-01 | 4.52E-01 | 1.58E-02 | 9.57E-01 |
|  | rs4803766 | PVRL2 | 19 | 3.24E-02 | 9.12E-01 | 9.86E-01 | 9.60E-01 | 9.68E-01 | 6.73E-01 | 3.24E-02 | 9.62E-01 |
| 452 | rs4802307 | PPP5C | 19 | 3.29E-02 | 3.29E-02 | 9.86E-01 | 4.24E-01 | 6.40E-01 | 3.11E-01 | 9.60E-01 | 9.99E-01 |
| 453 | rs432157 | PRKD2 | 19 | 1.65E-02 | NA | NA | 8.85E-01 | 1.65E-02 | 2.37E-01 | NA | NA |
| 454 | rs516246 | FUT2 | 19 | 9.37E-06 | 9.37E-06 | 1.00E+00 | 1.00E+00 | NA | NA | 8.36E-02 | 5.74E-01 |
|  | rs485186 | FUT2 | 19 | 6.18E-05 | 6.18E-05 | 1.00E+00 | 9.06E-01 | 6.38E-04 | 1.55E-01 | 1.70E-01 | 5.74E-01 |
|  | rs838143 | FUT1 | 19 | 3.13E-02 | 7.57E-01 | 6.47E-01 | 1.00E+00 | 8.93E-01 | 3.13E-02 | 2.02E-01 | 9.60E-01 |
|  | rs838133 | FGF21 | 19 | 1.88E-02 | 6.35E-01 | 9.29E-01 | 9.06E-01 | 2.88E-02 | 5.07E-01 | 1.88E-02 | 9.02E-01 |
| 461 | rs6060491 | FER1L4 | 20 | 2.00E-02 | NA | NA | 9.60E-01 | 9.68E-01 | 2.00E-02 | NA | NA |
|  | rs11697672 | C20orf152 | 20 | 2.84E-02 | 8.97E-01 | 2.84E-02 | 9.17E-01 | 8.00E-01 | NA | 1.00E+00 | 9.56E-01 |
| 474 | rs1885088 | HNF4A | 20 | 4.52E-02 | 8.63E-01 | 4.52E-02 | 1.00E+00 | 5.87E-01 | 1.00E+00 | 9.50E-01 | 9.46E-01 |
| 478 | rs6010652 | ZBTB46 | 20 | 2.84E-02 | 1.68E-01 | 2.84E-02 | 9.76E-02 | NA | NA | 8.06E-01 | 9.57E-01 |
|  | rs6011118 | ZBTB46 | 20 | 3.34E-02 | 1.93E-01 | 3.34E-02 | 4.65E-02 | NA | NA | 8.06E-01 | 9.99E-01 |
| 479 | rs7280420 | RUNX1 | 21 | 1.57E-02 | NA | NA | 1.57E-02 | NA | NA | NA | NA |
| 480 | rs2837690 | DSCAM | 21 | 2.77E-02 | 2.77E-02 | 9.00E-01 | 6.60E-01 | NA | NA | 9.87E-01 | 6.75E-01 |
| 483 | rs4820821 | ASCC2 | 22 | 6.50E-03 | 1.46E-01 | 9.00E-01 | 8.38E-01 | 6.50E-03 | NA | 6.30E-01 | 9.61E-01 |

Independent complex or single gene loci (r^2^ < 0.2) with SNP(s) with a conjunctional FDR (conjFDR) < 0.05 in *both* low density lipoprotein (LDL) *and* in the associated immune mediated disease. All SNPs with a conjFDR value < 0.05 (bidirectional association, i.e. association with the lipid phenotype given the immune mediated disease (condFDR< 0.05) and association with immune-mediated disease given the lipid phenotype (condFDR<0.05)) are listed and sorted in each LD block. For LDL and immune-mediated disease phenotype we defined the most significant SNP in each LD block based on the minimum conjFDR (min conjFDR). For comparison, the conjFDR values for each identified SNP are listed for all phenotypes, and the minimum (min) conjFDR across all phenotypes. All independent loci are listed consecutively, and Major Histocomaptibility Complex loci are marked (*).All data were first corrected for genomic inflation. Crohn’s Disease (CD), ulcerative colitis (UC), rheumatoid arthritis (RA), type 1 diabetes (T1D), celiac disease (CeD), psoriasis (PSOR) and sarcoidosis (SARC). Chromosome (Chr). NA; not available.

### Table J. Pleiotropic SNPs in high density lipoprotein (HDL) and immune mediated diseases (conjFDR<0.05).

| **#Locus** | **SNP** | **gene** | **chr** | **Min conjFDR** | **HDL & CD** | **HDL & UC** | **HDL & RA** | **HDL & T1D** | **HDL & CeD** | **HDL & PSOR** | **HDL & SARC** |
| --- | --- | --- | --- | --- | --- | --- | --- | --- | --- | --- | --- |
| 1 | rs707583 | *AK125078* | 1 | 4.48E-02 | 9.02E-01 | 8.50E-01 | 9.89E-01 | 4.48E-02 | NA | 9.11E-01 | 9.62E-01 |
| 9 | rs2293476 | *PABPC4* | 1 | 4.48E-02 | 2.34E-01 | 7.87E-01 | 1.00E+00 | 4.48E-02 | NA | 8.87E-01 | 1.00E+00 |
| 12 | rs6587980 | *DOCK7* | 1 | 3.50E-02 | 3.50E-02 | 8.94E-01 | 9.65E-01 | NA | NA | 9.73E-01 | 1.00E+00 |
| 13 | rs1474927 | *M96* | 1 | 2.01E-02 | 2.01E-02 | 2.22E-01 | 2.17E-01 | NA | NA | 9.11E-01 | 1.00E+00 |
| 31 | rs3813982 | *SRGAP2* | 1 | 8.04E-03 | 1.00E+00 | 8.04E-03 | 7.62E-02 | 7.59E-01 | 8.56E-01 | 9.11E-01 | 9.57E-01 |
|  | rs3849277 | *SRGAP2* | 1 | 4.77E-02 | NA | NA | 4.77E-02 | NA | NA | NA | NA |
| 45 | rs17799872 | *ADCY3* | 2 | 9.09E-03 | 6.74E-01 | 7.87E-01 | 9.28E-01 | 9.09E-03 | 8.66E-01 | 9.09E-01 | 8.01E-01 |
| 46 | rs12713007 | *FOXN2* | 2 | 4.77E-02 | 4.77E-02 | 8.13E-01 | 8.25E-01 | 1.00E+00 | NA | 9.72E-01 | 9.63E-01 |
| 50 | rs2290324 | *USP34* | 2 | 3.05E-02 | 5.48E-01 | 3.05E-02 | 9.86E-01 | 9.63E-01 | 1.28E-01 | 9.74E-01 | 9.90E-01 |
| 53 | rs11696093 | *CAPG* | 2 | 3.71E-02 | 8.49E-01 | 8.50E-01 | 4.09E-01 | 3.71E-02 | 8.71E-01 | 9.22E-01 | 1.00E+00 |
| 67 | rs6758317 | *ATG16L1* | 2 | 2.39E-02 | 2.39E-02 | 9.58E-01 | 9.89E-01 | 8.86E-01 | 1.00E+00 | 9.09E-01 | 1.00E+00 |
| 77 | rs7613875 | *MON1A* | 3 | 5.14E-05 | 5.14E-05 | 6.38E-04 | 9.89E-01 | NA | NA | 9.09E-01 | 9.92E-01 |
|  | rs12496973 | *RBM6* | 3 | 1.52E-04 | 1.52E-04 | 1.59E-04 | 1.00E+00 | NA | NA | 9.03E-01 | 1.00E+00 |
| 78 | rs2336142 | *PB1* | 3 | 3.36E-02 | 7.91E-01 | 3.36E-02 | 4.85E-01 | 5.33E-01 | 8.07E-01 | 1.00E+00 | 1.00E+00 |
| 79 | rs2336725 | *RFT1* | 3 | 3.72E-03 | 3.72E-03 | 8.86E-01 | 1.00E+00 | 5.33E-01 | 8.71E-01 | 8.94E-01 | 1.00E+00 |
| 96 | rs233816 | *SLC39A8* | 4 | 1.30E-03 | 1.30E-03 | 7.87E-01 | 9.28E-01 | NA | NA | 9.76E-01 | 9.65E-01 |
| 119 | rs272885 | *SLC22A4* | 5 | 1.62E-02 | 1.62E-02 | 8.22E-01 | 8.85E-01 | NA | NA | 8.94E-01 | 9.57E-01 |
| 120 | rs274547 | *SLC22A5* | 5 | 4.95E-02 | 4.95E-02 | 4.57E-01 | 8.32E-01 | 5.28E-01 | 2.16E-01 | 9.23E-01 | 9.85E-01 |
| 126* | rs3734523 | *SLC17A2* | 6 | 2.13E-04 | 9.77E-01 | 8.50E-01 | 5.43E-01 | 2.13E-04 | 3.40E-04 | 9.11E-01 | 1.00E+00 |
| 127* | rs2249099 | *TRIM31* | 6 | 2.01E-02 | 2.01E-02 | 7.87E-01 | 3.66E-02 | NA | NA | 1.10E-01 | 1.00E+00 |
|  | rs2844651 | *SFTA2* | 6 | 5.00E-02 | 8.57E-01 | 7.95E-01 | 5.69E-02 | 5.00E-02 | 5.70E-02 | 1.03E-01 | 1.00E+00 |
|  | rs3095089 | *DPCR1* | 6 | 1.44E-02 | 7.91E-01 | 9.58E-01 | 7.42E-01 | 1.44E-02 | NA | 3.76E-01 | 1.00E+00 |
|  | rs2523855 | *HCG22* | 6 | 3.71E-02 | NA | 3.07E-01 | 5.43E-01 | 3.71E-02 | NA | NA | NA |
|  | rs3823417 | *PSORS1C1* | 6 | 1.90E-02 | 2.98E-02 | 8.50E-01 | 1.90E-02 | NA | NA | 6.32E-02 | 9.11E-01 |
|  | rs2524084 | *HLA-B* | 6 | 4.95E-02 | 4.95E-02 | 8.57E-01 | 8.70E-02 | NA | NA | 1.23E-01 | 9.65E-01 |
|  | rs2523554 | *HLA-B* | 6 | 2.53E-03 | 2.53E-03 | 6.85E-01 | 8.37E-01 | 6.53E-03 | 6.86E-03 | 6.11E-01 | 5.29E-01 |
|  | rs2844571 | *HLA-B* | 6 | 1.32E-02 | 1.05E-01 | 5.38E-01 | 1.32E-02 | NA | NA | 5.48E-02 | 9.92E-01 |
|  | rs2844513 | *MICA* | 6 | 7.09E-04 | 5.17E-02 | 4.63E-01 | 7.09E-04 | 1.44E-03 | 1.99E-03 | 2.04E-02 | 9.65E-01 |
|  | rs9469027 | *LST1* | 6 | 3.66E-02 | NA | NA | 3.66E-02 | NA | NA | NA | NA |
|  | rs3117583 | *BAT3* | 6 | 2.14E-03 | 1.76E-01 | 7.39E-01 | 2.14E-03 | NA | NA | 4.70E-01 | 1.00E+00 |
|  | rs1150755 | *TNXB* | 6 | 4.48E-03 | 4.48E-03 | 3.42E-01 | 8.85E-01 | NA | NA | 5.16E-02 | 1.00E+00 |
|  | rs396960 | *NOTCH4* | 6 | 9.02E-03 | NA | NA | 9.02E-03 | NA | NA | NA | NA |
|  | rs507778 | *AK123889* | 6 | 2.68E-02 | 8.49E-01 | 7.87E-01 | 2.68E-02 | NA | NA | 8.79E-01 | 7.44E-01 |
|  | rs412657 | *AK123889* | 6 | 4.91E-02 | 6.22E-01 | 8.50E-01 | 4.92E-02 | NA | 4.91E-02 | 8.94E-01 | 5.82E-01 |
|  | rs411326 | *AK123889* | 6 | 2.04E-02 | 9.77E-01 | 2.04E-02 | 4.25E-02 | 3.71E-02 | 4.18E-02 | 1.00E+00 | 6.85E-01 |
|  | rs5007265 | *BTNL2* | 6 | 1.96E-02 | 1.96E-02 | 2.04E-02 | 4.25E-02 | NA | NA | 9.03E-02 | 1.39E-01 |
|  | rs3135353 | *HLA-DRA* | 6 | 6.12E-03 | 7.35E-03 | 1.22E-02 | 6.12E-03 | 6.53E-03 | NA | 4.20E-02 | 5.41E-01 |
|  | rs9268853 | *HLA-DRA* | 6 | 2.49E-02 | 9.77E-01 | 2.49E-02 | 4.92E-02 | NA | NA | 9.67E-02 | 3.22E-01 |
|  | rs12194148 | *HLA-DRA* | 6 | 3.62E-04 | NA | NA | 3.62E-04 | NA | NA | NA | NA |
|  | rs3828800 | *HLA-DQB1* | 6 | 2.27E-02 | NA | NA | 2.27E-02 | NA | NA | NA | NA |
|  | rs7774434 | *HLA-DQB1* | 6 | 1.04E-02 | 9.77E-01 | 8.79E-02 | 1.09E-02 | NA | 1.04E-02 | 9.11E-01 | 5.53E-01 |
|  | rs9275312 | *HLA-DQB1* | 6 | 4.63E-02 | 7.22E-02 | 4.63E-02 | 7.56E-02 | NA | 7.47E-02 | 9.08E-01 | 9.81E-01 |
|  | rs11752643 | *HLA-DQB1* | 6 | 1.72E-03 | NA | NA | 1.72E-03 | 2.74E-03 | 3.45E-03 | NA | NA |
|  | rs9275572 | *HLA-DQA2* | 6 | 1.72E-03 | 4.44E-01 | 5.82E-01 | 1.72E-03 | NA | 3.45E-03 | 2.55E-02 | 1.09E-02 |
|  | rs9276644 | *HLA-DQB2* | 6 | 4.31E-02 | 2.94E-01 | 9.58E-01 | 8.82E-02 | 4.31E-02 | 4.91E-02 | 9.09E-01 | 8.56E-01 |
|  | rs7383287 | *HLA-DOB* | 6 | 4.31E-02 | 4.44E-01 | 8.22E-01 | 4.92E-02 | 4.31E-02 | NA | 9.11E-01 | 1.00E+00 |
| 128* | rs241456 | *TAP2* | 6 | 2.90E-02 | 2.90E-02 | 3.05E-02 | 5.69E-02 | NA | NA | 1.03E-01 | 5.46E-01 |
| 141 | rs2489623 | *RSPO3* | 6 | 2.53E-03 | 2.53E-03 | 9.58E-01 | 9.64E-01 | 3.89E-01 | NA | 9.09E-01 | 1.00E+00 |
| 152 | rs836472 | *RAC1* | 7 | 2.12E-03 | 4.90E-01 | 2.12E-03 | 5.72E-01 | 7.59E-01 | 8.36E-01 | 9.11E-01 | 9.57E-01 |
| 162 | rs4917014 | *IKZF1* | 7 | 6.06E-03 | 6.06E-03 | 1.46E-01 | 3.69E-01 | 5.35E-02 | 8.36E-01 | 9.76E-01 | 9.92E-01 |
|  | rs11185603 | *IKZF1* | 7 | 1.39E-02 | NA | 4.63E-01 | 4.48E-01 | 1.39E-02 | NA | NA | NA |
| 164 | rs1178979 | *BAZ1B* | 7 | 3.60E-02 | 3.60E-02 | 7.39E-01 | 9.89E-01 | NA | NA | 9.76E-01 | 9.57E-01 |
| 171 | rs6956357 | *CALD1* | 7 | 3.00E-02 | NA | NA | 7.88E-01 | 8.06E-01 | 3.00E-02 | NA | NA |
| 179 | rs35335364 | *DLC1* | 8 | 3.76E-02 | NA | 3.76E-02 | 9.82E-01 | NA | NA | 8.21E-01 | 9.89E-01 |
| 200 | rs2223103 | *TRPS1* | 8 | 5.77E-03 | 9.02E-01 | 5.77E-03 | 1.00E+00 | 9.63E-01 | NA | 9.11E-01 | 9.11E-01 |
| 202 | rs4871187 | *DQ589334* | 8 | 3.73E-02 | 3.73E-02 | 1.00E+00 | 9.65E-01 | NA | NA | 1.00E+00 | 9.89E-01 |
| 214 | rs2492358 | *C9orf144B* | 9 | 1.90E-02 | 4.01E-01 | 8.86E-01 | 1.90E-02 | 3.89E-01 | 9.07E-01 | 9.76E-01 | 8.56E-01 |
| 217 | rs1421144 | *GKAP1* | 9 | 4.18E-02 | 1.00E+00 | 8.22E-01 | 7.88E-01 | 8.86E-01 | 4.18E-02 | 9.76E-01 | 9.57E-01 |
| 228 | rs167565 | *C9orf84* | 9 | 4.48E-02 | 8.49E-01 | 4.63E-01 | 6.94E-01 | 4.48E-02 | 9.07E-01 | 9.09E-01 | 9.57E-01 |
| 266 | rs102275 | *C11orf10* | 11 | 5.53E-05 | 5.53E-05 | 7.39E-01 | 4.85E-01 | 4.83E-01 | 3.62E-01 | 9.09E-01 | 1.00E+00 |
|  | rs968567 | *FADS2* | 11 | 6.12E-03 | 1.49E-01 | 8.86E-01 | 6.12E-03 | 2.81E-01 | 3.62E-01 | 9.22E-01 | 1.00E+00 |
| 267 | rs2727261 | *BEST1* | 11 | 1.96E-02 | 1.96E-02 | 6.85E-01 | 6.94E-01 | 5.33E-01 | 8.78E-01 | 9.76E-01 | 9.57E-01 |
| 269 | rs476037 | *SLC22A12* | 11 | 4.31E-02 | 5.35E-01 | 5.38E-01 | 9.57E-01 | 4.31E-02 | 7.71E-01 | 9.22E-01 | 1.00E+00 |
| 285 | rs473465 | *INHBE* | 12 | 5.35E-04 | 1.00E+00 | 5.35E-04 | 6.08E-01 | NA | NA | 9.76E-01 | 1.00E+00 |
| 291 | rs653178 | *ATXN2* | 12 | 3.17E-04 | NA | 2.76E-01 | 1.58E-01 | 3.17E-04 | 4.88E-04 | 7.95E-01 | 1.00E+00 |
| 292 | rs7315519 | *RPH3A* | 12 | 4.31E-02 | NA | 7.87E-01 | 9.89E-01 | 4.31E-02 | 8.36E-01 | 8.94E-01 | 9.85E-01 |
| 314 | rs1522772 | *BC037952* | 15 | 3.10E-02 | 9.77E-01 | 8.50E-01 | 8.37E-01 | 3.10E-02 | 1.00E+00 | 8.87E-01 | 9.65E-01 |
| 315 | rs3743031 | *RPAP1* | 15 | 1.84E-02 | 4.01E-01 | 1.84E-02 | 9.57E-01 | NA | NA | 6.32E-01 | 1.00E+00 |
| 319 | rs2899624 | *ALDH1A2* | 15 | 4.88E-03 | 9.02E-01 | 4.88E-03 | 8.85E-01 | 9.63E-01 | 8.78E-01 | 9.76E-01 | 9.85E-01 |
| 341 | rs243315 | *C16orf75* | 16 | 4.95E-02 | 4.95E-02 | 1.61E-01 | 5.20E-01 | 1.35E-01 | NA | 9.26E-01 | 1.00E+00 |
| 344 | rs12448482 | *NPIPL2* | 16 | 6.06E-03 | 6.06E-03 | 3.42E-01 | 4.48E-01 | NA | NA | 9.76E-01 | 1.00E+00 |
| 371 | rs2927299 | *CMIP* | 16 | 4.10E-02 | 6.74E-01 | 4.10E-02 | 6.94E-01 | NA | NA | 7.53E-01 | 9.92E-01 |
| 377 | rs879606 | *PPP1R1B* | 17 | 8.96E-03 | 8.96E-03 | 1.46E-01 | 6.54E-02 | 2.81E-01 | 9.82E-01 | 8.87E-01 | 9.92E-01 |
|  | rs9972882 | *STARD3* | 17 | 1.34E-02 | 1.34E-02 | 2.25E-02 | 1.58E-01 | NA | NA | 9.11E-01 | 9.57E-01 |
|  | rs907092 | *IKZF3* | 17 | 8.36E-06 | 1.59E-05 | 8.36E-06 | 4.65E-03 | 6.53E-04 | 1.00E+00 | 9.76E-01 | 1.00E+00 |
|  | rs9635726 | *IKZF3* | 17 | 7.70E-03 | 7.70E-03 | 8.04E-03 | 2.52E-01 | 4.48E-02 | 8.78E-01 | 1.00E+00 | 9.65E-01 |
|  | rs2872507 | *ZPBP2* | 17 | 3.19E-06 | 3.19E-06 | 3.20E-06 | 7.04E-03 | 5.23E-05 | 9.82E-01 | 9.22E-01 | 1.00E+00 |
|  | rs1008723 | *GSDMB* | 17 | 2.26E-05 | 1.77E-04 | 7.05E-05 | 1.89E-02 | 2.26E-05 | NA | 1.00E+00 | 1.00E+00 |
|  | rs2302776 | *MED24* | 17 | 1.22E-02 | 6.74E-01 | 2.25E-02 | 9.57E-01 | 1.22E-02 | 6.88E-01 | 9.09E-01 | 1.00E+00 |
|  | rs11078936 | *THRAP4* | 17 | 9.69E-03 | 2.94E-01 | 9.69E-03 | 1.00E+00 | 1.99E-02 | 8.66E-01 | 9.11E-01 | 9.62E-01 |
|  | rs868150 | *MED24* | 17 | 3.60E-02 | 3.60E-02 | 1.05E-01 | 9.64E-01 | 2.13E-01 | 8.71E-01 | 9.11E-01 | 9.65E-01 |
| 383 | rs10491182 | *NFE2L1* | 17 | 8.10E-03 | 7.31E-01 | 3.07E-01 | 8.85E-01 | 7.59E-01 | 8.10E-03 | 9.03E-01 | 1.00E+00 |
| 396 | rs1787328 | *MYO5B* | 18 | 1.56E-02 | NA | 6.85E-01 | 8.37E-01 | 7.04E-01 | 1.56E-02 | 7.95E-01 | 1.00E+00 |
| 406 | rs873016 | *DNM2* | 19 | 3.38E-02 | 9.77E-01 | 1.00E+00 | 9.89E-01 | NA | NA | 3.38E-02 | 1.00E+00 |
| 414 | rs12975577 | *CEBPA* | 19 | 2.90E-02 | 2.90E-02 | 4.46E-02 | 9.62E-01 | NA | NA | 8.95E-01 | NA |
| 426 | rs4802370 | *GLTSCR1* | 19 | 2.45E-02 | 2.45E-02 | 8.86E-01 | 9.89E-01 | 9.63E-01 | 8.36E-01 | 8.79E-01 | 9.92E-01 |
| 435 | rs7272152 | *SLC24A3* | 20 | 4.92E-02 | NA | NA | 4.92E-02 | NA | NA | NA | NA |
| 439 | rs6073240 | *TOX2* | 20 | 3.71E-02 | NA | 8.50E-01 | 9.89E-01 | 3.71E-02 | NA | NA | NA |
| 441 | rs4810482 | *MMP9* | 20 | 4.33E-02 | 4.33E-02 | 8.22E-01 | 6.08E-01 | 8.44E-01 | 9.82E-01 | 9.76E-01 | 9.92E-01 |
| 442 | rs2868764 | *NCOA5* | 20 | 3.36E-02 | 7.91E-01 | 4.24E-01 | 3.36E-02 | 8.86E-01 | NA | 9.11E-01 | 9.65E-01 |
| 448 | rs181360 | *UBE2L3* | 22 | 8.76E-07 | 8.76E-07 | 5.77E-03 | 4.02E-02 | 1.74E-01 | NA | 5.66E-01 | 1.00E+00 |
|  | rs181362 | *UBE2L3* | 22 | 7.15E-07 | 7.15E-07 | 8.28E-03 | 4.77E-02 | NA | NA | 5.66E-01 | 1.00E+00 |
|  | rs5754217 | *UBE2L3* | 22 | 1.57E-06 | 1.57E-06 | 1.00E-02 | 5.60E-02 | 1.93E-01 | 8.59E-05 | 5.66E-01 | 1.00E+00 |
|  | rs12158299 | *UBE2L3* | 22 | 6.10E-06 | 6.10E-06 | 3.00E-03 | 5.60E-02 | NA | NA | 5.41E-01 | 1.00E+00 |
| 450 | rs41176 | *MTMR3* | 22 | 1.09E-02 | 1.09E-02 | 2.76E-01 | 6.94E-01 | 1.99E-02 | NA | 8.94E-01 | 1.00E+00 |
|  | rs2412971 | *HORMAD2* | 22 | 1.11E-02 | 1.11E-02 | 2.25E-02 | 4.09E-01 | 2.34E-02 | 6.41E-01 | 9.22E-01 | 1.00E+00 |
|  | rs713875 | *HORMAD2* | 22 | 7.70E-03 | 7.70E-03 | 1.05E-01 | 3.29E-01 | NA | NA | 9.11E-01 | 1.00E+00 |
| 454 | rs1569501 | *RPL3* | 22 | 5.35E-04 | 1.34E-02 | 5.35E-04 | 4.09E-01 | NA | NA | 9.76E-01 | 1.00E+00 |
|  | rs137618 | *RPL3* | 22 | 5.37E-03 | 5.37E-03 | 1.96E-01 | 5.43E-01 | 1.93E-01 | 9.07E-01 | 1.00E+00 | 1.00E+00 |
|  | rs1569498 | *RPL3* | 22 | 6.06E-03 | 6.06E-03 | 4.99E-02 | 2.89E-01 | 2.55E-02 | 8.71E-01 | 1.00E+00 | 1.00E+00 |

Independent complex or single gene loci (r^2^ < 0.2) with SNP(s) with a conjunctional FDR (conjFDR) < 0.05 in *both* high density lipoprotein (HDL) *and* in the associated immune mediated disease. All SNPs with a conjFDR value < 0.05 (bidirectional association, i.e. association with the lipid phenotype given the immune mediated disease (condFDR< 0.05) and association with immune-mediated disease given the lipid phenotype (condFDR<0.05)) are listed and sorted in each LD block. For HDL and immune-mediated disease phenotype we defined the most significant SNP in each LD block based on the minimum conjFDR (min conjFDR). For comparison, the conjFDR values for each identified SNP are listed for all phenotypes, and the minimum (min) conjFDR across all phenotypes. All independent loci are listed consecutively, and Major Histocomaptibility Complex loci are marked (*).All data were first corrected for genomic inflation. Crohn’s Disease (CD), ulcerative colitis (UC), rheumatoid arthritis (RA), type 1 diabetes (T1D), celiac disease (CeD), psoriasis (PSOR) and sarcoidosis (SARC), chromosome (chr), NA; not available.

### Table K. Independent complex or single gene loci (r2 < 0.2) with z-scores for each significant pleiotropic loci from table E. in immune-mediated diseases and triglycerides (TG).

| **#locus** | **SNP** | **gene** | **chr** | **A1** | **A2** | **TG z** | **CD z** | **UC z** | **RA z** | **T1Dz** | | | **CeD z** | | **PSOR z** | | **SARC z** | |
| --- | --- | --- | --- | --- | --- | --- | --- | --- | --- | --- | --- | --- | --- | --- | --- | --- | --- | --- |
| 12 | rs4409689 | *DOCK7* | 1 | C | T | -1.28E+01 | 3.86E+00 | -5.59E-01 | -8.62E-01 | | NA | 2.26E+00 | | -6.06E-01 | | 1.05E+00 | |  |
| 42 | rs1260326 | *GCKR* | 2 | T | C | 2.50E+01 | 3.57E+00 | 3.29E+00 | -1.26E+00 | | NA | 1.31E+00 | | 9.08E-01 | | 4.50E-01 | |  |
|  | rs2068834 | *ZNF512* | 2 | T | C | -1.69E+01 | -1.97E+00 | -4.42E+00 | 1.24E+00 | | NA | -6.16E-01 | | -2.87E-01 | | 1.84E-01 | |  |
|  | rs898031 | *BRE* | 2 | C | T | 6.12E+00 | 1.23E+00 | 4.30E+00 | -4.71E-01 | | NA | -1.70E-01 | | 1.74E+00 | | 1.97E+00 | |  |
|  | rs4401177 | *BRE* | 2 | G | A | -8.84E+00 | -3.79E+00 | -9.33E-01 | -7.20E-01 | | NA | -1.14E+00 | | -2.71E+00 | | -1.10E+00 | |  |
|  | rs12617913 | *BRE* | 2 | C | T | -7.26E+00 | -3.75E+00 | -3.11E+00 | -1.16E+00 | | NA | NA | | -1.52E+00 | | 6.38E-01 | |  |
| 44 | rs2338437 | *FOSL2* | 2 | C | T | 4.87E+00 | 3.94E+00 | 2.03E+00 | -4.43E-01 | | NA | 5.48E-01 | | 3.69E-01 | | -8.13E-01 | |  |
| 75 | rs310751 | *NR_003112* | 3 | T | C | 3.72E+00 | 4.30E-01 | -3.72E-01 | 2.25E+00 | | NA | NA | | 8.97E-01 | | 5.31E-01 | |  |
| 139 | rs272869 | *SLC22A4* | 5 | A | G | 3.88E+00 | -4.54E+00 | -2.41E-01 | -6.87E-01 | | NA | NA | | -6.46E-01 | | 6.56E-01 | |  |
| 142 | rs7724832 | *TIMD4* | 5 | G | A | -6.71E+00 | 3.98E+00 | 1.74E+00 | 1.12E+00 | | NA | NA | | -1.05E+00 | | -1.65E+00 | |  |
| 149 | rs6904596 | *ZNF184* | 6 | G | A | 3.62E+00 | 2.16E+00 | 1.95E+00 | -1.37E+00 | | NA | -3.10E+01 | | 3.89E+00 | | 6.98E-01 | |  |
| 150 | rs9277936 | *RING1* | 6 | T | A | NA | NA | NA | NA | | NA | NA | | NA | | NA | |  |
| 151 | rs462093 | *WDR46* | 6 | A | T | NA | NA | NA | NA | | NA | NA | | NA | | NA | |  |
| 166 | rs2503322 | *RSPO3* | 6 | A | G | -4.06E+00 | -4.19E+00 | 8.19E-01 | -2.31E-01 | | NA | 1.62E+00 | | -9.14E-01 | | 1.76E-01 | |  |
| 205 | rs3021494 | *XKR6* | 8 | A | G | -4.00E+00 | 1.18E+00 | -1.04E+00 | 2.61E-01 | | NA | NA | | -4.37E+00 | | -6.38E-01 | |  |
|  | rs9644737 | *C8orf12* | 8 | A | G | 3.64E+00 | NA | -5.90E-01 | -4.41E-01 | | NA | -3.27E-02 | | -1.62E-01 | | 2.46E-01 | |  |
|  | rs998683 | *BLK* | 8 | G | A | 3.66E+00 | -1.36E+00 | 1.14E+00 | -3.61E+00 | | NA | NA | | -3.96E-01 | | -1.52E+00 | |  |
|  | rs1296023 | *CTSB* | 8 | A | G | 3.86E+00 | -4.61E-02 | 3.53E+00 | 8.27E-01 | | NA | NA | | -2.58E-01 | | 3.14E-01 | |  |
| 207 | rs1961456 | *NAT2* | 8 | A | G | -6.75E+00 | -9.33E-01 | -6.94E-01 | -3.82E+00 | | NA | NA | | -1.55E+00 | | 1.72E+00 | |  |
| 211 | rs2278615 | *INTS10* | 8 | A | G | 3.53E+00 | NA | NA | -3.83E+00 | | NA | NA | | NA | | NA | |  |
| 277 | rs102275 | *C11orf10* | 11 | T | C | -1.02E+01 | -4.95E+00 | -1.08E+00 | 2.14E+00 | | NA | 2.40E+00 | | -1.05E+00 | | 1.42E+00 | |  |
|  | rs968567 | *FADS2* | 11 | C | T | -5.12E+00 | -2.57E+00 | 5.72E-01 | 4.25E+00 | | NA | 2.42E+00 | | 4.91E-01 | | 1.39E+00 | |  |
| 311 | rs7297186 | *CUX2* | 12 | T | C | 3.64E+00 | NA | 6.49E-01 | 1.66E+00 | | NA | 3.31E+00 | | 2.74E-01 | | 1.09E-01 | |  |
|  | rs7398833 | *CUX2* | 12 | T | C | 3.77E+00 | NA | 6.69E-01 | 1.74E+00 | | NA | NA | | -2.19E-02 | | -9.36E-02 | |  |
| 363 | rs8062719 | *STX1B* | 16 | A | G | 4.25E+00 | -6.94E-01 | -1.65E+00 | 6.72E-02 | | NA | NA | | 4.38E+00 | | 6.72E-01 | |  |
| 391 | rs7221651 | *BPTF* | 17 | G | A | -4.02E+00 | 4.04E-01 | -6.44E-02 | 7.84E-01 | | NA | NA | | -2.40E-01 | | 8.36E-02 | |  |
| 409 | rs2965185 | *GATAD2A* | 19 | T | C | 6.10E+00 | -6.20E-01 | -2.91E-01 | -3.92E+00 | | NA | NA | | -8.23E-01 | | -1.03E+00 | |  |
| 425 | rs676388 | *FUT2* | 19 | T | C | -4.03E+00 | -5.48E+00 | 8.98E-02 | 2.77E-02 | | NA | -2.76E+00 | | -2.69E+00 | | -2.30E+00 | |  |

All independent loci are listed consecutively, and the same locus numbers are as in Table E. Crohn’s Disease (CD), ulcerative colitis (UC), rheumatoid arthritis (RA), type 1 diabetes (T1D), celiac disease (CeD), psoriasis (PSOR), sarcoidosis (SARC), chromosome (chr). NA; not available. All data were first corrected for genomic inflation.

### Table L. Independent complex or single gene loci (r^2^ < 0.2) with z-scores for each significant pleiotropic loci from Table F. in immune-mediated diseases and low density lipoprotein (LDL).

| **#locus** | **SNP** | **gene** | **chr** | **A1** | **A2** | **LDL z** | **CD z** | **UC z** | **RA z** | **T1D z** | **CeD z** | **PSOR z** | **SARC z** |
| --- | --- | --- | --- | --- | --- | --- | --- | --- | --- | --- | --- | --- | --- |
| 23 | rs4409689 | *DOCK7* | 1 | C | T | -8.42E+00 | 3.86E+00 | -5.59E-01 | -8.62E-01 | NA | 2.26E+00 | -6.06E-01 | 1.05E+00 |
| 38 | rs2938616 | *EPS8L3* | 1 | T | G | -4.11E+00 | -1.82E+00 | -4.24E+00 | 3.48E-01 | NA | 6.81E-01 | 8.23E-01 | 6.67E-01 |
| 44 | rs7551957 | *FCGR2A* | 1 | T | C | 4.28E+00 | NA | NA | 3.88E+00 | NA | NA | NA | NA |
|  | rs1801274 | *FCGR2A* | 1 | A | G | 3.75E+00 | 2.34E+00 | 8.49E+00 | 4.08E+00 | NA | -4.33E-01 | -2.07E+00 | -6.63E-01 |
| 82 | rs932206 | *CXCR4* | 2 | C | T | 4.11E+00 | 4.30E-01 | 1.72E+00 | -1.63E+00 | NA | 3.76E+00 | 1.24E+00 | -2.27E-01 |
| 99 | rs299639 | *TSEN2* | 3 | A | C | 4.40E+00 | -1.73E+00 | -1.26E-01 | -2.11E+00 | NA | -7.50E-02 | 3.64E-01 | -1.60E-01 |
| 136 | rs6878664 | *CTNND2* | 5 | A | G | 3.75E+00 | -5.91E-01 | 3.67E-01 | -4.19E+00 | NA | -2.34E+00 | 0.00E+00 | 2.65E-01 |
| 151 | rs9282763 | *LOC441108* | 5 | T | C | -4.03E+00 | 5.30E+00 | 2.01E+00 | 1.38E+00 | NA | NA | -3.27E-01 | 8.64E-01 |
| 155 | rs7724832 | *TIMD4* | 5 | G | A | -8.95E+00 | 3.98E+00 | 1.74E+00 | 1.12E+00 | NA | NA | -1.05E+00 | -1.65E+00 |
| 166 | rs12216125 | *TRIM38* | 6 | C | T | 3.71E+00 | 1.18E+00 | 3.88E-01 | 1.26E+00 | NA | 5.88E+00 | -1.16E+00 | 1.13E+00 |
|  | rs16891235 | *HIST1H1A* | 6 | T | C | -3.64E+00 | -2.93E-01 | 6.54E-01 | -1.70E+00 | NA | -1.98E+01 | 7.54E-01 | 3.15E-02 |
|  | rs13194984 | *BTN1A1* | 6 | G | T | 4.22E+00 | -5.22E-01 | 1.64E+00 | 7.70E-01 | NA | 3.11E+00 | -9.09E-01 | -1.53E+00 |
|  | rs13194491 | *C6orf41* | 6 | C | T | 3.87E+00 | NA | NA | 3.95E-02 | NA | 3.81E+00 | NA | NA |
|  | rs13214703 | *OR2B6* | 6 | T | C | 3.90E+00 | NA | NA | -2.57E-02 | NA | 3.64E+00 | NA | NA |
| 167 | rs3893464 | *HLA-G* | 6 | G | A | -3.94E+00 | NA | NA | 5.03E+00 | NA | -1.47E+01 | NA | NA |
|  | rs9378220 | *TRIM31* | 6 | C | A | -4.19E+00 | NA | NA | 3.17E+00 | NA | 1.05E+01 | NA | NA |
| 168 | rs1264344 | *C6orf214* | 6 | C | T | -3.91E+00 | 2.40E+00 | -2.23E-01 | -8.01E+00 | NA | 2.66E+01 | 4.47E+00 | -1.27E-01 |
| 169 | rs6930777 | *BTNL2* | 6 | C | T | -6.74E+00 | NA | -7.16E+00 | -6.11E+00 | NA | NA | NA | NA |
| 170 | rs2281389 | *HLA-DPB1* | 6 | A | G | -3.85E+00 | 1.01E+00 | 2.60E+00 | 5.52E+00 | NA | -5.09E-01 | 2.60E+00 | -2.53E+00 |
|  | rs2064473 | *HLA-DPB1* | 6 | G | A | -4.08E+00 | NA | NA | 5.31E+00 | NA | NA | NA | NA |
| 234 | rs1961456 | *NAT2* | 8 | A | G | -3.78E+00 | -9.33E-01 | -6.94E-01 | -3.82E+00 | NA | NA | -1.55E+00 | 1.72E+00 |
| 288 | rs11187157 | *HHEX* | 10 | T | C | 4.05E+00 | NA | -4.73E+00 | 2.56E-01 | NA | NA | -8.36E-01 | 5.16E-01 |
| 300 | rs102275 | *C11orf10* | 11 | T | C | 9.63E+00 | -4.95E+00 | -1.08E+00 | 2.14E+00 | NA | 2.40E+00 | -1.05E+00 | 1.42E+00 |
|  | rs968567 | *FADS2* | 11 | C | T | 5.20E+00 | -2.57E+00 | 5.72E-01 | 4.25E+00 | NA | 2.42E+00 | 4.91E-01 | 1.39E+00 |
| 331 | rs3184504 | *SH2B3* | 12 | T | C | -6.17E+00 | NA | NA | 2.88E+00 | NA | 7.22E+00 | NA | NA |
|  | rs630512 | *ATXN2* | 12 | C | T | -5.18E+00 | NA | 9.07E-01 | 1.55E+00 | NA | 2.40E+00 | 1.94E+00 | 8.73E-01 |
|  | rs11066320 | *PTPN11* | 12 | A | G | -5.80E+00 | NA | 2.58E+00 | 2.81E+00 | NA | 5.10E+00 | 1.77E+00 | 5.47E-01 |
| 365 | rs2472304 | *CYP1A2* | 15 | G | A | -3.86E+00 | -4.56E-01 | -6.91E-01 | 6.84E-01 | NA | 3.61E+00 | NA | 3.97E-01 |
| 424 | rs7253253 | *SLC44A2* | 19 | G | T | -4.52E+00 | 4.02E+00 | 2.87E+00 | -1.38E+00 | NA | NA | -2.52E+00 | -8.12E-01 |
|  | rs12974306 | *DNM2* | 19 | G | T | -3.90E+00 | 1.23E+00 | -2.07E-02 | 1.65E+00 | NA | 3.30E-02 | -4.01E+00 | -1.90E+00 |
|  | rs11085749 | *C19orf38* | 19 | G | A | 7.45E+00 | 7.73E-01 | -9.56E-02 | 3.95E-01 | NA | -1.14E+00 | 4.37E+00 | 1.36E+00 |
|  | rs12983316 | *SMARCA4* | 19 | A | G | -8.98E+00 | NA | -2.42E-01 | -4.57E-01 | NA | NA | -4.49E+00 | -2.21E+00 |
| 427 | rs7188 | *KANK2* | 19 | A | C | -8.32E+00 | -1.39E+00 | 3.76E-01 | 3.91E+00 | NA | 1.10E+00 | 4.23E-01 | -1.56E+00 |
| 432 | rs2965185 | *GATAD2A* | 19 | T | C | 4.82E+00 | -6.20E-01 | -2.91E-01 | -3.92E+00 | NA | NA | -8.23E-01 | -1.03E+00 |
| 454 | rs516246 | *FUT2* | 19 | C | T | -5.46E+00 | -5.50E+00 | -8.40E-02 | -3.95E-03 | NA | NA | -2.99E+00 | -2.30E+00 |
|  | rs485186 | *FUT2* | 19 | A | G | -4.91E+00 | -5.48E+00 | 2.18E-02 | -6.68E-01 | NA | -2.61E+00 | -2.70E+00 | -2.35E+00 |
| 483 | rs4820821 | *ASCC2* | 22 | C | A | -3.73E+00 | -2.71E+00 | -6.94E-01 | 1.01E+00 | NA | NA | -1.95E+00 | -1.05E+00 |

All independent loci are listed consecutively, and the same locus numbers are used as in Table F. Crohn’s Disease (CD), ulcerative colitis (UC), rheumatoid arthritis (RA), type 1 diabetes (T1D), celiac disease (CeD), psoriasis (PSOR), sarcoidosis (SARC), chromosome (chr). NA; not available. All data were first corrected for genomic inflation.

### Table M. Independent complex or single gene loci (r2 < 0.2) with z-scores for each significant pleiotropic locus from Table G in immune-mediated diseases and high density lipoprotein (HDL).

| #Locus | SNP | gene | chr | A1 | A2 | HDL z | CD z | UC z | RA z | T1D z | CeD z | PSOR z | SARC z |
| --- | --- | --- | --- | --- | --- | --- | --- | --- | --- | --- | --- | --- | --- |
| 31 | rs3813982 | *SRGAP2* | 1 | A | G | -3.63E+00 | 1.04E-01 | -3.91E+00 | 3.22E+00 | NA | 1.10E+00 | 6.51E-01 | -4.66E-01 |
| 45 | rs17799872 | *ADCY3* | 2 | G | A | 3.94E+00 | -1.20E+00 | -9.85E-01 | 8.94E-01 | NA | -9.73E-01 | -1.01E+00 | -2.05E+00 |
| 77 | rs7613875 | *MON1A* | 3 | C | A | 4.92E+00 | 5.18E+00 | 4.48E+00 | -3.02E-01 | NA | NA | 9.37E-01 | 1.88E-01 |
|  | rs12496973 | *RBM6* | 3 | T | C | 4.73E+00 | 4.97E+00 | 4.81E+00 | -3.06E-02 | NA | NA | 1.12E+00 | 8.56E-02 |
| 79 | rs2336725 | *RFT1* | 3 | C | T | 3.89E+00 | 3.98E+00 | -5.17E-01 | 6.13E-02 | NA | 7.66E-01 | -1.23E+00 | 1.28E+00 |
| 96 | rs233816 | *SLC39A8* | 4 | G | T | 4.27E+00 | -4.14E+00 | -1.02E+00 | 8.88E-01 | NA | NA | 3.43E-01 | 6.39E-01 |
| 126 | rs3734523 | *SLC17A2* | 6 | G | A | 4.97E+00 | -2.57E-01 | 7.35E-01 | -2.00E+00 | NA | -2.31E+01 | 6.05E-01 | -6.94E-02 |
| 141 | rs2489623 | *RSPO3* | 6 | A | C | 3.98E+00 | -4.15E+00 | 3.18E-01 | -3.97E-01 | NA | NA | -1.02E+00 | -3.80E-02 |
| 152 | rs836472 | *RAC1* | 7 | G | A | -4.09E+00 | 1.61E+00 | 4.15E+00 | -1.87E+00 | NA | 1.26E+00 | 7.46E-01 | -5.19E-01 |
| 162 | rs4917014 | *IKZF1* | 7 | T | G | -4.31E+00 | -3.71E+00 | -2.71E+00 | 2.44E+00 | NA | -1.32E+00 | 3.14E-01 | -1.76E-01 |
| 200 | rs2223103 | *TRPS1* | 8 | A | T | NA | NA | -3.84E+00 | NA | NA | NA | NA | NA |
| 266 | rs102275 | *C11orf10* | 11 | T | C | 9.91E+00 | -4.95E+00 | -1.08E+00 | 2.14E+00 | NA | 2.40E+00 | -1.05E+00 | 1.42E+00 |
|  | rs968567 | *FADS2* | 11 | C | T | 4.01E+00 | -2.57E+00 | 5.72E-01 | 4.25E+00 | NA | 2.42E+00 | 4.91E-01 | 1.39E+00 |
| 285 | rs473465 | *INHBE* | 12 | G | A | 4.35E+00 | 6.92E-02 | 4.50E+00 | 1.77E+00 | NA | NA | -1.55E-01 | -9.62E-01 |
| 291 | rs653178 | *ATXN2* | 12 | C | T | -4.89E+00 | NA | 2.36E+00 | 2.85E+00 | NA | 7.28E+00 | 1.85E+00 | 1.06E+00 |
| 319 | rs2899624 | *ALDH1A2* | 15 | A | G | 1.04E+01 | 4.04E-01 | -3.88E+00 | 1.05E+00 | NA | -6.68E-01 | 3.17E-01 | -8.20E-01 |
| 344 | rs12448482 | *NPIPL2* | 16 | A | G | 3.79E+00 | -3.73E+00 | -2.13E+00 | -2.22E+00 | NA | NA | 3.50E-01 | -1.24E-01 |
| 377 | rs879606 | *PPP1R1B* | 17 | A | G | -5.11E+00 | -3.57E+00 | -2.75E+00 | -3.28E+00 | NA | 3.50E-01 | 1.40E+00 | -2.18E-01 |
|  | rs907092 | *IKZF3* | 17 | G | A | -5.75E+00 | -5.22E+00 | -5.65E+00 | -4.11E+00 | NA | -1.07E-01 | -3.04E-01 | 1.34E+00 |
|  | rs9635726 | *IKZF3* | 17 | C | T | 3.67E+00 | 4.75E+00 | 4.61E+00 | 2.68E+00 | NA | -7.02E-01 | -3.88E-02 | 6.54E-01 |
|  | rs2872507 | *ZPBP2* | 17 | G | A | -5.66E+00 | -5.55E+00 | -6.02E+00 | -4.02E+00 | NA | 3.45E-01 | -5.45E-01 | 1.30E+00 |
|  | rs1008723 | *GSDMB* | 17 | G | T | -6.24E+00 | -4.66E+00 | -5.07E+00 | -3.71E+00 | NA | NA | -9.51E-02 | 1.03E+00 |
|  | rs11078936 | *THRAP4* | 17 | T | C | -3.62E+00 | -2.12E+00 | -3.89E+00 | -4.35E-02 | NA | 1.04E+00 | 8.78E-01 | 1.70E+00 |
| 383 | rs10491182 | *NFE2L1* | 17 | T | C | -4.18E+00 | -9.33E-01 | -2.28E+00 | 1.02E+00 | NA | -3.77E+00 | -1.08E+00 | -1.22E+00 |
| 448 | rs181360 | *UBE2L3* | 22 | T | G | 5.76E+00 | -6.43E+00 | -3.81E+00 | -3.46E+00 | NA | NA | -2.56E+00 | -9.72E-01 |
|  | rs181362 | *UBE2L3* | 22 | C | T | 5.82E+00 | -6.61E+00 | -3.70E+00 | -3.40E+00 | NA | NA | -2.57E+00 | -1.00E+00 |
|  | rs5754217 | *UBE2L3* | 22 | G | T | 5.67E+00 | -6.59E+00 | -3.67E+00 | -3.35E+00 | NA | -4.81E+00 | -2.56E+00 | -1.00E+00 |
|  | rs12158299 | *UBE2L3* | 22 | C | T | 5.39E+00 | -6.51E+00 | -4.05E+00 | -3.39E+00 | NA | NA | -2.66E+00 | -9.89E-01 |
|  | rs713875 | *HORMAD2* | 22 | C | G | NA | NA | 2.88E+00 | NA | NA | NA | NA | NA |
| 454 | rs1569501 | *RPL3* | 22 | T | A | NA | NA | -4.75E+00 | NA | NA | NA | NA | NA |
|  | rs137618 | *RPL3* | 22 | C | T | -3.76E+00 | 4.02E+00 | 2.59E+00 | 1.93E+00 | NA | -5.33E-01 | 8.60E-02 | 1.40E+00 |
|  | rs1569498 | *RPL3* | 22 | C | T | 3.87E+00 | -3.72E+00 | -3.13E+00 | -2.57E+00 | NA | 8.28E-01 | 9.60E-02 | -1.50E+00 |

All independent loci are listed consecutively, and the same locus numbers are used as in Table G. Crohn’s Disease (CD), ulcerative colitis (UC), rheumatoid arthritis (RA), type 1 diabetes (T1D), celiac disease (CeD), psoriasis (PSOR), sarcoidosis (SARC), chromosome (chr), NA; not available. All data were first corrected for genomic inflation.

### Table N. Correlation coefficients of z-scores.

|  | CD | UC | RA | T1D | CeD | PSOR | SARC |
| --- | --- | --- | --- | --- | --- | --- | --- |
| TG | 0.3783 | 0.1864 | 0.2080 | - | -0.2991 | 0.1898 | -0.3807 |
| LDL | 0.1692 | 0.2244 | 0.0579 | - | -0.2873 | 0.3086 | -0.0899 |
| HDL | 0.1778 | 0.2476 | 0.2863 | - | -0.3370 | 0.0367 | -0.4129 |

Pearson's r for all z-scores (conjFDR < 0.01). Triglycerides (TG), low-density lipoprotein (LDL) cholesterol, high-density lipoprotein (HDL) cholesterol, Crohn’s Disease (CD), ulcerative colitis (UC), rheumatoid arthritis (RA), type 1 diabetes (T1D), celiac disease (CeD), psoriasis (PSOR) and sarcoidosis (SARC).

### Table O. Enriched KEGG pathways in the functional gene network.

| **Pathway name** | **Z Score** | **Adjusted P value** | **Associated genes** |
| --- | --- | --- | --- |
| Fat digestion and absorption: KEGG-hsa04975 | 8.89 | 0.0064 | ABCA1, ABCG5, ABCG8, APOA1, APOB, NPC1L1, SCARB1 |
| Glycosphingolipid biosynthesis - lacto and neolacto series: KEGG-hsa00601 | 8.58 | 0.0064 | ABO, B4GALT1, FUT1, FUT2, ST3GAL4 |
| PPAR signaling pathway: KEGG-hsa03320 | 5.73 | 0.0064 | ANGPTL4, APOA1, CYP7A1, FADS2, PLTP, PPARG |
| Bile secretion: KEGG-hsa04976 | 5.73 | 0.0064 | ABCG5, ABCG8, ADCY3, CYP7A1, LDLR, SCARB1 |
| Vitamin digestion and absorption: KEGG-hsa04977 | 5.17 | 0.0064 | APOA1, APOB, SCARB1 |
| Maturity onset diabetes of the young: KEGG-hsa04950 | 5.04 | 0.0064 | HHEX, HNF1A, HNF4A |
| ABC transporters: KEGG-hsa02010 | 4.90 | 0.0064 | ABCA1, ABCA8, ABCG5, ABCG8 |
| Neurotrophin signaling pathway: KEGG-hsa04722 | 3.76 | 0.0064 | IRS1, MAP3K1, PTPN11, RAC1, RAF1, SH2B3 |
| Melanoma: KEGG-hsa05218 | 3.52 | 0.0064 | CDK6, FGF21, FGF3, RAF1 |
| Chronic myeloid leukemia: KEGG-hsa05220 | 3.44 | 0.0064 | CDK6, PTPN11, RAF1, RUNX1 |
| Adherens junction: KEGG-hsa04520 | 3.44 | 0.0064 | INSR, PTPRM, PVRL2, RAC1 |
| Acute myeloid leukemia: KEGG-hsa05221 | 2.88 | 0.0064 | CEBPA, RAF1, RUNX1 |
| Fc gamma R-mediated phagocytosis: KEGG-hsa04666 | 2.80 | 0.0064 | ASAP2, DNM2, RAC1, RAF1 |
| Pathways in cancer: KEGG-hsa05200 | 2.74 | 0.0064 | CDK6, CEBPA, FGF21, FGF3, MMP9, PPARG, RAC1, RAF1, RUNX1 |
| Renal cell carcinoma: KEGG-hsa05211 | 2.44 | 0.0250 | PTPN11, RAC1, RAF1 |
| Pancreatic cancer: KEGG-hsa05212 | 2.44 | 0.0460 | CDK6, RAC1, RAF1 |
| Leukocyte transendothelial migration: KEGG-hsa04670 | 2.30 | 0.0064 | CXCR4, MMP9, PTPN11, RAC1 |
| Osteoclast differentiation: KEGG-hsa04380 | 2.08 | 0.0115 | FOSL2, ITGB3, PPARG, RAC1 |
| Axon guidance: KEGG-hsa04360 | 2.06 | 0.0115 | CXCR4, PLXNC1, RAC1, SRGAP2 |
| Hepatitis C: KEGG-hsa05160 | 1.97 | 0.0250 | IRF1, LDLR, RAF1, SCARB1 |

### Table P. Enriched KEGG pathways in the protein interaction network.

| **Pathway name** | **Z Score** | **Adjusted P value** | **Associated genes** |
| --- | --- | --- | --- |
| Fat digestion and absorption: KEGG-hsa04975 | 9.60 | 0.0068 | ABCA1, ABCG5, ABCG8, APOA1, APOB, SCARB1 |
| Vitamin digestion and absorption: KEGG-hsa04977 | 6.62 | 0.0068 | APOA1, APOB, SCARB1 |
| Maturity onset diabetes of the young: KEGG-hsa04950 | 6.47 | 0.0068 | HHEX, HNF1A, HNF4A |
| PPAR signaling pathway: KEGG-hsa03320 | 6.11 | 0.0068 | ANGPTL4, APOA1, LPL, PLTP, PPARG |
| Bile secretion: KEGG-hsa04976 | 4.74 | 0.0068 | ABCG5, ABCG8, LDLR, SCARB1 |
| Chronic myeloid leukemia: KEGG-hsa05220 | 4.66 | 0.0068 | CDK6, PTPN11, RAF1, RUNX1 |
| ABC transporters: KEGG-hsa02010 | 4.63 | 0.0068 | ABCA1, ABCG5, ABCG8 |
| Neurotrophin signaling pathway: KEGG-hsa04722 | 4.15 | 0.0068 | IRS1, MAP3K1, PTPN11, RAC1, RAF1 |
| Osteoclast differentiation: KEGG-hsa04380 | 4.13 | 0.0068 | FCGR2A, FOSL2, ITGB3, PPARG, RAC1 |
| Acute myeloid leukemia: KEGG-hsa05221 | 3.92 | 0.0121 | CEBPA, RAF1, RUNX1 |
| Fc gamma R-mediated phagocytosis: KEGG-hsa04666 | 3.92 | 0.0068 | DNM2, FCGR2A, RAC1, RAF1 |
| Renal cell carcinoma: KEGG-hsa05211 | 3.40 | 0.0068 | PTPN11, RAC1, RAF1 |
| Pancreatic cancer: KEGG-hsa05212 | 3.40 | 0.0068 | CDK6, RAC1, RAF1 |
| Leukocyte transendothelial migration: KEGG-hsa04670 | 3.35 | 0.0068 | CXCR4, MMP9, PTPN11, RAC1 |
| Hepatitis C: KEGG-hsa05160 | 2.99 | 0.0068 | IRF1, LDLR, RAF1, SCARB1 |
| Pathways in cancer: KEGG-hsa05200 | 2.95 | 0.0068 | CDK6, CEBPA, MMP9, PPARG, RAC1, RAF1, RUNX1 |
| Insulin signaling pathway: KEGG-hsa04910 | 2.91 | 0.0068 | INSR, IRS1, PPP1R3B, RAF1 |
| Phagosome: KEGG-hsa04145 | 2.66 | 0.0068 | FCGR2A, ITGB3, RAC1, SCARB1 |
| Axon guidance: KEGG-hsa04360 | 2.06 | 0.0157 | CXCR4, RAC1, SRGAP2 |
| Endocytosis: KEGG-hsa04144 | 2.03 | 0.0157 | CXCR4, DNM2, LDLR, LDLRAP1 |

Table Q. Pleiotropic SNPs between high density lipoprotein (HDL) and immune identified by GWAS.

| **SNP** | **Chr** | **Gene** | **Immune Trait** |
| --- | --- | --- | --- |
| rs907092 | 17 | IKZF3 | CD |
| rs9909593 | 17 | IKZF3 | CD |
| rs12936231 | 17 | ZPBP2 | CD |
| rs2872507 | 17 | ZPBP2 | CD |
| rs9901146 | 17 | ZPBP2 | CD |
| rs8067378 | 17 | GSDMB | CD |
| rs8069176 | 17 | GSDMB | CD |
| rs2305480 | 17 | GSDMB | CD |
| rs11078927 | 17 | GSDMB | CD |
| rs4795400 | 17 | GSDMB | CD |
| rs8139142 | 22 | RIMBP3C | CD |
| rs2266959 | 22 | UBE2L3 | CD |
| rs181359 | 22 | UBE2L3 | CD |
| rs181360 | 22 | UBE2L3 | CD |
| rs181362 | 22 | UBE2L3 | CD |
| rs5754217 | 22 | UBE2L3 | CD |
| rs1034329 | 22 | UBE2L3 | CD |
| rs5998619 | 22 | UBE2L3 | CD |
| rs2283790 | 22 | UBE2L3 | CD |
| rs5998672 | 22 | UBE2L3 | CD |
| rs907092 | 17 | IKZF3 | UC |
| rs9909593 | 17 | IKZF3 | UC |
| rs3816470 | 17 | IKZF3 | UC |
| rs2872507 | 17 | ZPBP2 | UC |
| rs9901146 | 17 | ZPBP2 | UC |
| rs8067378 | 17 | GSDMB | UC |
| rs8069176 | 17 | GSDMB | UC |
| rs2305480 | 17 | GSDMB | UC |
| rs2305479 | 17 | GSDMB | UC |
| rs11078927 | 17 | GSDMB | UC |
| rs1008723 | 17 | GSDMB | UC |
| rs4795400 | 17 | GSDMB | UC |
| rs7216389 | 17 | GSDMB | UC |
| rs9303281 | 17 | GSDMB | UC |
| rs7219923 | 17 | GSDMB | UC |
| rs7224129 | 17 | GSDMB | UC |

Table R. Pleiotropic SNPs between low density lipoprotein (LDL) and immune identified by GWAS.

| **SNP** | **Chr** | **Gene** | **Immune Trait** |
| --- | --- | --- | --- |
| rs3763313 | 6 | BTNL2 | CD |
| rs6930777 | 6 | BTNL2 | UC |
| rs17423649 | 6 | BTNL2 | UC |
| rs17495612 | 6 | BTNL2 | UC |
| rs3817969 | 6 | BTNL2 | UC |
| rs4530903 | 6 | HLA-DQA1 | UC |
| rs10484561 | 6 | HLA-DQB1 | UC |
| rs13192471 | 6 | HLA-DQB1 | UC |
| rs6930777 | 6 | BTNL2 | RA |
| rs13218331 | 6 | HLA-DRA | RA |
| rs12529093 | 6 | HLA-DRA | RA |
| rs13209234 | 6 | HLA-DRA | RA |
| rs4530903 | 6 | HLA-DQA1 | RA |
| rs17533167 | 6 | HLA-DQA1 | RA |
| rs17599077 | 6 | HLA-DQA1 | RA |
| rs17499411 | 6 | HLA-DQB1 | RA |
| rs10484561 | 6 | HLA-DQB1 | RA |
| rs9275427 | 6 | HLA-DQB1 | RA |
| rs9275428 | 6 | HLA-DQB1 | RA |
| rs13192471 | 6 | HLA-DQB1 | RA |
| rs9275492 | 6 | HLA-DQA2 | RA |
| rs6930777 | 6 | BTNL2 | T1D |
| rs17423649 | 6 | BTNL2 | T1D |
| rs17495612 | 6 | BTNL2 | T1D |
| rs3763313 | 6 | BTNL2 | T1D |
| rs4530903 | 6 | HLA-DQA1 | T1D |
| rs10484561 | 6 | HLA-DQB1 | T1D |
| rs13192471 | 6 | HLA-DQB1 | T1D |
| rs3184504 | 12 | SH2B3 | T1D |
| rs11065987 | 12 | BRAP | T1D |
| rs17696736 | 12 | NAA25 | T1D |
| rs11066320 | 12 | PTPN11 | T1D |
| rs3763313 | 6 | BTNL2 | CeD |
| rs13209234 | 6 | HLA-DRA | CeD |
| rs10484561 | 6 | HLA-DQB1 | CeD |
| rs9275427 | 6 | HLA-DQB1 | CeD |
| rs9275428 | 6 | HLA-DQB1 | CeD |
| rs13192471 | 6 | HLA-DQB1 | CeD |
| rs3184504 | 12 | SH2B3 | CeD |

Table S. Pleiotropic SNPs between triglycerides (TG) and immune identified by GWAS.

| **SNP** | **Chr** | **Gene** | **Immune Trait** |
| --- | --- | --- | --- |
| rs440454 | 6 | SKIV2L | UC |
| rs437179 | 6 | SKIV2L | UC |
| rs592229 | 6 | SKIV2L | UC |
| rs389883 | 6 | STK19 | UC |
| rs3132550 | 6 | PSORS1C1 | RA |
| rs6921948 | 6 | HCG27 | RA |
| rs2853939 | 6 | HLA-B | RA |
| rs2524053 | 6 | HLA-B | RA |
| rs2524052 | 6 | HLA-B | RA |
| rs2524156 | 6 | HLA-B | RA |
| rs2524066 | 6 | HLA-B | RA |
| rs660594 | 6 | SLC44A4 | RA |
| rs577272 | 6 | SLC44A4 | RA |
| rs644827 | 6 | SLC44A4 | RA |
| rs644774 | 6 | SLC44A4 | RA |
| rs2242665 | 6 | SLC44A4 | RA |
| rs3130481 | 6 | SLC44A4 | RA |
| rs605203 | 6 | SLC44A4 | RA |
| rs589428 | 6 | NG36/G9A | RA |
| rs3869145 | 6 | NG36/G9A | RA |
| rs486416 | 6 | NG36/G9A | RA |
| rs659445 | 6 | NG36/G9A | RA |
| rs644045 | 6 | C2 | RA |
| rs537160 | 6 | CFB | RA |
| rs440454 | 6 | SKIV2L | RA |
| rs437179 | 6 | SKIV2L | RA |
| rs592229 | 6 | SKIV2L | RA |
| rs389883 | 6 | STK19 | RA |
| rs3130309 | 6 | AK123889 | RA |
| rs3115572 | 6 | AK123889 | RA |
| rs3115571 | 6 | AK123889 | RA |
| rs3130316 | 6 | AK123889 | RA |
| rs3096700 | 6 | AK123889 | RA |
| rs3130320 | 6 | AK123889 | RA |
| rs9268144 | 6 | C6orf10 | RA |
| rs9268219 | 6 | C6orf10 | RA |
| rs3129927 | 6 | C6orf10 | RA |
| rs3117103 | 6 | C6orf10 | RA |
| rs3129950 | 6 | BTNL2 | RA |
| rs3129856 | 6 | HLA-DRA | RA |
| rs11752643 | 6 | HLA-DQB1 | RA |
| rs3130544 | 6 | C6orf15 | T1D |
| rs7750641 | 6 | TCF19 | T1D |
| rs3099844 | 6 | HCG26 | T1D |
| rs3117582 | 6 | APOM | T1D |
| rs3130484 | 6 | MSH5 | T1D |
| rs3131379 | 6 | MSH5 | T1D |
| rs644827 | 6 | SLC44A4 | T1D |
| rs2242665 | 6 | SLC44A4 | T1D |
| rs1270942 | 6 | CFB | T1D |
| rs389884 | 6 | STK19 | T1D |
| rs1150753 | 6 | TNXB | T1D |
| rs1150752 | 6 | TNXB | T1D |
| rs3130320 | 6 | AK123889 | T1D |
| rs11752643 | 6 | HLA-DQB1 | T1D |
| rs3130544 | 6 | C6orf15 | CeD |
| rs3094205 | 6 | PSORS1C1 | CeD |
| rs1966 | 6 | PSORS1C1 | CeD |
| rs130067 | 6 | CCHCR1 | CeD |
| rs7750641 | 6 | TCF19 | CeD |
| rs3873380 | 6 | HLA-B | CeD |
| rs9366778 | 6 | HLA-B | CeD |
| rs3873386 | 6 | HLA-B | CeD |
| rs3094014 | 6 | HCP5 | CeD |
| rs3099844 | 6 | HCG26 | CeD |
| rs3117582 | 6 | APOM | CeD |
| rs3131379 | 6 | MSH5 | CeD |
| rs660594 | 6 | SLC44A4 | CeD |
| rs644827 | 6 | SLC44A4 | CeD |
| rs2242665 | 6 | SLC44A4 | CeD |
| rs659445 | 6 | NG36/G9A | CeD |
| rs1270942 | 6 | CFB | CeD |
| rs437179 | 6 | SKIV2L | CeD |
| rs592229 | 6 | SKIV2L | CeD |
| rs389884 | 6 | STK19 | CeD |
| rs389883 | 6 | STK19 | CeD |
| rs3096700 | 6 | AK123889 | CeD |
| rs3130320 | 6 | AK123889 | CeD |
| rs11752643 | 6 | HLA-DQB1 | CeD |
| rs3130991 | 6 | PSORS1C1 | PSOR |
| rs3094205 | 6 | PSORS1C1 | PSOR |
| rs1966 | 6 | PSORS1C1 | PSOR |
| rs130067 | 6 | CCHCR1 | PSOR |
| rs2524074 | 6 | HLA-B | PSOR |
| rs7754443 | 6 | HLA-B | PSOR |
| rs2524156 | 6 | HLA-B | PSOR |
| rs3873380 | 6 | HLA-B | PSOR |
| rs2524066 | 6 | HLA-B | PSOR |
| rs9366778 | 6 | HLA-B | PSOR |
| rs9461688 | 6 | HLA-B | PSOR |
| rs3873386 | 6 | HLA-B | PSOR |
| rs644045 | 6 | C2 | PSOR |
| rs440454 | 6 | SKIV2L | PSOR |
| rs437179 | 6 | SKIV2L | PSOR |
| rs592229 | 6 | SKIV2L | PSOR |
